# The intramolecular stabilizing effects of *O*-benzoyl substituents as a driving force of the acid-promoted pyranoside-*into*-furanoside rearrangement

**DOI:** 10.3762/bjoc.21.187

**Published:** 2025-11-07

**Authors:** Alexey G Gerbst, Sofya P Nikogosova, Darya A Rastrepaeva, Dmitry A Argunov, Vadim B Krylov, Nikolay E Nifantiev

**Affiliations:** 1 Laboratory of Glycoconjugate Chemistry, N.D. Zelinsky Institute of Organic Chemistry, Russian Academy of Sciences, Leninsky Prospect 47, 119991 Moscow, Russian Federation

**Keywords:** benzoylated galactofuranosides, DFT, DLPNO-CCSD(T), pyranoside-*into*-furanoside rearrangement, stabilizing factors

## Abstract

Furanoside derivatives are broadly present in the antigenic structures of pathogenic microorganisms and play a key role in their recognition by the host immune system. Despite the high demand for vaccine and diagnostic development, their chemical synthesis remains challenging. During the development of a new methodology for the synthesis of galactofuranoside building blocks, we encountered an unexpected predominance of the furanoside form in the equilibrium mixture of benzoylated β-galactosides. Since the furanoside form is typically less stable and is usually present only in minor amounts, we turned to computational studies to elucidate the driving force of this pyranoside-*into*-furanoside isomerisation. The DFT B3LYP-D3 approach was employed for this task with additional validation of its results at DLPNO-CCSD(T) level for the lowest energy conformers. The results demonstrate that the van-der-Waals interactions between phenyl rings of the benzoate substituents are crucial for the stabilization of the furanoside isomer. This outcome could not be rationalized within the framework of conventional carbohydrate chemistry, as the key intramolecular interactions determining the equilibrium lie outside the carbohydrate ring system. Consideration of such effects is essential to rationalize the reactivity of structurally complex and densely protected carbohydrate compounds.

## Introduction

Oligosaccharide chains bearing α-ᴅ-galactofuranosyl (Gal*f*) units are widely distributed in nature. Thus such compounds were discovered in fungi and yeasts [1–3], bacteria [4–7], brown and green seaweeds [8–9], lichens [10], and other species [11]. The presence of Gal*f*-units is often important for the biological activity of these substances. Synthetic oligosaccharides structurally related to natural Gal*f*-containing compounds are required for biochemical investigations and mapping active epitope fragments as well as the design of clinical diagnostics [12–14] and vaccines [15–18]. That is why new efficient and stereoselective methods for the synthesis of both Gal*f*-containing mono- and oligosaccharide derivatives are highly demanded.

**Figure 1:**
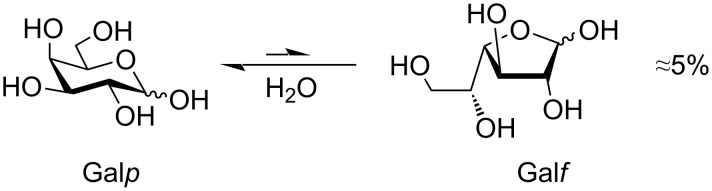
Isomerization of pyranoside and furanoside forms of unprotected ᴅ-galactose in an aqueous solution.

It is a well-known fact that galactofuranose form constitutes only 5% in water solution of unprotected ᴅ-galactose (Figure 1) [19]. The introduction of an alkyl or aryl substituent in the anomeric center prevents free interconversion between pyranose and furanose forms; however, this transformation can still occur under specific conditions, typically in the presence of an acid catalyst. Even under such conditions, the pyranoside form is generally considered to be thermodynamically more stable [20–22].

**Figure 2:**
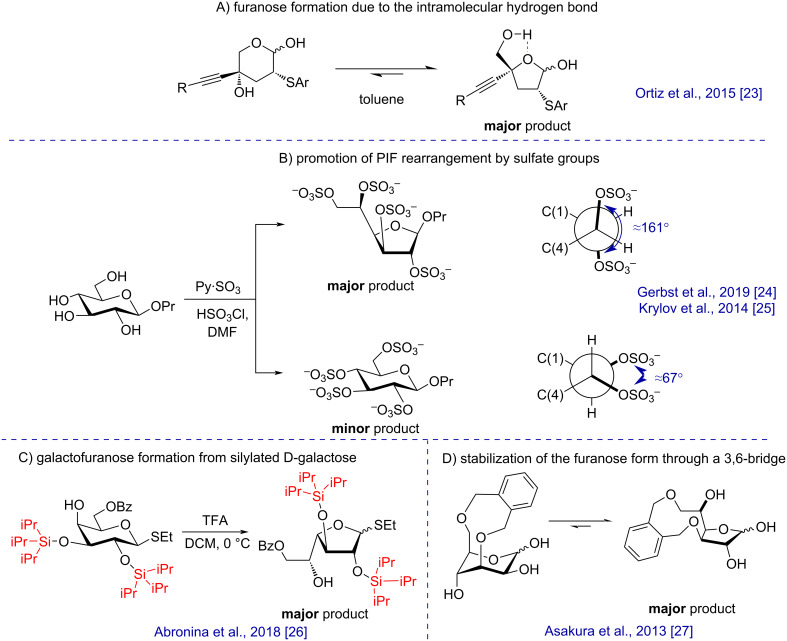
Known approaches for the stabilization of furanose forms (A)–(D).

Only several approaches towards shifting this equilibrium in favor for the galactofuranoside form are known, which are listed below. In one such study [23], the authors demonstrated a shift in the equilibrium towards a five-membered ring due to the formation of an intramolecular hydrogen bond (Figure 2A). It has been shown that furanoside is preferentially formed in nonpolar solvents such as toluene. This form was successfully fixed and purified as benzoate derivative.

Bulky substituents can also stabilize the furanose form through steric and electrostatic effects. In our previous work [24], we investigated the energetic aspects of the pyranoside-*into-*furanoside (PIF) rearrangement that proceeded under acid-promoted sulfation and whose mechanism was studied by us previously [25] along with the mechanism of possible similar transformations for glucosides and mannosides [28]. The stabilization of the furanose form was facilitated by the bulky and charged sulfate groups, leading to repulsive interactions (Figure 2B) [24]. This effect was observed across various configurations, such as *galacto*-, *gluco*-, *fuco*-, *arabino*-, and *xylo*-. However, for both α- and β-mannosides, pyranoside form remains the favorable one.

In another study [26] the presence of two bulky silyl substituents (TIPS or TBDPS) at the O2 and O3 positions of galactose also resulted in stabilization of the furanoside product (Figure 2C). Another example of the furanose form stabilization was demonstrated wherein the introduction of a 3,6-*O*-(*o*-xylylene) bridge locked the mannopyranose in unfavorable conformation, thereby shifting the equilibrium toward the furanoside form (Figure 2D) [27].

Besides the approaches described in Figure 2, transformation of galactopyranosides to galactofuranosides under the action of TsOH in methanol [29] and silica-supported perchloric acid [30] is known. However, in both these cases di-O-isopropylidene ketals were used that underwent removal upon the isomerization leading to the loss of the aglycon.

**Figure 3:**
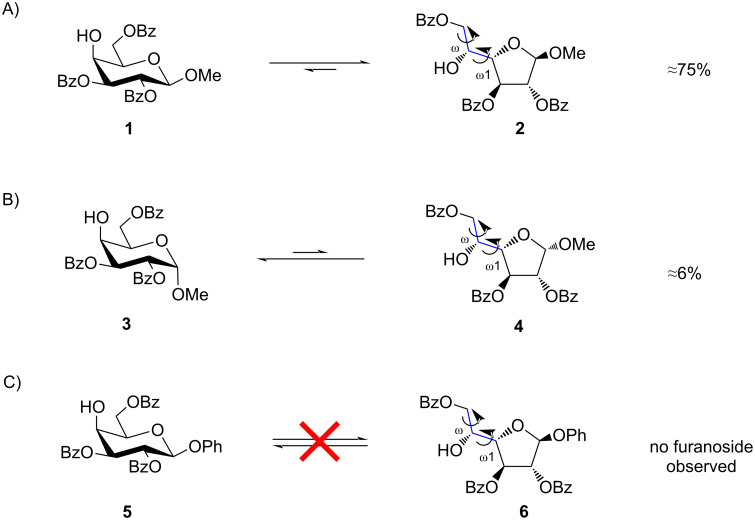
Equilibrium between pyranose and furanose forms in benzoylated derivatives [26]. Conditions: TfOH (cat.) in CD_2_Cl_2_; ratios determined by ^1^H NMR spectroscopy.

Recently, we observed another example of the PIF-rearrangement [31] where treatment with catalytic amounts of TfOH in CH_2_Cl_2_ unexpectedly shifted the equilibrium between selectively protected galactoside **1** and furanoside **2** in favor of the furanose form (Figure 3A), thus opening a new route towards Gal*f*-containing oligosaccharides. Observed prevalence of furanose formation could not be explained by using common concepts of carbohydrate chemistry since the intramolecular interactions that determine the outcome occur outside the carbohydrate cycle. In order to explain the factors regulating the prevalence of furanoside forms in this case, a computational study was carried out. Herein, we report the obtained results.

## Computational Details

All the calculations were performed using the ORCA 5.0.4 software package [32]. DFT B3LYP functional with the third order Grimme’s correction (D3) [33] and def2-TZVP [34] basis set were employed. CPCM model [35] with the default parameters for methylene chloride was used to account for bulk solvent effects. Such choice of computational parameters for protected carbohydrate molecules was validated in our previous investigation [36]. The defgrid3 option was used throughout the calculations. The optimized structures were subjected to hessian calculations and the resulting Gibbs free energies were used for the analysis in this work. For DLPNO-CCSD(T) calculations CC-pVTZ basis set was used. The Altona-Sundaralingam parameters [37–38] for furanoside rings were calculated using the online resource by Shinya Fushinobu [39].

## Results and Discussion

In this study three examples of the pyranoside into furanoside isomerization were examined. The first one represented a case where methyl β-ᴅ-galactoside **1** was involved and the reaction resulted in a considerable amount of the furanose form in the equilibrium mixture (Figure 3A). Other two examples were the cases in which the isomerization to furanoside gave only minute quantities of product in the equilibrium or did not occur at all (Figure 3B and 3C). We used a DFT B3LYP-D3 approach to study possible driving forces of these reactions.

**Figure 4:**
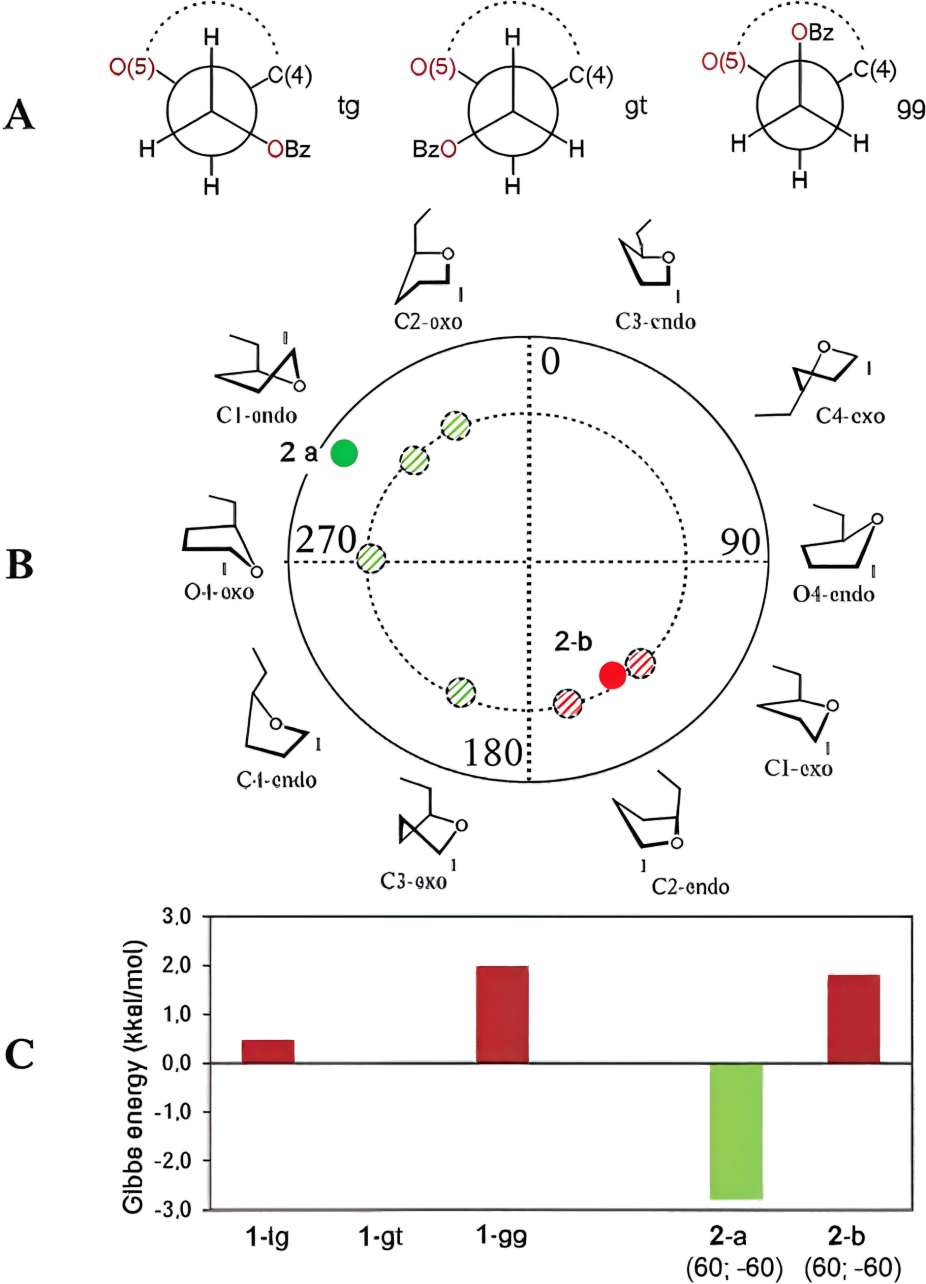
(A) Conformers arising during the rotation around the C5–C6 bond in pyranosides. (B) Furanoside ring conformers of monosaccharide **2** in the pseudorotation diagram. The dashed colored circles denote the initial conformations taken for optimization. Solid colored circles correspond to obtained conformations. (C) Calculated Gibbs free energies for various conformers of monosaccharides **1** and **2**. For conformers **2**-a and **2**-b, the optimal torsional angles ω1 and ω of the side chain are provided in parentheses (as labeled in Figure 3).

First, the energies of starting methyl β-galactopyranoside structure **1** were computed. The initial orientation of the methyl aglycon was chosen so that the torsional angle H1–C1–O1–C(Me) had the value of +40° for the β-structures and −40° for the α-isomers. The benzoate substituents at positions O-2 and O-3 were oriented to have the torsions Hn–Cn–On–CO at 0°. For the benzoate group at O-6 three possible rotamers [40] around the C5–C6 bond were analyzed: *trans-gauche* (*tg*), *gauche-gauche* (*gg*) and *gauche-trans* (*gt*) (Figure 4A). This was done by choosing the initial ω angle (O5–C5–C6–O6) as 180°, −60° or +60° correspondingly, after which the starting structures were fully optimized. Quite expectedly for galactose, the *gt* conformer was found to have the lowest energy. Supporting Information File 1, Table S1 contains the energies obtained for the three conformers and Figure 4C provides graphical representation of the resulting Gibbs energies relative to *gt* (bars 1-tg, 1-gt, 1-gg).

In comparison to pyranosides, furanosides possess more degrees of flexibility. The major difference is of course their potency to adopt a variety of conformations of the sugar ring [38]. This is commonly represented as a pseudorotation ring (Figure 4B). Several of them were chosen as starting conformations for the geometry optimizations. Additionally, the side chain in furanosides has one more rotatable bond, C4–C5. We used angle H4–C4–C5–H5 denoted as ω1 to describe this rotation. In the starting geometries it was also chosen as 180°, −60° or +60°. The rotation around C5–C6 linkage was described by ω angle O5–C5–C6–O6 (the same as in the pyranoside derivatives).

It appeared that the rotation around C5–C6 linkage played a crucial role for determining relative prevalence of either the furanoside or the pyranoside form in case of furanoside structure **2**. Thus, when this angle had the starting values of 180° or −60°, all the calculations led to a conclusion that no transformation to a furanoside should occur (the resulting energies are given in Supporting Information File 1, Table S2). Only when optimizations started at the value of +60° there were produced some conformers with the energy of the furanoside substantially lower than that of the pyranoside form. The energies are provided in Supporting Information File 1, Table S2 and the graphical representation is given in Figure 4C, bars **2**-a and **2**-b, and Figure 4B. The Altona-Sundralingam parameters for C1-*endo* and C1-*exo* conformations of compound **2** are given in Table 1.

**Table 1:** Altona-Sundaralingam parameters for C1-*endo* and C1-*exo* conformers of furanosides **2** and **4**.

Compound, conformer	P, degrees	φ, degrees

**2**, C1-*endo*	297.7	36.6
**2**, C1-*exo*	126.9	−30.6
**4**, C1-*endo*	298.7	39.5
**4**, C1-*exo*	136.7	−31.0

The resulting most stable C1-*endo* conformer of furanoside **2** is in agreement with the ^1^H,^1^H coupling constants that were observed for it previously [26]: ^3^*J*_1,2_ < 1 Hz, ^3^*J*_2,3_ = 1.6 Hz and ^3^*J*_3,4_ = 5.0 Hz. The corresponding torsional angles in the found conformer are measured to be approximately 90°, −101° and 129°.

**Figure 5:**
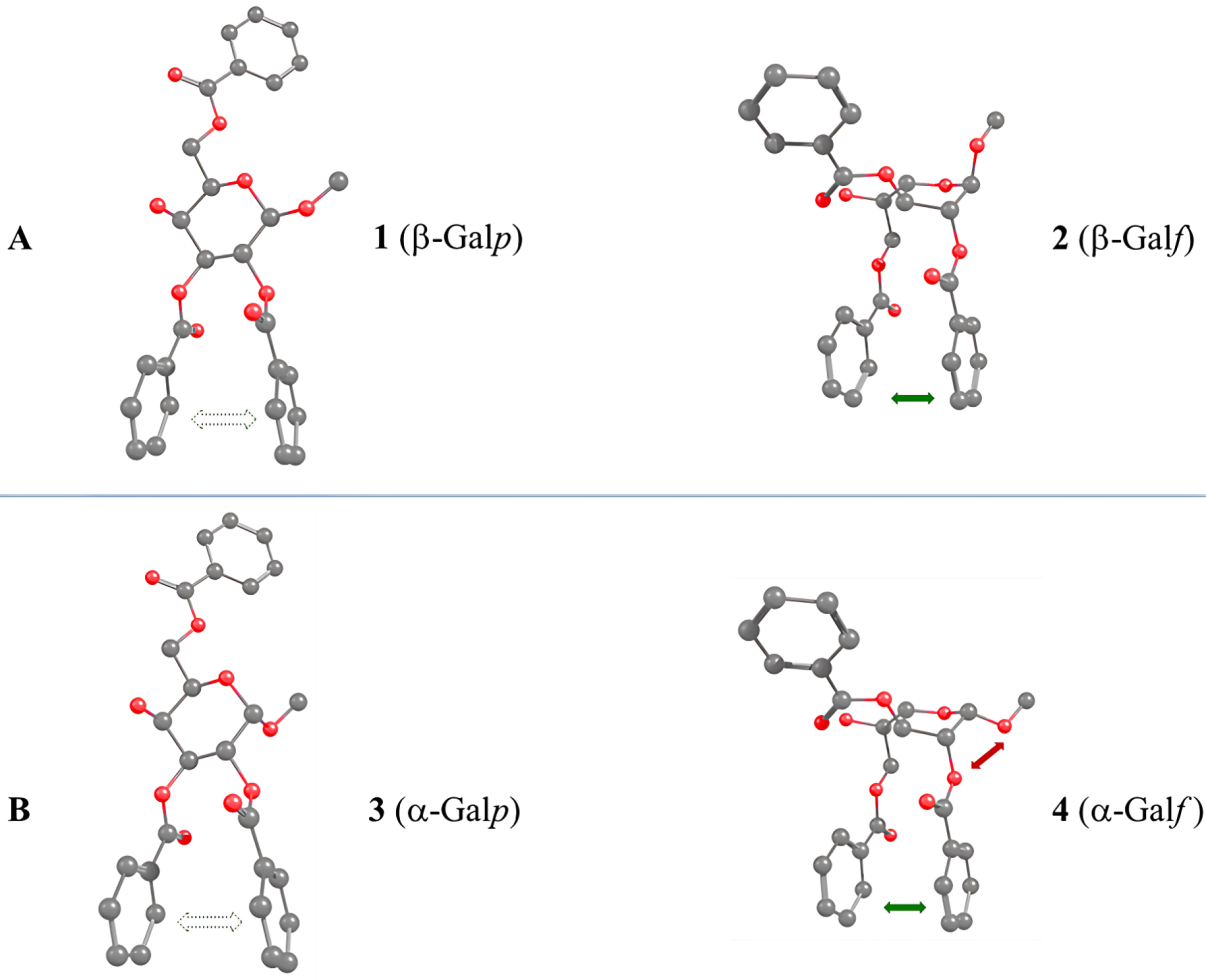
Structures of conformers with minimum energy for the methyl β-ᴅ-galactoside (A) and its α-counterpart (B). Green and red arrows indicate stabilizing and destabilizing spatial interactions, respectively.

A possible reason for this result was determined upon the examination of the obtained structures (graphical representation is given in Figure 5A). It is clearly seen that both the pyranoside and the furanoside forms exhibit π–π interactions between the phenyl rings of some benzoate substituents. These are benzoates at O2 and O3 in the pyranoside and benzoates at O2 and O6 in the furanoside. Obviously the orientation of the C5–C6 in the furanoside greatly influences the possibility of such an interaction in it, thus explaining the fact that when this angle is initially set to +60° the relative energy of the furanoside form decreases making the PIF-rearrangement favorable.

**Figure 6:**
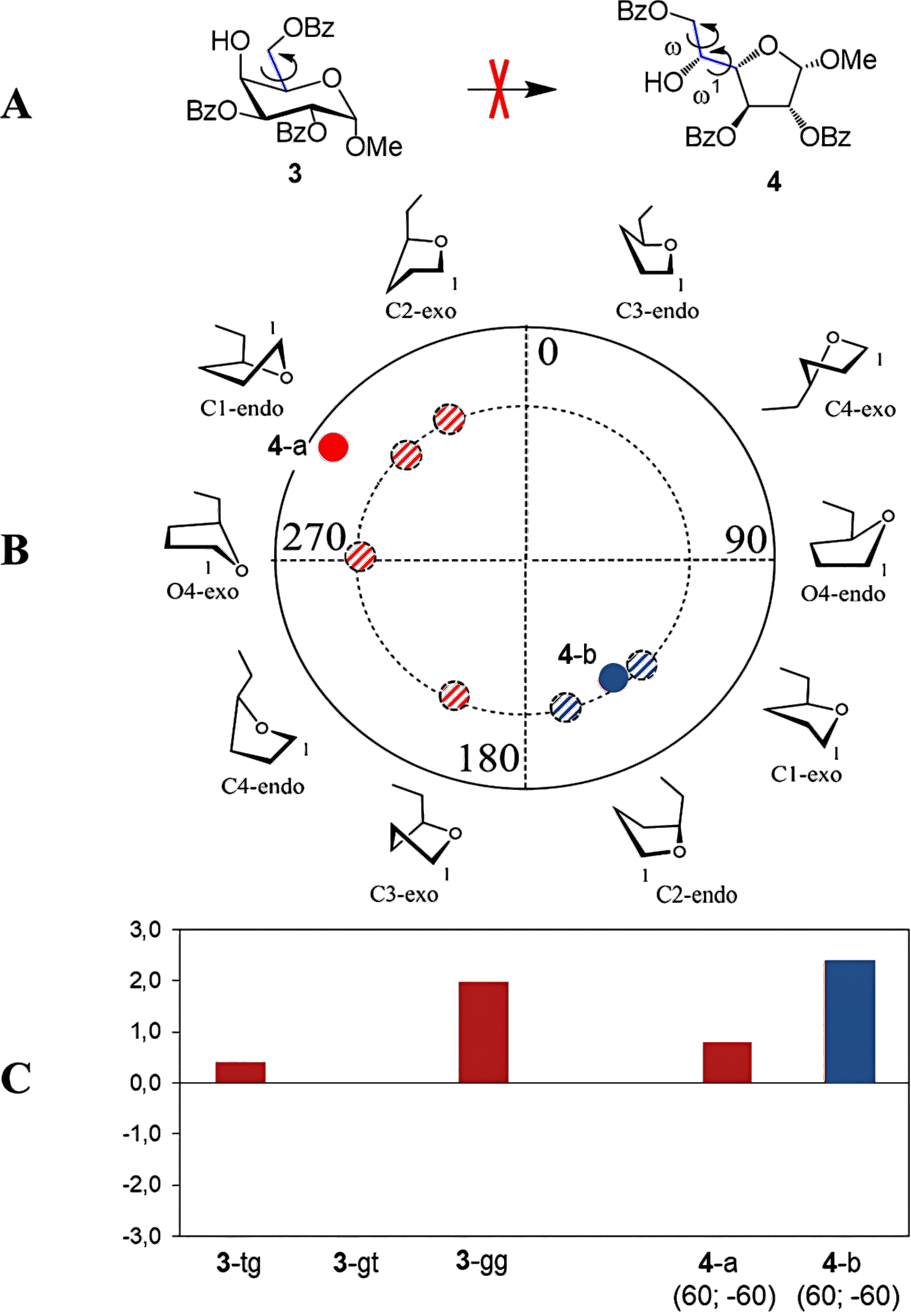
(A) Supposed PIF-rearrangement of monosaccharide **3**. (B) Furanoside ring conformers of monosaccharide **4** in the pseudorotation diagram. The dashed colored circles denote the initial conformations taken for optimization. Solid colored circles correspond to obtained conformations. (C) Calculated Gibbs free energies for various conformers of monosaccharides **3** and **4**. For conformers **4**-a and **4**-b, the optimal torsional angles ω1 and ω of the side chain are provided in parentheses (as labeled in Figure 3).

The described approach was then applied to methyl α-ᴅ-galactosides **3** and **4**. Interestingly, in this case we failed to discover a furanoside conformer with the energy lower than that of the pyranoside. The resulting energies are given in Supporting Information File 1, Tables S3 and S4 while the graphical representation is shown in Figure 6. The Altona-Sundaralingam parameters for C1-*endo* and C1-*exo* conformations of furanoside **4** are given in Table 1.

Looking at the obtained conformations (Figure 5B), one can find that the same π–π interactions are present in the α-galactoside structures as in their β-isomers. However, there is a possibility of another interaction. This is the repulsion between the *cis*-oriented O-1 and O-2 atoms in this case (Figure 5B) which is absent in the pyranoside where these two atoms are *trans-*oriented.

For further analysis of the interactions between the phenyl rings, two parameters were calculated: the distance between the geometrical centers of the involved rings and the angle between the planes formed by these rings. The results are provided in Table 2. It can be seen that for both α- and β-isomers these characteristic significantly decrease in the furanoside form as compared to the pyranoside counterpart. The explanation for this finding obviously lies in the nature of 6- and 5-membered aliphatic rings. The internal C–C–C angles in them differ leading to more freedom for the side substituents in furanosides. This, in our opinion, means that the π–π interactions between the phenyl rings must increase in this case. However, a repulsive interaction that occurs between O-1 and O-2 oxygen atoms in the α-isomer might also be stronger in its furanoside form. The latter fact is responsible for the relative instability of this furanoside as compared to its pyranoside isomer and explains the very low content of furanoside form **4** in the equilibrium mixture with corresponding pyranoside **3**.

**Table 2:** Distances and angles between interacting phenyl rings (Figure 5) in galactosides **1**–**4**.

Compound	Distance, Å	Angle, degrees

**1** (β-Gal*p*)	4.04	12.2
**2** (β-Gal*f*)	3.84	2.8
**3** (α-Gal*p*)	4.02	11.9
**4** (α-Gal*f*)	3.84	3.0

**Figure 7:**
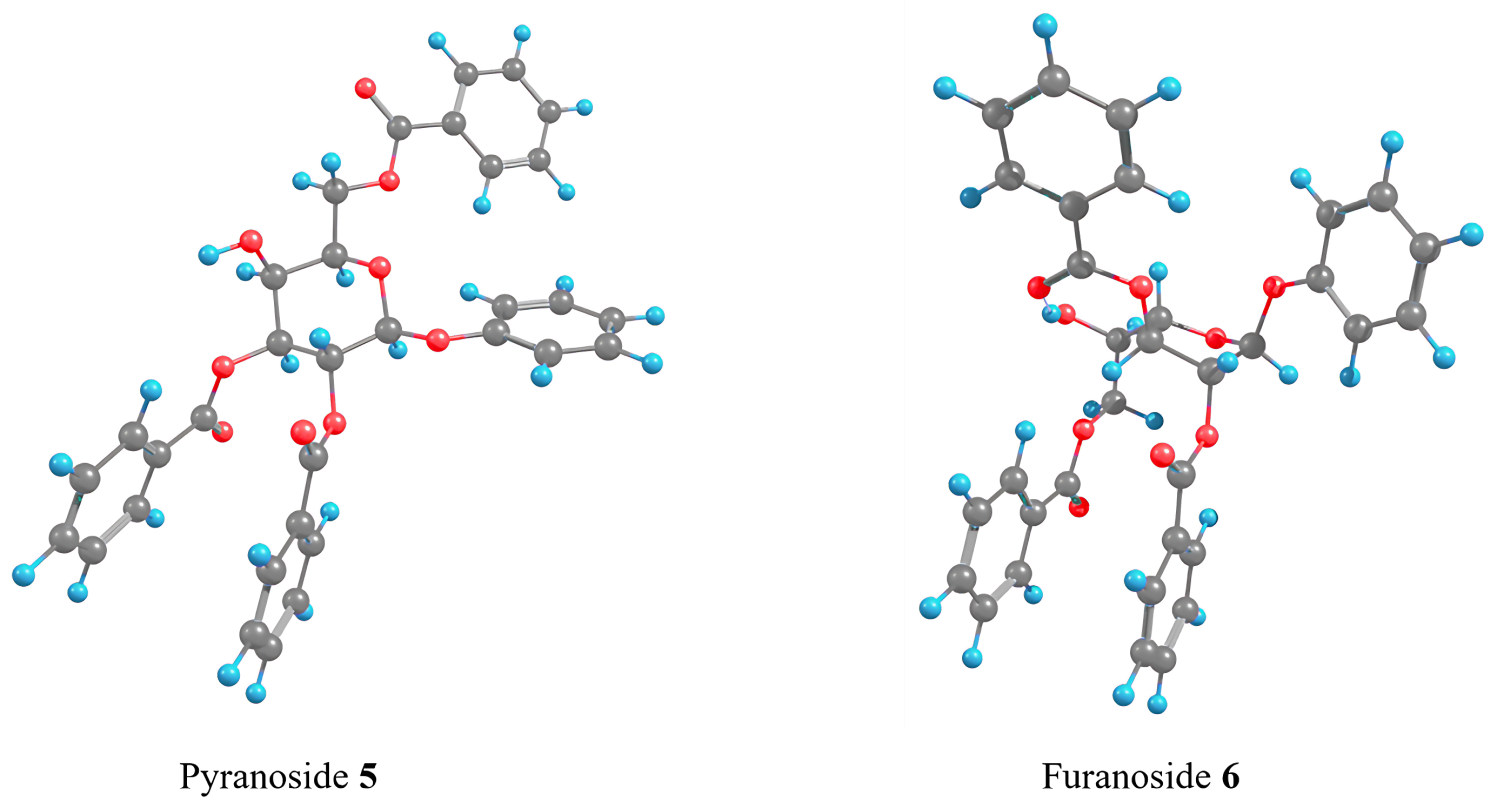
Graphical representations of the lowest energy conformers of phenyl β-ᴅ-galactosides **5** and **6**.

Finally, this approach was applied for the possible transformation phenyl β-ᴅ-galactosides **5** into its furanoside counterpart **6**. For this pair of structures the tendencies observed were the same as for methyl β-ᴅ-galactoside **1**, i.e., among the obtained furanoside conformers were such that had the lower energy than the pyranosides (the energies can be found in Supporting Information File 1, Tables S5 and S6), and the geometries of these conformers were also in accordance with the results obtained for the methyl galactosides. (Figure 7). In the both low energy conformations the π–π interactions described above were observed and no unfavorable interactions were found. All this suggests that the PIF-rearrangement should proceed in this case in a way similar as for compound **1**. However, no traces of furanoside product **6** were found in the experiment [26].

Additionally, single-point DLPNO-CCSD(T) calculations were performed on the conformations with lowest energies found for the studied compounds. For the pyranoside forms these were the *gt* conformers and for the furanoside forms – O4-*exo* ring conformers with the side chain having ω and ω1 torsions with values of −60° and +60°. This was done in order to prove that its results would correlate with those obtained with the DFT approach. From Table 3 it can be seen that, indeed, the energy predictions found with B3LYP-D3 are reproduced on the higher level coupled cluster approximation. For the methyl β-ᴅ-galactosides the furanoside form could be considered preferable while for the α-isomers the preference is for the pyranoside. Again, for the phenyl β-ᴅ-galactosides the DLPNO-CCSD(T) approach predicted prevalence of the furanoside form. However, phenyl group differs from methyl in electronic properties and in size. We suppose that these differences may cause restrictions on the stage of C1–O5 bond breaking which is supposed to be a rate limiting step in the PIF-rearrangement as was previously demonstrated in one of our works [24].

**Table 3:** DLPNO-CCSD(T) calculated energies of the lowest energy conformers for compounds studied in this work. The *gt* conformer of the pyranoside form is taken as 0.

Anomeric configuration and aglycon	Energy of the O4-*exo* furanoside conformer with ω/ω1 angles −60°/+60°, kcal/mol

β, methyl	−4.0
α, methyl	1.3
β, phenyl	−1.9

## Conclusion

A series of galactopyranosides for which the PIF-rearrangement is known to proceed have been studied computationally by means of a DFT approach and the results were confirmed by carrying out DLPNO-CCSD(T) calculations for the lowest energy conformers revealed. It was found that the driving force of this transformation obviously lies in the π–π interactions of the side benzoate substituents: these interactions are stronger in furanosides most probably due to the differences in geometry of 5- and 6-membered aliphatic rings. However, when it comes to the α-galactopyranoside, there might occur a repulsive O1–O2 interaction which is also stronger in the furanoside form. This leads to the increase of its energy and explains why its PIF-rearrangement gives only very low content of the corresponding furanoside in the equilibrium. Unlike this, in the case of phenyl β-ᴅ-galactoside **5** the rearrangement does not proceed at all apparently because of some kinetical reasons since the calculations still suggest prevalence of the furanoside form. Thus it is possible to determine preliminary that the current PIF-rearrangement is regulated by thermodynamics in case of methyl-aglycons and by mechanistic restrictions in case of the phenyl-aglycon.

## Abbreviations

Bz: benzoate; Bn: benzyl; TIPS: triisopropylsilyl; TBDPS: *tert*-butyldiphenylsilyl.

## Supporting Information

File 1 Cartesian coordinates, absolute Gibbs energies.

## Data Availability

All data that supports the findings of this study is available in the published article and/or the supporting information of this article.
