# Supplementary material for: The intramolecular stabilizing effects of O-benzoyl substituents as a driving force of the acid-promoted pyranoside-into-furanoside rearrangement
Source: Beilstein J Org Chem. 2025 Nov 7;21:2456–64. doi: 10.3762/bjoc.21.187 (PMC12599399; doi:10.3762/bjoc.21.187)
Supplement: File 1 — Cartesian coordinates, absolute Gibbs energies. [file Beilstein_J_Org_Chem-21-2456-s001.pdf]

BEILSTEIN JOURNAL OF ORGANIC CHEMISTRY

Supporting Information

for

The intramolecular stabilizing effects of *O*-benzoyl substituents as a driving force of the acid-promoted pyranoside-*into*-furanoside rearrangement

Alexey G. Gerbst, Sofya P. Nikogosova, Darya A. Rastrepaeva, Dmitry A. Argunov, Vadim B. Krylov and Nikolay E. Nifantiev

Beilstein J. Org. Chem. **2025,** *21,* 2456–2464. doi:10.3762/bjoc.21.187

Cartesian coordinates, absolute Gibbs energies

License and Terms: This is a supporting information file under the terms of the Creative Commons Attribution License (https://creativecommons.org/licenses/by/4.0). Please note that the reuse, redistribution and reproduction in particular requires that the author(s) and source are credited and that individual graphics may be subject to special legal provisions.
The license is subject to the *Beilstein Journal of Organic Chemistry* terms and conditions: (https://www.beilstein-journals.org/bjoc/terms)

CONTENTS

Page S1. **Absolute and relative Gibbs energies of the studied structures**
Page S5. **Cartesian coordinates of the optimized conformers of methyl β-D-galactopyranoside 1, Å**
Page S11. **Cartesian coordinates of the optimized conformers of methyl β-D-galactofuranoside 2, Å**
Page S114. **Cartesian coordinates of the optimized conformers of methyl α-D-galactopyranoside 3, Å**
Page S120. **Cartesian coordinates of the optimized conformers of methyl α-D-galactofuranoside 4, Å**
Page S224. **Cartesian coordinates of the optimized conformers of phenyl β-D-galactopyranoside 5, Å**
Page S230. **Cartesian coordinates of the optimized conformers of phenyl β-D-galactopyranoside 6, Å**

Absolute and relative Gibbs energies of the studied structures

Table S1. Absolute and relative Gibbs energies of methyl β-D-galactopyranoside **1**.

Conformer	Absolute Gibbs energy, a.u.	Relative Gibbs energy, kcal/mole
tg	-1759.108803	0.5
gt	-1759.109626	0.0
gg	-1759.106230	2.1

Table S2. Absolute and relative Gibbs energies of methyl β-D-galactofuranoside **2**. *Gauche-trans* conformer of the pyranoside form taken as reference.

Conformer, initial ω1/ω	Absolute Gibbs energy, a.u.	Relative Gibbs energy, kcal/mole
C1-endo, +60°/+60°	-1759.106203	2.1
C1-endo, +60°/-60°	-1759.110666	-0.7
C1-endo, +60°/180°	-1759.105511	2.6
C1-endo, -60°/+60°	-1759.107088	1.6
C1-endo, -60°/-60°	-1759.104207	3.4
C1-endo, -60°/180°	-1759.106545	1.9
C1-endo, 180°/+60°	-1759.103655	3.7
C1-endo, 180°/-60°	-1759.109128	0.3
C1-endo, 180°/180°	-1759.108588	0.7
C2-exo, +60°/+60°	-1759.105384	2.7
C2-exo, +60°/-60°	-1759.110917	-0.8

C2-exo, +60°/180°	-1759.105549	2.6
C2-exo, -60°/+60°	-1759.106275	2.1
C2-exo, -60°/-60°	-1759.103272	3.9
C2-exo, -60°/180°	-1759.104743	3.1
C2-exo, 180°/+60°	-1759.105817	2.4
C2-exo, 180°/-60°	-1759.110759	-0.7
C2-exo, 180°/180°	-1759.107881	1.1
C3-exo, +60°/+60°	-1759.101980	4.8
C3-exo, +60°/-60°	-1759.110816	-0.7
C3-exo, +60°/180°	-1759.105020	2.9
C3-exo, -60°/+60°	-1759.106001	2.3
C3-exo, -60°/-60°	-1759.104160	3.4
C3-exo, -60°/180°	-1759.105873	2.4
C3-exo, 180°/+60°	-1759.105602	0.4
C3-exo, 180°/-60°	-1759.109108	0.3
C3-exo, 180°/180°	-1759.108864	0.5
O4-exo, +60°/+60°	-1759.105168	2.8
O4-exo, +60°/-60°	-1759.114060	-2.8
O4-exo, +60°/180°	-1759.107118	1.6
O4-exo, -60°/+60°	-1759.107645	1.2
O4-exo, -60°/-60°	-1759.107277	1.5
O4-exo, -60°/180°	-1759.108023	1.0
O4-exo, 180°/+60°	-1759.104139	3.4
O4-exo, 180°/-60°	-1759.107978	1.0
O4-exo, 180°/180°	-1759.108883	0.5
C2-endo, +60°/+60°	-1759.105727	2.4
C2-endo, +60°/-60°	-1759.106514	2.0
C2-endo, +60°/180°	-1759.101697	5.0
C2-endo, -60°/+60°	-1759.102981	4.2
C2-endo, -60°/-60°	-1759.104125	3.5
C2-endo, -60°/180°	-1759.102511	4.5
C2-endo, 180°/+60°	-1759.105160	2.8
C2-endo, 180°/-60°	-1759.109443	0.1
C2-endo, 180°/180°	-1759.109040	0.4
C1-exo, +60°/+60°	-1759.102190	4.7
C1-exo, +60°/-60°	-1759.106726	1.8
C1-exo, +60°/180°	-1759.100703	5.6
C1-exo, -60°/+60°	-1759.106370	2.0
C1-exo, -60°/-60°	-1759.104306	3.3
C1-exo, -60°/180°	-1759.106562	1.9
C1-exo, 180°/+60°	-1759.105344	2.7
C1-exo, 180°/-60°	-1759.106738	1.8
C1-exo, 180°/180°	-1759.102840	4.3

Table S3. Absolute and relative Gibbs energies of methyl α-D- galactopyranoside **3**.

Conformer	Absolute Gibbs energy, a.u.	Relative Gibbs energy, kcal/mole

tg	-1759.110031	0.4
gt	-1759.110685	0.0
gg	-1759.107535	2.0

Table S4. Absolute and relative Gibbs energies of methyl α-D- galactofuranoside **4**. *Gauche-trans* conformer of the pyranoside form taken as reference.

Conformer, initial ω1/ω	Absolute Gibbs energy, a.u.	Relative Gibbs energy, kcal/mole
C1-endo, +60°/+60°	-1759.102193	5.3
C1-endo, +60°/-60°	-1759.106087	2.9
C1-endo, +60°/180°	-1759.101960	5.5
C1-endo, -60°/+60°	-1759.102026	5.4
C1-endo, -60°/-60°	-1759.099705	6.9
C1-endo, -60°/180°	-1759.101197	6.0
C1-endo, 180°/+60°	-1759.104897	3.6
C1-endo, 180°/-60°	-1759.104025	4.2
C1-endo, 180°/180°	-1759.104134	4.1
C2-exo, +60°/+60°	-1759.101515	5.8
C2-exo, +60°/-60°	-1759.106237	2.8
C2-exo, +60°/180°	-1759.101504	5.8
C2-exo, -60°/+60°	-1759.100663	6.3
C2-exo, -60°/-60°	-1759.103275	4.6
C2-exo, -60°/180°	-1759.101025	6.1
C2-exo, 180°/+60°	-1759.102834	4.9
C2-exo, 180°/-60°	-1759.105689	3.1
C2-exo, 180°/180°	-1759.105349	3.3
C3-exo, +60°/+60°	-1759.103514	4.5
C3-exo, +60°/-60°	-1759.106072	2.9
C3-exo, +60°/180°	-1759.102342	5.2
C3-exo, -60°/+60°	-1759.104931	3.6
C3-exo, -60°/-60°	-1759.099741	6.9
C3-exo, -60°/180°	-1759.104283	4.0
C3-exo, 180°/+60°	-1759.104756	3.7
C3-exo, 180°/-60°	-1759.103986	4.2
C3-exo, 180°/180°	-1759.104639	3.8
O4-exo, +60°/+60°	-1759.100880	6.2
O4-exo, +60°/-60°	-1759.109424	0.8
O4-exo, +60°/180°	-1759.101804	5.6
O4-exo, -60°/+60°	-1759.103211	4.7
O4-exo, -60°/-60°	-1759.103480	4.5
O4-exo, -60°/180°	-1759.104069	4.2
O4-exo, 180°/+60°	-1759.100481	6.4
O4-exo, 180°/-60°	-1759.105628	3.2
O4-exo, 180°/180°	-1759.104816	3.7
C2-endo, +60°/+60°	-1759.103419	4.6
C2-endo, +60°/-60°	-1759.106514	2.6
C2-endo, +60°/180°	-1759.101090	6.0
C2-endo, -60°/+60°	-1759.104704	3.8
C2-endo, -60°/-60°	-1759.101959	5.5

C2-endo, -60°/180°	-1759.103964	4.2
C2-endo, 180°/+60°	-1759.104742	3.7
C2-endo, 180°/-60°	-1759.108837	1.2
C2-endo, 180°/180°	-1759.107125	2.2
C1-exo, +60°/+60°	-1759.103779	4.3
C1-exo, +60°/-60°	-1759.106901	2.4
C1-exo, +60°/180°	-1759.101221	5.9
C1-exo, -60°/+60°	-1759.104803	3.7
C1-exo, -60°/-60°	-1759.102612	5.1
C1-exo, -60°/180°	-1759.104064	4.2
C1-exo, 180°/+60°	-1759.104917	3.6
C1-exo, 180°/-60°	-1759.108870	1.1
C1-exo, 180°/180°	-1759.107084	2.3

Table S5. Absolute and relative Gibbs energies of phenyl β-D-–galactopyranoside **5**.

Conformer	Absolute Gibbs energy, a.u.	Relative Gibbs energy, kcal/mole
tg	-1950.773263	1.0
gt	-1950.774828	0.0
gg	-1950.770704	2.6

Table S6. Absolute and relative Gibbs energies of phenyl β-D-galactofuranoside **6**. *Gauche-trans* conformer of the pyranoside form taken as reference.

Conformer, initial ω1/ω	Absolute Gibbs energy, a.u.	Relative Gibbs energy, kcal/mole
C1-endo, +60°/+60°	-1950.770196	2.9
C1-endo, +60°/-60°	-1950.775144	-0.2
C1-endo, +60°/180°	-1950.770005	3.0
C1-endo, -60°/+60°	-1950.771284	2.2
C1-endo, -60°/-60°	-1950.770118	3.0
C1-endo, -60°/180°	-1950.773564	0.8
C1-endo, 180°/+60°	-1950.769118	3.6
C1-endo, 180°/-60°	-1950.773159	1.0
C1-endo, 180°/180°	-1950.771557	2.1
C2-exo, +60°/+60°	-1950.768323	4.1
C2-exo, +60°/-60°	-1950.775193	-0.2
C2-exo, +60°/180°	-1950.770132	2.9
C2-exo, -60°/+60°	-1950.770443	2.8
C2-exo, -60°/-60°	-1950.765388	5.9
C2-exo, -60°/180°	-1950.772946	1.2
C2-exo, 180°/+60°	-1950.770557	2.7
C2-exo, 180°/-60°	-1950.773773	0.7
C2-exo, 180°/180°	-1950.772482	1.5

C3-exo, +60°/+60°	-1950.765878	5.6
C3-exo, +60°/-60°	-1950.774290	0.3
C3-exo, +60°/180°	-1950.769955	3.1
C3-exo, -60°/+60°	-1950.770138	2.9
C3-exo, -60°/-60°	-1950.771266	2.2
C3-exo, -60°/180°	-1950.773432	0.9
C3-exo, 180°/+60°	-1950.769465	3.4
C3-exo, 180°/-60°	-1950.773362	0.9
C3-exo, 180°/180°	-1950.772841	1.2
O4-exo, +60°/+60°	-1950.769289	3.5
O4-exo, +60°/-60°	-1950.777145	-1.5
O4-exo, +60°/180°	-1950.771609	2.0
O4-exo, -60°/+60°	-1950.772073	1.7
O4-exo, -60°/-60°	-1950.774371	0.3
O4-exo, -60°/180°	-1950.775305	-0.3
O4-exo, 180°/+60°	-1950.768576	3.9
O4-exo, 180°/-60°	-1950.772729	1.3
O4-exo, 180°/180°	-1950.773085	1.1
C2-endo, +60°/+60°	-1950.766568	5.2
C2-endo, +60°/-60°	-1950.770912	2.5
C2-endo, +60°/180°	-1950.769776	3.2
C2-endo, -60°/+60°	-1950.768686	3.9
C2-endo, -60°/-60°	-1950.771002	2.4
C2-endo, -60°/180°	-1950.771006	2.4
C2-endo, 180°/+60°	-1950.769052	3.6
C2-endo, 180°/-60°	-1950.774109	0.5
C2-endo, 180°/180°	-1950.772986	1.2
C1-exo, +60°/+60°	-1950.766426	5.3
C1-exo, +60°/-60°	-1950.770870	2.5
C1-exo, +60°/180°	-1950.766402	5.3
C1-exo, -60°/+60°	-1950.770150	2.9
C1-exo, -60°/-60°	-1950.771209	2.3
C1-exo, -60°/180°	-1950.769034	3.6
C1-exo, 180°/+60°	-1950.769080	3.6
C1-exo, 180°/-60°	-1950.773294	1.0
C1-exo, 180°/180°	-1950.767994	4.3

Cartesian coordinates of the optimized conformers, Å. Starting ring geometries and values of ω1/ω torsions before the geometry optimization are given for furanosides.

Methyl β-D-Gal*p* **1**, gg

	X	Y	Z
O	1.95870	0.98060	0.26280
C	2.81070	0.69590	1.28020
O	2.43820	0.33030	2.37110

C	4.22940	0.84240	0.88060
C	4.59870	1.22830	-0.41030
C	5.94100	1.27820	-0.75920
C	6.91760	0.93850	0.17230
C	6.55220	0.55300	1.45960
C	5.21300	0.50700	1.81430
H	3.83860	1.47020	-1.13810
H	6.22540	1.57280	-1.76100
H	7.96350	0.96900	-0.10600
H	7.31120	0.28180	2.18180
H	4.91440	0.19530	2.80540
O	1.49000	-1.69930	-0.16930
O	-1.76700	-0.25530	-0.98060
O	-2.88820	2.13900	-1.77650
C	-0.85900	-1.29520	-0.62770
C	0.55060	-0.72080	-0.62910
C	0.62470	0.45510	0.33760
C	-0.39010	1.53020	-0.03170
C	-1.76150	0.84590	-0.07590
C	-2.93940	1.73740	-0.39970
H	0.80400	-0.41230	-1.64000
H	0.43630	0.11330	1.35450
H	-1.94710	0.48190	0.94500
C	2.64090	-1.84780	-0.86860
C	-3.84480	2.97630	-2.19490
O	-0.08260	2.13240	-1.27820
H	-3.86740	1.19160	-0.22730
H	-2.92690	2.61740	0.24430
O	-0.93450	-2.29360	-1.57680
H	-1.12390	-1.67830	0.37090
C	-2.11740	-3.09330	-1.49300
H	-0.41260	2.28260	0.76480
H	0.80940	2.49860	-1.21710
H	-2.21770	-3.52120	-0.49100
H	-2.00340	-3.89220	-2.22200

H	-3.00550	-2.50470	-1.72940
O	2.78900	-1.44230	-1.99850
C	3.69820	-2.51350	-0.07060
C	3.48030	-2.93640	1.24280
C	4.52380	-3.48580	1.97470
C	5.78730	-3.60950	1.40450
C	6.00740	-3.18870	0.09550
C	4.96640	-2.64470	-0.64100
H	2.50440	-2.82100	1.69030
H	4.35380	-3.80920	2.99350
H	6.60170	-4.02910	1.98150
H	6.99140	-3.27820	-0.34600
H	5.12700	-2.30040	-1.65300
C	-3.70210	3.32510	-3.63240
O	-4.72260	3.39750	-1.46930
C	-4.64880	4.17610	-4.20640
C	-4.54880	4.52720	-5.54500
C	-3.50210	4.03120	-6.31800
C	-2.55630	3.18260	-5.74950
C	-2.65330	2.82800	-4.41050
H	-5.45660	4.55450	-3.59510
H	-5.28500	5.18660	-5.98640
H	-3.42380	4.30580	-7.36240
H	-1.74250	2.79700	-6.35010
H	-1.92020	2.17040	-3.96650

Methyl β-D-Gal*p* **1**, gt

	**X**	**Y**	**Z**
O	1.95980	0.99040	0.31540
C	2.78730	0.67410	1.34360
O	2.38780	0.30340	2.42330
C	4.21570	0.79370	0.97010
C	4.61530	1.18160	-0.31110
C	5.96410	1.20430	-0.63710
C	6.91700	0.83580	0.30780

C	6.52150	0.44840	1.58560
C	5.17570	0.42920	1.91730
H	3.87310	1.44600	-1.04940
H	6.27170	1.50020	-1.63160
H	7.96790	0.84500	0.04720
H	7.26200	0.15480	2.31810
H	4.85390	0.11610	2.90060
O	1.45210	-1.67680	-0.16180
O	-1.74300	-0.14670	-1.04360
O	-4.08320	1.27180	-0.41540
C	-0.87990	-1.21480	-0.66830
C	0.54240	-0.67440	-0.63000
C	0.61650	0.48570	0.35610
C	-0.37460	1.58460	-0.00300
C	-1.75700	0.93600	-0.11500
C	-2.80670	1.91880	-0.57180
H	0.82510	-0.35760	-1.63050
H	0.40280	0.12870	1.36300
H	-2.02920	0.56230	0.88050
C	2.61250	-1.84420	-0.84070
C	-5.16740	1.97980	-0.76250
O	-0.05670	2.21310	-1.23590
H	-2.77590	2.82050	0.03990
H	-2.66180	2.19000	-1.61570
O	-0.95890	-2.20880	-1.62200
H	-1.18280	-1.59230	0.32150
C	-2.16920	-2.96970	-1.57210
H	-0.39790	2.31880	0.80940
H	0.81600	2.61860	-1.15310
H	-2.30980	-3.39630	-0.57440
H	-2.06230	-3.77020	-2.30040
H	-3.03120	-2.35160	-1.82920
O	2.79100	-1.43170	-1.96360
C	3.63950	-2.54090	-0.02960
C	3.38850	-2.96920	1.27600

C	4.40590	-3.54860	2.02150
C	5.67600	-3.69770	1.47250
C	5.92900	-3.27160	0.17110
C	4.91430	-2.69710	-0.57880
H	2.40790	-2.83430	1.70740
H	4.21040	-3.87600	3.03440
H	6.47000	-4.14110	2.06000
H	6.91830	-3.38090	-0.25390
H	5.10070	-2.34830	-1.58480
C	-6.42620	1.20550	-0.60620
O	-5.11020	3.12520	-1.15870
C	-7.63740	1.85050	-0.86500
C	-8.83510	1.16210	-0.73560
C	-8.83030	-0.17590	-0.34990
C	-7.62510	-0.82360	-0.09340
C	-6.42470	-0.13720	-0.21920
H	-7.62750	2.88910	-1.16590
H	-9.77160	1.66640	-0.93550
H	-9.76490	-0.71340	-0.25020
H	-7.62090	-1.86420	0.20430
H	-5.48840	-0.63910	-0.02270

Methyl β-D-Gal*p* **1**, tg

	**X**	**Y**	**Z**
O	1.90700	1.03350	0.18090
C	2.73880	0.81150	1.22940
O	2.34620	0.50920	2.33280
C	4.16500	0.93930	0.84940
C	4.55800	1.25510	-0.45340
C	5.90650	1.28860	-0.77960
C	6.86590	1.00160	0.18690
C	6.47690	0.68600	1.48620
C	5.13130	0.65710	1.81800
H	3.81130	1.45590	-1.20710

H	6.20920	1.52900	-1.79050
H	7.91670	1.01850	-0.07340
H	7.22250	0.45530	2.23590
H	4.81480	0.39890	2.81880
O	1.46080	-1.67180	-0.10600
O	-1.77800	-0.28950	-1.07820
O	-2.99520	2.88610	0.06960
C	-0.88050	-1.30510	-0.63490
C	0.52750	-0.72660	-0.64120
C	0.57450	0.50370	0.25690
C	-0.44000	1.54710	-0.18760
C	-1.80820	0.86070	-0.23700
C	-2.90490	1.74440	-0.79790
H	0.80020	-0.47620	-1.66300
H	0.36630	0.21830	1.28760
H	-2.06920	0.56680	0.78760
C	2.62630	-1.85570	-0.77190
C	-3.87320	3.84130	-0.27460
O	-0.13950	2.07280	-1.47180
H	-2.66580	2.06590	-1.80960
H	-3.85310	1.20800	-0.80350
O	-0.93440	-2.36320	-1.51760
H	-1.17050	-1.62070	0.37980
C	-2.11600	-3.16140	-1.40450
H	-0.47690	2.34700	0.55750
H	0.71490	2.52000	-1.42360
H	-2.23260	-3.52580	-0.37950
H	-1.98620	-4.00410	-2.07950
H	-3.00170	-2.59280	-1.69320
O	2.79590	-1.51770	-1.92080
C	3.66930	-2.46770	0.08580
C	3.42290	-2.82230	1.41430
C	4.45240	-3.32280	2.19910
C	5.73040	-3.46490	1.66680
C	5.97910	-3.11170	0.34300

C	4.95210	-2.61730	-0.44630
H	2.43550	-2.69240	1.83160
H	4.26010	-3.59370	3.22910
H	6.53380	-3.84610	2.28450
H	6.97430	-3.21510	-0.06940
H	5.13470	-2.32550	-1.47090
C	-3.86430	4.98880	0.66840
O	-4.58600	3.75900	-1.25200
C	-4.74920	6.04360	0.43490
C	-4.77080	7.13690	1.28870
C	-3.90870	7.18350	2.38120
C	-3.02490	6.13450	2.61770
C	-3.00060	5.03840	1.76570
H	-5.41310	5.99540	-0.41740
H	-5.45840	7.95210	1.10400
H	-3.92560	8.03680	3.04740
H	-2.35410	6.17090	3.46640
H	-2.31440	4.22410	1.94820

Methyl β-D-Gal*f* **2**,C1-endo, +60°/+60°

	**X**	**Y**	**Z**
C	2.46480	2.86100	-4.05170
O	2.00940	4.11520	-4.52290
C	1.31330	2.32770	-3.20930
C	0.71430	3.59800	-2.58820
C	1.21110	4.73490	-3.50480
O	3.57780	2.96960	-3.21250
H	2.68920	2.25490	-4.93280
O	1.24730	3.78000	-1.27210
C	4.74660	3.45600	-3.87230
H	4.99990	2.81650	-4.72410
H	5.55190	3.42730	-3.14180
H	4.60400	4.48110	-4.22000
H	1.62380	1.60740	-2.45890

O	0.38400	1.73880	-4.12680
C	-0.57360	0.94250	-3.60290
C	0.39040	3.81050	-0.22870
H	-0.36650	3.54250	-2.52310
C	0.12760	5.56490	-4.18260
C	-0.82090	4.75050	-5.04160
H	0.63070	6.29080	-4.83200
O	-0.56080	6.23780	-3.13410
O	-1.70440	5.71400	-5.65320
H	-0.28310	4.20330	-5.81370
H	-1.41520	4.05290	-4.45250
H	-1.26850	6.76470	-3.52680
H	1.82270	5.41270	-2.90530
C	-2.68080	5.23710	-6.44120
C	-3.54460	6.31210	-6.99340
C	-3.32350	7.66140	-6.70520
C	-4.15780	8.63130	-7.24470
C	-5.21480	8.26190	-8.07170
C	-5.43820	6.91810	-8.36050
C	-4.60640	5.94620	-7.82370
H	-2.50300	7.94770	-6.06340
H	-3.98400	9.67570	-7.02000
H	-5.86390	9.02040	-8.49080
H	-6.26000	6.62990	-9.00310
H	-4.76970	4.89940	-8.03990
C	1.10020	3.96990	1.06510
C	0.33570	4.04940	2.23140
C	0.95750	4.19440	3.46280
C	2.34660	4.25980	3.53720
C	3.11220	4.17980	2.37750
C	2.49390	4.03550	1.14270
H	-0.74190	3.99590	2.15980
H	0.36160	4.25600	4.36420
H	2.83180	4.37230	4.49860
H	4.19180	4.22930	2.43550

H	3.08660	3.97070	0.24190
C	-1.49720	0.41900	-4.63670
C	-1.37010	0.76300	-5.98470
C	-2.52490	-0.43640	-4.23160
C	-3.41540	-0.94420	-5.16570
C	-3.28570	-0.59970	-6.50900
C	-2.26430	0.25340	-6.91590
H	-0.58220	1.43150	-6.29850
H	-2.61560	-0.69380	-3.18530
H	-4.21060	-1.60630	-4.84880
H	-3.98230	-0.99460	-7.23780
H	-2.16750	0.52610	-7.95870
O	-2.82600	4.05470	-6.66640
O	-0.81070	3.72100	-0.34640
O	-0.64740	0.70110	-2.41870

Methyl β-D-Gal*f* **2**,C1-endo, +60°/-60°

	**X**	**Y**	**Z**
C	0.04910	3.15180	-1.53550
O	0.55080	3.82520	-2.67380
C	-1.35110	3.71890	-1.34320
C	-1.22030	5.17040	-1.82790
C	0.09610	5.18380	-2.63380
O	0.77410	3.43950	-0.37420
H	0.07150	2.08350	-1.76350
O	-1.10230	6.04770	-0.70120
C	2.12010	2.96460	-0.40760
H	2.14120	1.88560	-0.59150
H	2.55030	3.17710	0.56850
H	2.69790	3.47400	-1.18140
H	-1.70350	3.65780	-0.31820
O	-2.20980	2.97590	-2.21740
C	-3.53850	3.08510	-2.00540
C	-2.00940	7.03720	-0.56530
H	-2.07530	5.46660	-2.41800

C	0.03420	5.70510	-4.06680
C	-0.87120	4.94750	-5.02380
H	1.04550	5.58800	-4.47650
O	-0.30340	7.08190	-3.95770
O	-2.24910	5.16560	-4.67140
H	-0.70830	5.30590	-6.04120
H	-0.64770	3.88120	-4.99040
H	-0.30470	7.47730	-4.83810
H	0.81400	5.80510	-2.09260
C	-3.17500	4.67490	-5.51050
C	-4.55900	4.98850	-5.07710
C	-4.82230	5.77700	-3.95450
C	-6.13470	6.02520	-3.57760
C	-7.18730	5.49510	-4.31760
C	-6.92680	4.71520	-5.44070
C	-5.61750	4.46190	-5.81940
H	-4.01230	6.19200	-3.37360
H	-6.33520	6.63010	-2.70280
H	-8.21000	5.68680	-4.01770
H	-7.74450	4.29660	-6.01290
H	-5.40040	3.84730	-6.68160
C	-1.76810	7.84810	0.65410
C	-2.61060	8.93460	0.90090
C	-2.42230	9.71930	2.02920
C	-1.39260	9.42290	2.91860
C	-0.55160	8.34030	2.67700
C	-0.73560	7.55310	1.54820
H	-3.40660	9.15340	0.20250
H	-3.07670	10.56090	2.21640
H	-1.24600	10.03530	3.79950
H	0.24810	8.10940	3.36890
H	-0.08480	6.71170	1.36000
C	-4.33080	2.30900	-2.98700
C	-3.72800	1.61480	-4.03870
C	-5.72060	2.30150	-2.85510

C	-6.50020	1.60420	-3.76420
C	-5.89780	0.91480	-4.81280
C	-4.51310	0.92240	-4.94950
H	-2.65380	1.63040	-4.14900
H	-6.17690	2.85440	-2.04630
H	-7.57760	1.60810	-3.66440
H	-6.50810	0.37770	-5.52790
H	-4.04520	0.39410	-5.76990
O	-2.89080	4.04530	-6.50780
O	-2.90900	7.23470	-1.35160
O	-4.00480	3.75080	-1.10760

Methyl β-D-Gal*f* **2**,C1-endo, +60°/180°

	**X**	**Y**	**Z**
C	3.01160	5.78160	-6.18230
O	1.84550	6.35130	-5.62670
C	2.96120	4.31510	-5.77310
C	2.27200	4.35440	-4.40150
C	1.57500	5.72950	-4.36420
O	4.18850	6.31930	-5.64660
H	2.95940	5.94280	-7.26210
O	3.26950	4.28910	-3.37600
C	4.37480	7.70080	-5.95220
H	4.36150	7.85900	-7.03560
H	5.34790	7.98040	-5.55470
H	3.60000	8.31460	-5.48860
H	3.94060	3.84880	-5.72360
O	2.13920	3.65400	-6.74300
C	2.13060	2.30450	-6.71530
C	3.15900	3.33280	-2.42960
H	1.58790	3.52340	-4.27430
C	0.06720	5.71270	-4.12060
C	-0.76170	4.78970	-5.00670
H	-0.29310	6.73810	-4.25730
O	-0.08110	5.29810	-2.76530

O	-0.75340	5.30970	-6.34340
H	-0.38160	3.76820	-5.00880
H	-1.78730	4.76670	-4.63350
H	-1.00380	5.40620	-2.50240
H	2.02330	6.31430	-3.55730
C	-1.47130	4.64740	-7.25980
C	-1.30730	5.22130	-8.61910
C	-0.32250	6.17270	-8.89550
C	-0.16630	6.65000	-10.18990
C	-0.99570	6.18930	-11.20890
C	-1.98250	5.24630	-10.93340
C	-2.13500	4.75970	-9.64320
H	0.32370	6.52010	-8.10220
H	0.60360	7.37970	-10.40510
H	-0.87200	6.56350	-12.21740
H	-2.62700	4.88730	-11.72530
H	-2.88680	4.01560	-9.41900
C	4.27930	3.38570	-1.45720
C	4.27760	2.47480	-0.39840
C	5.30710	2.48660	0.53150
C	6.34480	3.40700	0.40890
C	6.35070	4.31580	-0.64550
C	5.32160	4.30860	-1.57750
H	3.46650	1.76450	-0.31550
H	5.30200	1.77950	1.35100
H	7.14850	3.41560	1.13430
H	7.15770	5.03070	-0.74140
H	5.32510	5.01260	-2.39680
C	1.28430	1.72580	-7.78480
C	0.81610	2.49610	-8.85110
C	0.96230	0.36830	-7.71250
C	0.17060	-0.20990	-8.69350
C	-0.29400	0.56100	-9.75680
C	0.03210	1.91120	-9.83570
H	1.06530	3.54470	-8.90900

H	1.33100	-0.21850	-6.88230
H	-0.08570	-1.25960	-8.63070
H	-0.91090	0.10830	-10.52280
H	-0.33020	2.51210	-10.65870
O	-2.16350	3.68670	-6.99510
O	2.25010	2.53490	-2.38710
O	2.74880	1.66260	-5.89530

Methyl β-D-Gal*f* **2**,C1-endo, -60°/+60°

	**X**	**Y**	**Z**
C	1.89150	2.43590	-3.15590
O	1.36690	3.36210	-4.08330
C	1.55570	3.02400	-1.79120
C	1.63070	4.53580	-2.02850
C	1.53620	4.69090	-3.56280
O	3.28560	2.32710	-3.22600
H	1.40880	1.47630	-3.35670
O	2.89840	4.98450	-1.52610
C	3.75420	1.78540	-4.46080
H	3.30450	0.80410	-4.64420
H	4.83280	1.68080	-4.36660
H	3.52230	2.45010	-5.29530
H	2.23220	2.69910	-1.00660
O	0.21290	2.62310	-1.49690
C	-0.22560	2.83420	-0.23960
C	3.05020	6.30980	-1.34920
H	0.83260	5.06350	-1.51670
C	0.39470	5.57410	-4.03360
C	0.31100	5.59180	-5.54690
H	0.61400	6.58680	-3.68020
O	-0.81830	5.10530	-3.45260
O	-0.71590	6.54890	-5.88070
H	1.25690	5.90630	-5.98990
H	0.03370	4.61460	-5.93860

H	-1.52110	5.72350	-3.68750
H	2.47500	5.11290	-3.93140
C	-1.00140	6.70650	-7.18230
C	-2.08170	7.69950	-7.41500
C	-2.71620	8.36460	-6.36270
C	-3.72180	9.28420	-6.62970
C	-4.09870	9.54510	-7.94400
C	-3.46830	8.88410	-8.99500
C	-2.46340	7.96440	-8.73210
H	-2.42250	8.16280	-5.34280
H	-4.21150	9.79780	-5.81240
H	-4.88290	10.26290	-8.14910
H	-3.76050	9.08640	-10.01730
H	-1.96600	7.44440	-9.53930
C	4.39270	6.66090	-0.82530
C	4.66700	8.00400	-0.55700
C	5.91050	8.37590	-0.06770
C	6.88810	7.40940	0.15430
C	6.61970	6.07020	-0.11430
C	5.37580	5.69310	-0.60230
H	3.89940	8.74470	-0.73470
H	6.11900	9.41740	0.14050
H	7.85890	7.70000	0.53570
H	7.38010	5.31930	0.05680
H	5.16690	4.65450	-0.81350
C	-1.64330	2.43780	-0.06300
C	-2.43040	2.01170	-1.13610
C	-2.19490	2.51010	1.21820
C	-3.51970	2.15510	1.42590
C	-4.30210	1.73060	0.35480
C	-3.75700	1.66140	-0.92420
H	-2.00690	1.96410	-2.12870
H	-1.57700	2.84390	2.04050
H	-3.94350	2.20960	2.42030
H	-5.33660	1.45540	0.51690

H	-4.36630	1.33480	-1.75700
O	-0.42730	6.09840	-8.06060
O	2.17460	7.11050	-1.59850
O	0.47760	3.29900	0.63070

Methyl β-D-Gal*f* **2**,C1-endo, -60°/-60°

	**X**	**Y**	**Z**
C	-1.91750	2.89110	-2.64760
O	-1.48910	3.73700	-3.68940
C	-1.34830	3.51870	-1.38180
C	-0.02950	4.13460	-1.85830
C	-0.16070	4.19580	-3.39920
O	-1.38700	1.59760	-2.74540
H	-3.00980	2.87250	-2.68150
O	1.02880	3.25430	-1.45070
C	-1.81480	0.89930	-3.91480
H	-2.90790	0.84610	-3.95140
H	-1.40210	-0.10490	-3.84790
H	-1.44900	1.38730	-4.82030
H	-1.20210	2.80720	-0.57470
O	-2.27260	4.54130	-0.99110
C	-2.09790	5.09220	0.22630
C	2.27780	3.75420	-1.44350
H	0.13220	5.11670	-1.42530
C	0.07760	5.58520	-3.97520
C	0.16710	5.63040	-5.49020
H	1.06770	5.88350	-3.61390
O	-0.92060	6.46040	-3.45960
O	-1.06390	5.15690	-6.05700
H	0.34690	6.65470	-5.82150
H	0.99160	5.00460	-5.83700
H	-0.61770	7.36930	-3.57270
H	0.57170	3.51050	-3.83570
C	-1.12910	5.03660	-7.38680

C	-2.41030	4.42520	-7.82710
C	-3.31260	3.88190	-6.90830
C	-4.49100	3.30110	-7.35810
C	-4.77670	3.26420	-8.72020
C	-3.87930	3.80590	-9.63690
C	-2.69740	4.38180	-9.19260
H	-3.08150	3.90890	-5.85280
H	-5.18670	2.87570	-6.64620
H	-5.69730	2.81220	-9.06760
H	-4.10100	3.77740	-10.69600
H	-1.99000	4.80240	-9.89430
C	3.28410	2.75590	-1.00690
C	4.61690	3.16160	-0.90690
C	5.59080	2.25870	-0.50630
C	5.23980	0.94500	-0.20590
C	3.91290	0.53620	-0.30640
C	2.93460	1.43710	-0.70480
H	4.87600	4.18440	-1.14400
H	6.62230	2.57690	-0.42820
H	6.00030	0.24010	0.10580
H	3.64060	-0.48530	-0.07430
H	1.90480	1.12100	-0.78620
C	-3.07920	6.16900	0.50330
C	-3.99610	6.59840	-0.45990
C	-3.06640	6.76670	1.76570
C	-3.96450	7.78090	2.06410
C	-4.87760	8.20650	1.10270
C	-4.89090	7.61590	-0.15770
H	-4.00140	6.14240	-1.43910
H	-2.35110	6.42810	2.50250
H	-3.95340	8.24040	3.04400
H	-5.57770	8.99900	1.33550
H	-5.59860	7.94910	-0.90590
O	-0.23640	5.38230	-8.13400
O	2.53280	4.89560	-1.76400

O	-1.22890	4.73290	0.99050

Methyl β-D-Gal*f* **2**,C1-endo, -60°/180°

	**X**	**Y**	**Z**
C	3.85230	5.14460	-3.20840
O	2.77320	5.23830	-4.11210
C	3.19900	5.08530	-1.83300
C	1.95240	5.96130	-1.99780
C	1.74730	6.05680	-3.52530
O	4.67450	6.27840	-3.22440
H	4.41350	4.24500	-3.47330
O	2.25510	7.24160	-1.42060
C	5.34190	6.48130	-4.46960
H	5.93140	5.59850	-4.73780
H	6.00320	7.33420	-4.33380
H	4.62870	6.69450	-5.26810
H	3.84530	5.43740	-1.03470
O	2.83280	3.71760	-1.61890
C	2.45620	3.37170	-0.37180
C	1.22600	8.08770	-1.23880
H	1.09100	5.53300	-1.49530
C	0.38600	5.58850	-4.00890
C	0.27300	5.61360	-5.52720
H	-0.35150	6.27080	-3.57910
O	0.16620	4.25780	-3.55020
O	0.47260	6.97740	-5.94270
H	1.02640	4.97400	-5.98420
H	-0.71720	5.27910	-5.83880
H	-0.78270	4.08450	-3.55110
H	1.88720	7.09460	-3.83440
C	0.48500	7.22370	-7.26050
C	0.75130	8.65300	-7.56680
C	1.00400	9.58910	-6.56040
C	1.25490	10.91350	-6.89320

C	1.25370	11.31070	-8.22740
C	1.00170	10.38030	-9.23240
C	0.75210	9.05560	-8.90390
H	1.00490	9.27960	-5.52520
H	1.45160	11.63620	-6.11180
H	1.44910	12.34430	-8.48400
H	1.00030	10.68830	-10.27000
H	0.55640	8.32290	-9.67480
C	1.65360	9.38440	-0.65920
C	0.67830	10.35530	-0.42030
C	1.03600	11.58360	0.11620
C	2.36950	11.84950	0.41660
C	3.34460	10.88510	0.17870
C	2.99120	9.65440	-0.35790
H	-0.35330	10.13660	-0.65930
H	0.27760	12.33350	0.30010
H	2.64850	12.80830	0.83520
H	4.38120	11.09230	0.41120
H	3.74710	8.90590	-0.54590
C	2.04980	1.94840	-0.28110
C	1.95400	1.13420	-1.41270
C	1.74650	1.42610	0.97840
C	1.35610	0.10120	1.10640
C	1.26130	-0.70810	-0.02300
C	1.55860	-0.19010	-1.28030
H	2.17950	1.53970	-2.38820
H	1.82200	2.06570	1.84710
H	1.12490	-0.30110	2.08420
H	0.95470	-1.74170	0.07690
H	1.48160	-0.81840	-2.15820
O	0.30000	6.36370	-8.09550
O	0.08280	7.80450	-1.52870
O	2.45660	4.15240	0.55480

Methyl β-D-Gal*f* **2**,C1-endo, 180°/+60°

	**X**	**Y**	**Z**
C	3.50560	4.24140	-4.71270
O	2.64210	5.36830	-4.68290
C	2.59230	3.00880	-4.76110
C	1.18440	3.57670	-4.55160
C	1.44590	4.98090	-4.00380
O	4.29120	4.14370	-3.55910
H	4.13160	4.34720	-5.60210
O	0.40600	2.84480	-3.60250
C	5.24900	5.19550	-3.43120
H	5.89930	5.22980	-4.31140
H	5.84250	4.97170	-2.54750
H	4.75690	6.16200	-3.30710
H	2.85180	2.30820	-3.97270
O	2.70880	2.37130	-6.03510
C	2.79210	1.02300	-6.06670
C	-0.29990	1.78740	-4.05880
H	0.66110	3.61550	-5.50660
C	0.36430	6.00560	-4.28400
C	-0.98750	5.54800	-3.76110
H	0.29750	6.15010	-5.36910
O	0.76550	7.21680	-3.65180
O	-1.89620	6.63470	-4.02380
H	-1.34310	4.65610	-4.27370
H	-0.95480	5.35660	-2.68830
H	0.06830	7.86990	-3.79420
H	1.61910	4.92680	-2.92330
C	-3.16390	6.47790	-3.60640
C	-4.01260	7.66030	-3.90020
C	-3.50430	8.79920	-4.53050
C	-4.33660	9.88170	-4.78210
C	-5.67640	9.83420	-4.40780
C	-6.18600	8.70070	-3.77980
C	-5.35760	7.61720	-3.52620

H	-2.46460	8.83570	-4.82170
H	-3.94020	10.76260	-5.27020
H	-6.32310	10.67990	-4.60530
H	-7.22770	8.66310	-3.48850
H	-5.74080	6.73130	-3.03890
C	-1.14930	1.18330	-3.00620
C	-1.82720	-0.00120	-3.30380
C	-2.64220	-0.59370	-2.35070
C	-2.78890	-0.00420	-1.09750
C	-2.11750	1.17790	-0.79830
C	-1.29680	1.77190	-1.74740
H	-1.70570	-0.44710	-4.28140
H	-3.16370	-1.51320	-2.58290
H	-3.42720	-0.46570	-0.35460
H	-2.23480	1.63760	0.17440
H	-0.77750	2.69090	-1.51820
C	2.78950	0.49720	-7.45390
C	2.64580	1.33310	-8.56430
C	2.92400	-0.88110	-7.63630
C	2.91700	-1.41830	-8.91520
C	2.77390	-0.58260	-10.01990
C	2.63820	0.79130	-9.84260
H	2.53740	2.39870	-8.42530
H	3.03220	-1.51930	-6.77020
H	3.02180	-2.48680	-9.05210
H	2.76710	-1.00210	-11.01800
H	2.52510	1.44070	-10.70110
O	-3.55230	5.47160	-3.05310
O	-0.23410	1.40100	-5.20410
O	2.86530	0.33620	-5.07320

Methyl β-D-Gal*f* **2**,C1-endo, 180°/-60°

	**X**	**Y**	**Z**
C	3.77070	6.05140	-2.39100

O	2.89790	6.16960	-3.49780
C	3.24640	4.85620	-1.59760
C	1.76500	4.76280	-2.00050
C	1.57430	5.90200	-3.02030
O	3.71660	7.16360	-1.54130
H	4.77660	5.90890	-2.79270
O	0.91170	4.97330	-0.87420
C	4.17200	8.37080	-2.15280
H	5.19420	8.24930	-2.52580
H	4.15360	9.13730	-1.38120
H	3.51970	8.66460	-2.97740
H	3.36170	4.98940	-0.52620
O	3.98260	3.70490	-2.03350
C	3.89890	2.60370	-1.25820
C	0.00850	4.02030	-0.55660
H	1.56040	3.78790	-2.43530
C	0.68230	5.58040	-4.20730
C	-0.69050	5.06970	-3.80340
H	1.16170	4.78280	-4.78810
O	0.59350	6.77480	-4.97980
O	-1.26710	6.00870	-2.87700
H	-1.33000	4.99040	-4.68410
H	-0.62790	4.08770	-3.33580
H	0.14420	6.57110	-5.80980
H	1.18040	6.77790	-2.49980
C	-2.42990	5.67100	-2.29840
C	-2.88750	6.68660	-1.31780
C	-2.20200	7.88920	-1.12810
C	-2.65600	8.80380	-0.18870
C	-3.78950	8.52210	0.56850
C	-4.47170	7.32340	0.38360
C	-4.02430	6.40900	-0.55820
H	-1.31780	8.10140	-1.71130
H	-2.12300	9.73450	-0.04270
H	-4.13760	9.23420	1.30610

H	-5.34710	7.09910	0.97910
H	-4.53980	5.47030	-0.70480
C	-0.86440	4.43180	0.56690
C	-1.88050	3.55960	0.96340
C	-2.74320	3.91810	1.98800
C	-2.59630	5.14920	2.62090
C	-1.58550	6.02060	2.22890
C	-0.72070	5.66680	1.20380
H	-1.98830	2.61110	0.45600
H	-3.53370	3.24290	2.28870
H	-3.27630	5.43340	3.41400
H	-1.48020	6.98250	2.71250
H	0.05500	6.34820	0.88800
C	4.68970	1.47030	-1.79590
C	5.41180	1.56990	-2.98810
C	4.69830	0.27260	-1.07700
C	5.42270	-0.81410	-1.54420
C	6.14170	-0.71180	-2.73240
C	6.13470	0.47940	-3.45250
H	5.40430	2.49440	-3.54690
H	4.13480	0.20600	-0.15650
H	5.42740	-1.74030	-0.98430
H	6.70660	-1.56040	-3.09710
H	6.69290	0.55840	-4.37640
O	-3.01750	4.64030	-2.55060
O	-0.08580	2.96920	-1.15060
O	3.24320	2.56880	-0.24050

Methyl β-D-Gal*f* **2**,C1-endo, 180°/180°

	**X**	**Y**	**Z**
C	2.34470	4.62170	-7.23900
O	2.08120	5.52880	-6.18310
C	0.97450	4.28530	-7.83420
C	-0.01910	4.69360	-6.73600

C	0.86840	5.10500	-5.55290
O	2.91150	3.42440	-6.78570
H	3.01120	5.13350	-7.93740
O	-0.89300	3.64250	-6.31540
C	4.21290	3.58710	-6.22130
H	4.88600	4.06230	-6.94230
H	4.57740	2.59000	-5.98400
H	4.17490	4.18960	-5.31170
H	0.90240	3.23350	-8.08990
O	0.73870	5.08960	-8.99400
C	0.60270	4.47100	-10.18390
C	-1.88900	3.31840	-7.16690
H	-0.61630	5.52970	-7.09450
C	0.34560	6.23640	-4.68500
C	-0.95030	5.88720	-3.95940
H	0.19410	7.12230	-5.31150
O	1.34800	6.47780	-3.70060
O	-2.03970	5.91540	-4.89680
H	-0.88600	4.90120	-3.49970
H	-1.13440	6.62480	-3.17680
H	1.13640	7.29900	-3.23930
H	1.05680	4.23100	-4.92000
C	-3.21000	5.41080	-4.46940
C	-4.23940	5.36950	-5.53590
C	-4.01720	5.91760	-6.80070
C	-4.99450	5.81530	-7.78080
C	-6.19340	5.16540	-7.50510
C	-6.41880	4.62080	-6.24340
C	-5.44710	4.72470	-5.26090
H	-3.08010	6.40810	-7.01890
H	-4.81680	6.23450	-8.76260
H	-6.95020	5.07840	-8.27450
H	-7.34730	4.10700	-6.03150
H	-5.60230	4.29270	-4.28230
C	-2.89440	2.42080	-6.55500

C	-3.98070	2.02010	-7.33500
C	-4.97540	1.22690	-6.78480
C	-4.89190	0.83080	-5.45280
C	-3.80900	1.22620	-4.67260
C	-2.81030	2.01880	-5.21990
H	-4.04100	2.34900	-8.36290
H	-5.82090	0.92530	-7.38920
H	-5.67280	0.21730	-5.02140
H	-3.74710	0.92280	-3.63560
H	-1.97680	2.33920	-4.61220
C	0.20620	5.41480	-11.25880
C	-0.08880	6.75510	-10.99600
C	0.11730	4.92870	-12.56510
C	-0.26000	5.77370	-13.59860
C	-0.55360	7.10890	-13.33350
C	-0.46830	7.59710	-12.03280
H	-0.02390	7.13200	-9.98580
H	0.34530	3.88930	-12.75720
H	-0.32640	5.39320	-14.60970
H	-0.84940	7.76800	-14.14000
H	-0.69860	8.63430	-11.82610
O	-3.37970	5.01740	-3.33490
O	-1.94140	3.74830	-8.29800
O	0.78850	3.28590	-10.34660

Methyl β-D-Gal*f* **2**,C2-exo, +60°/+60°

	**X**	**Y**	**Z**
O	-2.24720	1.76900	1.43300
O	-3.79890	6.65810	-0.10930
O	0.05550	3.96680	-0.12480
O	-3.31950	3.21870	-1.16360
O	-2.10490	4.07030	1.84670
O	-1.75150	8.14820	1.17240
C	-1.14840	3.19170	-0.08550

C	-2.34230	4.04990	-0.52710
C	-2.92170	4.59820	0.79210
C	-2.96520	6.12350	0.91390
C	-1.44020	2.91050	1.38300
C	-1.60230	6.76530	0.79660
H	-1.04550	2.29090	-0.68220
H	-2.04110	4.82920	-1.21870
H	-3.36860	6.36500	1.90300
H	-0.54530	2.79070	1.99880
H	-3.94560	4.22720	0.89270
H	-1.22070	6.71850	-0.22390
H	-0.89440	6.27860	1.46290
C	0.68700	4.04890	-1.31720
C	-3.63300	3.46400	-2.45390
C	-0.62490	8.85190	1.35100
C	-2.59320	1.37130	2.75980
H	-1.69100	1.19820	3.35540
H	-3.20930	2.12820	3.24920
H	-3.15550	0.44450	2.67120
C	-0.88850	10.26690	1.72160
C	-2.18560	10.77470	1.82920
C	-2.38260	12.10470	2.17620
C	-1.29020	12.93300	2.41750
C	0.00390	12.42990	2.31060
C	0.20460	11.10180	1.96330
H	-3.03230	10.13120	1.63850
H	-3.38860	12.49580	2.25800
H	-1.44690	13.96970	2.68780
H	0.85400	13.07330	2.49740
H	1.20440	10.69910	1.87580
C	1.88910	4.91310	-1.26260
C	2.21340	5.66000	-0.12770
C	2.70360	4.98080	-2.39620
C	3.83540	5.78180	-2.39180
C	4.15650	6.52560	-1.25850

C	3.34410	6.46540	-0.13040
H	-4.71560	6.41830	0.07680
H	1.57760	5.62330	0.74370
H	2.44010	4.40140	-3.27040
H	4.46620	5.82960	-3.27000
H	5.03820	7.15400	-1.25690
H	3.58720	7.05100	0.74650
C	-4.63150	2.49620	-2.97340
C	-5.11570	1.44170	-2.19480
C	-6.04610	0.55900	-2.72690
C	-6.49780	0.72350	-4.03330
C	-6.01670	1.77330	-4.81150
C	-5.08600	2.65670	-4.28440
H	-4.76210	1.31380	-1.18220
H	-6.41860	-0.25810	-2.12270
H	-7.22350	0.03350	-4.44510
H	-6.36700	1.90140	-5.82750
H	-4.70320	3.47510	-4.87850
O	0.29510	3.46910	-2.30530
O	-3.15510	4.37030	-3.09780
O	0.48260	8.37330	1.22210

Methyl β-D-Gal*f* **2**,C2-exo, +60°/-60°

	**X**	**Y**	**Z**
O	-3.34670	3.26710	-2.40000
O	-4.14360	7.19480	1.08050
O	-1.24560	6.09770	-2.69020
O	-4.82150	6.14330	-2.29110
O	-2.42630	4.43060	-0.58750
O	-1.77400	8.02300	-0.05910
C	-2.52540	5.45380	-2.67280
C	-3.49500	6.21000	-1.75270
C	-3.43780	5.43320	-0.42050
C	-3.12260	6.23200	0.84640

C	-2.32710	4.12020	-1.96400
C	-1.78170	6.93460	0.88310
H	-2.91420	5.34840	-3.68100
H	-3.20870	7.24610	-1.64530
H	-3.08340	5.50500	1.66670
H	-1.34170	3.67420	-2.11860
H	-4.41140	4.95490	-0.27370
H	-0.98460	6.23390	0.63710
H	-1.60770	7.33320	1.88190
C	-1.09960	7.14380	-3.53100
C	-5.46710	7.30090	-2.54280
C	-0.69210	8.81660	-0.06160
C	-3.29120	1.96450	-1.81850
H	-2.32220	1.49630	-2.01920
H	-3.45460	2.00780	-0.73990
H	-4.08210	1.38080	-2.28430
C	-0.80400	9.92640	-1.04060
C	-1.96330	10.13620	-1.79170
C	-2.01310	11.17590	-2.70950
C	-0.91370	12.01220	-2.87920
C	0.23970	11.80850	-2.12720
C	0.29540	10.76920	-1.21140
H	-2.82020	9.49150	-1.66620
H	-2.91020	11.33070	-3.29500
H	-0.95420	12.81960	-3.59950
H	1.09840	12.45270	-2.26460
H	1.19050	10.59090	-0.63240
C	0.24860	7.75060	-3.44510
C	1.20310	7.30210	-2.52900
C	0.54910	8.81420	-4.29790
C	1.79350	9.42120	-4.23820
C	2.74270	8.97400	-3.32350
C	2.44570	7.91650	-2.46920
H	-4.97170	6.73320	1.26340
H	0.96720	6.48760	-1.86020

H	-0.20270	9.16160	-4.99240
H	2.02060	10.25100	-4.89450
H	3.71150	9.45470	-3.27160
H	3.18030	7.57460	-1.75180
C	-6.81900	7.07120	-3.11070
C	-7.30090	5.78800	-3.38260
C	-8.57220	5.62470	-3.91610
C	-9.36770	6.73630	-4.17890
C	-8.89020	8.01620	-3.90900
C	-7.61980	8.18410	-3.37770
H	-6.68160	4.92640	-3.17960
H	-8.94290	4.62990	-4.12720
H	-10.35900	6.60560	-4.59440
H	-9.50810	8.88100	-4.11350
H	-7.23630	9.17240	-3.16440
O	-1.98710	7.52420	-4.26210
O	-4.99350	8.39370	-2.32400
O	0.26370	8.62900	0.66180

Methyl β-D-Gal*f* **2**,C2-exo, +60°/180°

	**X**	**Y**	**Z**
O	-3.96380	3.78740	4.53230
O	-3.36610	5.73280	-0.39310
O	-0.66960	4.52410	3.47940
O	-3.18860	2.73330	1.61820
O	-3.37520	5.66860	3.26620
O	-1.45570	7.46950	2.09940
C	-1.92720	3.84770	3.35490
C	-2.46130	3.93060	1.91780
C	-3.42360	5.13380	1.93690
C	-3.13290	6.23990	0.91770
C	-2.93720	4.66200	4.15390
C	-1.70940	6.77330	0.87340
H	-1.84010	2.82050	3.69660

H	-1.65800	4.03820	1.19840
H	-3.80480	7.07610	1.13980
H	-2.51040	5.16890	5.02310
H	-4.43240	4.76160	1.73020
H	-1.61790	7.46080	0.03260
H	-0.97420	5.97880	0.74450
C	0.42090	3.86130	3.03850
C	-2.83660	2.02210	0.52570
C	-0.24140	8.01230	2.25270
C	-4.99160	4.41350	5.29950
H	-4.56920	4.88730	6.19160
H	-5.52180	5.16380	4.70960
H	-5.68270	3.62770	5.59620
C	-0.06990	8.62200	3.59530
C	-0.99340	8.40070	4.61980
C	-0.77400	8.94580	5.87760
C	0.35860	9.72000	6.11510
C	1.27600	9.94760	5.09260
C	1.06550	9.39590	3.83730
H	-1.86530	7.79080	4.43090
H	-1.48470	8.76540	6.67380
H	0.52710	10.14450	7.09680
H	2.15710	10.54850	5.27730
H	1.77830	9.55010	3.03900
C	1.67260	4.62010	3.26640
C	1.72040	5.72230	4.12190
C	2.83040	4.19910	2.60750
C	4.02240	4.88270	2.79490
C	4.06730	5.98150	3.65010
C	2.91790	6.39700	4.31470
H	-4.29940	5.50130	-0.47940
H	0.82710	6.04690	4.63340
H	2.78140	3.34350	1.94800
H	4.91600	4.56140	2.27570
H	4.99880	6.51350	3.79720

H	2.95040	7.25130	4.97720
C	-3.66700	0.80150	0.36930
C	-4.66920	0.46450	1.28290
C	-5.41720	-0.69010	1.09520
C	-5.17070	-1.51190	-0.00100
C	-4.17250	-1.17870	-0.91290
C	-3.42260	-0.02610	-0.72910
H	-4.85790	1.10210	2.13420
H	-6.19240	-0.94960	1.80460
H	-5.75560	-2.41170	-0.14430
H	-3.97970	-1.81740	-1.76510
H	-2.64400	0.24310	-1.42940
O	0.35650	2.77080	2.51560
O	-1.95120	2.35120	-0.23050
O	0.61060	7.99260	1.38920

Methyl β-D-Gal*f* **2**,C2-exo, -60°/+60°

	**X**	**Y**	**Z**
O	-1.76460	1.79410	0.44040
O	-2.62000	6.69540	-0.27770
O	-1.99530	4.02690	-2.29600
O	-4.66580	2.96690	-0.16760
O	-1.55700	4.12280	0.58440
O	-2.54940	7.82610	2.33780
C	-2.55730	3.18020	-1.28670
C	-3.65490	3.92300	-0.51740
C	-2.93940	4.48790	0.72870
C	-3.04420	6.00120	0.89400
C	-1.46870	2.98080	-0.24040
C	-2.18850	6.46070	2.05430
H	-2.91440	2.25110	-1.72040
H	-4.10230	4.70230	-1.12610
H	-4.09020	6.22840	1.11120
H	-0.45580	2.96140	-0.65030

H	-3.35530	4.00700	1.61900
H	-1.13120	6.41140	1.79900
H	-2.36990	5.84980	2.93930
C	-2.69950	4.16940	-3.43700
C	-5.85150	3.46030	0.23550
C	-1.86140	8.44320	3.30740
C	-0.83000	1.48160	1.47330
H	0.18520	1.42430	1.06750
H	-0.85870	2.22910	2.26860
H	-1.11890	0.51200	1.87290
C	-2.29710	9.84980	3.51150
C	-3.29350	10.43690	2.72750
C	-3.66680	11.75520	2.95360
C	-3.05050	12.49290	3.96030
C	-2.05660	11.91090	4.74290
C	-1.68050	10.59430	4.51930
H	-3.76990	9.86370	1.94550
H	-4.43860	12.20760	2.34430
H	-3.34400	13.52050	4.13470
H	-1.57630	12.48370	5.52570
H	-0.90960	10.13090	5.11960
C	-2.06190	5.12150	-4.37740
C	-0.93340	5.86560	-4.02300
C	-2.62780	5.27760	-5.64490
C	-2.06790	6.16550	-6.55170
C	-0.94340	6.90600	-6.19600
C	-0.37930	6.75640	-4.93230
H	-3.39130	6.92750	-0.80640
H	-0.50060	5.75220	-3.03970
H	-3.50280	4.69860	-5.90690
H	-2.50690	6.28210	-7.53400
H	-0.50790	7.60060	-6.90320
H	0.49240	7.33490	-4.65500
C	-6.82860	2.39380	0.56150
C	-6.49320	1.03880	0.49570

C	-7.44120	0.07560	0.81340
C	-8.72420	0.45740	1.19530
C	-9.06080	1.80700	1.26200
C	-8.11620	2.77300	0.94770
H	-5.49630	0.74430	0.20160
H	-7.17950	-0.97340	0.76300
H	-9.46150	-0.29640	1.44130
H	-10.05830	2.10420	1.55900
H	-8.36420	3.82440	0.99630
O	-3.73660	3.57850	-3.64550
O	-6.07340	4.64980	0.31610
O	-0.98160	7.90030	3.94360

Methyl β-D-Gal*f* **2**,C2-exo, -60°/-60°

	**X**	**Y**	**Z**
O	-0.17140	5.41300	-2.56110
O	-3.77030	7.67690	0.09360
O	-3.20750	7.14040	-3.17570
O	-2.78310	3.82770	-1.87900
O	-1.32970	6.73740	-1.01600
O	-1.35480	7.64000	1.62170
C	-2.43900	5.96260	-2.90110
C	-3.03670	5.22810	-1.69930
C	-2.27440	5.80080	-0.47910
C	-3.17540	6.47260	0.56480
C	-1.09560	6.44880	-2.37470
C	-2.47110	6.76990	1.87130
H	-2.36300	5.33380	-3.78250
H	-4.11060	5.38160	-1.63240
H	-3.94720	5.73580	0.81150
H	-0.74690	7.37450	-2.84040
H	-1.74390	4.97700	0.00710
H	-2.11220	5.84350	2.32120
H	-3.16090	7.25480	2.56220

C	-4.34620	6.98350	-3.88330
C	-3.46900	2.98070	-1.08880
C	-0.54960	7.92770	2.64890
C	1.13460	5.72880	-2.07890
H	1.50860	6.64210	-2.55340
H	1.13200	5.86290	-0.99540
H	1.77630	4.89110	-2.34250
C	0.62380	8.73540	2.22400
C	0.89950	8.96450	0.87300
C	2.01590	9.70740	0.51240
C	2.85590	10.22790	1.49310
C	2.58200	10.00110	2.83950
C	1.47110	9.25380	3.20470
H	0.24590	8.55250	0.11730
H	2.23200	9.87970	-0.53420
H	3.72440	10.80860	1.20860
H	3.23490	10.40570	3.60220
H	1.24890	9.06690	4.24660
C	-5.10530	8.25040	-4.00840
C	-4.73380	9.40340	-3.31150
C	-6.23230	8.26880	-4.83350
C	-6.97770	9.43100	-4.96560
C	-6.60580	10.57870	-4.26970
C	-5.48640	10.56260	-3.44260
H	-4.44660	7.46910	-0.56200
H	-3.86830	9.38680	-2.66520
H	-6.51200	7.36960	-5.36520
H	-7.84840	9.44370	-5.60830
H	-7.18970	11.48480	-4.37090
H	-5.20040	11.45350	-2.89850
C	-3.13860	1.56080	-1.35770
C	-2.17400	1.19220	-2.29930
C	-1.89560	-0.15080	-2.51450
C	-2.57650	-1.12920	-1.79560
C	-3.53800	-0.76450	-0.85660

C	-3.81800	0.57610	-0.63660
H	-1.64490	1.95310	-2.85440
H	-1.14730	-0.43460	-3.24320
H	-2.35760	-2.17570	-1.96640
H	-4.06720	-1.52510	-0.29740
H	-4.56160	0.87280	0.09020
O	-4.68990	5.91840	-4.34570
O	-4.26340	3.36090	-0.25520
O	-0.75890	7.55930	3.78680

Methyl β-D-Gal*f* **2**,C2-exo, -60°/180°

	**X**	**Y**	**Z**
O	-6.13370	3.52600	1.30090
O	-2.38250	6.25840	-0.57650
O	-4.27030	3.80010	-1.70050
O	-6.73740	6.06120	-0.41750
O	-4.08560	4.64220	1.08190
O	-3.32720	7.54560	2.64340
C	-5.33750	4.16620	-0.81890
C	-5.36830	5.68560	-0.63330
C	-4.50400	5.93700	0.61970
C	-3.27410	6.81140	0.38610
C	-4.94990	3.65580	0.56340
C	-2.45240	6.97730	1.65440
H	-6.28430	3.77190	-1.17490
H	-4.99750	6.20430	-1.51300
H	-3.63460	7.79110	0.06280
H	-4.39020	2.71690	0.54830
H	-5.12590	6.41230	1.38090
H	-1.61380	7.64940	1.47510
H	-2.07430	6.01700	2.00230
C	-4.49340	3.93740	-3.02360
C	-7.01420	7.37750	-0.44450
C	-2.86330	7.64660	3.89680

C	-5.92500	3.07210	2.63790
H	-5.38700	2.11850	2.64000
H	-5.36170	3.80520	3.21850
H	-6.91010	2.93640	3.07880
C	-3.88180	8.19050	4.83210
C	-5.17960	8.49620	4.41310
C	-6.10210	8.99470	5.32320
C	-5.73610	9.19200	6.65170
C	-4.44360	8.88880	7.07190
C	-3.51930	8.38890	6.16580
H	-5.46270	8.34200	3.38190
H	-7.10700	9.22910	4.99620
H	-6.45740	9.58120	7.35910
H	-4.15830	9.04190	8.10470
H	-2.51280	8.14790	6.47960
C	-3.29200	3.61960	-3.83180
C	-2.04920	3.37560	-3.24120
C	-3.42020	3.58490	-5.22230
C	-2.31760	3.30240	-6.01510
C	-1.08010	3.05950	-5.42420
C	-0.94730	3.09880	-4.03910
H	-2.65900	6.52370	-1.46100
H	-1.94720	3.41320	-2.16640
H	-4.38600	3.77900	-5.66830
H	-2.42060	3.27260	-7.09200
H	-0.21910	2.84150	-6.04340
H	0.01560	2.91440	-3.58060
C	-8.44320	7.66270	-0.17110
C	-9.35260	6.64810	0.13960
C	-10.67960	6.96410	0.39780
C	-11.10500	8.28860	0.34630
C	-10.20080	9.30150	0.03720
C	-8.87350	8.99070	-0.21960
H	-9.01990	5.62120	0.18270
H	-11.38230	6.17730	0.63970

H	-12.14070	8.53160	0.54760
H	-10.53120	10.33140	-0.00260
H	-8.16070	9.76770	-0.45920
O	-5.56110	4.28760	-3.47640
O	-6.17000	8.22020	-0.66460
O	-1.73860	7.32300	4.21460

Methyl β-D-Gal*f* **2**,C2-exo, 180°/+60°

	**X**	**Y**	**Z**
O	-2.77470	1.35780	1.47280
O	-4.36460	6.13910	1.06700
O	-0.94610	4.19630	2.53590
O	-1.35350	2.78800	-0.76620
O	-3.56760	3.55600	1.34240
O	-2.09250	7.94450	0.33170
C	-1.26580	3.17420	1.58270
C	-1.49280	3.82220	0.21530
C	-2.93370	4.33590	0.30490
C	-3.02270	5.80560	0.72830
C	-2.65040	2.65950	1.95790
C	-2.55660	6.75990	-0.34920
H	-0.50620	2.39880	1.56600
H	-0.77140	4.60780	0.01330
H	-2.38030	5.94210	1.60220
H	-2.84270	2.69100	3.03420
H	-3.47240	4.17290	-0.62970
H	-3.37710	7.01460	-1.02000
H	-1.73970	6.34690	-0.93750
C	0.30490	4.70980	2.50430
C	-1.18880	3.18040	-2.04500
C	-1.54170	8.90950	-0.41530
C	-4.01650	0.73850	1.80750
H	-4.17160	0.75180	2.89130
H	-4.85130	1.24230	1.31630

H	-3.95600	-0.29030	1.45980
C	-0.96020	9.99780	0.41370
C	-0.78410	9.85610	1.79270
C	-0.21380	10.88680	2.52720
C	0.17660	12.06330	1.89410
C	0.00250	12.20720	0.52000
C	-0.55970	11.17620	-0.21930
H	-1.07950	8.94020	2.28340
H	-0.07100	10.76990	3.59320
H	0.61870	12.86640	2.47020
H	0.30640	13.12130	0.02660
H	-0.69570	11.27390	-1.28770
C	0.44130	5.90380	3.37150
C	-0.63320	6.41070	4.10770
C	1.67870	6.55090	3.41380
C	1.84040	7.69200	4.18530
C	0.76910	8.19240	4.92020
C	-0.46580	7.55200	4.87990
H	-4.65540	5.50170	1.73380
H	-1.59350	5.91760	4.07090
H	2.50160	6.15460	2.83510
H	2.79900	8.19340	4.21230
H	0.89520	9.08480	5.51980
H	-1.30010	7.94520	5.44590
C	-1.06660	2.03300	-2.97500
C	-1.17860	0.71130	-2.53470
C	-1.05970	-0.33180	-3.44290
C	-0.82820	-0.06310	-4.78910
C	-0.71740	1.25310	-5.23020
C	-0.83720	2.29890	-4.32690
H	-1.36070	0.50580	-1.48980
H	-1.14770	-1.35500	-3.10110
H	-0.73510	-0.87910	-5.49460
H	-0.53800	1.46210	-6.27690
H	-0.75370	3.32550	-4.65610

O	1.18500	4.24280	1.81750
O	-1.14880	4.34670	-2.37230
O	-1.51980	8.88080	-1.62830

Methyl β-D-Gal*f* **2**,C2-exo, 180°/-60°

	**X**	**Y**	**Z**
O	-5.81670	3.26370	1.60720
O	-3.10870	7.16480	2.65380
O	-2.51790	2.70740	0.45010
O	-4.96190	4.90300	-1.00350
O	-3.94660	4.53690	2.21570
O	-3.99570	8.04490	-0.01750
C	-3.85360	3.20900	0.31450
C	-3.83100	4.66980	-0.15720
C	-3.96680	5.49430	1.14030
C	-2.85420	6.49690	1.42020
C	-4.42340	3.30030	1.72420
C	-2.69360	7.54380	0.34050
H	-4.43590	2.57390	-0.34610
H	-2.92010	4.88780	-0.70580
H	-1.90460	5.94970	1.47770
H	-4.05980	2.52630	2.40430
H	-4.92760	6.01020	1.12940
H	-2.22930	7.11830	-0.54740
H	-2.07160	8.35880	0.70920
C	-1.90320	2.30410	-0.68230
C	-4.78910	5.63340	-2.12490
C	-4.04510	8.91770	-1.03580
C	-6.49880	3.36440	2.85740
H	-6.17090	2.57230	3.53810
H	-6.32390	4.33620	3.32310
H	-7.55890	3.24730	2.64420
C	-5.43540	9.21570	-1.46070
C	-6.53400	8.56370	-0.89660

C	-7.81340	8.83360	-1.36060
C	-8.00390	9.75550	-2.38450
C	-6.91180	10.41200	-2.94550
C	-5.63120	10.14140	-2.48730
H	-6.38340	7.83630	-0.11240
H	-8.66090	8.31440	-0.93290
H	-9.00250	9.95710	-2.75110
H	-7.05940	11.12670	-3.74480
H	-4.77260	10.63440	-2.92210
C	-0.52360	1.82110	-0.43600
C	0.04190	1.81460	0.84200
C	0.22040	1.36680	-1.52750
C	1.51680	0.90930	-1.34310
C	2.07780	0.90360	-0.06850
C	1.33990	1.35630	1.02160
H	-3.23530	6.49100	3.33470
H	-0.53160	2.16790	1.68660
H	-0.22690	1.37800	-2.51190
H	2.09070	0.55810	-2.19080
H	3.09000	0.54690	0.07490
H	1.77660	1.35250	2.01190
C	-6.06230	5.84200	-2.85370
C	-7.25180	5.22650	-2.45700
C	-8.42490	5.47610	-3.15510
C	-8.41840	6.34390	-4.24310
C	-7.23450	6.96040	-4.63790
C	-6.05880	6.70770	-3.94870
H	-7.25630	4.56270	-1.60480
H	-9.34600	4.99830	-2.84720
H	-9.33710	6.54440	-4.77980
H	-7.23130	7.64410	-5.47660
H	-5.13520	7.18980	-4.23660
O	-2.43970	2.34590	-1.76730
O	-3.71660	6.07540	-2.47260
O	-3.05260	9.38850	-1.54970

Methyl β-D-Gal*f* **2**,C2-exo, 180°/180°

	**X**	**Y**	**Z**
O	0.61460	3.82940	2.99500
O	-3.76180	5.81660	1.47240
O	-0.59650	6.82410	4.45800
O	1.02380	6.33010	1.28180
O	-1.48170	4.76060	2.53230
O	-2.10560	8.96730	1.41360
C	0.18030	6.09330	3.50030
C	-0.10240	6.65810	2.10810
C	-1.36500	5.90680	1.66050
C	-2.67620	6.68520	1.78130
C	-0.39560	4.68490	3.44090
C	-2.78460	7.85270	0.81430
H	1.23410	6.11280	3.75790
H	-0.23690	7.73410	2.12330
H	-2.77300	7.06270	2.80520
H	-0.79990	4.34120	4.39720
H	-1.25300	5.55660	0.63200
H	-3.83550	8.10500	0.67070
H	-2.33650	7.62650	-0.15220
C	-0.06700	7.98180	4.91340
C	1.18700	7.05720	0.15880
C	-1.83920	10.02170	0.62850
C	0.21990	2.45900	2.93930
H	-0.14520	2.12540	3.91620
H	-0.55880	2.30410	2.18990
H	1.10580	1.88950	2.66700
C	-1.06770	11.07110	1.34210
C	-0.80770	10.99120	2.71210
C	-0.08320	11.99390	3.34110
C	0.38960	13.07780	2.60700
C	0.13250	13.16090	1.24080

C	-0.59670	12.16280	0.61020
H	-1.17850	10.15230	3.28070
H	0.11200	11.92870	4.40320
H	0.95740	13.85770	3.09870
H	0.50070	14.00310	0.66920
H	-0.80410	12.21570	-0.44980
C	-1.01750	8.74450	5.75460
C	-2.36260	8.38260	5.86300
C	-0.54710	9.88170	6.41530
C	-1.41320	10.65020	7.17800
C	-2.75460	10.29010	7.28110
C	-3.22720	9.15780	6.62360
H	-3.64530	5.01490	2.00030
H	-2.72870	7.50790	5.34550
H	0.49490	10.15400	6.31920
H	-1.04590	11.53070	7.68890
H	-3.43190	10.89320	7.87250
H	-4.27050	8.88100	6.70120
C	2.37600	6.63420	-0.61930
C	3.17380	5.55740	-0.22370
C	4.27510	5.19590	-0.98780
C	4.58590	5.90510	-2.14460
C	3.79200	6.97850	-2.54100
C	2.68940	7.34210	-1.78190
H	2.92910	5.00720	0.67330
H	4.89160	4.36080	-0.68120
H	5.44600	5.62120	-2.73790
H	4.03330	7.52980	-3.44060
H	2.06350	8.17260	-2.07820
O	1.05450	8.34350	4.63660
O	0.43420	7.95120	-0.16000
O	-2.19730	10.09610	-0.52710

Methyl β-D-Gal*f* **2**,C3-exo, +60°/+60°

	X	Y	Z
C	3.61300	4.74390	-2.47340
O	3.69420	4.57160	-3.88490
C	2.12650	4.63940	-2.11370
C	1.43200	4.37250	-3.44990
C	2.43630	4.92710	-4.46180
O	4.06980	6.00010	-2.06850
H	4.21650	3.95090	-2.02410
O	0.18180	5.04810	-3.56360
C	5.47810	6.17560	-2.23070
H	5.72150	7.15110	-1.81550
H	6.02550	5.39840	-1.68790
H	5.75830	6.14670	-3.28560
H	1.77620	5.57420	-1.68280
O	1.94090	3.57350	-1.17740
C	0.88420	3.65880	-0.34020
C	-0.94070	4.33610	-3.33300
H	1.28350	3.30230	-3.57090
C	2.38840	4.38440	-5.88430
C	2.39940	2.87040	-5.95260
H	3.28560	4.75460	-6.39430
O	1.22020	4.92590	-6.49180
O	2.53760	2.53990	-7.35000
H	3.23770	2.45540	-5.39390
H	1.46770	2.44460	-5.58170
H	1.19350	4.62700	-7.40950
H	2.32570	6.01560	-4.51710
C	2.50050	1.23660	-7.67180
C	0.76200	2.46780	0.53420
C	-0.29640	2.42650	1.44500
C	1.65910	1.39890	0.46320
C	1.49640	0.30230	1.29880
C	0.44090	0.26540	2.20550
C	-0.45540	1.32880	2.27780
H	-0.98670	3.25770	1.48850

H	2.47600	1.42700	-0.24320
H	2.19220	-0.52490	1.24200
H	0.31600	-0.59190	2.85490
H	-1.27710	1.30000	2.98160
C	-2.16590	5.15780	-3.47850
C	-2.11730	6.50050	-3.86200
C	-3.29340	7.22810	-3.98450
C	-4.51960	6.62300	-3.72410
C	-4.57100	5.28530	-3.34060
C	-3.39850	4.55420	-3.21860
H	-1.16500	6.96850	-4.06400
H	-3.25380	8.26780	-4.28290
H	-5.43490	7.19360	-3.81950
H	-5.52410	4.81460	-3.13690
H	-3.42390	3.51500	-2.92080
C	2.62040	1.00480	-9.13410
C	2.76320	2.05710	-10.04220
C	2.87040	1.79070	-11.40070
C	2.83540	0.47700	-11.85970
C	2.69300	-0.57390	-10.95740
C	2.58620	-0.31180	-9.59920
H	2.79060	3.07650	-9.68540
H	2.98110	2.60780	-12.10180
H	2.91890	0.27240	-12.91970
H	2.66540	-1.59570	-11.31330
H	2.47530	-1.11890	-8.88830
O	2.37920	0.35880	-6.84420
O	-0.93230	3.16050	-3.04230
O	0.13210	4.60710	-0.32100

Methyl β-D-Gal*f* **2**,C3-exo, +60°/-60°

	**X**	**Y**	**Z**
C	-0.43800	5.33240	-3.80230
O	0.80410	4.75910	-4.16100

C	-1.41090	4.82910	-4.86000
C	-0.54360	4.72200	-6.12270
C	0.90550	4.74840	-5.59100
O	-0.43130	6.72930	-3.87980
H	-0.66350	4.99150	-2.78890
O	-0.77150	5.86450	-6.95700
C	0.44690	7.35010	-2.94110
H	0.30730	8.42370	-3.04590
H	0.19490	7.04450	-1.92050
H	1.48800	7.09430	-3.14760
H	-2.26610	5.48240	-5.00260
O	-1.83460	3.52840	-4.43380
C	-2.91390	3.00620	-5.05390
C	-1.18110	5.65630	-8.22530
H	-0.76980	3.82530	-6.68150
C	1.82070	3.59380	-5.98910
C	1.38260	2.20360	-5.55940
H	2.77050	3.76790	-5.46730
O	1.99510	3.70770	-7.39530
O	0.22300	1.80150	-6.30920
H	2.18750	1.49220	-5.74800
H	1.15200	2.19270	-4.49380
H	2.60610	3.02230	-7.69320
H	1.37360	5.67170	-5.94140
C	-0.17360	0.52580	-6.18090
C	-3.23390	1.64200	-4.57450
C	-4.36600	1.00950	-5.09170
C	-2.42550	0.97570	-3.65060
C	-2.74880	-0.31360	-3.25270
C	-3.87950	-0.93960	-3.76820
C	-4.68800	-0.27610	-4.68690
H	-4.97530	1.53000	-5.81690
H	-1.54260	1.45970	-3.25960
H	-2.11490	-0.83300	-2.54580
H	-4.12650	-1.94770	-3.46010

H	-5.56090	-0.76730	-5.09640
C	-1.40380	6.93050	-8.95330
C	-1.23490	8.17420	-8.33900
C	-1.45990	9.33890	-9.06050
C	-1.85210	9.27000	-10.39420
C	-2.02070	8.03200	-11.00920
C	-1.79830	6.86580	-10.29170
H	-0.93170	8.22600	-7.30350
H	-1.32970	10.30100	-8.58210
H	-2.02670	10.18020	-10.95410
H	-2.32580	7.97750	-12.04630
H	-1.92680	5.89840	-10.75720
C	-1.35380	0.21050	-7.02310
C	-1.88700	1.13390	-7.92590
C	-3.00470	0.79850	-8.67700
C	-3.59260	-0.45500	-8.53530
C	-3.05840	-1.37840	-7.64130
C	-1.94280	-1.04770	-6.88780
H	-1.43640	2.10810	-8.04350
H	-3.41890	1.51840	-9.37110
H	-4.46780	-0.71110	-9.11910
H	-3.51930	-2.35060	-7.52400
H	-1.52680	-1.75000	-6.17940
O	0.38240	-0.26650	-5.44930
O	-1.34280	4.55620	-8.70510
O	-3.53190	3.60490	-5.90630

Methyl β-D-Gal*f* **2**,C3-exo, +60°/180°

	**X**	**Y**	**Z**
C	5.56770	5.35460	-5.56070
O	4.50990	4.92380	-6.39510
C	5.12840	5.01290	-4.13770
C	3.59890	4.90770	-4.22970
C	3.28290	5.19120	-5.70940

O	5.75770	6.74170	-5.60920
H	6.46440	4.82170	-5.88550
O	2.97620	5.88070	-3.38910
C	6.18670	7.21330	-6.88650
H	6.37590	8.27900	-6.77840
H	7.10730	6.70580	-7.19250
H	5.41750	7.05300	-7.64450
H	5.43320	5.77330	-3.42390
O	5.73220	3.75570	-3.79780
C	5.59020	3.35240	-2.51870
C	1.97180	5.47520	-2.58510
H	3.28060	3.91900	-3.92050
C	2.15180	4.39800	-6.36300
C	2.03170	2.94060	-5.93570
H	2.32240	4.44380	-7.44390
O	0.94660	5.07490	-6.01460
O	3.27840	2.29150	-6.22640
H	1.80310	2.86060	-4.87450
H	1.23150	2.45360	-6.49750
H	0.20810	4.65480	-6.47320
H	3.02460	6.24990	-5.80480
C	3.51730	1.10680	-5.64540
C	6.27470	2.06740	-2.24360
C	6.07980	1.47350	-0.99380
C	7.10580	1.45370	-3.18320
C	7.74080	0.26030	-2.86850
C	7.54270	-0.33000	-1.62440
C	6.70870	0.27610	-0.68770
H	5.43310	1.95800	-0.27520
H	7.25080	1.90520	-4.15280
H	8.38350	-0.21230	-3.59840
H	8.03630	-1.26340	-1.38440
H	6.55170	-0.18440	0.27910
C	1.44730	6.58140	-1.74680
C	1.98610	7.86960	-1.79630

C	1.46260	8.87060	-0.98900
C	0.40200	8.59320	-0.13130
C	-0.13740	7.31040	-0.08010
C	0.38320	6.30730	-0.88440
H	2.80940	8.08360	-2.46210
H	1.88220	9.86750	-1.02850
H	-0.00400	9.37600	0.49700
H	-0.96210	7.09390	0.58660
H	-0.02560	5.30680	-0.85370
C	4.87370	0.59430	-5.96490
C	5.81210	1.37900	-6.63970
C	7.07420	0.86760	-6.90970
C	7.40230	-0.42670	-6.51630
C	6.46830	-1.20990	-5.84430
C	5.20920	-0.70000	-5.56470
H	5.55470	2.38560	-6.93540
H	7.80350	1.47970	-7.42460
H	8.38750	-0.82260	-6.72810
H	6.72560	-2.21340	-5.53150
H	4.47780	-1.29520	-5.03550
O	2.70940	0.53550	-4.94400
O	1.55360	4.33920	-2.56110
O	4.96370	3.99050	-1.70080

Methyl β-D-Gal*f* **2**,C3-exo, -60°/+60°

	**X**	**Y**	**Z**
C	2.48350	4.34990	-2.24210
O	2.76980	3.91150	-3.56620
C	1.54320	5.55430	-2.38020
C	1.28730	5.65380	-3.88050
C	2.53180	4.99580	-4.46700
O	3.63130	4.77040	-1.56470
H	2.01890	3.50770	-1.72240
O	1.18650	6.99860	-4.34930

C	4.52840	3.70500	-1.24690
H	5.33170	4.13800	-0.65490
H	4.01540	2.93450	-0.66230
H	4.94200	3.25660	-2.15250
H	2.03340	6.45480	-2.01750
O	0.35100	5.31630	-1.62720
C	-0.29590	6.39180	-1.12930
C	-0.04890	7.53170	-4.46460
H	0.39460	5.09570	-4.15080
C	2.37650	4.49480	-5.88890
C	3.62740	3.76930	-6.34490
H	2.23750	5.38110	-6.52060
O	1.23220	3.65400	-5.96690
O	3.41790	3.44760	-7.73480
H	4.50900	4.40430	-6.24770
H	3.77690	2.85070	-5.77970
H	1.12130	3.38590	-6.88770
H	3.36750	5.70550	-4.44450
C	4.37840	2.73920	-8.34940
C	-1.54570	6.01880	-0.42400
C	-2.28530	7.03410	0.18710
C	-1.99960	4.69860	-0.36650
C	-3.18170	4.40130	0.29830
C	-3.91410	5.41570	0.90850
C	-3.46460	6.73250	0.85220
H	-1.92650	8.05270	0.13370
H	-1.43200	3.91270	-0.84290
H	-3.53260	3.37830	0.33990
H	-4.83540	5.18050	1.42640
H	-4.03430	7.52190	1.32520
C	-0.01240	8.93810	-4.93310
C	1.18800	9.59760	-5.21000
C	1.16970	10.91810	-5.63840
C	-0.04190	11.58590	-5.79170
C	-1.23980	10.93160	-5.51570

C	-1.22600	9.61230	-5.08770
H	2.12780	9.07940	-5.08770
H	2.10080	11.42710	-5.85160
H	-0.05290	12.61590	-6.12530
H	-2.18210	11.45080	-5.63330
H	-2.14880	9.09350	-4.86750
C	4.05300	2.44760	-9.76930
C	2.85980	2.87370	-10.35840
C	2.59890	2.57650	-11.68940
C	3.52400	1.85490	-12.43820
C	4.71410	1.42860	-11.85420
C	4.97830	1.72370	-10.52460
H	2.14210	3.43410	-9.77710
H	1.67370	2.90800	-12.14300
H	3.31810	1.62500	-13.47600
H	5.43400	0.86720	-12.43550
H	5.89860	1.39800	-10.05950
O	5.39770	2.39150	-7.79220
O	-1.06020	6.91820	-4.20790
O	0.11370	7.52460	-1.25010

Methyl β-D-Gal*f* **2**,C3-exo, -60°/-60°

	**X**	**Y**	**Z**
C	0.05550	1.92180	-4.19280
O	0.93100	2.34620	-5.22700
C	-0.46630	3.19680	-3.51720
C	0.18410	4.32290	-4.31500
C	1.42420	3.64470	-4.89280
O	0.71400	1.15770	-3.22250
H	-0.73630	1.33650	-4.66810
O	0.56880	5.43510	-3.50720
C	1.18360	-0.09930	-3.71170
H	1.58200	-0.63940	-2.85560
H	0.36120	-0.67010	-4.15550

H	1.96900	0.03790	-4.45690
H	-0.14600	3.23050	-2.47860
O	-1.89590	3.21870	-3.58250
C	-2.55110	3.89360	-2.61430
C	-0.27180	6.49010	-3.44380
H	-0.47570	4.65050	-5.11480
C	2.01860	4.36260	-6.09380
C	3.36530	3.81540	-6.53520
H	2.21690	5.38710	-5.75240
O	1.05950	4.36770	-7.14350
O	3.21000	2.44190	-6.92200
H	3.74370	4.38820	-7.38380
H	4.08640	3.88660	-5.71930
H	1.30840	5.04650	-7.78220
H	2.19790	3.57700	-4.11870
C	4.31730	1.74870	-7.20840
C	-4.02080	3.87440	-2.81040
C	-4.81700	4.55160	-1.88370
C	-4.61830	3.21040	-3.88490
C	-5.99950	3.22430	-4.02550
C	-6.78930	3.89940	-3.09920
C	-6.19650	4.56360	-2.02840
H	-4.34370	5.06400	-1.05770
H	-4.00450	2.68840	-4.60430
H	-6.46030	2.70900	-4.85830
H	-7.86610	3.90890	-3.21210
H	-6.80990	5.08990	-1.30860
C	0.22830	7.55120	-2.53680
C	1.43330	7.42520	-1.84070
C	1.85600	8.44420	-0.99790
C	1.08120	9.59040	-0.84450
C	-0.12100	9.71830	-1.53560
C	-0.54670	8.70270	-2.37900
H	2.03240	6.53410	-1.95820
H	2.78950	8.34420	-0.45940

H	1.41310	10.38300	-0.18580
H	-0.72480	10.60840	-1.41550
H	-1.47900	8.78860	-2.91990
C	4.02070	0.32310	-7.50630
C	2.72900	-0.19610	-7.38360
C	2.49390	-1.53730	-7.65540
C	3.54130	-2.36370	-8.05280
C	4.82910	-1.84820	-8.17670
C	5.06920	-0.50930	-7.90250
H	1.92120	0.44870	-7.06910
H	1.49360	-1.93870	-7.55560
H	3.35480	-3.40900	-8.26480
H	5.64380	-2.49050	-8.48550
H	6.06510	-0.09730	-7.99160
O	5.42860	2.23800	-7.21250
O	-1.31060	6.54790	-4.06250
O	-1.98750	4.44600	-1.69590

Methyl β-D-Gal*f* **2**,C3-exo, -60°/180°

	**X**	**Y**	**Z**
C	2.29570	7.49570	-4.42940
O	2.59620	6.16750	-4.83100
C	0.86660	7.77760	-4.91470
C	0.48580	6.52250	-5.70170
C	1.83850	5.88850	-6.01140
O	3.14090	8.43400	-5.03390
H	2.39540	7.52900	-3.34150
O	-0.19880	6.81350	-6.92090
C	4.50190	8.32810	-4.61460
H	5.03860	9.14890	-5.08520
H	4.57570	8.41720	-3.52590
H	4.93710	7.37780	-4.92990
H	0.84900	8.65550	-5.55500
O	0.01580	7.98710	-3.78250

C	-1.05510	8.79190	-3.95020
C	-1.54630	6.72980	-6.92570
H	-0.11590	5.86100	-5.08430
C	1.80170	4.39770	-6.28310
C	3.19590	3.81790	-6.48930
H	1.21480	4.25900	-7.19740
O	1.18660	3.73540	-5.18440
O	3.78300	4.50190	-7.61070
H	3.81000	3.96830	-5.60260
H	3.13340	2.75060	-6.70390
H	0.85010	2.88340	-5.48640
H	2.28540	6.38520	-6.87810
C	5.05130	4.19330	-7.92020
C	-1.88670	8.88290	-2.72570
C	-3.02220	9.69570	-2.75960
C	-1.57080	8.17980	-1.56020
C	-2.38480	8.29340	-0.44120
C	-3.51450	9.10560	-0.47880
C	-3.83240	9.80670	-1.63920
H	-3.25860	10.23290	-3.66770
H	-0.69530	7.54780	-1.53310
H	-2.13850	7.74740	0.46030
H	-4.14770	9.19150	0.39530
H	-4.71140	10.43750	-1.66930
C	-2.12350	7.08660	-8.24480
C	-1.32300	7.45250	-9.33030
C	-1.91070	7.78160	-10.54430
C	-3.29550	7.74870	-10.68170
C	-4.09590	7.38590	-9.60150
C	-3.51270	7.05590	-8.38690
H	-0.24870	7.47960	-9.22180
H	-1.28860	8.06470	-11.38360
H	-3.75080	8.00680	-11.62960
H	-5.17280	7.36170	-9.70720
H	-4.12300	6.77440	-7.53990

C	5.57170	5.00140	-9.05280
C	4.81430	6.01480	-9.64610
C	5.34440	6.75160	-10.69680
C	6.62840	6.48120	-11.16140
C	7.38540	5.47140	-10.57270
C	6.86000	4.73450	-9.52100
H	3.81840	6.22460	-9.28350
H	4.75640	7.53760	-11.15290
H	7.03920	7.05690	-11.98120
H	8.38370	5.26030	-10.93350
H	7.43810	3.94890	-9.05420
O	5.68770	3.34740	-7.32820
O	-2.19460	6.40070	-5.95800
O	-1.29440	9.36710	-4.98830

Methyl β-D-Gal*f* **2**,C3-exo, 180°/+60°

	**X**	**Y**	**Z**
C	3.85220	5.82920	-2.85690
O	3.62420	5.64930	-4.24870
C	3.29720	4.57630	-2.16770
C	2.72390	3.74190	-3.31430
C	2.49770	4.78390	-4.40920
O	3.17090	6.93870	-2.34690
H	4.93110	5.94600	-2.72660
O	1.48710	3.10910	-2.98420
C	3.66850	8.18520	-2.83550
H	3.11650	8.96690	-2.31820
H	4.73670	8.28330	-2.61620
H	3.51020	8.27590	-3.91190
H	2.51330	4.84720	-1.46520
O	4.35170	3.89920	-1.47990
C	4.03820	3.24730	-0.33840
C	1.53400	1.83930	-2.52290
H	3.45190	2.99470	-3.62830

C	2.48160	4.25680	-5.83080
C	1.43370	3.17060	-6.01190
H	3.47120	3.84180	-6.05720
O	2.20950	5.36770	-6.67920
O	1.46010	2.82680	-7.41070
H	1.66370	2.28260	-5.42610
H	0.43920	3.52960	-5.74680
H	2.16480	5.04270	-7.58780
H	1.56510	5.32720	-4.21740
C	0.54290	1.93980	-7.83340
C	5.19990	2.51990	0.22750
C	5.02300	1.83070	1.42950
C	6.44420	2.49910	-0.40780
C	7.49890	1.79560	0.15830
C	7.31880	1.11170	1.35720
C	6.07960	1.13000	1.99230
H	4.05500	1.85120	1.91100
H	6.58230	3.02790	-1.33950
H	8.46150	1.77980	-0.33620
H	8.14320	0.56370	1.79580
H	5.93860	0.59750	2.92390
C	0.18280	1.27380	-2.30440
C	-0.97420	1.94620	-2.70650
C	-2.21830	1.36690	-2.49760
C	-2.31440	0.12120	-1.88380
C	-1.16290	-0.54980	-1.47980
C	0.08260	0.02250	-1.69170
H	-0.89770	2.91160	-3.18500
H	-3.11330	1.88650	-2.81450
H	-3.28640	-0.32700	-1.72080
H	-1.23770	-1.51780	-1.00150
H	0.98490	-0.48850	-1.38520
C	0.63780	1.67060	-9.29060
C	1.59420	2.29160	-10.09830
C	1.64330	2.00840	-11.45670

C	0.74150	1.10720	-12.01530
C	-0.21290	0.48700	-11.21320
C	-0.26510	0.76700	-9.85540
H	2.29480	2.99020	-9.66430
H	2.38520	2.49080	-12.08000
H	0.78220	0.88840	-13.07490
H	-0.91460	-0.21380	-11.64680
H	-1.00150	0.29230	-9.22170
O	-0.26490	1.42650	-7.08950
O	2.57190	1.24840	-2.32720
O	2.93290	3.26680	0.15410

Methyl β-D-Gal*f* **2**,C3-exo, 180°/-60°

	**X**	**Y**	**Z**
C	2.03530	7.76020	-4.16560
O	2.74740	6.67590	-4.73030
C	0.89870	7.12480	-3.36420
C	0.76240	5.71260	-3.95700
C	1.78240	5.68750	-5.10900
O	1.44710	8.58020	-5.13720
H	2.74470	8.32200	-3.55330
O	-0.55460	5.49610	-4.46390
C	2.39650	9.24760	-5.96920
H	1.82910	9.90540	-6.62390
H	3.08780	9.84070	-5.36170
H	2.96440	8.53380	-6.56880
H	-0.02690	7.68470	-3.46190
O	1.30560	7.09100	-1.98910
C	0.33820	6.84550	-1.08110
C	-1.27740	4.46240	-3.98260
H	0.99140	4.96890	-3.19760
C	2.50420	4.36970	-5.33690
C	1.56820	3.18320	-5.48710
H	3.13170	4.17030	-4.45960

O	3.31300	4.55450	-6.49650
O	0.60650	3.48980	-6.51310
H	2.13700	2.29780	-5.77620
H	1.04990	2.96680	-4.55360
H	3.88800	3.78570	-6.59720
H	1.28040	5.98650	-6.03260
C	-0.38690	2.60910	-6.70860
C	0.85070	6.84420	0.31030
C	-0.05430	6.59480	1.34490
C	2.19540	7.08260	0.60610
C	2.62590	7.07200	1.92600
C	1.72080	6.82400	2.95410
C	0.38030	6.58510	2.66210
H	-1.09220	6.41040	1.10410
H	2.89710	7.27420	-0.19260
H	3.66780	7.25720	2.15330
H	2.05970	6.81650	3.98230
H	-0.32370	6.39150	3.46110
C	-2.59400	4.34580	-4.65120
C	-3.01570	5.25840	-5.62140
C	-4.24470	5.08980	-6.24160
C	-5.05560	4.01190	-5.90150
C	-4.63840	3.10000	-4.93600
C	-3.41130	3.26550	-4.31200
H	-2.37850	6.08630	-5.89400
H	-4.56390	5.79100	-7.00100
H	-6.00940	3.87700	-6.39570
H	-5.26680	2.25800	-4.67610
H	-3.07050	2.55980	-3.56740
C	-1.35440	3.06900	-7.73550
C	-1.15820	4.25030	-8.45540
C	-2.09740	4.65240	-9.39420
C	-3.23660	3.88380	-9.61520
C	-3.43560	2.70870	-8.89680
C	-2.49690	2.30070	-7.96100

H	-0.27750	4.84920	-8.27520
H	-1.94410	5.56830	-9.95030
H	-3.97150	4.20350	-10.34320
H	-4.32620	2.11600	-9.06060
H	-2.64460	1.39570	-7.38870
O	-0.47530	1.56740	-6.09330
O	-0.87080	3.70690	-3.12770
O	-0.81600	6.65130	-1.39260

Methyl β-D-Gal*f* **2**, C3-exo, 180°/180°

	**X**	**Y**	**Z**
C	5.84260	3.69280	-3.78490
O	5.02450	4.39990	-4.70020
C	5.76240	2.23190	-4.23630
C	4.44550	2.15950	-5.02190
C	3.87490	3.58590	-4.95130
O	5.35680	3.75030	-2.47450
H	6.84250	4.12700	-3.85580
O	3.48140	1.25960	-4.46860
C	5.38540	5.06220	-1.91130
H	5.05880	4.96560	-0.87820
H	6.40180	5.46790	-1.93890
H	4.71310	5.73530	-2.44690
H	5.78760	1.55010	-3.39220
O	6.82410	1.92860	-5.15050
C	8.00910	1.57490	-4.61830
C	3.70970	-0.05780	-4.65460
H	4.66010	1.86210	-6.04590
C	3.17900	4.08200	-6.20700
C	1.90340	3.30710	-6.52490
H	3.87630	4.01820	-7.04970
O	2.81130	5.43610	-5.95670
O	2.24700	2.00590	-7.03040
H	1.28180	3.20480	-5.63580

H	1.33650	3.84530	-7.28630
H	2.52940	5.83660	-6.78880
H	3.17790	3.65200	-4.10940
C	1.24950	1.10700	-7.09970
C	9.01140	1.22630	-5.65530
C	10.30100	0.88710	-5.23980
C	8.69750	1.22470	-7.01690
C	9.66790	0.88660	-7.95050
C	10.95230	0.55010	-7.53240
C	11.26790	0.55090	-6.17610
H	10.53240	0.89080	-4.18350
H	7.69980	1.48420	-7.33980
H	9.42230	0.88450	-9.00460
H	11.70690	0.28710	-8.26290
H	12.26640	0.28950	-5.85040
C	2.54110	-0.89420	-4.29790
C	1.32960	-0.33380	-3.88600
C	0.24240	-1.15470	-3.62310
C	0.35880	-2.53430	-3.76700
C	1.56660	-3.09520	-4.17280
C	2.65450	-2.27840	-4.43910
H	1.23680	0.73790	-3.78940
H	-0.69820	-0.71840	-3.31290
H	-0.49300	-3.17190	-3.56670
H	1.65510	-4.16720	-4.29120
H	3.59320	-2.69860	-4.77200
C	1.71450	-0.23160	-7.53640
C	3.02830	-0.46050	-7.94980
C	3.42700	-1.73940	-8.31260
C	2.52050	-2.79350	-8.26100
C	1.20870	-2.56730	-7.85210
C	0.80540	-1.29080	-7.49410
H	3.73540	0.35540	-7.97440
H	4.44780	-1.91510	-8.62620
H	2.83680	-3.79210	-8.53510

H	0.50560	-3.38860	-7.80330
H	-0.20580	-1.10400	-7.16120
O	0.10220	1.37570	-6.81370
O	4.75770	-0.48170	-5.08860
O	8.21310	1.54940	-3.42400

Methyl β-D-Galf **2**, O4-exo, +60°/+60°

	**X**	**Y**	**Z**
C	-0.10980	2.24720	-6.41220
O	-1.51770	2.12700	-6.49140
C	0.24180	1.68760	-5.03500
C	-0.96140	2.09300	-4.17750
C	-2.08550	2.37000	-5.19600
O	0.32410	3.57500	-6.44160
H	0.30230	1.67700	-7.24820
O	-0.70850	3.30960	-3.46260
C	0.08150	4.23060	-7.68660
H	0.52630	5.22020	-7.61020
H	0.55080	3.67890	-8.50740
H	-0.98920	4.32400	-7.87920
O	0.25040	0.25560	-5.07500
C	1.39520	-0.33890	-5.47820
C	-0.03290	3.20680	-2.29930
C	-3.35530	1.52440	-5.05580
C	-3.08030	0.03530	-5.10170
H	-4.00170	1.78060	-5.89760
O	-4.07560	1.91070	-3.89150
O	-4.34950	-0.63280	-5.22190
C	-4.32740	-1.97240	-5.16750
H	-2.38030	3.41730	-5.10440
C	0.15470	4.52050	-1.63770
C	-5.68130	-2.57250	-5.28260
C	-6.83270	-1.78670	-5.37850
C	-8.07830	-2.39200	-5.47860

C	-8.18190	-3.78010	-5.48480
C	-7.03620	-4.56590	-5.38990
C	-5.78990	-3.96490	-5.28840
H	-6.75080	-0.70940	-5.37090
H	-8.96880	-1.78110	-5.55130
H	-9.15450	-4.24930	-5.56330
H	-7.11580	-5.64530	-5.39490
H	-4.89260	-4.56370	-5.21350
C	1.28970	-1.81690	-5.49600
C	0.08540	-2.47720	-5.23940
C	0.03530	-3.86410	-5.27970
C	1.18250	-4.59690	-5.56910
C	2.38480	-3.94110	-5.82410
C	2.43870	-2.55560	-5.79090
H	-0.81070	-1.91890	-5.01840
H	-0.90190	-4.36890	-5.08630
H	1.14070	-5.67850	-5.59750
H	3.27720	-4.51040	-6.04940
H	3.36500	-2.03420	-5.98960
C	0.79650	4.54910	-0.39730
C	0.99170	5.75640	0.25720
C	0.54990	6.94220	-0.32410
C	-0.08760	6.91830	-1.56130
C	-0.28710	5.71200	-2.21900
H	1.13540	3.62150	0.04300
H	1.48770	5.77450	1.21900
H	0.70320	7.88450	0.18670
H	-0.42930	7.84020	-2.01370
H	-0.77990	5.69220	-3.18000
H	1.18570	2.06590	-4.65620
H	-1.19230	1.30780	-3.46310
H	-3.64370	1.54590	-3.10780
O	0.36500	2.14890	-1.86250
O	2.38430	0.29010	-5.78230
O	-3.30250	-2.61050	-5.04140

H	-2.58330	-0.31080	-4.19450
H	-2.45610	-0.21390	-5.95850

Methyl β-D-Galf **2**, O4-exo, +60°/-60°

	**X**	**Y**	**Z**
C	-0.56080	2.24690	-3.30630
O	-1.50110	2.14970	-4.35790
C	-1.20170	1.49520	-2.14760
C	-2.70280	1.72400	-2.35350
C	-2.81930	2.25700	-3.80380
O	-0.36440	3.56370	-2.87660
H	0.36890	1.80220	-3.66930
O	-3.15420	2.69270	-1.39560
C	0.23050	4.40600	-3.86420
H	0.40220	5.36960	-3.38970
H	1.18400	3.98680	-4.20090
H	-0.43130	4.53340	-4.72300
O	-0.88730	0.10900	-2.33020
C	-1.15930	-0.71260	-1.29240
C	-4.44390	2.63690	-1.03220
C	-3.79310	1.54700	-4.75010
C	-3.52740	0.06920	-4.98820
H	-3.65910	2.02400	-5.72480
O	-5.13560	1.79640	-4.36970
O	-3.85010	-0.66530	-3.79130
C	-3.77840	-2.00400	-3.85530
H	-3.12050	3.30710	-3.75200
C	-4.78980	3.62140	0.01610
C	-4.13810	-2.65340	-2.57020
C	-4.58780	-1.91930	-1.46960
C	-4.88730	-2.56870	-0.28050
C	-4.74520	-3.94990	-0.18390
C	-4.30500	-4.68400	-1.28130
C	-4.00210	-4.03870	-2.47040

H	-4.70380	-0.84800	-1.54170
H	-5.22990	-1.99640	0.57190
H	-4.97510	-4.45320	0.74680
H	-4.18680	-5.75700	-1.20510
H	-3.64420	-4.59510	-3.32500
C	-0.87130	-2.13090	-1.60250
C	-0.47730	-2.54260	-2.87790
C	-0.24930	-3.88790	-3.12950
C	-0.40600	-4.82420	-2.11220
C	-0.79590	-4.41550	-0.83990
C	-1.03170	-3.07400	-0.58540
H	-0.36620	-1.81540	-3.66880
H	0.04430	-4.20690	-4.12110
H	-0.23170	-5.87380	-2.31280
H	-0.92840	-5.14500	-0.05180
H	-1.35120	-2.74500	0.39330
C	-6.11060	3.67010	0.46910
C	-6.47400	4.57820	1.45240
C	-5.52200	5.44250	1.98710
C	-4.20530	5.39760	1.53720
C	-3.83600	4.49000	0.55430
H	-6.83990	2.99400	0.04490
H	-7.49760	4.61380	1.80210
H	-5.80650	6.15140	2.75440
H	-3.46620	6.06990	1.95320
H	-2.81510	4.45370	0.20300
H	-0.86200	1.83960	-1.17570
H	-3.25230	0.80500	-2.20520
H	-5.24540	1.59400	-3.42400
O	-5.23450	1.85080	-1.52320
O	-1.59880	-0.30720	-0.23890
O	-3.44650	-2.59800	-4.85900
H	-2.48270	-0.10070	-5.24820
H	-4.15960	-0.28710	-5.80060

Methyl β-D-Galf **2**, O4-exo, +60°/180°

	X	Y	Z
C	-3.42920	2.63430	-8.43340
O	-4.19750	2.41240	-7.27180
C	-2.02600	2.13820	-8.08360
C	-1.92580	2.39890	-6.57600
C	-3.37860	2.62440	-6.11530
O	-3.30330	3.99400	-8.74810
H	-3.91370	2.08820	-9.24430
O	-1.18640	3.59490	-6.29240
C	-4.53820	4.60970	-9.11190
H	-4.30520	5.63170	-9.40230
H	-4.99450	4.08270	-9.95610
H	-5.23620	4.61770	-8.27220
O	-1.89330	0.72300	-8.26510
C	-1.74450	0.27760	-9.52990
C	0.15890	3.49930	-6.31180
C	-3.88980	1.74730	-4.96520
C	-3.61790	0.25090	-5.06400
H	-4.96890	1.90330	-4.91450
O	-3.37190	2.22190	-3.72630
O	-4.21690	-0.24460	-6.26690
C	-3.92940	-1.50350	-6.62490
H	-3.47090	3.66070	-5.78070
C	0.82140	4.78760	-5.99510
C	-4.46560	-1.83880	-7.96680
C	-5.08150	-0.87420	-8.76790
C	-5.51420	-1.20830	-10.04360
C	-5.33990	-2.50370	-10.52210
C	-4.73260	-3.46760	-9.72230
C	-4.29530	-3.13690	-8.44890
H	-5.20340	0.13150	-8.39380
H	-5.98170	-0.45720	-10.66710
H	-5.67150	-2.76050	-11.52030

H	-4.58820	-4.47210	-10.09780
H	-3.80410	-3.87030	-7.82550
C	-1.44920	-1.17270	-9.58560
C	-1.00090	-1.87430	-8.46490
C	-0.70770	-3.22650	-8.56770
C	-0.87020	-3.88510	-9.78240
C	-1.32270	-3.18890	-10.89970
C	-1.60600	-1.83500	-10.80440
H	-0.88340	-1.36290	-7.52110
H	-0.36020	-3.76830	-7.69770
H	-0.64890	-4.94230	-9.85770
H	-1.45860	-3.70330	-11.84210
H	-1.95960	-1.28300	-11.66420
C	2.21670	4.81480	-5.93290
C	2.87690	5.99900	-5.64030
C	2.14820	7.16330	-5.41090
C	0.75780	7.14090	-5.47440
C	0.09270	5.95750	-5.76490
H	2.77080	3.90410	-6.11400
H	3.95800	6.01610	-5.59140
H	2.66390	8.08780	-5.18340
H	0.19170	8.04630	-5.29760
H	-0.98620	5.93900	-5.81660
H	-1.26120	2.65900	-8.65270
H	-1.43790	1.56010	-6.08690
H	-2.42460	2.03530	-3.67750
O	0.73960	2.46560	-6.56230
O	-1.83880	1.00370	-10.49530
O	-3.28540	-2.25910	-5.92720
H	-4.05420	-0.24830	-4.19820
H	-2.54770	0.03620	-5.07630

Methyl β-D-Galf **2**, O4-exo, -60°/+60°

	X	Y	Z
C	0.15190	2.49620	-5.75880
O	-1.07140	1.83590	-6.03250
C	-0.13570	3.31730	-4.50280
C	-1.61270	3.69550	-4.65950
C	-2.15490	2.73040	-5.72850
O	0.51550	3.39330	-6.76470
H	0.90610	1.71720	-5.62620
O	-1.77340	5.03130	-5.15370
C	0.83010	2.76510	-8.00820
H	1.16640	3.55230	-8.67910
H	1.62970	2.02990	-7.87340
H	-0.04720	2.27210	-8.43180
O	-0.04860	2.48430	-3.34110
C	1.17730	2.28360	-2.81550
C	-1.67860	6.03080	-4.25250
C	-3.33960	1.89460	-5.26080
C	-3.84430	1.00690	-6.38400
H	-4.13860	2.58010	-4.96900
O	-3.01840	1.14680	-4.09580
O	-5.05730	0.38550	-5.92200
C	-5.65690	-0.47140	-6.76030
H	-2.43140	3.30230	-6.61730
C	-1.87960	7.36590	-4.86610
C	-6.87850	-1.08200	-6.17410
C	-7.27550	-0.82450	-4.85930
C	-8.42060	-1.42330	-4.35110
C	-9.17500	-2.27830	-5.14950
C	-8.78220	-2.53670	-6.46020
C	-7.63730	-1.94210	-6.97080
H	-6.68750	-0.16300	-4.23980
H	-8.72440	-1.22370	-3.33160
H	-10.06780	-2.74330	-4.75070
H	-9.36800	-3.20150	-7.08180
H	-7.32110	-2.13610	-7.98650

C	1.13740	1.40650	-1.62060
C	-0.05330	0.83470	-1.16390
C	-0.04150	0.01640	-0.04250
C	1.15270	-0.23290	0.62790
C	2.34040	0.33570	0.17480
C	2.33400	1.15210	-0.94670
H	-0.97940	1.02930	-1.68510
H	-0.96430	-0.42660	0.30930
H	1.15820	-0.87060	1.50290
H	3.26900	0.14210	0.69590
H	3.24930	1.59950	-1.30910
C	-1.83880	8.48710	-4.03400
C	-2.02070	9.75520	-4.56590
C	-2.24220	9.91120	-5.93190
C	-2.28180	8.79660	-6.76480
C	-2.10190	7.52540	-6.23640
H	-1.66510	8.35170	-2.97530
H	-1.98960	10.62150	-3.91800
H	-2.38340	10.90120	-6.34690
H	-2.45270	8.91820	-7.82660
H	-2.13020	6.65980	-6.88210
H	0.52100	4.17630	-4.40740
H	-2.12760	3.59470	-3.70860
H	-2.21070	0.64590	-4.27840
O	-1.45210	5.83640	-3.07830
O	2.18050	2.77480	-3.28380
O	-5.23640	-0.71190	-7.87310
H	-3.11550	0.23590	-6.63530
H	-4.05720	1.59660	-7.27700

Methyl β-D-Galf **2**, O4-exo, -60°/-60°

	**X**	**Y**	**Z**
C	-0.98690	0.04860	-3.07010
O	-2.06890	0.09760	-3.98060

C	-0.92280	1.45740	-2.48320
C	-1.40440	2.34250	-3.63800
C	-2.04830	1.37010	-4.64530
O	0.23370	-0.19730	-3.70240
H	-1.22380	-0.73220	-2.34380
O	-0.31980	3.00330	-4.30160
C	0.30860	-1.48610	-4.31460
H	1.32170	-1.59200	-4.69580
H	0.10790	-2.27110	-3.57850
H	-0.40470	-1.57290	-5.13640
O	-1.88910	1.59360	-1.43420
C	-1.53680	1.15100	-0.21000
C	0.17060	4.11390	-3.71300
C	-3.47710	1.75050	-5.03500
C	-3.99130	1.02590	-6.26640
H	-3.46910	2.81280	-5.29430
O	-4.35310	1.61170	-3.92530
O	-3.92970	-0.38830	-6.01900
C	-4.26500	-1.21340	-7.01760
H	-1.42200	1.32000	-5.53910
C	1.27800	4.72350	-4.48840
C	-4.03220	-2.63740	-6.66250
C	-3.36340	-2.99580	-5.48860
C	-3.14320	-4.33620	-5.20080
C	-3.59180	-5.32170	-6.07590
C	-4.25930	-4.96630	-7.24540
C	-4.47610	-3.62780	-7.54050
H	-3.01140	-2.22550	-4.81720
H	-2.61960	-4.61250	-4.29450
H	-3.42020	-6.36620	-5.84790
H	-4.60800	-5.73250	-7.92580
H	-4.98860	-3.33810	-8.44770
C	-2.61970	1.33690	0.78590
C	-3.86530	1.86310	0.43280
C	-4.85060	2.01520	1.39900

C	-4.59930	1.64590	2.71740
C	-3.35910	1.12090	3.07170
C	-2.37210	0.96530	2.10940
H	-4.05980	2.14710	-0.59120
H	-5.81510	2.42190	1.12360
H	-5.36960	1.76670	3.46850
H	-3.16340	0.83360	4.09660
H	-1.40530	0.55820	2.37150
C	1.86470	5.89080	-3.99380
C	2.90470	6.49390	-4.68560
C	3.36590	5.93430	-5.87450
C	2.78500	4.77050	-6.37010
C	1.74320	4.16400	-5.68140
H	1.49770	6.31440	-3.06910
H	3.35650	7.39840	-4.29960
H	4.17820	6.40500	-6.41400
H	3.14450	4.33520	-7.29350
H	1.29290	3.25970	-6.06420
H	0.06870	1.71650	-2.12560
H	-2.10880	3.08260	-3.26840
H	-4.16320	0.75520	-3.51610
O	-0.25990	4.54520	-2.66580
O	-0.45390	0.66130	0.02450
O	-4.69620	-0.82750	-8.08430
H	-3.37790	1.26830	-7.13600
H	-5.02250	1.31710	-6.46660

Methyl β-D-Galf **2**, O4-exo, -60°/180°

	**X**	**Y**	**Z**
C	-1.95240	4.87140	-6.67900
O	-2.45390	3.54860	-6.74530
C	-1.79340	5.13820	-5.18260
C	-2.93780	4.33110	-4.55860
C	-3.38810	3.36560	-5.66830

O	-2.85740	5.81400	-7.17130
H	-1.01680	4.88230	-7.24260
O	-4.05990	5.15740	-4.22310
C	-3.12000	5.68250	-8.56920
H	-3.76710	6.51230	-8.84410
H	-2.18780	5.73780	-9.14020
H	-3.62220	4.73780	-8.78660
O	-0.57550	4.55230	-4.70810
C	0.55730	5.26630	-4.87100
C	-3.98570	5.85490	-3.07150
C	-3.36320	1.89770	-5.26900
C	-3.86940	0.98790	-6.38150
H	-4.00310	1.77820	-4.39190
O	-2.06080	1.48480	-4.88080
O	-5.21930	1.39070	-6.67380
C	-5.83430	0.78640	-7.70120
H	-4.39280	3.64100	-5.99180
C	-5.21210	6.64810	-2.81360
C	-7.19540	1.32990	-7.94430
C	-7.70670	2.39850	-7.20280
C	-8.98050	2.88140	-7.47090
C	-9.74980	2.30200	-8.47600
C	-9.24350	1.23650	-9.21590
C	-7.97030	0.75220	-8.95220
H	-7.10810	2.84860	-6.42400
H	-9.37330	3.71050	-6.89650
H	-10.74300	2.68040	-8.68280
H	-9.84120	0.78520	-9.99730
H	-7.56500	-0.07360	-9.52060
C	1.74880	4.55240	-4.35210
C	1.66170	3.26810	-3.80780
C	2.80590	2.63730	-3.33850
C	4.03760	3.28200	-3.40810
C	4.12710	4.56160	-3.95020
C	2.98690	5.19530	-4.42210

H	0.70510	2.76870	-3.75500
H	2.73700	1.64230	-2.91800
H	4.92780	2.78730	-3.04040
H	5.08470	5.06290	-4.00460
H	3.04240	6.18840	-4.84630
C	-5.26700	7.41950	-1.65050
C	-6.39500	8.17670	-1.37040
C	-7.47460	8.16800	-2.24990
C	-7.42360	7.40120	-3.41060
C	-6.29670	6.64190	-3.69490
H	-4.42190	7.41720	-0.97590
H	-6.43390	8.77320	-0.46820
H	-8.35490	8.75920	-2.03090
H	-8.26240	7.39540	-4.09450
H	-6.25510	6.04790	-4.59620
H	-1.82910	6.19550	-4.94010
H	-2.58850	3.81420	-3.66920
H	-1.44200	1.78120	-5.56380
O	-3.01590	5.82350	-2.34580
O	0.57360	6.36270	-5.38530
O	-5.31940	-0.09920	-8.35030
H	-3.85760	-0.05040	-6.05140
H	-3.25540	1.08490	-7.27720

Methyl β-D-Galf **2**, O4-exo, 180°/+60°

	**X**	**Y**	**Z**
C	-0.76240	3.11910	-7.49640
O	-2.09810	2.97120	-7.04520
C	-0.06910	1.79920	-7.14430
C	-0.95550	1.20020	-6.04250
C	-2.04840	2.25270	-5.81070
O	-0.08190	4.14060	-6.82370
H	-0.81080	3.31560	-8.57020
O	-0.27350	0.98180	-4.80440

C	-0.61220	5.44210	-7.07780
H	0.03620	6.14890	-6.56490
H	-0.60930	5.65310	-8.15200
H	-1.63050	5.53340	-6.69490
O	-0.08740	0.93040	-8.28110
C	1.09240	0.52900	-8.79990
C	0.52120	-0.10760	-4.73280
C	-3.43210	1.69420	-5.50500
C	-3.39780	0.83920	-4.24920
H	-3.75160	1.08020	-6.35030
O	-4.39670	2.73360	-5.40660
O	-4.70110	0.25290	-4.09810
C	-4.88820	-0.52520	-3.02150
H	-1.73490	2.91980	-4.99930
C	1.09680	-0.30040	-3.38140
C	-6.26580	-1.07660	-2.94890
C	-7.24220	-0.75430	-3.89510
C	-8.51780	-1.29170	-3.78520
C	-8.82540	-2.15160	-2.73470
C	-7.85460	-2.47460	-1.79010
C	-6.57930	-1.93880	-1.89580
H	-7.00260	-0.08470	-4.70830
H	-9.27250	-1.03950	-4.51890
H	-9.82080	-2.56950	-2.65190
H	-8.09290	-3.14310	-0.97300
H	-5.81680	-2.18160	-1.16860
C	0.91110	-0.44260	-9.90700
C	-0.35270	-0.88720	-10.30420
C	-0.47020	-1.80300	-11.34110
C	0.66830	-2.27910	-11.98520
C	1.92900	-1.83780	-11.59170
C	2.05090	-0.92270	-10.55620
H	-1.23530	-0.51840	-9.80260
H	-1.45010	-2.14680	-11.64620
H	0.57320	-2.99380	-12.79290

H	2.81470	-2.20760	-12.09190
H	3.02410	-0.57310	-10.24050
C	2.01890	-1.33340	-3.19780
C	2.57590	-1.55360	-1.94670
C	2.21280	-0.74600	-0.87190
C	1.29210	0.28270	-1.05030
C	0.73450	0.50890	-2.30130
H	2.29080	-1.95380	-4.04080
H	3.29190	-2.35320	-1.80800
H	2.64670	-0.91910	0.10490
H	1.00790	0.90810	-0.21400
H	0.01790	1.30500	-2.44090
H	0.95200	1.96030	-6.81400
H	-1.37320	0.25880	-6.39730
H	-4.16860	3.30180	-4.65790
O	0.72200	-0.82990	-5.68390
O	2.16490	0.92290	-8.40090
O	-4.01710	-0.74410	-2.20560
H	-3.16800	1.44580	-3.37060
H	-2.65680	0.04440	-4.32240

Methyl β-D-Galf **2**, O4-exo, 180°/-60°

	**X**	**Y**	**Z**
C	-2.16090	5.17730	-6.00070
O	-2.88690	4.04410	-6.43850
C	-1.05990	4.60920	-5.10650
C	-1.68340	3.32290	-4.54490
C	-3.00340	3.15640	-5.31990
O	-2.93030	6.03720	-5.20730
H	-1.79080	5.68380	-6.89530
O	-2.00460	3.43170	-3.15530
C	-4.02150	6.63680	-5.90620
H	-4.49030	7.33300	-5.21430
H	-3.66120	7.17940	-6.78610

H	-4.74870	5.88500	-6.21920
O	0.05800	4.25360	-5.92850
C	1.25700	4.81690	-5.66320
C	-1.07130	3.04510	-2.25800
C	-3.29480	1.74950	-5.83060
C	-3.18170	0.69980	-4.73930
H	-2.55840	1.50020	-6.59840
O	-4.56120	1.70230	-6.47970
O	-4.07330	1.07230	-3.66830
C	-3.73280	0.71620	-2.41790
H	-3.82270	3.48160	-4.67320
C	-1.54820	3.20770	-0.86490
C	-4.65080	1.27630	-1.39690
C	-5.62190	2.22670	-1.72040
C	-6.43490	2.75130	-0.72530
C	-6.28780	2.32640	0.59190
C	-5.32190	1.37810	0.91580
C	-4.50310	0.85710	-0.07400
H	-5.72840	2.56050	-2.74230
H	-7.18120	3.49410	-0.97590
H	-6.92140	2.73930	1.36670
H	-5.20090	1.05450	1.94130
H	-3.73840	0.13190	0.16700
C	2.31730	4.32160	-6.57540
C	2.05350	3.38950	-7.58250
C	3.08000	2.95600	-8.41070
C	4.37100	3.44730	-8.23900
C	4.63700	4.37600	-7.23610
C	3.61420	4.81260	-6.40690
H	1.05140	3.00890	-7.71500
H	2.87320	2.23410	-9.19020
H	5.16940	3.10680	-8.88610
H	5.64060	4.75820	-7.10170
H	3.80810	5.53290	-5.62420
C	-0.83590	2.57940	0.15820

C	-1.25820	2.70200	1.47380
C	-2.38360	3.46440	1.77480
C	-3.09050	4.09830	0.75820
C	-2.68130	3.96600	-0.56040
H	0.03750	1.99190	-0.08970
H	-0.71150	2.20430	2.26430
H	-2.71310	3.55890	2.80180
H	-3.96980	4.68350	0.99110
H	-3.23610	4.44650	-1.35230
H	-0.75220	5.30040	-4.32900
H	-0.99470	2.49590	-4.69630
H	-5.24810	1.88460	-5.82430
O	0.01210	2.61060	-2.57880
O	1.43140	5.62860	-4.78280
O	-2.77030	0.02270	-2.16620
H	-2.16680	0.62000	-4.35680
H	-3.48820	-0.27150	-5.12780

Methyl β-D-Galf **2**, O4-exo, 180°/180°

	**X**	**Y**	**Z**
C	-2.00220	0.85720	-8.90020
O	-2.66270	1.64130	-7.92430
C	-2.46560	-0.57260	-8.62220
C	-2.81500	-0.56080	-7.12680
C	-2.63100	0.90760	-6.69610
O	-0.61100	0.87590	-8.75440
H	-2.30410	1.24580	-9.87530
O	-1.95270	-1.37580	-6.32700
C	-0.02860	2.15840	-8.98800
H	1.04960	2.02710	-8.93010
H	-0.30120	2.52700	-9.98210
H	-0.35120	2.88030	-8.23490
O	-3.67810	-0.85270	-9.33400
C	-3.55830	-1.24160	-10.61810

C	-2.10150	-2.71230	-6.44890
C	-3.69480	1.47480	-5.76330
C	-3.65490	0.85920	-4.36870
H	-4.67990	1.32470	-6.21040
O	-3.54530	2.88320	-5.62170
O	-4.08840	-0.50990	-4.43410
C	-3.68810	-1.32810	-3.44480
H	-1.64710	1.01380	-6.22420
C	-1.31940	-3.46610	-5.44280
C	-4.13520	-2.72750	-3.64750
C	-4.99060	-3.08770	-4.69070
C	-5.35290	-4.41690	-4.86050
C	-4.86290	-5.38950	-3.99490
C	-4.01380	-5.03190	-2.95060
C	-3.65380	-3.70570	-2.77460
H	-5.36220	-2.33280	-5.36790
H	-6.01120	-4.69470	-5.67340
H	-5.13870	-6.42690	-4.13620
H	-3.62560	-5.78950	-2.28260
H	-2.98300	-3.41650	-1.97820
C	-4.87120	-1.53550	-11.24310
C	-6.06990	-1.42400	-10.53340
C	-7.27470	-1.71060	-11.16080
C	-7.29050	-2.10840	-12.49470
C	-6.09750	-2.22050	-13.20420
C	-4.89130	-1.93520	-12.58120
H	-6.05610	-1.11600	-9.49800
H	-8.20200	-1.62400	-10.60930
H	-8.23190	-2.33100	-12.98110
H	-6.10920	-2.52990	-14.24130
H	-3.95800	-2.01810	-13.12090
C	-1.39000	-4.85990	-5.46420
C	-0.71430	-5.60450	-4.50970
C	0.03350	-4.96130	-3.52750
C	0.10820	-3.57130	-3.50440

C	-0.56440	-2.82270	-4.45930
H	-1.98720	-5.34580	-6.22290
H	-0.77740	-6.68480	-4.52420
H	0.55530	-5.54280	-2.77790
H	0.68560	-3.07110	-2.73780
H	-0.51990	-1.74390	-4.43400
H	-1.70440	-1.30260	-8.87590
H	-3.84040	-0.89900	-6.99730
H	-2.64060	3.07710	-5.34140
O	-2.81520	-3.21390	-7.28900
O	-2.48830	-1.33600	-11.17910
O	-3.02130	-0.95120	-2.50470
H	-4.33070	1.41380	-3.71750
H	-2.64800	0.90630	-3.95340

Methyl β-D-Galf **2**, C2-endo, +60°/+60°

	**X**	**Y**	**Z**
C	3.88600	3.73180	-0.45120
H	4.02730	4.78190	-0.21500
C	2.82110	3.06940	0.43520
H	3.26280	2.58650	1.29970
C	2.10300	2.08650	-0.51240
C	2.15210	0.61780	-0.10960
H	1.65550	0.04500	-0.90160
O	1.41350	0.52270	1.10340
C	3.36490	3.49000	-1.86180
H	4.14970	3.40910	-2.61790
H	1.05240	2.37870	-0.57300
O	2.47620	4.52700	-2.16080
O	5.12340	3.01650	-0.35540
O	1.89000	4.05910	0.88540
O	2.73120	2.22640	-1.79430
C	3.55790	0.07070	0.05050
H	4.09190	0.54270	0.87410

H	4.13410	0.18470	-0.86610
O	3.38850	-1.33170	0.34740
H	1.44270	-0.39480	1.40320
C	5.92640	3.31420	0.68930
C	1.75800	4.26150	2.21430
C	4.49390	-2.03820	0.63180
C	4.19590	-3.45740	0.95460
C	2.89060	-3.95580	0.96860
C	2.66110	-5.28950	1.27940
C	3.72930	-6.13110	1.57650
C	5.03130	-5.63760	1.56310
C	5.26460	-4.30550	1.25350
O	5.60550	-1.55470	0.62090
C	7.15860	2.49120	0.70310
C	8.08450	2.70610	1.72710
C	9.24890	1.95460	1.78010
C	9.49500	0.98420	0.81180
C	8.57430	0.76650	-0.20880
C	7.40800	1.51660	-0.26630
O	5.64730	4.16150	1.50800
C	0.76720	5.32470	2.51570
O	2.37590	3.64140	3.04990
C	0.50280	5.61950	3.85510
C	-0.41540	6.60650	4.18270
C	-1.07420	7.30590	3.17470
C	-0.81260	7.01600	1.83860
C	0.10480	6.02830	1.50620
H	2.06190	-3.30220	0.73790
H	1.64890	-5.67270	1.29030
H	3.54730	-7.17070	1.81840
H	5.86240	-6.29130	1.79410
H	6.27080	-3.90980	1.24010
H	7.88000	3.46140	2.47340
H	9.96390	2.12280	2.57490
H	10.40320	0.39630	0.85460

H	8.76270	0.00820	-0.95770
H	6.68950	1.34330	-1.05360
H	1.02130	5.06970	4.62840
H	-0.61790	6.83140	5.22180
H	-1.79050	8.07650	3.43040
H	-1.32380	7.56040	1.05510
H	0.31010	5.80340	0.46980
C	1.90660	4.43380	-3.46660
H	1.30350	5.32770	-3.60840
H	2.69390	4.39720	-4.22650
H	1.27530	3.54780	-3.55770

Methyl β-D-Galf **2**, C2-endo, +60°/-60°

	**X**	**Y**	**Z**
C	3.78840	3.09100	-0.62260
H	3.49350	4.12920	-0.76950
C	2.84270	2.40910	0.36400
H	3.38660	1.76330	1.03750
C	1.86260	1.62460	-0.52860
C	1.75910	0.12440	-0.22370
H	0.98880	-0.27500	-0.89470
O	1.33510	0.03970	1.13160
C	3.61280	2.31800	-1.92230
H	4.31400	1.47440	-1.97670
H	0.86400	2.04670	-0.41110
O	3.79310	3.15320	-3.00840
O	5.16920	3.01820	-0.25990
O	2.12040	3.37560	1.13750
O	2.27150	1.83130	-1.88310
C	3.00200	-0.70130	-0.50670
H	3.29840	-0.59800	-1.55000
H	2.79640	-1.75580	-0.31250
O	4.07170	-0.27160	0.35450
H	1.14720	-0.88230	1.34710

C	5.63470	3.91920	0.62730
C	2.40320	3.46670	2.45250
C	5.28950	-0.79700	0.14380
C	6.29570	-0.30780	1.11930
C	5.96030	0.56380	2.15810
C	6.93800	0.99510	3.04370
C	8.25030	0.55780	2.90300
C	8.58750	-0.31480	1.87220
C	7.61550	-0.74520	0.98230
O	5.52010	-1.58270	-0.75160
C	7.08890	3.76870	0.86920
C	7.66840	4.51910	1.89440
C	9.02500	4.40350	2.15820
C	9.81090	3.54490	1.39410
C	9.23650	2.79830	0.37040
C	7.87840	2.90400	0.10870
O	4.92610	4.74680	1.15660
C	1.65770	4.56910	3.10920
O	3.17260	2.72730	3.02600
C	1.80900	4.73810	4.48740
C	1.13870	5.76140	5.14170
C	0.31480	6.62400	4.42330
C	0.16260	6.46040	3.04940
C	0.83060	5.43640	2.39120
H	4.94440	0.90890	2.27620
H	6.67510	1.68070	3.83850
H	9.01220	0.90400	3.58940
H	9.61010	-0.65040	1.75770
H	7.86660	-1.41850	0.17420
H	7.04590	5.18110	2.48030
H	9.47040	4.98050	2.95840
H	10.86970	3.45310	1.60120
H	9.84520	2.12290	-0.21610
H	7.42870	2.31310	-0.67510
H	2.45330	4.06290	5.03330

H	1.25840	5.88820	6.20990
H	-0.20720	7.42360	4.93370
H	-0.47600	7.13210	2.49030
H	0.71600	5.31050	1.32460
C	3.90270	2.46690	-4.25850
H	4.15630	3.21650	-5.00460
H	4.69370	1.71240	-4.21160
H	2.95830	1.98860	-4.52620

Methyl β-D-Gal*f* **2**, C2-endo, +60°/180°

	**X**	**Y**	**Z**
C	3.77590	3.18740	-1.22960
H	3.34660	4.17620	-1.38140
C	2.97510	2.40200	-0.20970
H	3.58740	1.60570	0.20540
C	1.81630	1.87220	-1.05960
C	1.12610	0.58750	-0.59750
H	0.57970	0.20140	-1.46480
O	0.22260	0.97570	0.43480
C	3.63060	2.35280	-2.49360
H	4.42630	1.60340	-2.57350
H	1.04230	2.64560	-1.10950
O	3.64330	3.17860	-3.60950
O	5.15330	3.29830	-0.87740
O	2.52160	3.25220	0.84160
O	2.38280	1.67000	-2.36020
C	2.04350	-0.50110	-0.05260
H	1.45540	-1.38490	0.20450
H	2.56410	-0.16010	0.83970
O	2.98990	-0.84660	-1.07740
H	-0.33460	0.22140	0.66320
C	5.59600	4.47180	-0.37350
C	2.62640	2.81620	2.11470
C	4.12260	-1.45790	-0.70300

C	5.06280	-1.63640	-1.83990
C	4.74960	-1.19780	-3.12920
C	5.66680	-1.36070	-4.15850
C	6.89710	-1.96200	-3.90870
C	7.21090	-2.40280	-2.62550
C	6.29710	-2.24110	-1.59390
O	4.34710	-1.80480	0.43720
C	7.04170	4.42470	-0.04440
C	7.63510	5.57510	0.48080
C	8.98430	5.57740	0.80260
C	9.74870	4.43070	0.60280
C	9.16110	3.28180	0.08110
C	7.81110	3.27510	-0.24290
O	4.88550	5.43880	-0.21730
C	2.13730	3.82140	3.08890
O	3.07350	1.73120	2.41300
C	2.11950	3.47370	4.44170
C	1.67560	4.38510	5.38850
C	1.24990	5.64980	4.99040
C	1.26800	6.00080	3.64350
C	1.70840	5.09070	2.69210
H	3.79600	-0.72730	-3.31850
H	5.42300	-1.01570	-5.15500
H	7.61150	-2.08640	-4.71280
H	8.16750	-2.87000	-2.43050
H	6.52970	-2.57570	-0.59220
H	7.03000	6.45850	0.63150
H	9.44050	6.47080	1.20900
H	10.80180	4.43260	0.85430
H	9.75520	2.39030	-0.07320
H	7.35420	2.38360	-0.64700
H	2.45460	2.48900	4.73720
H	1.66160	4.11160	6.43560
H	0.90480	6.36160	5.72970
H	0.93920	6.98460	3.33450

H	1.72570	5.36260	1.64690
C	3.76780	2.47060	-4.84520
H	3.86470	3.22010	-5.62740
H	4.65740	1.83380	-4.83330
H	2.88480	1.85640	-5.03520

Methyl β-D-Galf **2**, C2-endo, -60°/+60°

	**X**	**Y**	**Z**
C	3.77130	3.51710	-0.94500
H	3.44210	4.53920	-1.12410
C	2.86310	2.82480	0.05010
H	3.38420	2.00630	0.54050
C	1.71340	2.30470	-0.82840
C	1.21810	0.92480	-0.42300
H	0.81380	1.01000	0.59210
O	2.32620	0.03110	-0.43620
C	3.58740	2.69300	-2.20890
H	4.22600	1.79960	-2.20540
H	0.87560	3.00530	-0.77670
O	3.84720	3.46530	-3.32620
O	5.13840	3.50290	-0.54070
O	2.42030	3.76650	1.03160
O	2.21600	2.29340	-2.17440
C	0.11780	0.44910	-1.35000
H	0.48930	0.31120	-2.36410
H	-0.71830	1.14980	-1.36070
O	-0.32720	-0.81920	-0.82650
H	2.02090	-0.83190	-0.13040
C	5.67240	4.63870	-0.03980
C	2.17230	3.31730	2.27870
C	-1.26090	-1.47710	-1.53090
C	-1.63030	-2.77800	-0.91540
C	-1.03890	-3.23410	0.26560
C	-1.41710	-4.45740	0.80280

C	-2.38480	-5.23020	0.16710
C	-2.97600	-4.77870	-1.01000
C	-2.60060	-3.55690	-1.54980
O	-1.74230	-1.04070	-2.55480
C	7.09230	4.46080	0.35240
C	7.76950	5.55900	0.88770
C	9.09720	5.43880	1.27100
C	9.75640	4.22110	1.12290
C	9.08510	3.12410	0.59050
C	7.75630	3.24010	0.20520
O	5.05390	5.67310	0.07030
C	1.82980	4.41890	3.21090
O	2.22820	2.14660	2.58510
C	1.43730	4.08700	4.50980
C	1.11690	5.08670	5.41650
C	1.19050	6.42330	5.03270
C	1.58420	6.75820	3.74030
C	1.90200	5.76100	2.82810
H	-0.28890	-2.63330	0.75930
H	-0.95730	-4.80810	1.71770
H	-2.67800	-6.18350	0.58850
H	-3.72840	-5.37890	-1.50500
H	-3.05200	-3.19490	-2.46330
H	7.24580	6.49850	0.99810
H	9.61860	6.29200	1.68530
H	10.79250	4.12730	1.42280
H	9.59730	2.17760	0.47580
H	7.23440	2.38910	-0.20740
H	1.38640	3.04540	4.79560
H	0.81080	4.82590	6.42130
H	0.94210	7.20350	5.74120
H	1.64470	7.79720	3.44320
H	2.21200	6.01930	1.82610
C	3.93290	2.71960	-4.54340
H	4.25460	3.41620	-5.31420

H	4.66670	1.91370	-4.44730
H	2.96230	2.29810	-4.81300

Methyl β-D-Galf **2**, C2-endo, -60°/-60°

	**X**	**Y**	**Z**
C	3.89260	3.95720	-0.72140
H	3.84170	5.02610	-0.53730
C	3.18140	3.14800	0.36740
H	3.88320	2.75370	1.09560
C	2.44720	2.02150	-0.39840
C	2.79210	0.62300	0.09840
H	2.57510	0.63320	1.17170
O	4.18170	0.39950	-0.12230
C	3.17880	3.52910	-1.99690
H	3.81240	3.54690	-2.88730
H	1.36960	2.17210	-0.28730
O	2.06640	4.36740	-2.15270
O	5.25420	3.53460	-0.86010
O	2.26220	4.03440	1.02450
O	2.82250	2.18470	-1.77420
C	1.92480	-0.47970	-0.48290
H	0.87930	-0.31540	-0.21580
H	2.23650	-1.44720	-0.08520
O	2.05710	-0.48770	-1.91230
H	4.47190	-0.31500	0.45720
C	6.13310	3.99760	0.05080
C	1.77710	3.64580	2.21720
C	1.27190	-1.31720	-2.60650
C	1.41960	-1.12190	-4.07250
C	2.14280	-0.04530	-4.59370
C	2.24260	0.11820	-5.96900
C	1.62930	-0.79030	-6.82730
C	0.90900	-1.86390	-6.30950
C	0.80080	-2.02770	-4.93570

O	0.52490	-2.12240	-2.08860
C	7.49940	3.46190	-0.16110
C	8.53200	3.94730	0.64470
C	9.82390	3.46880	0.48300
C	10.09090	2.49950	-0.48060
C	9.06380	2.01010	-1.28260
C	7.77000	2.48930	-1.12730
O	5.81900	4.76370	0.93540
C	0.86060	4.65070	2.80930
O	2.06780	2.58770	2.73360
C	0.31980	4.39050	4.07060
C	-0.54200	5.30400	4.65990
C	-0.87100	6.48080	3.99200
C	-0.33580	6.74280	2.73400
C	0.52900	5.83260	2.14150
H	2.61050	0.65920	-3.92040
H	2.79790	0.95540	-6.37210
H	1.71100	-0.66130	-7.89930
H	0.43210	-2.57020	-6.97670
H	0.24060	-2.85440	-4.52020
H	8.31040	4.69840	1.39040
H	10.62230	3.84910	1.10690
H	11.09910	2.12470	-0.60540
H	9.27110	1.25430	-2.02900
H	6.97060	2.10740	-1.74530
H	0.58320	3.47280	4.57820
H	-0.95770	5.10010	5.63810
H	-1.54430	7.19320	4.45190
H	-0.59230	7.65710	2.21460
H	0.94380	6.03350	1.16450
C	1.28030	4.05390	-3.30240
H	0.49740	4.80670	-3.36070
H	1.89420	4.09050	-4.20830
H	0.83100	3.06280	-3.21370

Methyl β-D-Galf **2**, C2-endo, -60°/180°

	**X**	**Y**	**Z**
C	3.99360	3.49040	-1.04530
H	3.58870	4.47860	-1.25690
C	3.09970	2.73710	-0.08210
H	3.66520	1.97720	0.45170
C	2.04530	2.10280	-1.00330
C	1.67540	0.67990	-0.60980
H	1.24850	0.72180	0.39650
O	2.86120	-0.10890	-0.62230
C	3.93760	2.63890	-2.30330
H	4.64360	1.79900	-2.25570
H	1.14150	2.71520	-0.99480
O	4.19090	3.41670	-3.41850
O	5.33810	3.59750	-0.58240
O	2.52590	3.65330	0.85600
O	2.60160	2.13520	-2.32860
C	0.66250	0.06060	-1.56170
H	0.44470	-0.96640	-1.26680
H	1.03730	0.06630	-2.58440
O	-0.53830	0.85010	-1.47720
H	2.73200	-0.87570	-0.05160
C	5.74900	4.77880	-0.07090
C	2.23480	3.19950	2.09130
C	-1.56300	0.48970	-2.26390
C	-2.73590	1.38990	-2.11830
C	-2.71440	2.50090	-1.27110
C	-3.83310	3.31720	-1.17040
C	-4.97600	3.03040	-1.91150
C	-5.00060	1.92370	-2.75620
C	-3.88450	1.10620	-2.86020
O	-1.51960	-0.46810	-3.00680
C	7.16460	4.73240	0.37140
C	7.72130	5.89020	0.92010

C	9.04100	5.89330	1.34710
C	9.81270	4.74000	1.22980
C	9.26170	3.58390	0.68450
C	7.94130	3.57660	0.25530
O	5.03580	5.75330	0.00990
C	1.74030	4.27900	2.98050
O	2.36750	2.04100	2.42110
C	1.29160	3.93090	4.25670
C	0.82910	4.91070	5.12280
C	0.81550	6.24400	4.72100
C	1.26420	6.59530	3.45110
C	1.72450	5.61760	2.57940
H	-1.82650	2.72310	-0.69700
H	-3.81340	4.17780	-0.51430
H	-5.84680	3.66880	-1.83090
H	-5.88890	1.69970	-3.33260
H	-3.89000	0.24430	-3.51320
H	7.11140	6.77890	1.00610
H	9.46870	6.79240	1.77140
H	10.84270	4.74230	1.56360
H	9.86150	2.68760	0.59360
H	7.51300	2.67980	-0.16790
H	1.30920	2.89240	4.55730
H	0.48020	4.63700	6.11010
H	0.45630	7.00890	5.39790
H	1.25670	7.63190	3.14010
H	2.07720	5.88910	1.59510
C	4.39270	2.66640	-4.61890
H	4.69410	3.37740	-5.38480
H	5.18260	1.92270	-4.47690
H	3.47230	2.16570	-4.92640

Methyl β-D-Galf **2**, C2-endo, 180°/+60°

	X	Y	Z
C	4.29050	3.44020	-0.78890
H	3.90940	4.45190	-0.90060
C	3.53470	2.70310	0.31800
H	4.21360	2.03860	0.85140
C	2.48640	1.90910	-0.46110
C	1.94930	0.66890	0.22590
H	2.77630	-0.03840	0.36130
O	0.96420	0.11260	-0.63860
C	4.00130	2.61810	-2.05150
H	4.90330	2.20070	-2.50620
H	1.64760	2.56300	-0.72650
O	3.32920	3.44540	-2.95680
O	5.69990	3.47690	-0.55340
O	2.88990	3.58310	1.23940
O	3.20580	1.51680	-1.63270
C	1.34870	0.99930	1.58280
H	0.55950	1.74620	1.49650
H	2.10110	1.35030	2.28670
O	0.78800	-0.23310	2.07480
H	0.58140	-0.65540	-0.19500
C	6.21260	4.59490	0.00590
C	3.55420	3.90600	2.37160
C	0.10260	-0.16760	3.22900
C	-0.46150	-1.48020	3.63350
C	-0.28450	-2.63210	2.86260
C	-0.83410	-3.83680	3.28040
C	-1.56130	-3.89890	4.46560
C	-1.73940	-2.75280	5.23620
C	-1.19190	-1.54720	4.82210
O	-0.02890	0.86150	3.85620
C	7.67140	4.48220	0.24670
C	8.32620	5.56610	0.83630
C	9.68980	5.50550	1.08270
C	10.40810	4.36230	0.74120

C	9.75920	3.27950	0.15470
C	8.39420	3.33560	-0.09250
O	5.54550	5.56940	0.27050
C	2.73890	4.74970	3.27570
O	4.68070	3.52970	2.60380
C	3.34830	5.27260	4.41870
C	2.61770	6.05710	5.29870
C	1.27350	6.31910	5.04540
C	0.66200	5.79720	3.90910
C	1.39060	5.01600	3.02270
H	0.28030	-2.58390	1.94290
H	-0.69560	-4.72750	2.68120
H	-1.98890	-4.83960	4.78870
H	-2.30460	-2.80020	6.15800
H	-1.32330	-0.64980	5.41100
H	7.75620	6.44730	1.09710
H	10.19340	6.34710	1.54030
H	11.47260	4.31520	0.93320
H	10.31730	2.39060	-0.10970
H	7.88960	2.49490	-0.54570
H	4.39200	5.05970	4.60440
H	3.09350	6.46380	6.18160
H	0.70280	6.92920	5.73400
H	-0.38360	5.99800	3.71450
H	0.91680	4.60790	2.14200
C	3.08540	2.82750	-4.22120
H	2.62960	3.58410	-4.85610
H	4.02520	2.48940	-4.66960
H	2.40710	1.97830	-4.11860

Methyl β-D-Galf **2**, C2-endo, 180°/-60°

	**X**	**Y**	**Z**
C	4.33660	3.98500	-0.69590
H	4.14400	5.05050	-0.61300

C	3.58530	3.18630	0.38270
H	4.29730	2.69420	1.04000
C	2.73580	2.17570	-0.41210
C	2.64650	0.77980	0.18150
H	3.65110	0.33970	0.17410
O	1.77160	0.03840	-0.66490
C	3.82410	3.40120	-2.01050
H	4.59340	3.29670	-2.77910
H	1.72840	2.58070	-0.53170
O	2.78000	4.22720	-2.44560
O	5.74740	3.73200	-0.64360
O	2.73230	4.03580	1.15160
O	3.38610	2.09570	-1.68600
C	2.17100	0.77380	1.62490
H	2.90900	1.22160	2.28930
H	1.99340	-0.25190	1.95250
O	0.94160	1.51870	1.69820
H	1.79360	-0.88830	-0.39480
C	6.45070	4.41570	0.28310
C	2.89230	4.07440	2.49240
C	0.42480	1.74180	2.91640
C	-0.80400	2.57240	2.86270
C	-1.37210	2.96910	1.64950
C	-2.51190	3.76020	1.64490
C	-3.08610	4.16380	2.84690
C	-2.52020	3.77230	4.05640
C	-1.38400	2.97710	4.06530
O	0.92850	1.31700	3.93470
C	7.89240	4.06930	0.27020
C	8.73430	4.71790	1.17660
C	10.08920	4.42160	1.20220
C	10.61100	3.47520	0.32390
C	9.77510	2.82560	-0.58000
C	8.41850	3.11960	-0.60950
O	5.93810	5.21670	1.03310

C	1.86820	4.91740	3.15120
O	3.75760	3.46090	3.07670
C	1.89210	5.01310	4.54430
C	0.93130	5.76280	5.20560
C	-0.05870	6.41920	4.47970
C	-0.08590	6.32610	3.09200
C	0.87270	5.57700	2.42620
H	-0.91900	2.66180	0.71830
H	-2.95000	4.06710	0.70390
H	-3.97110	4.78760	2.84070
H	-2.96020	4.09400	4.99140
H	-0.92920	2.67440	4.99810
H	8.31570	5.44930	1.85420
H	10.73860	4.92610	1.90580
H	11.66840	3.24360	0.34450
H	10.18060	2.08930	-1.26180
H	7.76840	2.61460	-1.30910
H	2.66110	4.49000	5.09540
H	0.94850	5.83050	6.28560
H	-0.81490	6.99590	4.99690
H	-0.86360	6.82510	2.52960
H	0.84390	5.49040	1.35030
C	2.18310	3.79550	-3.66880
H	1.45110	4.55220	-3.94230
H	2.93930	3.71400	-4.45620
H	1.68580	2.83160	-3.54500

Methyl β-D-Galf **2**, C2-endo, 180°/180°

	**X**	**Y**	**Z**
C	4.60420	3.89210	-0.56160
H	4.41930	4.95860	-0.48070
C	3.75940	3.06760	0.41950
H	4.42260	2.49950	1.06830
C	2.91640	2.14680	-0.47790

C	2.67250	0.74390	0.05110
H	3.63810	0.25960	0.23380
O	1.94410	0.05450	-0.96220
C	4.20200	3.35540	-1.93860
H	5.04030	3.22550	-2.62710
H	1.95060	2.62410	-0.67440
O	3.24560	4.22970	-2.46490
O	5.98270	3.60690	-0.29160
O	2.87280	3.84720	1.22680
O	3.67780	2.06360	-1.68630
C	1.82940	0.72440	1.32270
H	1.49980	-0.29700	1.51990
H	0.94990	1.35830	1.21280
O	2.62280	1.18180	2.43080
H	1.91680	-0.88420	-0.73820
C	6.89150	4.47820	-0.76880
C	3.43510	4.55480	2.22940
C	1.95760	1.48200	3.56020
C	2.83850	2.04770	4.61050
C	4.22350	2.12530	4.45290
C	5.00210	2.70030	5.44760
C	4.40380	3.20220	6.59910
C	3.02280	3.12350	6.75990
C	2.24260	2.54540	5.77130
O	0.76230	1.31820	3.68180
C	8.27680	4.12190	-0.37520
C	9.32430	4.90740	-0.86170
C	10.63460	4.61190	-0.51510
C	10.90630	3.53170	0.32060
C	9.86500	2.74740	0.80890
C	8.55230	3.03850	0.46310
O	6.59050	5.44050	-1.44140
C	2.43240	5.10630	3.16900
O	4.63350	4.69080	2.33680
C	2.88880	5.86490	4.24840

C	1.99020	6.34930	5.18620
C	0.63140	6.07720	5.05260
C	0.17220	5.32360	3.97610
C	1.06800	4.83880	3.03390
H	4.68550	1.74840	3.55240
H	6.07490	2.76520	5.32020
H	5.01250	3.65900	7.36920
H	2.55610	3.52210	7.65110
H	1.16830	2.49100	5.87630
H	9.09920	5.74380	-1.50900
H	11.44390	5.22200	-0.89490
H	11.92900	3.30170	0.59160
H	10.07630	1.90900	1.46010
H	7.74280	2.43220	0.84260
H	3.94820	6.05410	4.34900
H	2.34800	6.92960	6.02670
H	-0.06940	6.44890	5.78950
H	-0.88350	5.10770	3.87530
H	0.71570	4.24110	2.20630
C	2.79140	3.86290	-3.76800
H	2.11630	4.65000	-4.09640
H	3.63610	3.79060	-4.46080
H	2.26000	2.90950	-3.74570

Methyl β-D-Galf **2**, C1-exo, +60°/+60°

	**X**	**Y**	**Z**
C	4.33120	2.29640	-1.01880
H	4.22420	3.31060	-1.40090
C	3.24480	1.99540	0.00430
H	3.59070	1.24210	0.70720
C	2.07340	1.50920	-0.86320
C	1.30750	0.31140	-0.30220
H	0.47160	0.10650	-0.98130
O	0.81920	0.71490	0.97220

C	4.04830	1.30110	-2.13130
H	4.49630	0.32040	-1.91770
H	1.36560	2.33110	-0.98810
O	4.50950	1.78470	-3.33970
O	5.65310	2.09150	-0.52480
O	2.83920	3.15720	0.73220
O	2.62240	1.17850	-2.14550
C	2.15060	-0.94600	-0.20660
H	2.98220	-0.83160	0.48820
H	2.53200	-1.23790	-1.18400
O	1.26670	-1.97130	0.29040
H	0.32980	-0.02410	1.35570
C	6.30420	3.15750	-0.01070
C	3.42170	3.38270	1.92770
C	1.80540	-3.18090	0.51580
C	7.65400	2.80310	0.48870
C	8.15510	1.50090	0.41410
C	9.42510	1.21930	0.89910
C	10.19890	2.23140	1.45980
C	9.70160	3.52990	1.53680
C	8.43320	3.81560	1.05350
O	5.82420	4.26790	0.03000
C	0.82050	-4.15540	1.05030
C	-0.51130	-3.80730	1.29020
C	-1.39490	-4.75270	1.79330
C	-0.95640	-6.04690	2.05900
C	0.37010	-6.39710	1.82060
C	1.25610	-5.45520	1.31810
O	2.97200	-3.43060	0.29960
C	2.93580	4.63210	2.56120
C	1.96250	5.43690	1.96350
C	1.54340	6.60040	2.59450
C	2.09250	6.96710	3.82000
C	3.06390	6.16780	4.41710
C	3.48450	5.00380	3.79070

O	4.24750	2.64190	2.41400
H	7.55300	0.71590	-0.01970
H	9.81140	0.21000	0.84050
H	11.18880	2.00840	1.83750
H	10.30240	4.31690	1.97370
H	8.03410	4.81900	1.10840
H	-0.85110	-2.80280	1.08340
H	-2.42590	-4.48020	1.97860
H	-1.64770	-6.78200	2.45140
H	0.71180	-7.40310	2.02680
H	2.28870	-5.71420	1.12890
H	1.53890	5.15220	1.01150
H	0.78900	7.22200	2.12990
H	1.76440	7.87570	4.30890
H	3.49250	6.45340	5.36910
H	4.23910	4.37510	4.24270
C	4.50770	0.81840	-4.39460
H	4.99260	1.28690	-5.24780
H	5.06800	-0.07290	-4.09680
H	3.48780	0.53540	-4.66310

Methyl β-D-Galf **2**, C1-exo, +60°/-60°

	**X**	**Y**	**Z**
C	4.23830	2.42130	-0.92850
H	4.27360	3.47100	-1.21780
C	3.12990	2.19470	0.09810
H	3.45070	1.51080	0.87000
C	1.95710	1.63120	-0.72700
C	1.40470	0.28460	-0.24180
H	0.54690	0.05260	-0.88490
O	0.98070	0.50710	1.09770
C	3.83370	1.57220	-2.12570
H	4.24130	0.55560	-2.04280
H	1.13330	2.34510	-0.70120

O	4.26230	2.15830	-3.30140
O	5.53240	1.98380	-0.50780
O	2.73580	3.42820	0.71210
O	2.40790	1.52820	-2.08010
C	2.33780	-0.90650	-0.37490
H	2.64580	-1.03470	-1.41200
H	1.82410	-1.81530	-0.05510
O	3.49210	-0.70800	0.46090
H	0.50220	-0.27070	1.41020
C	6.24500	2.80420	0.28880
C	3.01510	3.59740	2.02000
C	4.49300	-1.59770	0.36230
C	7.59130	2.26430	0.59090
C	8.08790	1.12240	-0.04060
C	9.35650	0.65660	0.27210
C	10.13050	1.31970	1.21890
C	9.63560	2.45540	1.85460
C	8.37120	2.92960	1.53900
O	5.81290	3.85800	0.70100
C	5.60390	-1.30820	1.30340
C	5.54540	-0.25470	2.21880
C	6.61150	-0.02190	3.07630
C	7.73640	-0.83780	3.03030
C	7.79610	-1.89210	2.12330
C	6.73550	-2.12650	1.26190
O	4.47190	-2.52730	-0.41690
C	2.63230	4.94780	2.50240
C	2.11160	5.92120	1.64610
C	1.77590	7.17350	2.14310
C	1.95660	7.46080	3.49300
C	2.47600	6.49300	4.34900
C	2.81370	5.24110	3.85590
O	3.51350	2.74040	2.71600
H	7.48000	0.59970	-0.76390
H	9.73690	-0.23220	-0.21380

H	11.11690	0.94830	1.46660
H	10.23530	2.96870	2.59520
H	7.97310	3.80960	2.02520
H	4.67620	0.38390	2.26250
H	6.56620	0.80360	3.77450
H	8.57020	-0.64690	3.69340
H	8.67420	-2.52340	2.08200
H	6.77290	-2.93850	0.54890
H	1.97440	5.69790	0.59820
H	1.37400	7.92610	1.47710
H	1.69390	8.43830	3.87760
H	2.61760	6.71560	5.39870
H	3.21930	4.48160	4.50990
C	4.15180	1.31010	-4.44790
H	4.62630	1.83700	-5.27250
H	4.66810	0.36160	-4.27190
H	3.10520	1.11590	-4.69100

Methyl β-D-Galf **2**, C1-exo, +60°/180°

	**X**	**Y**	**Z**
C	4.29160	2.39340	-1.00500
H	4.30300	3.43530	-1.32100
C	3.13620	2.14090	-0.04380
H	3.40390	1.36500	0.66840
C	1.97850	1.72440	-0.96710
C	1.23200	0.46560	-0.51220
H	0.40930	0.29780	-1.21500
O	0.73710	0.78130	0.78540
C	3.96160	1.50760	-2.19480
H	4.29180	0.47360	-2.02780
H	1.25090	2.53610	-1.01250
O	4.53190	2.00720	-3.34800
O	5.56180	2.02230	-0.47330
O	2.76350	3.31930	0.67900

O	2.53650	1.55680	-2.27480
C	2.06340	-0.81190	-0.43640
H	1.51070	-1.56660	0.12690
H	3.02260	-0.65920	0.05730
O	2.27620	-1.29140	-1.77440
H	0.10280	0.10500	1.05400
C	6.29890	2.98270	0.12470
C	3.30010	3.49860	1.90330
C	3.20470	-2.24370	-1.94730
C	7.57540	2.45490	0.66280
C	7.93040	1.10870	0.54300
C	9.13530	0.66140	1.06780
C	9.98930	1.55120	1.71330
C	9.63750	2.89310	1.83540
C	8.43460	3.34420	1.31240
O	5.94180	4.13660	0.20410
C	3.39050	-2.59730	-3.37760
C	2.63640	-1.99850	-4.38950
C	2.85880	-2.34290	-5.71570
C	3.83280	-3.28320	-6.03910
C	4.58510	-3.88340	-5.03250
C	4.36430	-3.54290	-3.70580
O	3.82530	-2.73810	-1.02950
C	2.86790	4.77490	2.52290
C	1.99580	5.65520	1.87720
C	1.62510	6.84130	2.49640
C	2.12180	7.15540	3.75830
C	2.99230	6.28080	4.40360
C	3.36470	5.09450	3.78870
O	4.04810	2.70090	2.42410
H	7.26590	0.41850	0.04420
H	9.40810	-0.38180	0.97420
H	10.92800	1.19900	2.12210
H	10.30060	3.58500	2.33830
H	8.14870	4.38310	1.40190

H	1.88880	-1.26100	-4.13770
H	2.27520	-1.87480	-6.49780
H	4.00610	-3.54790	-7.07460
H	5.34290	-4.61440	-5.28320
H	4.94320	-3.99960	-2.91490
H	1.61310	5.41140	0.89700
H	0.94940	7.52150	1.99420
H	1.83150	8.08160	4.23810
H	3.38020	6.52530	5.38400
H	4.04140	4.40760	4.27790
C	4.49830	1.10230	-4.45590
H	5.09010	1.55660	-5.24730
H	4.93240	0.13840	-4.17660
H	3.47530	0.94890	-4.80410

Methyl β-D-Galf **2**, C1-exo, -60°/+60°

	**X**	**Y**	**Z**
C	4.58830	2.22890	-1.11740
H	4.39330	3.26270	-1.39270
C	3.54480	1.73530	-0.11990
H	3.97860	0.99340	0.54560
C	2.50030	1.09580	-1.02890
C	1.59440	0.09030	-0.34630
H	0.99040	0.65160	0.37760
O	2.39240	-0.87520	0.32680
C	4.40040	1.31460	-2.33550
H	5.26200	0.66610	-2.51550
H	1.87240	1.87720	-1.47350
O	4.15300	2.12190	-3.44920
O	5.92650	2.10240	-0.62890
O	2.94570	2.78580	0.63870
O	3.30020	0.46410	-2.03150
C	0.66350	-0.56430	-1.34840
H	1.21950	-1.16670	-2.06500

H	0.07050	0.18030	-1.88110
O	-0.21070	-1.41840	-0.58300
H	1.79980	-1.48710	0.78120
C	6.46920	3.17420	-0.01280
C	3.46060	3.05240	1.85850
C	-1.08360	-2.17220	-1.27040
C	7.83220	2.89630	0.50050
C	8.43790	1.64510	0.35920
C	9.71360	1.43260	0.86430
C	10.38940	2.46330	1.51120
C	9.78780	3.71110	1.65440
C	8.51330	3.92750	1.15160
O	5.89530	4.23440	0.09910
C	-1.90670	-3.04000	-0.38960
C	-1.74640	-3.05160	0.99850
C	-2.53730	-3.88250	1.78080
C	-3.48970	-4.70430	1.18480
C	-3.65200	-4.69510	-0.19820
C	-2.86350	-3.86640	-0.98320
O	-1.17640	-2.13420	-2.47890
C	2.76290	4.17590	2.52880
C	1.69650	4.85090	1.92910
C	1.07820	5.90000	2.59590
C	1.51960	6.28110	3.85980
C	2.58310	5.61120	4.45950
C	3.20340	4.56240	3.79690
O	4.38690	2.43560	2.33480
H	7.91130	0.84510	-0.14040
H	10.18060	0.46240	0.75450
H	11.38380	2.29420	1.90460
H	10.31230	4.51250	2.15850
H	8.03360	4.89070	1.25780
H	-1.00720	-2.41360	1.46090
H	-2.41060	-3.88960	2.85570
H	-4.10490	-5.35130	1.79730

H	-4.39220	-5.33380	-0.66260
H	-2.97930	-3.85050	-2.05820
H	1.35610	4.55580	0.94740
H	0.25240	6.42130	2.12930
H	1.03600	7.10020	4.37690
H	2.92760	5.90810	5.44160
H	4.03130	4.03530	4.25040
C	4.09290	1.39630	-4.67800
H	3.97510	2.13200	-5.47050
H	5.01790	0.83210	-4.83530
H	3.24420	0.70940	-4.68740

Methyl β-D-Galf **2**, C1-exo, -60°/-60°

	**X**	**Y**	**Z**
C	4.48950	2.51860	-1.11750
H	4.52410	3.58710	-1.31270
C	3.34340	2.17440	-0.16790
H	3.64050	1.36980	0.50030
C	2.24000	1.69980	-1.11250
C	1.19800	0.81680	-0.44370
H	0.82130	1.40080	0.40630
O	1.83400	-0.37020	0.01400
C	4.14840	1.74810	-2.40020
H	4.92010	1.02980	-2.68890
H	1.72880	2.56860	-1.54300
O	3.94310	2.68700	-3.41910
O	5.75940	2.07520	-0.62500
O	2.88930	3.29670	0.58840
O	2.97710	1.00040	-2.11710
C	-0.01620	0.53040	-1.31000
H	-0.51300	1.46290	-1.58280
H	-0.72610	-0.09610	-0.76680
O	0.40960	-0.15320	-2.49780
H	1.27030	-0.78370	0.67880

C	6.46690	2.94070	0.13140
C	3.31380	3.41360	1.86520
C	-0.50190	-0.36830	-3.45260
C	7.73350	2.34990	0.62750
C	8.10290	1.03420	0.33640
C	9.29650	0.52490	0.83020
C	10.12480	1.32240	1.61460
C	9.75860	2.63360	1.90750
C	8.56680	3.14620	1.41660
O	6.09470	4.06830	0.36880
C	0.09730	-0.98890	-4.66280
C	1.47690	-1.18090	-4.77720
C	2.00300	-1.74620	-5.93110
C	1.15880	-2.12550	-6.97100
C	-0.21650	-1.93640	-6.85840
C	-0.74620	-1.36760	-5.70890
O	-1.67470	-0.07750	-3.33370
C	2.80290	4.64550	2.51380
C	1.99380	5.56050	1.83510
C	1.54510	6.70280	2.48440
C	1.90020	6.93820	3.80950
C	2.70740	6.02870	4.48820
C	3.15790	4.88630	3.84320
O	4.02690	2.59810	2.40510
H	7.45810	0.41520	-0.27010
H	9.58040	-0.49470	0.60390
H	11.05470	0.92210	1.99870
H	10.40150	3.25370	2.51870
H	8.26960	4.16190	1.63850
H	2.12690	-0.87830	-3.96900
H	3.07220	-1.88930	-6.02030
H	1.57180	-2.56720	-7.86910
H	-0.87310	-2.23110	-7.66680
H	-1.81190	-1.21180	-5.61040
H	1.72100	5.37810	0.80600

H	0.91900	7.41030	1.95630
H	1.54910	7.83020	4.31290
H	2.98500	6.21180	5.51820
H	3.78670	4.17330	4.35820
C	3.66510	2.09470	-4.68860
H	3.62590	2.90720	-5.41090
H	4.45870	1.39350	-4.96700
H	2.70950	1.56820	-4.67600

Methyl β-D-Galf **2**, C1-exo, -60°/180°

	**X**	**Y**	**Z**
C	4.41040	3.21440	-0.63640
H	4.71340	4.25490	-0.56970
C	3.51230	2.79320	0.53340
H	4.07780	2.25410	1.28710
C	2.41580	1.92250	-0.11730
C	2.22490	0.54240	0.50090
H	1.79640	0.69130	1.49340
O	3.45360	-0.17650	0.58990
C	3.55620	2.90940	-1.86080
H	4.12910	2.63940	-2.75140
H	1.46940	2.46480	-0.06350
O	2.75570	4.03640	-2.09070
O	5.55660	2.35890	-0.72000
O	2.96690	3.98490	1.11820
O	2.80660	1.77440	-1.49110
C	1.29710	-0.32600	-0.33630
H	1.04810	-1.24260	0.19740
H	1.76950	-0.57980	-1.28370
O	0.10190	0.43540	-0.58110
H	3.78090	-0.12980	1.49490
C	6.57250	2.61480	0.12900
C	2.40590	3.86300	2.33490
C	-0.75920	-0.04250	-1.48990

C	7.67630	1.63350	-0.00040
C	7.57890	0.51810	-0.83670
C	8.63440	-0.38030	-0.91650
C	9.78880	-0.17020	-0.16760
C	9.88820	0.94050	0.66660
C	8.83490	1.83890	0.75240
O	6.56010	3.54580	0.90430
C	-1.87450	0.90130	-1.76060
C	-1.89110	2.19320	-1.22810
C	-2.94120	3.05160	-1.52530
C	-3.97930	2.62610	-2.34930
C	-3.96610	1.33880	-2.88020
C	-2.91620	0.47960	-2.58930
O	-0.62410	-1.12100	-2.02780
C	1.86480	5.14750	2.84090
C	1.91900	6.32130	2.08460
C	1.39470	7.49970	2.59810
C	0.81650	7.51370	3.86430
C	0.76080	6.34530	4.61990
C	1.28210	5.16520	4.11030
O	2.35640	2.80880	2.93270
H	6.68010	0.35400	-1.41300
H	8.55630	-1.24540	-1.56220
H	10.61040	-0.87240	-0.23290
H	10.78560	1.10380	1.24920
H	8.89790	2.70420	1.39780
H	-1.08340	2.52290	-0.59100
H	-2.94920	4.05330	-1.11530
H	-4.79740	3.29730	-2.57840
H	-4.77310	1.00710	-3.52070
H	-2.89240	-0.52100	-2.99860
H	2.36570	6.30790	1.10100
H	1.43670	8.40770	2.01060
H	0.40890	8.43450	4.26200
H	0.31100	6.35570	5.60430

H	1.24440	4.25070	4.68610
C	1.82830	3.86950	-3.16340
H	1.34090	4.83150	-3.30570
H	2.35010	3.58240	-4.08200
H	1.07990	3.11210	-2.92210

Methyl β-D-Galf **2**, C1-exo, 180°/+60°

	**X**	**Y**	**Z**
C	4.58510	2.22620	-1.10800
H	4.41460	3.27840	-1.32080
C	3.54290	1.70430	-0.11790
H	3.99940	0.97190	0.54710
C	2.50540	1.05030	-1.02960
C	1.66760	-0.04320	-0.39560
H	2.33210	-0.87390	-0.12920
O	0.72550	-0.46420	-1.37690
C	4.36040	1.38370	-2.36960
H	5.23180	0.78210	-2.64140
H	1.84140	1.81670	-1.44570
O	4.01660	2.25150	-3.41090
O	5.92150	2.03780	-0.63680
O	2.92990	2.74310	0.64640
O	3.31420	0.47330	-2.05810
C	0.95930	0.44310	0.85790
H	0.31580	1.29760	0.64790
H	1.66290	0.70680	1.64540
O	0.15210	-0.66420	1.30320
H	0.17420	-1.15250	-0.98250
C	6.52090	3.08120	-0.02150
C	3.44970	3.02090	1.86320
C	-0.61340	-0.45850	2.38830
C	7.86930	2.73210	0.48700
C	8.41380	1.45460	0.33190
C	9.67810	1.17540	0.83320

C	10.40310	2.16520	1.49070
C	9.86260	3.43890	1.64790
C	8.59990	3.72210	1.14830
O	6.00340	4.16920	0.09210
C	-1.42250	-1.64980	2.75030
C	-1.37280	-2.83260	2.00790
C	-2.15080	-3.91950	2.38340
C	-2.97980	-3.83310	3.49820
C	-3.03130	-2.65600	4.24030
C	-2.25560	-1.56770	3.86830
O	-0.62650	0.59250	2.99210
C	2.68760	4.07730	2.56810
C	1.50220	4.60540	2.04970
C	0.81700	5.58700	2.75250
C	1.31110	6.04820	3.96930
C	2.49300	5.52470	4.48740
C	3.17870	4.54070	3.79090
O	4.42130	2.45350	2.30890
H	7.84920	0.68640	-0.17590
H	10.09780	0.18510	0.71240
H	11.38840	1.94400	1.88140
H	10.42520	4.20850	2.16030
H	8.16770	4.70620	1.26560
H	-0.72850	-2.89970	1.14330
H	-2.11080	-4.83440	1.80640
H	-3.58550	-4.68240	3.78860
H	-3.67550	-2.58800	5.10740
H	-2.28670	-0.64790	4.43590
H	1.11940	4.24550	1.10610
H	-0.10300	5.99220	2.35140
H	0.77510	6.81510	4.51420
H	2.87770	5.88390	5.43310
H	4.09680	4.12490	4.18250
C	3.88040	1.59920	-4.67420
H	3.68940	2.37780	-5.40940

H	4.80260	1.06860	-4.93240
H	3.04780	0.89320	-4.66340

Methyl β-D-Galf **2**, C1-exo, 180°/-60°

	**X**	**Y**	**Z**
C	4.05190	2.13970	-1.01580
H	3.83990	3.16590	-1.31220
C	3.01220	1.66390	-0.01330
H	3.41780	0.86230	0.60320
C	1.87510	1.18390	-0.91690
C	1.04240	0.03910	-0.35580
H	1.68530	-0.84900	-0.32030
O	-0.03150	-0.15980	-1.27100
C	3.86120	1.21030	-2.20600
H	4.53570	0.34710	-2.16040
H	1.21700	2.02660	-1.14760
O	4.07770	1.91920	-3.38220
O	5.39340	2.03300	-0.53990
O	2.53940	2.72740	0.81420
O	2.52030	0.73110	-2.11680
C	0.54780	0.26890	1.06060
H	1.37670	0.32040	1.76490
H	-0.09990	-0.55690	1.36180
O	-0.20590	1.49300	1.10710
H	-0.47900	-0.98560	-1.04820
C	5.93240	3.11080	0.07120
C	3.08870	2.89200	2.03730
C	-0.33830	2.08030	2.30650
C	7.30870	2.85100	0.55580
C	7.89410	1.58450	0.48230
C	9.18120	1.38820	0.96440
C	9.88970	2.45140	1.51660
C	9.30940	3.71530	1.58930
C	8.02190	3.91480	1.11340

O	5.34340	4.16040	0.20050
C	-1.01530	3.39750	2.22660
C	-1.35740	3.97800	1.00300
C	-1.95230	5.23160	0.97480
C	-2.21240	5.90770	2.16350
C	-1.87680	5.32850	3.38370
C	-1.27820	4.07840	3.41580
O	0.07010	1.58210	3.33500
C	2.57000	4.10540	2.70860
C	1.93280	5.12150	1.99250
C	1.47470	6.25110	2.65410
C	1.63510	6.36540	4.03100
C	2.26400	5.35130	4.74860
C	2.73800	4.22680	4.08900
O	3.89410	2.12310	2.51200
H	7.34210	0.75990	0.05540
H	9.63160	0.40560	0.91010
H	10.89350	2.29530	1.89120
H	9.86030	4.54230	2.01810
H	7.55870	4.89050	1.16560
H	-1.15130	3.45190	0.08220
H	-2.21040	5.68350	0.02560
H	-2.67200	6.88780	2.13820
H	-2.07190	5.85700	4.30770
H	-0.99650	3.62500	4.35580
H	1.80240	5.02600	0.92510
H	0.97920	7.03530	2.09770
H	1.26440	7.24200	4.54700
H	2.38360	5.43810	5.82080
H	3.22760	3.43180	4.63470
C	4.14210	1.09640	-4.54860
H	4.41530	1.74710	-5.37630
H	4.90230	0.31840	-4.42730
H	3.17550	0.63110	-4.75350

Methyl β-D-Galf **2**, C1-exo, 180°/180°

	**X**	**Y**	**Z**
C	-1.07350	1.26800	-2.85160
H	-1.84160	0.79740	-3.46270
C	-1.14820	0.74530	-1.41590
H	-1.23540	1.55780	-0.70580
C	0.14330	-0.07480	-1.23700
C	1.19450	0.57170	-0.33130
H	1.56050	1.49940	-0.78400
O	2.24490	-0.38470	-0.23110
C	0.30930	0.85660	-3.34970
H	1.04010	1.66050	-3.18350
H	-0.08940	-1.05740	-0.82440
O	0.26330	0.51740	-4.68800
O	-1.17630	2.69270	-2.94640
O	-2.29030	-0.11490	-1.28330
O	0.65020	-0.28020	-2.55850
C	0.66420	0.88970	1.06700
H	1.49640	0.93550	1.77050
H	-0.04450	0.13720	1.41520
O	0.02220	2.17530	1.01480
H	3.01550	0.04210	0.16370
C	-2.40260	3.22660	-2.77460
C	-3.14170	0.12120	-0.26630
C	-0.72420	2.53550	2.07250
C	-2.39940	4.70520	-2.88090
C	-1.22790	5.42920	-3.11460
C	-1.27300	6.81530	-3.18060
C	-2.48340	7.48330	-3.01650
C	-3.65270	6.76340	-2.78540
C	-3.61130	5.37890	-2.71670
O	-3.38300	2.55260	-2.54860
C	-1.42220	3.82770	1.85710
C	-1.29220	4.55050	0.66860

C	-1.96780	5.75270	0.51090
C	-2.78000	6.23620	1.53200
C	-2.91590	5.51620	2.71630
C	-2.23800	4.31670	2.87940
O	-0.80010	1.86930	3.08210
C	-4.32060	-0.77900	-0.30120
C	-4.51100	-1.71310	-1.32270
C	-5.63570	-2.52720	-1.31540
C	-6.57320	-2.41450	-0.29270
C	-6.38580	-1.48470	0.72690
C	-5.26370	-0.66920	0.72320
O	-2.95490	0.97060	0.57710
H	-0.28850	4.91000	-3.23540
H	-0.36370	7.37510	-3.35640
H	-2.51460	8.56440	-3.06500
H	-4.59310	7.28250	-2.65380
H	-4.51000	4.80820	-2.52820
H	-0.66910	4.17160	-0.12810
H	-1.86520	6.30970	-0.40950
H	-3.30860	7.17250	1.40390
H	-3.54980	5.88990	3.51010
H	-2.33540	3.74730	3.79340
H	-3.78350	-1.79820	-2.11660
H	-5.78190	-3.24920	-2.10840
H	-7.44940	-3.05050	-0.29000
H	-7.11410	-1.39660	1.52270
H	-5.10630	0.05780	1.50800
C	1.54950	0.34460	-5.28920
H	1.37830	0.19160	-6.35230
H	2.16320	1.23810	-5.14040
H	2.06280	-0.52370	-4.87150

Methyl α-D-Galf **3**, gg

	X	Y	Z
O	2.06670	0.96990	0.34970
C	2.94720	0.69210	1.34300
O	2.61170	0.27730	2.42840
C	4.35110	0.91450	0.92440
C	4.67960	1.36010	-0.35820
C	6.01180	1.48290	-0.72720
C	7.01930	1.15720	0.17580
C	6.69470	0.71210	1.45480
C	5.36540	0.59290	1.82950
H	3.89650	1.59150	-1.06470
H	6.26450	1.82400	-1.72280
H	8.05760	1.24490	-0.11840
H	7.47800	0.45150	2.15460
H	5.09880	0.23440	2.81380
O	1.69260	-1.71620	-0.15540
O	-1.64020	-0.38830	-0.87260
O	-2.78330	1.98550	-1.72120
C	-0.69450	-1.38960	-0.53620
C	0.69630	-0.76440	-0.54320
C	0.75030	0.40030	0.43650
C	-0.29160	1.44750	0.05500
C	-1.64860	0.73870	0.01250
C	-2.83910	1.60050	-0.33920
H	0.90430	-0.41440	-1.55030
H	0.57640	0.05530	1.45260
H	-1.83540	0.39220	1.03530
C	2.83110	-1.76890	-0.88870
C	-3.75150	2.79930	-2.15790
O	0.00980	2.04700	-1.19570
H	-3.75770	1.03850	-0.16880
H	-2.85170	2.48940	0.29240
H	-0.78810	-2.14310	-1.32350
O	-0.92790	-1.95370	0.71870
H	-0.33500	2.20760	0.84340

H	0.89370	2.43220	-1.13200
O	2.92550	-1.31100	-2.00460
C	3.94340	-2.40810	-0.14620
C	3.78210	-2.89460	1.15330
C	4.87190	-3.41650	1.83620
C	6.12470	-3.44940	1.23100
C	6.28820	-2.96490	-0.06430
C	5.20130	-2.44810	-0.75230
H	2.81330	-2.84930	1.62790
H	4.74610	-3.78940	2.84440
H	6.97490	-3.84790	1.77010
H	7.26370	-2.98370	-0.53250
H	5.31690	-2.05510	-1.75260
C	-3.60100	3.13740	-3.59730
O	-4.64570	3.20980	-1.44620
C	-4.56420	3.95550	-4.19130
C	-4.45780	4.29620	-5.53220
C	-3.38790	3.82270	-6.28740
C	-2.42540	3.00730	-5.69900
C	-2.52910	2.66300	-4.35770
H	-5.38970	4.31690	-3.59350
H	-5.20690	4.92990	-5.98910
H	-3.30460	4.08920	-7.33360
H	-1.59340	2.63960	-6.28560
H	-1.78290	2.03130	-3.89810
C	-2.11630	-2.74250	0.78380
H	-2.14250	-3.18640	1.77640
H	-3.00590	-2.12710	0.63410
H	-2.09200	-3.53320	0.02760

Methyl α-D-Galf **3**, gt

	**X**	**Y**	**Z**
O	2.07540	0.96390	0.34730
C	2.91410	0.67510	1.37310

O	2.53070	0.28820	2.45300
C	4.33730	0.84700	0.99770
C	4.72080	1.26020	-0.28050
C	6.06740	1.33500	-0.60770
C	7.03450	0.99350	0.33300
C	6.65500	0.58080	1.60770
C	5.31120	0.50960	1.94060
H	3.96850	1.50400	-1.01570
H	6.36280	1.65090	-1.59980
H	8.08400	1.04350	0.07150
H	7.40670	0.30790	2.33680
H	5.00210	0.17640	2.92130
O	1.63990	-1.72170	-0.12190
O	-1.61250	-0.29960	-0.99710
O	-3.95980	1.14500	-0.43540
C	-0.72040	-1.32760	-0.60480
C	0.68910	-0.74740	-0.56440
C	0.74190	0.43110	0.39850
C	-0.26030	1.49970	-0.02160
C	-1.63390	0.83620	-0.12090
C	-2.68570	1.78330	-0.64230
H	0.94740	-0.42110	-1.56800
H	0.52550	0.10510	1.41280
H	-1.91670	0.51310	0.88520
C	2.80120	-1.82620	-0.81310
C	-5.04890	1.81920	-0.82910
O	0.06840	2.07640	-1.27830
H	-2.65600	2.72490	-0.09370
H	-2.54660	1.98320	-1.70300
H	-0.80650	-2.09060	-1.38400
O	-1.02620	-1.86290	0.64760
H	-0.30360	2.27110	0.75470
H	0.93590	2.49410	-1.20200
O	2.94870	-1.39580	-1.93410
C	3.86630	-2.48340	-0.01900

C	3.64550	-2.93540	1.28420
C	4.69430	-3.47540	2.01550
C	5.96520	-3.56090	1.45500
C	6.18800	-3.11110	0.15610
C	5.14210	-2.57620	-0.58000
H	2.66300	-2.84950	1.72380
H	4.52240	-3.82130	3.02650
H	6.78350	-3.97330	2.03180
H	7.17780	-3.17080	-0.27750
H	5.30440	-2.20930	-1.58380
C	-6.30330	1.05640	-0.59730
O	-4.99970	2.92860	-1.31820
C	-7.51750	1.66080	-0.92980
C	-8.71110	0.98110	-0.73340
C	-8.69940	-0.30710	-0.20500
C	-7.49140	-0.91370	0.12710
C	-6.29500	-0.23630	-0.06690
H	-7.51300	2.66090	-1.34100
H	-9.64990	1.45370	-0.99190
H	-9.63090	-0.83760	-0.05240
H	-7.48170	-1.91560	0.53690
H	-5.35680	-0.70670	0.18950
C	-2.25500	-2.58980	0.67690
H	-2.33890	-3.02120	1.67180
H	-3.10590	-1.93160	0.48990
H	-2.24450	-3.38860	-0.07110

Methyl α-D-Galf **3**, tg

	**X**	**Y**	**Z**
O	2.03620	1.01110	0.27620
C	2.90340	0.80900	1.29830
O	2.55450	0.47450	2.40710
C	4.31270	1.00300	0.88270
C	4.65660	1.35700	-0.42410

C	5.99320	1.45460	-0.78490
C	6.98970	1.19410	0.15090
C	6.64970	0.84050	1.45420
C	5.31590	0.74730	1.82040
H	3.88210	1.53740	-1.15450
H	6.25800	1.72460	-1.79900
H	8.03150	1.26120	-0.13630
H	7.42450	0.63030	2.17990
H	5.03750	0.45910	2.82440
O	1.68180	-1.71040	-0.02630
O	-1.63860	-0.45380	-0.90730
O	-2.90890	2.74530	0.11150
C	-0.69880	-1.42320	-0.47200
C	0.69010	-0.79520	-0.50360
C	0.72140	0.44100	0.38460
C	-0.32080	1.44730	-0.08640
C	-1.67630	0.73800	-0.11310
C	-2.78270	1.57870	-0.71820
H	0.91500	-0.52260	-1.53080
H	0.53250	0.17210	1.42100
H	-1.93810	0.48610	0.91860
C	2.83120	-1.81910	-0.73630
C	-3.79120	3.67700	-0.28140
O	-0.03050	1.94250	-1.38640
H	-2.53880	1.87380	-1.73660
H	-3.71930	1.02200	-0.71760
H	-0.77720	-2.23750	-1.19820
O	-0.95530	-1.88400	0.81910
H	-0.37420	2.26920	0.63350
H	0.81690	2.40380	-1.35160
O	2.94120	-1.45250	-1.88410
C	3.93350	-2.39280	0.07170
C	3.75270	-2.77760	1.40240
C	4.83300	-3.23910	2.14150
C	6.09590	-3.31220	1.56130

C	6.27890	-2.92920	0.23500
C	5.20150	-2.47350	-0.50880
H	2.77590	-2.70090	1.85620
H	4.69190	-3.53300	3.17350
H	6.93870	-3.66270	2.14370
H	7.26220	-2.97900	-0.21430
H	5.33240	-2.15890	-1.53460
C	-3.81970	4.85210	0.62670
O	-4.48020	3.55570	-1.27180
C	-4.71350	5.88670	0.34200
C	-4.77010	7.00390	1.16270
C	-3.93430	7.09490	2.27270
C	-3.04170	6.06620	2.56010
C	-2.98240	4.94630	1.74150
H	-5.35660	5.80390	-0.52330
H	-5.46450	7.80330	0.93850
H	-3.97860	7.96690	2.91300
H	-2.39140	6.13690	3.42240
H	-2.28960	4.14760	1.96360
C	-2.14430	-2.66730	0.92700
H	-2.18470	-3.03380	1.95020
H	-3.03170	-2.06630	0.71780
H	-2.10860	-3.51350	0.23400

Methyl α-D-Galf **4**, C1-endo, +60°/+60°

	**X**	**Y**	**Z**
C	2.45700	2.97010	-4.00570
O	3.19930	4.19570	-4.07380
C	0.99880	3.41740	-3.96080
C	1.07450	4.68370	-3.10000
C	2.53210	5.16820	-3.27050
O	2.73600	2.17230	-5.09000
O	0.87770	4.33360	-1.72320
H	0.33040	2.67200	-3.53970

O	0.60610	3.75170	-5.29300
C	-0.71190	3.72370	-5.58160
C	-0.28460	4.68890	-1.13190
H	0.33290	5.41930	-3.39150
C	2.70040	6.54130	-3.91050
C	2.07690	6.66170	-5.28760
H	3.77670	6.72510	-4.00890
O	2.12480	7.46800	-2.99560
O	2.41100	7.99140	-5.74040
H	2.48960	5.92580	-5.97540
H	0.99320	6.55970	-5.25790
H	2.19220	8.35140	-3.38000
H	2.98640	5.21350	-2.27670
C	1.87460	8.39720	-6.90220
C	2.26250	9.78710	-7.25570
C	3.09260	10.55620	-6.43610
C	3.42600	11.85190	-6.80770
C	2.93480	12.38600	-7.99560
C	2.10750	11.62200	-8.81470
C	1.77220	10.32710	-8.44670
H	3.47390	10.14120	-5.51440
H	4.06900	12.44540	-6.17080
H	3.19640	13.39660	-8.28290
H	1.72510	12.03650	-9.73850
H	1.13040	9.72360	-9.07380
C	-0.34640	4.24880	0.28400
C	-1.48990	4.56380	1.02190
C	-1.59150	4.17320	2.34900
C	-0.55240	3.46520	2.94730
C	0.58850	3.14840	2.21540
C	0.69450	3.53790	0.88700
H	-2.28960	5.11400	0.54550
H	-2.47910	4.41950	2.91730
H	-0.63190	3.16020	3.98320
H	1.39610	2.59780	2.68030

H	1.57970	3.29290	0.31840
C	-0.97930	4.06130	-7.00000
C	0.05340	4.28500	-7.91360
C	-2.30940	4.14860	-7.41860
C	-2.60360	4.45850	-8.73800
C	-1.57180	4.67930	-9.64720
C	-0.24590	4.59140	-9.23390
H	1.08160	4.22090	-7.58990
H	-3.10030	3.97400	-6.70210
H	-3.63490	4.52850	-9.05890
H	-1.80190	4.92120	-10.67720
H	0.55570	4.76670	-9.93960
O	1.15500	7.69260	-7.57660
O	-1.16060	5.29960	-1.70030
O	-1.56060	3.44960	-4.76280
H	2.70610	2.45630	-3.06550
C	4.01900	1.54130	-5.03400
H	4.07080	0.86820	-5.88660
H	4.81970	2.28030	-5.10080
H	4.12460	0.96970	-4.10700

Methyl α-D-Galf **4**, C1-endo, +60°/-60°

	**X**	**Y**	**Z**
C	0.03360	3.19530	-1.47760
O	1.36090	3.69440	-1.68450
C	-0.82050	3.99530	-2.45650
C	-0.15050	5.37470	-2.43850
C	1.26090	5.10430	-1.86200
H	-0.27780	3.43820	-0.45020
O	-0.86110	6.24440	-1.54730
H	-1.86750	4.04510	-2.17110
O	-0.68140	3.39670	-3.74720
C	-1.62660	3.68430	-4.66420
C	-1.42220	7.36080	-2.05950

H	-0.12850	5.81540	-3.42420
C	2.45180	5.57510	-2.69240
C	2.59970	4.95640	-4.07240
H	3.34830	5.26330	-2.14160
O	2.35620	6.99330	-2.72920
O	1.55100	5.42910	-4.93590
H	3.56220	5.24210	-4.49910
H	2.55720	3.86920	-4.00450
H	3.12430	7.34880	-3.19330
H	1.32070	5.61170	-0.89350
C	1.63430	5.09370	-6.23260
C	0.51780	5.64890	-7.03720
C	-0.44220	6.49990	-6.48410
C	-1.48020	6.97610	-7.27240
C	-1.56430	6.61130	-8.61280
C	-0.60530	5.76860	-9.16730
C	0.43210	5.28850	-8.38290
H	-0.38490	6.78690	-5.44480
H	-2.22610	7.62920	-6.83810
H	-2.37840	6.98040	-9.22390
H	-0.67410	5.47730	-10.20730
H	1.17460	4.62120	-8.79730
C	-2.12860	8.15560	-1.02430
C	-2.75500	9.34080	-1.41730
C	-3.42700	10.11590	-0.48360
C	-3.47780	9.71250	0.84830
C	-2.85530	8.53220	1.24410
C	-2.18160	7.75340	0.31280
H	-2.70820	9.64290	-2.45440
H	-3.91090	11.03340	-0.79230
H	-4.00240	10.31770	1.57690
H	-2.89470	8.21860	2.27930
H	-1.69800	6.83740	0.61920
C	-1.36370	3.02360	-5.96410
C	-0.23780	2.22250	-6.16910

C	-2.26660	3.23440	-7.00750
C	-2.04680	2.64880	-8.24420
C	-0.92300	1.85300	-8.44750
C	-0.01970	1.64180	-7.41030
H	0.46500	2.06620	-5.36410
H	-3.12680	3.86720	-6.84060
H	-2.74330	2.82210	-9.05410
H	-0.74760	1.40300	-9.41660
H	0.85790	1.02920	-7.57030
O	2.53050	4.40830	-6.67890
O	-1.34850	7.66980	-3.22780
O	-2.57230	4.40520	-4.43200
O	-0.01880	1.84060	-1.70060
C	0.56330	1.05640	-0.65490
H	0.36920	0.01550	-0.90310
H	1.64030	1.22450	-0.59490
H	0.10110	1.29780	0.30700

Methyl α-D-Galf **4**, C1-endo, +60°/180°

	**X**	**Y**	**Z**
C	2.97840	5.69810	-6.09660
O	1.78430	6.27490	-5.56000
C	2.89100	4.22050	-5.72230
C	2.22190	4.26970	-4.34450
C	1.48090	5.62590	-4.32780
H	3.84840	6.14560	-5.59240
O	3.04690	5.89780	-7.45650
O	3.24610	4.28130	-3.34040
H	3.85920	3.72840	-5.68570
O	2.04220	3.57790	-6.67660
C	2.04850	2.22960	-6.69280
C	3.23980	3.30920	-2.40190
H	1.57280	3.41850	-4.17540
C	-0.03360	5.55160	-4.13860

C	-0.80540	4.64460	-5.09050
H	-0.42230	6.57020	-4.24510
O	-0.21100	5.07580	-2.80710
O	-0.78580	5.23280	-6.39800
H	-0.38650	3.63920	-5.13800
H	-1.83710	4.56480	-4.74350
H	-1.14310	5.15170	-2.56740
H	1.87660	6.20910	-3.49070
C	-1.51410	4.63420	-7.34930
C	-1.31210	5.25830	-8.68110
C	-0.28220	6.17470	-8.90600
C	-0.08690	6.69680	-10.17740
C	-0.92230	6.31660	-11.22420
C	-1.95370	5.40790	-10.99990
C	-2.14530	4.87580	-9.73300
H	0.37320	6.45260	-8.09400
H	0.71930	7.39760	-10.35240
H	-0.76830	6.72530	-12.21490
H	-2.60240	5.11090	-11.81380
H	-2.93170	4.15690	-9.54880
C	4.36900	3.44960	-1.44890
C	4.47860	2.51680	-0.41490
C	5.51890	2.60830	0.49810
C	6.45690	3.63110	0.38390
C	6.35170	4.56260	-0.64500
C	5.31160	4.47500	-1.56050
H	3.74450	1.72670	-0.33770
H	5.59990	1.88360	1.29790
H	7.26910	3.70210	1.09630
H	7.08070	5.35760	-0.73410
H	5.22930	5.19760	-2.35920
C	1.18290	1.68380	-7.76460
C	0.74290	2.47830	-8.82500
C	0.81480	0.33790	-7.70270
C	0.00210	-0.20410	-8.68740

C	-0.43490	0.59080	-9.74480
C	-0.06050	1.92900	-9.81420
H	1.03130	3.51750	-8.87380
H	1.16230	-0.26820	-6.87710
H	-0.29210	-1.24420	-8.63220
H	-1.06810	0.16620	-10.51360
H	-0.40090	2.54860	-10.63300
O	-2.24120	3.68690	-7.13390
O	2.40480	2.43500	-2.35260
O	2.68860	1.56340	-5.90940
C	3.33360	7.24640	-7.83430
H	3.47070	7.24350	-8.91310
H	2.50630	7.90900	-7.57290
H	4.24910	7.59510	-7.34700

Methyl α-D-Galf **4**, C1-endo, -60°/+60°

	**X**	**Y**	**Z**
C	1.96950	2.49260	-3.09090
O	2.57070	2.97760	-4.29540
C	0.95920	3.57320	-2.71590
C	1.67990	4.85730	-3.12980
C	2.72670	4.39310	-4.17390
H	2.73930	2.43450	-2.30640
O	1.38820	1.26360	-3.29670
O	2.30930	5.37820	-1.94750
H	0.70320	3.57220	-1.65990
O	-0.20800	3.37240	-3.51370
C	-1.31320	4.05840	-3.16890
C	2.77470	6.64030	-2.01340
H	0.99560	5.59610	-3.53300
C	2.58490	5.05470	-5.53370
C	3.55960	4.45820	-6.52970
H	2.82370	6.11390	-5.39380
O	1.24030	4.91190	-5.98050

O	3.42150	5.23360	-7.73830
H	4.58700	4.52900	-6.16950
H	3.31940	3.41740	-6.73950
H	1.14340	5.41460	-6.79880
H	3.72530	4.62070	-3.78610
C	4.12970	4.83300	-8.80490
C	3.89860	5.68750	-9.99830
C	3.02840	6.78040	-9.96950
C	2.84540	7.55090	-11.11000
C	3.52720	7.23660	-12.28220
C	4.39460	6.14780	-12.31430
C	4.58020	5.37560	-11.17670
H	2.50040	7.02440	-9.05910
H	2.17090	8.39710	-11.08480
H	3.38260	7.83940	-13.16990
H	4.92500	5.90240	-13.22530
H	5.25110	4.52760	-11.18820
C	3.37770	7.09270	-0.73650
C	3.92510	8.37690	-0.68370
C	4.49810	8.84120	0.49100
C	4.52750	8.02650	1.62000
C	3.98320	6.74620	1.57160
C	3.40930	6.27710	0.39770
H	3.89550	8.99960	-1.56720
H	4.92140	9.83660	0.52810
H	4.97440	8.38930	2.53700
H	4.00580	6.11340	2.44930
H	2.98570	5.28410	0.35930
C	-2.44450	3.79720	-4.09160
C	-2.30750	2.97120	-5.21040
C	-3.67210	4.40710	-3.82370
C	-4.75380	4.19150	-4.66500
C	-4.61490	3.36800	-5.77930
C	-3.39230	2.76020	-6.05070
H	-1.35670	2.50340	-5.42000

H	-3.76530	5.04520	-2.95560
H	-5.70420	4.66450	-4.45400
H	-5.45910	3.20060	-6.43630
H	-3.28420	2.12160	-6.91790
O	4.87500	3.87650	-8.77970
O	2.69820	7.31370	-3.01850
O	-1.35700	4.80090	-2.21170
C	2.32640	0.18780	-3.39560
H	1.74310	-0.72820	-3.45370
H	2.94180	0.28810	-4.29170
H	2.96980	0.15930	-2.51110

Methyl α-D-Galf **4**, C1-endo, -60°/-60°

	**X**	**Y**	**Z**
C	-1.63460	2.67100	-2.56010
O	-1.07550	2.28500	-3.81600
C	-1.17190	4.11250	-2.37140
C	0.23690	4.08040	-2.96550
C	0.22870	2.85610	-3.91920
H	-1.19720	2.04890	-1.76390
O	-3.00490	2.54400	-2.58050
O	1.14240	3.88650	-1.86590
H	-1.16980	4.43250	-1.33290
O	-2.02810	4.94960	-3.15030
C	-1.94230	6.27650	-2.94540
C	2.44710	4.11880	-2.10170
H	0.48330	5.00300	-3.48010
C	0.58030	3.21060	-5.35820
C	0.64750	2.02200	-6.30280
H	1.59940	3.61030	-5.31110
O	-0.32180	4.21250	-5.81670
O	-0.66550	1.46380	-6.45860
H	1.01360	2.34600	-7.27910
H	1.32790	1.26250	-5.91380

H	0.04840	4.61800	-6.61040
H	0.97390	2.13770	-3.56020
C	-0.78500	0.34320	-7.17560
C	-2.18440	-0.15900	-7.19350
C	-3.19600	0.46920	-6.46190
C	-4.48820	-0.03790	-6.49050
C	-4.77840	-1.16800	-7.24990
C	-3.77240	-1.79480	-7.98110
C	-2.47870	-1.29330	-7.95220
H	-2.96470	1.34290	-5.87040
H	-5.26860	0.44770	-5.91890
H	-5.78710	-1.56100	-7.27120
H	-3.99740	-2.67380	-8.57130
H	-1.68780	-1.77270	-8.51270
C	3.28730	3.92200	-0.89550
C	4.66450	4.12570	-1.01020
C	5.48760	3.95320	0.09300
C	4.94060	3.57590	1.31680
C	3.56870	3.37180	1.43520
C	2.74120	3.54380	0.33390
H	5.07640	4.41830	-1.96620
H	6.55410	4.11230	0.00050
H	5.58320	3.44140	2.17760
H	3.14370	3.07930	2.38670
H	1.67620	3.38690	0.42490
C	-2.84980	7.04080	-3.83580
C	-3.64790	6.40890	-4.79330
C	-2.89030	8.43070	-3.70550
C	-3.72200	9.18220	-4.52280
C	-4.51630	8.55010	-5.47620
C	-4.47740	7.16500	-5.61020
H	-3.61450	5.33420	-4.89660
H	-2.26740	8.90940	-2.96240
H	-3.75160	10.25910	-4.41850
H	-5.16460	9.13680	-6.11460

H	-5.09370	6.67390	-6.35220
O	0.14800	-0.19610	-7.73590
O	2.86740	4.45160	-3.18910
O	-1.20000	6.77090	-2.12420
C	-3.46030	1.18920	-2.51360
H	-4.54510	1.22960	-2.44730
H	-3.16880	0.63520	-3.40740
H	-3.05520	0.69310	-1.62650

Methyl α-D-Galf **4**, C1-endo, -60°/180°

	**X**	**Y**	**Z**
C	3.85450	5.14970	-3.16380
O	2.77820	5.22170	-4.10170
C	3.16480	5.05060	-1.80510
C	1.94000	5.94830	-1.99140
C	1.74650	6.02650	-3.52590
H	4.42680	6.08920	-3.20500
O	4.66560	4.07160	-3.43120
O	2.28170	7.23190	-1.43970
H	3.79140	5.38240	-0.98170
O	2.76820	3.69200	-1.61290
C	2.39360	3.33060	-0.37190
C	1.27890	8.11810	-1.29590
H	1.06620	5.55500	-1.48200
C	0.38620	5.55410	-4.01020
C	0.27300	5.58600	-5.52810
H	-0.35290	6.23370	-3.57820
O	0.17060	4.22210	-3.55520
O	0.43960	6.95740	-5.93350
H	1.04080	4.96710	-5.98990
H	-0.70930	5.23100	-5.84190
H	-0.77710	4.04210	-3.56950
H	1.87810	7.06700	-3.83650
C	0.44480	7.21490	-7.24900

C	0.66000	8.65540	-7.54310
C	0.85990	9.59450	-6.52770
C	1.06160	10.93020	-6.84900
C	1.06390	11.33560	-8.18070
C	0.86440	10.40220	-9.19470
C	0.66360	9.06650	-8.87760
H	0.85750	9.27890	-5.49440
H	1.21690	11.65530	-6.06050
H	1.22100	12.37790	-8.42830
H	0.86590	10.71660	-10.23040
H	0.50850	8.33160	-9.65560
C	1.73630	9.41000	-0.72930
C	0.79380	10.42740	-0.56250
C	1.17840	11.65300	-0.03890
C	2.50580	11.86940	0.32210
C	3.44810	10.85810	0.15760
C	3.06780	9.63020	-0.36700
H	-0.23340	10.24640	-0.84740
H	0.44560	12.43930	0.08830
H	2.80570	12.82590	0.73120
H	4.47960	11.02640	0.43860
H	3.79790	8.84440	-0.49490
C	1.98380	1.90660	-0.30300
C	1.95850	1.09100	-1.43750
C	1.61120	1.38350	0.93720
C	1.21920	0.05710	1.04340
C	1.19470	-0.75360	-0.08870
C	1.56360	-0.23530	-1.32690
H	2.24300	1.49640	-2.39750
H	1.63350	2.02400	1.80820
H	0.93240	-0.34540	2.00630
H	0.88780	-1.78860	-0.00580
H	1.54330	-0.86530	-2.20670
O	0.29030	6.35600	-8.09150
O	0.13410	7.86740	-1.60680

O	2.39600	4.09650	0.56790
C	5.50300	4.24680	-4.57780
H	6.16830	3.38710	-4.61060
H	4.90860	4.28590	-5.49260
H	6.09190	5.16440	-4.48480

Methyl α-D-Galf **4**, C1-endo, 180°/+60°

	**X**	**Y**	**Z**
C	3.35990	4.47960	-4.68260
O	3.73470	5.37820	-3.65570
C	2.05000	3.89670	-4.16310
C	1.37320	5.13750	-3.58680
C	2.55760	5.95850	-3.06040
H	4.17020	3.75250	-4.77960
O	3.11870	5.11950	-5.90130
O	0.48940	4.85000	-2.49900
H	2.26110	3.18540	-3.36670
O	1.30740	3.26730	-5.19970
C	0.47190	2.26460	-4.85450
C	-0.81660	4.65420	-2.78350
H	0.82380	5.65130	-4.37270
C	2.46570	7.44360	-3.38000
C	1.21540	8.05370	-2.77010
H	2.43120	7.56690	-4.46860
O	3.63530	8.05910	-2.85200
O	1.23880	9.44850	-3.13180
H	0.30440	7.60210	-3.16130
H	1.22430	7.96170	-1.68350
H	3.58440	9.00530	-3.03890
H	2.64440	5.83090	-1.97940
C	0.24230	10.21550	-2.65840
C	0.36170	11.63380	-3.08200
C	1.41070	12.08060	-3.88970
C	1.47890	13.41690	-4.26050

C	0.50400	14.31210	-3.82930
C	-0.54260	13.86980	-3.02420
C	-0.61430	12.53540	-2.65170
H	2.16630	11.38520	-4.22530
H	2.29240	13.76050	-4.88630
H	0.55970	15.35350	-4.12030
H	-1.30110	14.56520	-2.68860
H	-1.42190	12.17880	-2.02730
C	-1.61420	4.34650	-1.57320
C	-2.98020	4.10060	-1.73080
C	-3.76580	3.80530	-0.62650
C	-3.19220	3.75410	0.64140
C	-1.83120	3.99790	0.80260
C	-1.04100	4.29300	-0.30000
H	-3.41230	4.14270	-2.72100
H	-4.82370	3.61470	-0.75240
H	-3.80560	3.52350	1.50330
H	-1.38560	3.95770	1.78810
H	0.01550	4.48090	-0.17650
C	-0.27420	1.73390	-6.01990
C	-0.08100	2.23050	-7.31170
C	-1.20040	0.71180	-5.79980
C	-1.92550	0.18980	-6.86080
C	-1.72960	0.68510	-8.14750
C	-0.80790	1.70420	-8.37080
H	0.63310	3.02300	-7.48210
H	-1.34380	0.33800	-4.79540
H	-2.64340	-0.60120	-6.68660
H	-2.29600	0.27780	-8.97550
H	-0.65670	2.08960	-9.37090
O	-0.64970	9.77760	-1.96410
O	-1.26690	4.72780	-3.90490
O	0.35460	1.86020	-3.71910
C	4.29050	5.67690	-6.49570
H	3.99350	6.06740	-7.46650

H	4.69320	6.48600	-5.88290
H	5.05700	4.90680	-6.62860

Methyl α-D-Galf **4**, C1-endo, 180°/-60°

	**X**	**Y**	**Z**
C	3.76130	6.09750	-2.37820
O	3.18880	7.26780	-2.97200
C	2.80770	4.96050	-2.76590
C	1.46270	5.67740	-2.96550
C	1.78680	7.17020	-2.74300
H	3.73900	6.21970	-1.28430
O	5.04470	5.89890	-2.82460
O	0.49860	5.24500	-2.00390
H	2.73950	4.19820	-1.99450
O	3.26420	4.37450	-3.98810
C	2.78800	3.15000	-4.28840
C	-0.65160	4.68680	-2.44140
H	1.09050	5.48490	-3.96810
C	1.06550	8.14290	-3.66150
C	-0.44260	7.96220	-3.66900
H	1.41180	7.96010	-4.68620
O	1.43840	9.45030	-3.23500
O	-0.91660	8.01520	-2.31060
H	-0.90780	8.76390	-4.24520
H	-0.72660	7.01120	-4.11800
H	1.10250	10.09000	-3.87520
H	1.55830	7.42180	-1.70150
C	-2.20860	7.71800	-2.10070
C	-2.56740	7.73710	-0.66100
C	-1.65120	8.11780	0.32260
C	-2.02500	8.10400	1.65880
C	-3.30910	7.70630	2.02020
C	-4.22220	7.32380	1.04220
C	-3.85400	7.34090	-0.29470

H	-0.65300	8.41830	0.03960
H	-1.31370	8.39870	2.41960
H	-3.59600	7.68950	3.06410
H	-5.21730	7.00460	1.32330
H	-4.55000	7.03610	-1.06360
C	-1.57440	4.36870	-1.32720
C	-2.82890	3.84170	-1.64160
C	-3.73620	3.55830	-0.63220
C	-3.39610	3.79990	0.69590
C	-2.14750	4.32460	1.01320
C	-1.23730	4.61100	0.00690
H	-3.08270	3.66730	-2.67790
H	-4.70990	3.15570	-0.87940
H	-4.10830	3.58750	1.48320
H	-1.88890	4.52460	2.04440
H	-0.27360	5.03250	0.25040
C	3.33870	2.62770	-5.56260
C	4.28250	3.34030	-6.30710
C	2.89130	1.38460	-6.01570
C	3.38190	0.85890	-7.20190
C	4.32270	1.57080	-7.94180
C	4.77160	2.80970	-7.49310
H	4.63030	4.30110	-5.95640
H	2.16030	0.84230	-5.43190
H	3.03220	-0.10420	-7.55070
H	4.70580	1.16000	-8.86740
H	5.50340	3.36250	-8.06790
O	-2.97770	7.45060	-2.99960
O	-0.89710	4.50250	-3.61260
O	1.99830	2.56010	-3.58360
C	5.99980	6.80380	-2.26140
H	6.97670	6.48280	-2.61540
H	5.80800	7.82700	-2.58970
H	5.97080	6.75880	-1.16860

Methyl α-D-Galf **4**, C1-endo, 180°/180°

	X	Y	Z
C	2.17080	4.65790	-7.26180
O	1.91580	5.61790	-6.23010
C	0.78660	4.34970	-7.86150
C	-0.18770	4.74760	-6.74630
C	0.71870	5.19950	-5.58690
H	2.57990	3.74880	-6.79710
O	3.04030	5.16860	-8.19500
O	-1.01700	3.67750	-6.28390
H	0.69560	3.30380	-8.13740
O	0.53080	5.17570	-8.99770
C	0.73280	4.64670	-10.21790
C	-2.02060	3.29520	-7.10120
H	-0.81910	5.55970	-7.10070
C	0.18600	6.33940	-4.73440
C	-1.08100	5.97380	-3.96690
H	-0.00670	7.20270	-5.38090
O	1.20390	6.63660	-3.78250
O	-2.19370	5.94240	-4.87640
H	-0.97580	5.00420	-3.48060
H	-1.26850	6.72840	-3.20140
H	0.97800	7.46280	-3.33700
H	0.91940	4.33610	-4.93830
C	-3.33340	5.40440	-4.40910
C	-4.38620	5.30230	-5.44840
C	-4.21960	5.84000	-6.72600
C	-5.21520	5.67970	-7.67960
C	-6.37650	4.98110	-7.36480
C	-6.54650	4.44690	-6.09020
C	-5.55700	4.60920	-5.13380
H	-3.31150	6.36950	-6.97430
H	-5.08050	6.09150	-8.67140
H	-7.14720	4.84870	-8.11360

H	-7.44560	3.89540	-5.84790
H	-5.66900	4.18630	-4.14550
C	-2.97310	2.37310	-6.44120
C	-4.05930	1.90700	-7.18410
C	-5.00750	1.08900	-6.58970
C	-4.87770	0.73370	-5.24990
C	-3.79470	1.19440	-4.50660
C	-2.84220	2.01150	-5.09830
H	-4.15650	2.20500	-8.21860
H	-5.85300	0.73630	-7.16580
H	-5.62270	0.10120	-4.78380
H	-3.69700	0.92280	-3.46350
H	-2.00930	2.38220	-4.51920
C	0.43080	5.61520	-11.30210
C	0.02960	6.92620	-11.03320
C	0.55840	5.18680	-12.62520
C	0.28580	6.05900	-13.66910
C	-0.11420	7.36490	-13.39800
C	-0.24110	7.79640	-12.08070
H	-0.06700	7.26000	-10.01050
H	0.87000	4.17020	-12.82190
H	0.38490	5.72250	-14.69310
H	-0.32640	8.04570	-14.21280
H	-0.55130	8.81170	-11.86970
O	-3.46020	5.02810	-3.26320
O	-2.12030	3.69410	-8.24010
O	1.10600	3.50860	-10.39970
C	4.40490	5.19920	-7.76710
H	4.99010	5.53220	-8.62100
H	4.53780	5.89570	-6.93700
H	4.73250	4.20070	-7.46310

Methyl α-D-Galf **4**, C2-exo, +60°/+60°

	X	Y	Z
H	-2.17870	2.76550	1.76960
O	-2.74680	6.02190	-2.18580
O	-0.58770	2.06150	-1.09840
O	-4.02920	3.00380	-0.69310
O	-1.30510	4.31310	0.70700
O	0.05640	6.45790	-2.56040
C	-1.74890	2.23200	-0.28310
C	-2.64380	3.26660	-0.96590
C	-2.28580	4.60390	-0.28590
C	-1.75120	5.69200	-1.22240
C	-1.37570	2.91840	1.03380
C	-0.50400	5.27180	-1.96390
H	-2.24400	1.27850	-0.13070
H	-2.48680	3.26920	-2.03970
H	-1.50580	6.56410	-0.60760
O	-0.16800	2.47770	1.52050
H	-3.19360	4.99420	0.18620
H	-0.73150	4.55290	-2.75060
H	0.21840	4.83520	-1.27730
C	0.00440	0.84690	-1.10300
C	-4.64300	2.08660	-1.47070
C	1.18150	6.29750	-3.26980
C	1.68080	7.57200	-3.84780
C	1.00080	8.78010	-3.67310
C	1.50530	9.94660	-4.23220
C	2.68740	9.91490	-4.96650
C	3.36710	8.71250	-5.14290
C	2.86570	7.54470	-4.58640
H	0.08240	8.80270	-3.10460
H	0.97620	10.88100	-4.09560
H	3.07860	10.82620	-5.40110
H	4.28630	8.68670	-5.71390
H	3.38390	6.60460	-4.71660
C	1.22400	0.82300	-1.94670

C	1.69630	1.96340	-2.60080
C	1.91130	-0.38670	-2.07620
C	3.05790	-0.45570	-2.85350
C	3.52650	0.68300	-3.50490
C	2.84520	1.88980	-3.37690
H	-3.46590	6.49140	-1.74470
H	1.17740	2.90440	-2.50780
H	1.53770	-1.26310	-1.56480
H	3.58690	-1.39480	-2.95230
H	4.42200	0.62860	-4.11120
H	3.20510	2.77780	-3.87960
C	-6.07240	1.91110	-1.11580
C	-6.67310	2.63280	-0.08080
C	-8.01500	2.43340	0.21410
C	-8.76260	1.51670	-0.51960
C	-8.16670	0.79590	-1.55130
C	-6.82590	0.99120	-1.84870
H	-6.09210	3.34400	0.48800
H	-8.47820	2.99320	1.01630
H	-9.80900	1.36370	-0.28740
H	-8.74740	0.08270	-2.12180
H	-6.35130	0.43730	-2.64690
O	-0.43040	-0.10480	-0.49480
O	-4.07030	1.48500	-2.35220
O	1.72410	5.22110	-3.41380
C	0.11610	2.89740	2.85780
H	1.03640	2.39740	3.15070
H	0.25540	3.97910	2.90520
H	-0.69350	2.60220	3.53170

Methyl α-D-Galf **4**, C2-exo, +60°/-60°

	**X**	**Y**	**Z**
H	-2.96580	3.60100	-1.27670
O	-3.67770	8.62150	-1.12720

O	-2.49650	5.09700	-4.20540
O	-5.28320	5.48720	-1.95180
O	-1.95920	5.40580	-1.35010
O	-2.56230	8.27370	-3.62000
C	-3.31120	4.67660	-3.10860
C	-4.00720	5.88600	-2.47230
C	-3.08280	6.28170	-1.29670
C	-2.57980	7.72730	-1.26390
C	-2.38410	4.19840	-1.99490
C	-1.74030	8.17540	-2.44190
H	-4.02050	3.91920	-3.42990
H	-4.14870	6.68410	-3.18610
H	-1.91270	7.80050	-0.39660
O	-1.30410	3.48930	-2.46270
H	-3.64370	6.11040	-0.36990
H	-0.93450	7.46400	-2.62050
H	-1.31050	9.15330	-2.22970
C	-3.09220	5.19520	-5.41070
C	-6.38960	6.06530	-2.46700
C	-2.00730	8.84270	-4.70060
C	-2.94540	8.92080	-5.84810
C	-4.28090	8.52470	-5.74150
C	-5.11270	8.59350	-6.85020
C	-4.62090	9.06050	-8.06550
C	-3.29260	9.46240	-8.17160
C	-2.45720	9.39280	-7.06730
H	-4.67020	8.16050	-4.80260
H	-6.14480	8.27850	-6.76550
H	-5.27130	9.10830	-8.92980
H	-2.90660	9.81830	-9.11800
H	-1.41980	9.68740	-7.14010
C	-2.15970	5.66970	-6.45950
C	-0.83180	5.99500	-6.17300
C	-2.64560	5.81190	-7.76050
C	-1.81160	6.27480	-8.76580

C	-0.48940	6.60160	-8.47780
C	-0.00170	6.46200	-7.18210
H	-4.08850	8.48140	-0.26490
H	-0.45840	5.89310	-5.16470
H	-3.67870	5.57050	-7.96670
H	-2.19390	6.39430	-9.77100
H	0.15870	6.97200	-9.26200
H	1.02390	6.72330	-6.95630
C	-7.63270	5.54840	-1.84250
C	-7.61300	4.55780	-0.85720
C	-8.80430	4.10620	-0.30520
C	-10.01780	4.63890	-0.73090
C	-10.04070	5.62650	-1.71240
C	-8.85290	6.07980	-2.26710
H	-6.67110	4.14420	-0.52790
H	-8.78640	3.33810	0.45720
H	-10.94500	4.28470	-0.29840
H	-10.98380	6.04130	-2.04390
H	-8.85680	6.84580	-3.03020
O	-4.25930	4.92530	-5.59210
O	-6.35990	6.90920	-3.33460
O	-0.86100	9.24010	-4.72070
C	-0.56420	2.81540	-1.44020
H	0.18830	2.21510	-1.94610
H	-0.07660	3.53140	-0.77570
H	-1.22290	2.16490	-0.85710

Methyl α-D-Galf **4**, C2-exo, +60°/180°

	**X**	**Y**	**Z**
H	-3.74170	3.92690	4.27510
O	-3.09250	5.73190	-0.47570
O	-0.60120	4.50800	3.36510
O	-3.17940	2.70560	1.58930
O	-3.31910	5.62510	3.17330

O	-1.34290	7.46860	2.13160
C	-1.84370	3.80880	3.26360
C	-2.39260	3.88010	1.83490
C	-3.31440	5.11910	1.84020
C	-2.93960	6.22820	0.85100
C	-2.88870	4.58820	4.05960
C	-1.51100	6.74730	0.90540
H	-1.72770	2.78240	3.60130
H	-1.60190	3.93330	1.09570
H	-3.61370	7.07200	1.03390
O	-2.37900	5.12100	5.22190
H	-4.32260	4.78110	1.57300
H	-1.35000	7.41390	0.05870
H	-0.77510	5.94460	0.85250
C	0.50620	3.84660	2.97100
C	-2.86900	1.93410	0.52400
C	-0.17330	8.09420	2.31610
C	-0.07890	8.70950	3.66410
C	-1.01830	8.42850	4.65900
C	-0.87130	8.98000	5.92440
C	0.20440	9.81990	6.19920
C	1.13830	10.10620	5.20640
C	1.00030	9.54880	3.94340
H	-1.84080	7.76280	4.44390
H	-1.59390	8.75150	6.69730
H	0.31690	10.24930	7.18680
H	1.97540	10.75820	5.42010
H	1.72660	9.75080	3.16830
C	1.73630	4.64330	3.18890
C	1.74110	5.77180	4.01090
C	2.91330	4.23690	2.55630
C	4.08200	4.96220	2.73520
C	4.08380	6.08820	3.55550
C	2.91490	6.48890	4.19500
H	-4.01910	5.50270	-0.62050

H	0.83200	6.08180	4.50340
H	2.89770	3.36050	1.92280
H	4.99090	4.65260	2.23580
H	4.99690	6.65310	3.69490
H	2.91350	7.36430	4.83040
C	-3.76610	0.75720	0.40940
C	-4.80040	0.52020	1.31860
C	-5.61190	-0.59630	1.16870
C	-5.39680	-1.48000	0.11490
C	-4.36700	-1.24640	-0.79280
C	-3.55400	-0.13200	-0.64690
H	-4.96650	1.20590	2.13650
H	-6.41240	-0.77770	1.87430
H	-6.03110	-2.35000	0.00100
H	-4.19890	-1.93310	-1.61240
H	-2.75060	0.06020	-1.34460
O	0.47380	2.73450	2.49180
O	-1.96610	2.18560	-0.24090
O	0.69980	8.13580	1.47480
C	-3.36660	5.68390	6.08830
H	-2.85210	5.96200	7.00520
H	-3.81890	6.57080	5.64010
H	-4.14620	4.94900	6.31090

Methyl α-D-Galf **4**, C2-exo, -60°/+60°

	**X**	**Y**	**Z**
H	-1.60010	2.88400	0.73870
O	-3.72760	4.92630	-2.97140
O	-2.52890	1.90390	-2.29640
O	-4.58490	2.60420	0.54780
O	-1.96840	4.29460	-0.72870
O	-3.90100	7.68590	-2.34260
C	-2.75650	2.10480	-0.90030
C	-3.97270	3.00700	-0.68740

C	-3.38350	4.43590	-0.59220
C	-3.90770	5.41170	-1.64370
C	-1.61360	2.95920	-0.35930
C	-3.18740	6.73680	-1.52740
H	-2.85920	1.15140	-0.38960
H	-4.69530	2.90060	-1.49050
H	-4.96970	5.56520	-1.43600
O	-0.39210	2.61380	-0.88820
H	-3.61940	4.83710	0.39990
H	-2.16160	6.65310	-1.88410
H	-3.17790	7.08440	-0.49410
C	-3.27610	0.96790	-2.91160
C	-5.82480	3.07420	0.78720
C	-3.42720	8.93860	-2.36660
C	-4.22010	9.83220	-3.25110
C	-5.29680	9.35990	-4.00600
C	-6.00630	10.23100	-4.82210
C	-5.64790	11.57440	-4.88850
C	-4.57540	12.04820	-4.13730
C	-3.86300	11.18040	-3.32220
H	-5.57230	8.31650	-3.95520
H	-6.83900	9.86190	-5.40690
H	-6.20340	12.25140	-5.52530
H	-4.29590	13.09260	-4.18820
H	-3.02750	11.53550	-2.73470
C	-2.98230	0.88100	-4.36250
C	-2.08270	1.75020	-4.98570
C	-3.63860	-0.09660	-5.11380
C	-3.39560	-0.20690	-6.47500
C	-2.49890	0.66050	-7.09390
C	-1.84520	1.63800	-6.34900
H	-4.50710	4.42540	-3.23660
H	-1.57890	2.50950	-4.40570
H	-4.33370	-0.76250	-4.62120
H	-3.90420	-0.96680	-7.05420

H	-2.31050	0.57500	-8.15670
H	-1.15020	2.31330	-6.83090
C	-6.38640	2.57710	2.06590
C	-5.67900	1.70190	2.89440
C	-6.24750	1.26200	4.08210
C	-7.52000	1.69130	4.44840
C	-8.22720	2.56350	3.62480
C	-7.66310	3.00560	2.43720
H	-4.69200	1.36840	2.60870
H	-5.69840	0.58360	4.72210
H	-7.96070	1.34620	5.37520
H	-9.21660	2.89740	3.90920
H	-8.20200	3.68260	1.78890
O	-4.09380	0.28900	-2.32840
O	-6.39740	3.82150	0.02330
O	-2.45640	9.29210	-1.72950
C	0.72020	3.22310	-0.22600
H	1.61670	2.78400	-0.65740
H	0.72620	4.30270	-0.38710
H	0.68790	3.01370	0.84760

Methyl α-D-Galf **4**, C2-exo, -60°/-60°

	**X**	**Y**	**Z**
H	-0.45640	5.84360	-1.48280
O	-4.87920	6.46800	-2.79350
O	-2.22670	5.61560	-4.28220
O	-2.11200	3.41030	-1.45430
O	-2.31360	6.74430	-1.62950
O	-4.45990	8.29380	-0.65610
C	-1.64580	5.06420	-3.09920
C	-2.73110	4.47030	-2.20080
C	-3.16050	5.64390	-1.28200
C	-4.62530	6.03940	-1.46300
C	-1.13500	6.22880	-2.25790

C	-5.13490	7.04260	-0.44220
H	-0.87660	4.33960	-3.35150
H	-3.56160	4.07060	-2.77430
H	-5.21030	5.12980	-1.31240
O	-0.51990	7.20470	-3.00670
H	-2.98940	5.35750	-0.23990
H	-4.94740	6.69250	0.57420
H	-6.20700	7.18980	-0.57360
C	-2.53270	4.76060	-5.27670
C	-2.93610	2.58500	-0.78020
C	-4.76350	9.31220	0.15590
C	-3.97400	10.53150	-0.15920
C	-3.00540	10.53620	-1.16640
C	-2.27940	11.69120	-1.42390
C	-2.51730	12.84530	-0.68250
C	-3.48330	12.84380	0.32060
C	-4.20920	11.69040	0.58280
H	-2.82160	9.63710	-1.73630
H	-1.52710	11.69090	-2.20220
H	-1.95030	13.74510	-0.88570
H	-3.66870	13.74080	0.89720
H	-4.96050	11.67510	1.36060
C	-3.16570	5.44720	-6.42840
C	-3.40850	6.82340	-6.42400
C	-3.53080	4.68050	-7.53730
C	-4.13200	5.28390	-8.63220
C	-4.37270	6.65550	-8.62540
C	-4.01060	7.42280	-7.52180
H	-4.21720	7.13780	-3.01510
H	-3.12670	7.41650	-5.56630
H	-3.33990	3.61620	-7.52870
H	-4.41390	4.68710	-9.48990
H	-4.84280	7.12590	-9.47980
H	-4.19880	8.48870	-7.51640
C	-2.20750	1.50760	-0.06860

C	-0.81590	1.39230	-0.12480
C	-0.17860	0.36570	0.55880
C	-0.92330	-0.54810	1.29910
C	-2.31020	-0.43560	1.35680
C	-2.95110	0.58870	0.67560
H	-0.23910	2.10140	-0.70040
H	0.89910	0.27760	0.51380
H	-0.42330	-1.34780	1.83080
H	-2.88940	-1.14600	1.93220
H	-4.02730	0.68730	0.71170
O	-2.31110	3.57090	-5.21400
O	-4.13970	2.72950	-0.77080
O	-5.58540	9.23310	1.04570
C	0.20060	8.16590	-2.22880
H	0.71160	8.81610	-2.93500
H	-0.47790	8.75750	-1.61200
H	0.93510	7.66680	-1.58970

Methyl α-D-Galf **4**, C2-exo, -60°/180°

	**X**	**Y**	**Z**
H	-5.95550	3.52980	1.01940
O	-2.23980	6.00410	-0.48120
O	-4.23050	3.83220	-1.80820
O	-6.69310	6.00310	-0.35820
O	-4.11260	4.46870	1.03230
O	-3.30850	7.51120	2.59160
C	-5.32310	4.14710	-0.94280
C	-5.33080	5.64540	-0.64890
C	-4.44190	5.79330	0.60840
C	-3.16610	6.61180	0.40860
C	-5.02640	3.54910	0.42990
C	-2.41720	6.79730	1.71890
H	-6.26110	3.80190	-1.36800
H	-4.98200	6.23100	-1.49590

H	-3.47340	7.59490	0.04040
O	-4.47500	2.29050	0.35120
H	-5.03470	6.28670	1.38270
H	-1.51310	7.38370	1.55730
H	-2.14980	5.83880	2.16220
C	-4.42710	3.99740	-3.13150
C	-6.95160	7.31940	-0.23000
C	-2.95220	7.65160	3.87540
C	-3.99120	8.35720	4.67000
C	-5.20740	8.75310	4.10630
C	-6.15380	9.40320	4.88690
C	-5.89270	9.66300	6.22940
C	-4.68170	9.27030	6.79360
C	-3.73400	8.61860	6.01730
H	-5.40960	8.55110	3.06430
H	-7.09540	9.70700	4.44790
H	-6.63220	10.17090	6.83560
H	-4.47780	9.47220	7.83720
H	-2.79030	8.30740	6.44390
C	-3.20650	3.70120	-3.91930
C	-1.99410	3.37450	-3.30560
C	-3.28570	3.76640	-5.31230
C	-2.16440	3.50370	-6.08540
C	-0.95730	3.17820	-5.47170
C	-0.87370	3.11570	-4.08370
H	-2.52650	6.14290	-1.39120
H	-1.93090	3.32940	-2.22830
H	-4.22860	4.02250	-5.77570
H	-2.22950	3.55290	-7.16460
H	-0.08200	2.97460	-6.07550
H	0.06520	2.86520	-3.60720
C	-8.36010	7.59330	0.14020
C	-9.29110	6.56770	0.32430
C	-10.59670	6.87490	0.68260
C	-10.97850	8.20170	0.85960

C	-10.05260	9.22580	0.67730
C	-8.74730	8.92370	0.31850
H	-8.99310	5.53820	0.18820
H	-11.31660	6.07930	0.82470
H	-11.99730	8.43790	1.14020
H	-10.34920	10.25740	0.81570
H	-8.01760	9.70880	0.17560
O	-5.48580	4.35360	-3.60120
O	-6.10250	8.16880	-0.39540
O	-1.90120	7.24200	4.32080
C	-4.40860	1.60730	1.60620
H	-4.05840	0.59990	1.39360
H	-3.71030	2.10260	2.28360
H	-5.39850	1.56220	2.07040

Methyl α-D-Galf **4**, C2-exo, 180°/+60°

	**X**	**Y**	**Z**
H	-3.49050	2.34650	1.41380
O	-0.81990	6.26440	1.89430
O	-0.26500	2.14510	2.13770
O	-1.91780	2.55290	-1.04280
O	-2.50000	4.14050	1.47070
O	1.98230	5.69880	0.96220
C	-1.35000	2.08060	1.20980
C	-1.04970	2.98400	0.01580
C	-1.44900	4.37340	0.51820
C	-0.30650	5.12610	1.20970
C	-2.56980	2.75470	1.84680
C	0.74450	5.59740	0.22860
H	-1.54260	1.05270	0.91480
H	-0.01660	2.91300	-0.30820
H	0.17020	4.45340	1.92590
O	-2.57700	2.60020	3.21960
H	-1.85980	4.98270	-0.28810

H	0.47630	6.57040	-0.18300
H	0.88020	4.89700	-0.59380
C	0.85240	1.44580	1.84570
C	-1.61670	2.99000	-2.28430
C	3.07670	6.04300	0.27060
C	4.31080	5.97510	1.09570
C	4.31910	5.37810	2.35890
C	5.49910	5.31680	3.08760
C	6.67210	5.85330	2.56490
C	6.66700	6.44760	1.30540
C	5.49140	6.50480	0.57070
H	3.41120	4.95340	2.76130
H	5.50350	4.84660	4.06190
H	7.59030	5.80610	3.13680
H	7.57900	6.86400	0.89740
H	5.47490	6.95940	-0.41040
C	1.94790	1.72970	2.80310
C	1.77130	2.58460	3.89470
C	3.19190	1.13460	2.58080
C	4.24850	1.39030	3.44220
C	4.06790	2.23750	4.53200
C	2.83060	2.83360	4.75640
H	-1.52380	5.94890	2.47800
H	0.81160	3.05010	4.06500
H	3.31900	0.47960	1.72990
H	5.21250	0.93200	3.26430
H	4.89340	2.43760	5.20310
H	2.69250	3.49690	5.60030
C	-2.57370	2.50410	-3.30600
C	-3.66830	1.70170	-2.97320
C	-4.54000	1.27460	-3.96560
C	-4.32470	1.64380	-5.29040
C	-3.23470	2.44320	-5.62490
C	-2.36150	2.87310	-4.63660
H	-3.83450	1.41600	-1.94470

H	-5.38760	0.65370	-3.70600
H	-5.00630	1.30890	-6.06210
H	-3.06730	2.73050	-6.65480
H	-1.51190	3.49490	-4.88300
O	0.93100	0.69780	0.89700
O	-0.66230	3.70110	-2.51000
O	3.04840	6.36420	-0.89920
C	-3.80440	2.99620	3.83680
H	-3.72070	2.73680	4.88970
H	-3.96270	4.07190	3.73610
H	-4.64810	2.46000	3.39150

Methyl α-D-Galf **4**, C2-exo, 180°/-60°

	**X**	**Y**	**Z**
H	-4.96710	3.90500	2.04850
O	-0.98430	5.37680	0.16090
O	-4.32280	1.77020	-0.41620
O	-5.59470	5.12420	-0.65650
O	-3.08290	3.84830	1.19690
O	-2.79400	6.92900	-1.57330
C	-4.94360	2.93920	0.12570
C	-4.52270	4.17370	-0.67660
C	-3.31120	4.74260	0.10170
C	-2.00590	4.84460	-0.67870
C	-4.32570	3.20180	1.49590
C	-2.10310	5.71200	-1.91390
H	-6.02290	2.81930	0.15580
H	-4.29120	3.91240	-1.70400
H	-1.72830	3.83800	-1.01630
O	-4.11810	2.05230	2.21950
H	-3.58220	5.72880	0.48560
H	-2.65840	5.20560	-2.70140
H	-1.10320	5.94350	-2.27970
C	-4.93400	1.18610	-1.46690

C	-5.89190	5.78210	-1.79850
C	-3.06360	7.77090	-2.58320
C	-3.94730	8.88910	-2.16910
C	-4.46790	8.97730	-0.87620
C	-5.32980	10.01290	-0.54490
C	-5.67480	10.96500	-1.49820
C	-5.15440	10.88340	-2.78690
C	-4.29500	9.84800	-3.12250
H	-4.21140	8.22860	-0.14110
H	-5.74340	10.06940	0.45330
H	-6.35540	11.76630	-1.23990
H	-5.42580	11.62180	-3.53020
H	-3.89280	9.76620	-4.12280
C	-4.20650	-0.01470	-1.94370
C	-3.03650	-0.46380	-1.32520
C	-4.71980	-0.70470	-3.04440
C	-4.07010	-1.83330	-3.52220
C	-2.90460	-2.27900	-2.90390
C	-2.38980	-1.59390	-1.80690
H	-0.94270	4.83170	0.95780
H	-2.63950	0.06880	-0.47340
H	-5.62520	-0.34740	-3.51540
H	-4.47020	-2.36540	-4.37550
H	-2.39780	-3.16000	-3.27720
H	-1.48410	-1.94090	-1.32650
C	-6.95480	6.79480	-1.60000
C	-7.60440	6.95820	-0.37440
C	-8.57000	7.94440	-0.22990
C	-8.88650	8.77340	-1.30200
C	-8.23910	8.61310	-2.52370
C	-7.27870	7.62530	-2.67440
H	-7.34950	6.32090	0.45960
H	-9.07170	8.07090	0.72080
H	-9.63350	9.54810	-1.18370
H	-8.47680	9.26470	-3.35430

H	-6.75870	7.49950	-3.61340
O	-5.95920	1.61310	-1.95130
O	-5.33230	5.56910	-2.85060
O	-2.63600	7.61100	-3.70670
C	-3.77580	2.28130	3.59010
H	-3.74480	1.30550	4.06900
H	-2.79910	2.76190	3.67290
H	-4.53300	2.90520	4.07440

Methyl α-D-Galf **4**, C2-exo, 180°/180°

	**X**	**Y**	**Z**
H	-0.58120	3.79280	3.99380
O	-3.53360	4.98380	0.67640
O	-0.43870	6.84080	4.41320
O	1.02450	6.30130	1.35370
O	-1.25890	4.28230	2.15790
O	-2.46060	8.36060	1.02320
C	-0.10260	5.87000	3.42470
C	-0.25930	6.40710	1.99580
C	-1.27050	5.46730	1.33480
C	-2.69970	6.00660	1.21240
C	-1.04870	4.65530	3.51270
C	-2.81730	7.19200	0.26780
H	0.92390	5.54040	3.56790
H	-0.57790	7.44330	1.99050
H	-3.06020	6.31050	2.19940
O	-2.22250	5.00030	4.17790
H	-0.92980	5.16260	0.34490
H	-3.84850	7.28190	-0.07460
H	-2.16000	7.09910	-0.59590
C	0.48610	7.78590	4.67950
C	1.25840	7.12230	0.31370
C	-2.22780	9.49460	0.34690
C	-1.74900	10.58520	1.23520

C	-1.59910	10.40330	2.61270
C	-1.14810	11.44920	3.40610
C	-0.83950	12.67820	2.83070
C	-0.98440	12.86250	1.45800
C	-1.43960	11.82050	0.66270
H	-1.83640	9.44920	3.05940
H	-1.03580	11.30570	4.47160
H	-0.48580	13.49160	3.45170
H	-0.74310	13.81760	1.00940
H	-1.55760	11.95100	-0.40420
C	-0.00610	8.79480	5.64800
C	-1.32860	8.80190	6.09800
C	0.88230	9.78030	6.08340
C	0.45320	10.76360	6.96240
C	-0.86700	10.77220	7.40540
C	-1.75570	9.79220	6.97220
H	-3.33180	4.16690	1.15290
H	-2.01750	8.04390	5.75500
H	1.90110	9.76870	5.72150
H	1.14360	11.52680	7.29720
H	-1.20400	11.54460	8.08520
H	-2.78280	9.80220	7.31290
C	2.62630	6.96370	-0.23760
C	3.54720	6.06750	0.31120
C	4.81790	5.95850	-0.23730
C	5.17550	6.74020	-1.33220
C	4.25970	7.63420	-1.88110
C	2.98900	7.74640	-1.33620
H	3.26870	5.46290	1.16200
H	5.53020	5.26450	0.18980
H	6.16740	6.65320	-1.75740
H	4.53730	8.24230	-2.73230
H	2.26810	8.43690	-1.75180
O	1.58240	7.80020	4.16520
O	0.43140	7.90050	-0.11040

O	-2.39850	9.60190	-0.84840
C	-3.05980	3.88470	4.48050
H	-3.89050	4.26850	5.06840
H	-3.44420	3.41750	3.57120
H	-2.50910	3.14010	5.06380

Methyl α-D-Galf **4**, C3-exo, +60°/+60°

	**X**	**Y**	**Z**
C	3.46950	4.56740	-2.74710
O	4.25010	5.46280	-3.52650
C	2.05480	4.78090	-3.27690
C	2.28910	4.95680	-4.77460
C	3.64110	5.67820	-4.81330
H	3.59840	4.86050	-1.70240
O	3.83600	3.23500	-2.93180
O	1.30070	5.78840	-5.39030
H	1.65000	5.70250	-2.86150
O	1.20810	3.68560	-2.95230
C	-0.11130	3.93150	-2.80530
C	0.23660	5.18530	-5.95650
H	2.30180	3.99060	-5.27060
C	4.59160	5.26370	-5.93400
C	4.99750	3.80370	-5.89950
H	5.50050	5.86850	-5.83080
O	3.92230	5.58710	-7.14920
O	5.90660	3.63200	-7.00780
H	5.50790	3.55610	-4.97300
H	4.14780	3.13570	-6.03350
H	4.49350	5.32700	-7.88340
H	3.45640	6.74840	-4.92460
C	6.39360	2.39730	-7.20850
C	-0.88220	2.69480	-2.53470
C	-2.26680	2.80080	-2.38370
C	-0.26640	1.44420	-2.43860

C	-1.03250	0.31330	-2.19050
C	-2.41190	0.42320	-2.03970
C	-3.02840	1.66810	-2.13700
H	-2.73280	3.77320	-2.46420
H	0.80380	1.36080	-2.55900
H	-0.55380	-0.65460	-2.11610
H	-3.00660	-0.46090	-1.84760
H	-4.10120	1.75370	-2.02180
C	-0.71290	6.15990	-6.54620
C	-0.45530	7.53300	-6.55810
C	-1.37650	8.40520	-7.12220
C	-2.55720	7.91440	-7.67230
C	-2.81770	6.54640	-7.65980
C	-1.89860	5.67140	-7.09980
H	0.46080	7.91260	-6.12960
H	-1.17420	9.46840	-7.13230
H	-3.27450	8.59730	-8.10990
H	-3.73640	6.16430	-8.08570
H	-2.08860	4.60710	-7.08230
C	7.30060	2.32460	-8.38260
C	7.57190	3.43980	-9.17970
C	8.42580	3.32250	-10.26820
C	9.01250	2.09600	-10.56660
C	8.74450	0.98290	-9.77410
C	7.89110	1.09580	-8.68610
H	7.11660	4.39160	-8.94740
H	8.63390	4.18780	-10.88420
H	9.67830	2.00770	-11.41580
H	9.20060	0.02890	-10.00530
H	7.67400	0.23920	-8.06300
O	6.11180	1.45560	-6.49850
O	0.08390	3.98360	-5.97130
O	-0.59380	5.03870	-2.89120
C	5.05730	2.87880	-2.28450
H	5.21790	1.82100	-2.47900

H	5.89800	3.45320	-2.68070
H	4.98110	3.04820	-1.20610

Methyl α-D-Galf **4**, C3-exo, +60°/-60°

	**X**	**Y**	**Z**
C	-0.43270	5.38590	-3.84010
O	0.85960	6.00500	-3.83030
C	-0.28730	4.23360	-4.82990
C	0.66500	4.81140	-5.88410
C	1.34610	6.00400	-5.16960
H	-1.16540	6.10620	-4.23480
O	-0.78320	4.96960	-2.57840
O	-0.09480	5.30160	-6.99720
H	-1.23260	3.91870	-5.26290
O	0.33520	3.14450	-4.14440
C	0.21930	1.92240	-4.70110
C	0.12780	4.75290	-8.21080
H	1.36830	4.06920	-6.23230
C	2.87210	6.00810	-5.12850
C	3.53080	4.84350	-4.40780
H	3.15730	6.89790	-4.55290
O	3.29350	6.13260	-6.48110
O	3.35260	3.63350	-5.16490
H	4.59880	5.03740	-4.30090
H	3.09770	4.72150	-3.41490
H	4.25650	6.19820	-6.50650
H	1.03260	6.91720	-5.68590
C	4.03800	2.55400	-4.75760
C	0.92000	0.88050	-3.91470
C	0.88080	-0.43580	-4.37790
C	1.62760	1.18780	-2.75020
C	2.29240	0.18390	-2.06050
C	2.25080	-1.12700	-2.52510
C	1.54320	-1.43560	-3.68360

H	0.34120	-0.65870	-5.28740
H	1.66510	2.20760	-2.39660
H	2.84850	0.42540	-1.16410
H	2.77550	-1.90740	-1.98840
H	1.52010	-2.45320	-4.05100
C	-0.74140	5.34380	-9.25860
C	-1.67260	6.34560	-8.97270
C	-2.46390	6.86580	-9.98800
C	-2.33150	6.39110	-11.28980
C	-1.40450	5.39260	-11.57780
C	-0.61200	4.87000	-10.56630
H	-1.77480	6.71330	-7.96230
H	-3.18460	7.64170	-9.76410
H	-2.95020	6.79850	-12.07940
H	-1.30110	5.02270	-12.58970
H	0.11110	4.09420	-10.77690
C	3.80990	1.37360	-5.62770
C	3.03710	1.44720	-6.78920
C	2.83630	0.31020	-7.55900
C	3.40610	-0.90120	-7.17870
C	4.18110	-0.97530	-6.02500
C	4.38240	0.15760	-5.25150
H	2.59100	2.38290	-7.09080
H	2.23090	0.36980	-8.45420
H	3.24330	-1.78760	-7.77890
H	4.61790	-1.91850	-5.72350
H	4.96980	0.11060	-4.34550
O	4.75870	2.56100	-3.78170
O	0.94610	3.88340	-8.41070
O	-0.38910	1.72230	-5.72970
C	-1.17560	6.03060	-1.70260
H	-1.52560	5.56240	-0.78550
H	-0.33100	6.68570	-1.48050
H	-1.98520	6.61580	-2.14890

Methyl α-D-Galf **4**, C3-exo, +60°/180°

	X	Y	Z
C	5.32200	5.35140	-5.02090
O	4.42350	4.96210	-6.06120
C	4.65930	4.86200	-3.72270
C	3.16420	4.86430	-4.06090
C	3.10010	5.14280	-5.57590
H	5.39220	6.44960	-5.00460
O	6.55750	4.78030	-5.21860
O	2.45220	5.91550	-3.39490
H	4.89800	5.50310	-2.87930
O	5.03680	3.51430	-3.43540
C	6.10430	3.31930	-2.63500
C	2.12240	5.70330	-2.10450
H	2.73120	3.90970	-3.77820
C	2.13750	4.29440	-6.40660
C	2.11520	2.79740	-6.11990
H	2.41350	4.45050	-7.45480
O	0.83740	4.81700	-6.14310
O	3.43750	2.27150	-6.29300
H	1.77210	2.59530	-5.10450
H	1.42900	2.30770	-6.81300
H	0.20590	4.40740	-6.74770
H	2.79150	6.18620	-5.70770
C	3.59360	0.94600	-6.15570
C	6.41480	1.87880	-2.46960
C	7.65790	1.52620	-1.94080
C	5.50980	0.88250	-2.83990
C	5.84840	-0.45370	-2.68540
C	7.09310	-0.80240	-2.17040
C	7.99780	0.18860	-1.79820
H	8.35160	2.30610	-1.65760
H	4.54770	1.15600	-3.24540
H	5.14630	-1.22260	-2.97880

H	7.35970	-1.84610	-2.06150
H	8.96690	-0.08250	-1.39940
C	1.34800	6.82720	-1.52460
C	0.97440	7.93930	-2.28370
C	0.24650	8.96520	-1.69610
C	-0.11030	8.88790	-0.35280
C	0.26100	7.78100	0.40620
C	0.98730	6.75310	-0.17710
H	1.25060	7.99740	-3.32640
H	-0.04300	9.82520	-2.28600
H	-0.67750	9.68990	0.10250
H	-0.01590	7.72080	1.45070
H	1.28210	5.88770	0.40030
C	5.01960	0.54080	-6.20710
C	6.04590	1.48610	-6.15130
C	7.36850	1.06730	-6.11940
C	7.67290	-0.29000	-6.15390
C	6.65060	-1.23350	-6.22130
C	5.32700	-0.82030	-6.24250
H	5.80920	2.53780	-6.09680
H	8.70560	-0.61400	-6.12220
H	6.88710	-2.28940	-6.24550
H	4.52340	-1.54330	-6.27470
O	2.66380	0.18400	-5.98780
O	2.43710	4.69880	-1.50460
O	6.72570	4.22200	-2.12030
C	7.30220	5.34590	-6.29930
H	6.82350	5.13140	-7.25670
H	7.39930	6.42810	-6.17240
H	8.28620	4.88380	-6.27170
H	8.16150	1.80130	-6.05450

Methyl α-D-Galf **4**, C3-exo, -60°/+60°

	X	Y	Z
C	2.41030	4.25110	-2.41470
O	3.74390	4.00560	-2.83830
C	1.91790	5.31970	-3.38890
C	2.55100	4.86080	-4.69740
C	3.91060	4.34150	-4.22890
H	2.46590	4.58910	-1.37610
O	1.59380	3.12730	-2.51810
O	2.76950	5.92250	-5.63110
H	2.32500	6.28840	-3.10240
O	0.49690	5.37800	-3.41690
C	-0.07680	6.55400	-3.74610
C	1.80650	6.16950	-6.54340
H	1.94990	4.08210	-5.15820
C	4.44910	3.17570	-5.04090
C	5.77480	2.69690	-4.48220
H	4.61510	3.55480	-6.05810
O	3.48930	2.12810	-5.06130
O	6.23140	1.65770	-5.37190
H	6.50820	3.50400	-4.45900
H	5.65330	2.28700	-3.48070
H	3.84220	1.41170	-5.60390
H	4.63810	5.15530	-4.29280
C	7.36420	1.01930	-5.03840
C	-1.55580	6.46270	-3.78390
C	-2.27890	7.61030	-4.11750
C	-2.23170	5.27160	-3.50660
C	-3.61850	5.23450	-3.56150
C	-4.33550	6.38050	-3.89330
C	-3.66430	7.56850	-4.17170
H	-1.74480	8.52550	-4.33290
H	-1.67310	4.38290	-3.25190
H	-4.14040	4.31100	-3.34650
H	-5.41690	6.34790	-3.93610
H	-4.22130	8.45940	-4.43110

C	2.14630	7.31550	-7.42090
C	3.33670	8.03300	-7.27790
C	3.60570	9.10280	-8.12090
C	2.69150	9.46200	-9.10720
C	1.50340	8.74990	-9.25090
C	1.23080	7.68020	-8.41090
H	4.04400	7.75520	-6.51030
H	4.52820	9.65750	-8.00810
H	2.90370	10.29730	-9.76250
H	0.79130	9.02980	-10.01640
H	0.31140	7.11990	-8.51000
C	7.71840	-0.05520	-6.00130
C	6.91540	-0.35510	-7.10490
C	7.28510	-1.37170	-7.97550
C	8.45470	-2.09230	-7.75140
C	9.25730	-1.79560	-6.65290
C	8.89090	-0.78100	-5.78040
H	6.00790	0.20460	-7.27890
H	6.66060	-1.60210	-8.82900
H	8.74040	-2.88440	-8.43200
H	10.16700	-2.35540	-6.47780
H	9.50540	-0.54170	-4.92350
O	8.01610	1.30520	-4.05680
O	0.78630	5.52220	-6.62330
O	0.55670	7.55890	-3.98260
C	1.93810	2.08380	-1.60890
H	1.17730	1.31350	-1.71370
H	2.91760	1.66430	-1.84680
H	1.94290	2.45700	-0.57940

Methyl α-D-Galf **4**, C3-exo, -60°/-60°

	**X**	**Y**	**Z**
C	-0.14560	1.92930	-4.38730
O	1.13240	2.21880	-4.90090

C	-0.51410	3.17020	-3.56010
C	0.14580	4.28610	-4.35280
C	1.43880	3.62090	-4.81950
H	-0.07930	1.02460	-3.77730
O	-1.02870	1.71380	-5.45780
O	0.47890	5.43170	-3.56380
H	-0.06990	3.10740	-2.56750
O	-1.92870	3.29590	-3.45450
C	-2.42930	3.92470	-2.37190
C	-0.42610	6.42970	-3.48720
H	-0.48030	4.58170	-5.19100
C	1.97460	4.19430	-6.12500
C	3.35320	3.68880	-6.51430
H	2.10390	5.26830	-5.93110
O	1.01060	3.98760	-7.14770
O	3.28300	2.27980	-6.77860
H	3.69960	4.20370	-7.41230
H	4.06720	3.87800	-5.71070
H	1.23800	4.54370	-7.90290
H	2.20650	3.76600	-4.05320
C	4.42040	1.65570	-7.10340
C	-3.90590	4.04220	-2.42840
C	-4.56260	4.63260	-1.34630
C	-4.64250	3.59430	-3.52800
C	-6.02380	3.73480	-3.53890
C	-6.67490	4.32060	-2.45710
C	-5.94290	4.76970	-1.36070
H	-3.98150	4.97910	-0.50280
H	-4.13490	3.14480	-4.36880
H	-6.59260	3.38860	-4.39210
H	-7.75220	4.42900	-2.46880
H	-6.44860	5.22700	-0.52030
C	0.02700	7.53220	-2.60520
C	1.25880	7.49750	-1.94670
C	1.63470	8.55180	-1.12530

C	0.78610	9.64210	-0.95570
C	-0.44300	9.67870	-1.60940
C	-0.82210	8.62770	-2.43130
H	1.91510	6.64950	-2.07620
H	2.58930	8.52280	-0.61600
H	1.08160	10.46230	-0.31360
H	-1.10400	10.52530	-1.47660
H	-1.77460	8.64280	-2.94260
C	4.21540	0.20080	-7.32750
C	2.96690	-0.40030	-7.14490
C	2.81790	-1.76390	-7.36020
C	3.90850	-2.53190	-7.75880
C	5.15330	-1.93510	-7.94130
C	5.30720	-0.57300	-7.72540
H	2.12370	0.19920	-6.83330
H	1.85070	-2.22830	-7.21680
H	3.78880	-3.59490	-7.92660
H	6.00170	-2.53170	-8.25110
H	6.26880	-0.09720	-7.86220
O	5.48900	2.22440	-7.19960
O	-1.48440	6.41120	-4.07550
O	-1.73980	4.34160	-1.46740
C	-2.00530	0.70000	-5.22700
H	-2.53750	0.56170	-6.16600
H	-1.52420	-0.24040	-4.93950
H	-2.71430	0.99530	-4.45050

Methyl α-D-Galf **4**, C3-exo, -60°/180°

	**X**	**Y**	**Z**
C	2.32350	7.19980	-3.85900
O	3.04860	6.46980	-4.83750
C	1.06530	7.64400	-4.60310
C	0.76480	6.42290	-5.46400
C	2.16580	5.95410	-5.85270

H	2.96570	8.02660	-3.54210
O	1.95600	6.42250	-2.76260
O	0.04650	6.73470	-6.66200
H	1.30190	8.49380	-5.24190
O	0.02490	7.99140	-3.69720
C	-0.89180	8.89220	-4.10720
C	-1.29940	6.65890	-6.63160
H	0.22190	5.67560	-4.89200
C	2.30390	4.44960	-6.01810
C	3.72300	4.04370	-6.39500
H	1.62420	4.16140	-6.82870
O	1.94120	3.80400	-4.80530
O	4.00730	4.63790	-7.67460
H	4.43780	4.39790	-5.65360
H	3.79850	2.95850	-6.47400
H	1.73980	2.88000	-4.99610
H	2.43990	6.41860	-6.80230
C	5.25300	4.50380	-8.15290
C	-1.93470	9.13270	-3.08140
C	-2.95560	10.03880	-3.37800
C	-1.92280	8.47940	-1.84620
C	-2.92350	8.73540	-0.91860
C	-3.93840	9.63990	-1.21750
C	-3.95360	10.29120	-2.44830
H	-2.95610	10.53640	-4.33790
H	-1.13510	7.77670	-1.61700
H	-2.91250	8.22850	0.03770
H	-4.71770	9.83680	-0.49210
H	-4.74330	10.99370	-2.68160
C	-1.91110	7.04110	-7.92740
C	-1.13950	7.41570	-9.03050
C	-1.75970	7.77140	-10.22060
C	-3.14840	7.75680	-10.31610
C	-3.92000	7.38550	-9.21800
C	-3.30420	7.02870	-8.02730

H	-0.06210	7.42960	-8.95410
H	-1.16010	8.06110	-11.07380
H	-3.62920	8.03600	-11.24520
H	-4.99980	7.37580	-9.29100
H	-3.89150	6.74070	-7.16640
C	5.43450	5.18630	-9.46030
C	4.39730	5.89280	-10.07500
C	4.61290	6.51790	-11.29600
C	5.86040	6.44210	-11.90890
C	6.89600	5.73900	-11.29880
C	6.68450	5.11310	-10.07870
H	3.42960	5.95170	-9.59830
H	3.80780	7.06500	-11.76940
H	6.02560	6.93050	-12.86090
H	7.86630	5.67960	-11.77450
H	7.48060	4.56440	-9.59450
O	6.12200	3.89110	-7.56960
O	-1.92370	6.31830	-5.65130
O	-0.85470	9.43230	-5.19070
C	3.06070	6.00120	-1.96480
H	2.64510	5.48810	-1.10040
H	3.70610	5.31760	-2.51970
H	3.64600	6.86510	-1.63230

Methyl α-D-Galf **4**, C3-exo, 180°/+60°

	**X**	**Y**	**Z**
C	3.65040	5.80940	-3.01620
O	2.98950	6.90820	-3.61460
C	2.61360	4.69510	-3.10980
C	2.05090	4.91950	-4.51080
C	2.12060	6.44410	-4.66600
H	3.90050	6.10730	-1.99480
O	4.79580	5.41980	-3.71470
O	0.68980	4.50040	-4.64890

H	1.83580	4.86060	-2.36630
O	3.20350	3.41410	-2.92650
C	2.42420	2.42830	-2.43290
C	0.45950	3.24640	-5.09570
H	2.66640	4.39620	-5.23930
C	2.62630	6.90080	-6.02670
C	1.72060	6.39690	-7.13730
H	3.63660	6.50340	-6.17770
O	2.65540	8.32350	-6.00720
O	2.27350	6.91250	-8.36380
H	1.69900	5.30870	-7.18640
H	0.70370	6.77160	-7.01470
H	2.95950	8.62620	-6.87240
H	1.13480	6.87920	-4.48820
C	1.60290	6.63800	-9.49540
C	3.14790	1.13800	-2.34250
C	2.45440	0.02500	-1.86170
C	4.48390	1.00970	-2.73140
C	5.11650	-0.22240	-2.63680
C	4.42200	-1.32920	-2.15670
C	3.09020	-1.20430	-1.76950
H	1.42000	0.13550	-1.56660
H	5.01990	1.86950	-3.10610
H	6.15130	-0.32010	-2.93870
H	4.91800	-2.28910	-2.08540
H	2.54950	-2.06500	-1.39750
C	-0.98660	2.92980	-5.15800
C	-1.96650	3.86190	-4.80710
C	-3.30870	3.51450	-4.87780
C	-3.67890	2.23920	-5.29530
C	-2.70430	1.30770	-5.64420
C	-1.36220	1.65120	-5.57660
H	-1.67780	4.85040	-4.48100
H	-4.06650	4.23800	-4.60630
H	-4.72660	1.97090	-5.34800

H	-2.99190	0.31570	-5.96750
H	-0.59480	0.93760	-5.84320
C	2.23940	7.23700	-10.69590
C	3.40950	7.99640	-10.61340
C	3.96580	8.53910	-11.76390
C	3.35970	8.32850	-12.99920
C	2.19380	7.57200	-13.08480
C	1.63510	7.02770	-11.93760
H	3.87980	8.15910	-9.65450
H	4.87210	9.12700	-11.69730
H	3.79560	8.75360	-13.89460
H	1.72200	7.40770	-14.04490
H	0.73000	6.43850	-11.99030
O	0.59030	5.97200	-9.51300
O	1.34600	2.48230	-5.40590
O	1.26900	2.59460	-2.11000
C	5.85190	6.37820	-3.66040
H	6.70760	5.92980	-4.16020
H	5.57020	7.30070	-4.17230
H	6.11120	6.60510	-2.62130

Methyl α-D-Galf **4**, C3-exo, 180°/-60°

	**X**	**Y**	**Z**
C	1.94830	7.73980	-4.14420
O	2.55750	7.57620	-5.42950
C	1.75810	6.31060	-3.61830
C	1.68010	5.46680	-4.90070
C	1.87910	6.48480	-6.04320
H	0.95770	8.19760	-4.28800
O	2.73260	8.51450	-3.32510
O	0.40640	4.83290	-5.03050
H	0.85610	6.20960	-3.02080
O	2.90050	5.94930	-2.83740
C	2.75920	4.90080	-2.00290

C	0.35740	3.48400	-5.09980
H	2.46270	4.71300	-4.88780
C	2.69940	5.99110	-7.22390
C	2.19410	4.68230	-7.80620
H	3.72150	5.80790	-6.87070
O	2.68700	7.03710	-8.19200
O	0.79520	4.82890	-8.11230
H	2.74230	4.44890	-8.72050
H	2.33040	3.85740	-7.10770
H	3.31920	6.81840	-8.88830
H	0.89350	6.80640	-6.39770
C	0.13570	3.73090	-8.51270
C	4.00360	4.60840	-1.25060
C	4.01080	3.51120	-0.38640
C	5.15190	5.39290	-1.38750
C	6.29390	5.07970	-0.66270
C	6.29730	3.98510	0.19720
C	5.15470	3.20090	0.33450
H	3.11720	2.91000	-0.29010
H	5.14710	6.24180	-2.05560
H	7.18210	5.68910	-0.76890
H	7.19000	3.74270	0.76000
H	5.15730	2.34900	1.00200
C	-1.02150	2.98560	-5.30890
C	-2.12190	3.84490	-5.35940
C	-3.39030	3.33170	-5.58610
C	-3.56730	1.96400	-5.76770
C	-2.47300	1.10480	-5.72040
C	-1.20350	1.61290	-5.49110
H	-1.98080	4.90780	-5.23360
H	-4.23920	4.00030	-5.63620
H	-4.55720	1.56810	-5.95580
H	-2.60980	0.04140	-5.86840
H	-0.34370	0.95840	-5.46040
C	-1.31060	3.98620	-8.72580

C	-1.86260	5.26200	-8.58650
C	-3.22420	5.45120	-8.77540
C	-4.04060	4.37110	-9.09890
C	-3.49320	3.09920	-9.23580
C	-2.13210	2.90670	-9.05190
H	-1.22790	6.09670	-8.32700
H	-3.65040	6.44000	-8.66550
H	-5.10390	4.52020	-9.23880
H	-4.12870	2.25720	-9.47760
H	-1.69580	1.92210	-9.14500
O	0.67580	2.65510	-8.66220
O	1.33970	2.77940	-5.02880
O	1.72500	4.27820	-1.89650
C	2.70690	9.90850	-3.64800
H	3.27760	10.41620	-2.87390
H	3.16460	10.09170	-4.62190
H	1.67830	10.28120	-3.65130

Methyl α-D-Galf **4**, C3-exo, 180°/180°

	**X**	**Y**	**Z**
C	5.70810	3.82220	-3.95520
O	4.85980	4.46840	-4.91170
C	5.67480	2.33480	-4.34830
C	4.36000	2.19680	-5.12550
C	3.74290	3.60740	-5.09350
H	5.26580	3.94860	-2.95610
O	6.97790	4.34460	-4.00310
O	3.42840	1.28100	-4.54200
H	5.71350	1.68770	-3.47760
O	6.75030	2.02410	-5.23530
C	7.86300	1.48080	-4.70890
C	3.70400	-0.03250	-4.68700
H	4.58160	1.87900	-6.14180
C	2.98740	4.02500	-6.34410

C	1.74060	3.18340	-6.60000
H	3.66410	3.95990	-7.20320
O	2.56830	5.37070	-6.13180
O	2.13180	1.88960	-7.08910
H	1.15360	3.07090	-5.68870
H	1.12310	3.67610	-7.35290
H	2.24170	5.72600	-6.96810
H	3.06720	3.67830	-4.23060
C	1.17360	0.94710	-7.12170
C	8.90250	1.23310	-5.73980
C	10.09940	0.63400	-5.34090
C	8.71470	1.58210	-7.07960
C	9.71620	1.33110	-8.00790
C	10.90690	0.73220	-7.60630
C	11.09740	0.38400	-6.27160
H	10.23450	0.36880	-4.30130
H	7.79110	2.04850	-7.38960
H	9.56830	1.60280	-9.04520
H	11.68600	0.53750	-8.33260
H	12.02300	-0.08140	-5.95830
C	2.57360	-0.90060	-4.28550
C	1.34640	-0.37430	-3.87560
C	0.29670	-1.22770	-3.56720
C	0.46670	-2.60590	-3.66360
C	1.69040	-3.13260	-4.06760
C	2.74080	-2.28340	-4.37920
H	1.21180	0.69560	-3.81600
H	-0.65640	-0.81830	-3.25870
H	-0.35580	-3.26930	-3.42770
H	1.82060	-4.20390	-4.14880
H	3.69110	-2.67770	-4.71080
C	1.69010	-0.38060	-7.53320
C	3.00530	-0.56210	-7.96560
C	3.45370	-1.83150	-8.30270
C	2.59640	-2.92300	-8.20510

C	1.28350	-2.74420	-7.77670
C	0.83010	-1.47740	-7.44520
H	3.67380	0.28390	-8.02800
H	4.47520	-1.97070	-8.63200
H	2.95210	-3.91370	-8.45890
H	0.61890	-3.59410	-7.69220
H	-0.18210	-1.32590	-7.09770
O	0.02010	1.17280	-6.82320
O	4.76190	-0.43120	-5.12040
O	7.98610	1.21740	-3.53300
C	7.10700	5.62670	-3.38220
H	8.16490	5.87750	-3.40650
H	6.53770	6.38260	-3.92660
H	6.76230	5.58520	-2.34480

Methyl α-D-Galf **4**, O4-exo, +60°/+60°

	**X**	**Y**	**Z**
C	-0.11900	2.27560	-6.35740
O	-1.22530	3.03950	-6.85280
C	-0.71910	1.40050	-5.25340
C	-1.80800	2.29760	-4.66370
C	-2.11450	3.32590	-5.77460
H	0.62210	2.96160	-5.92230
O	0.43650	1.52450	-7.36650
O	-1.30480	3.02530	-3.53250
O	-1.35870	0.25640	-5.82180
C	-0.67140	-0.90800	-5.82190
C	-1.31320	2.38690	-2.34210
C	-3.55120	3.33730	-6.30920
C	-3.98630	1.99990	-6.87330
H	-3.58160	4.07210	-7.11620
O	-4.44040	3.82810	-5.31340
O	-5.22780	2.21850	-7.56880
C	-5.80220	1.14430	-8.12790

H	-1.91360	4.32300	-5.37320
C	-0.77170	3.22590	-1.24610
C	-7.08260	1.46940	-8.80740
C	-7.62780	2.75560	-8.78070
C	-8.83020	3.01200	-9.42610
C	-9.49230	1.99050	-10.10130
C	-8.95110	0.70780	-10.13010
C	-7.75100	0.44710	-9.48460
H	-7.11380	3.54670	-8.25420
H	-9.25130	4.00880	-9.40230
H	-10.42930	2.19360	-10.60420
H	-9.46510	-0.08710	-10.65500
H	-7.32050	-0.54470	-9.49890
C	-1.41270	-1.99730	-6.50240
C	-2.65840	-1.78820	-7.09880
C	-3.30940	-2.84010	-7.72920
C	-2.72360	-4.10190	-7.76800
C	-1.48120	-4.31340	-7.17450
C	-0.82700	-3.26540	-6.54410
H	-3.12100	-0.81430	-7.07710
H	-4.27350	-2.66930	-8.18940
H	-3.23330	-4.92040	-8.26060
H	-1.02440	-5.29420	-7.20460
H	0.13820	-3.41650	-6.08070
C	-0.72040	2.67930	0.03850
C	-0.21910	3.42740	1.09340
C	0.23420	4.72540	0.87190
C	0.18550	5.27360	-0.40660
C	-0.31560	4.52850	-1.46550
H	-1.07470	1.67010	0.19690
H	-0.18070	3.00050	2.08720
H	0.62590	5.30900	1.69550
H	0.53830	6.28220	-0.57850
H	-0.35320	4.95300	-2.45810
H	0.01750	1.09270	-4.51800

H	-2.66390	1.70550	-4.35260
H	-4.60220	3.13760	-4.65690
O	-1.72070	1.25400	-2.20950
O	0.41800	-1.03250	-5.31010
O	-5.31640	0.03320	-8.07510
H	-4.14620	1.26040	-6.08690
H	-3.24060	1.61750	-7.56830
C	1.22920	2.27570	-8.29010
H	1.69010	1.55460	-8.96110
H	0.60880	2.96650	-8.86430
H	2.00630	2.83460	-7.76050

Methyl α-D-Galf **4**, O4-exo, +60°/-60°

	**X**	**Y**	**Z**
C	-0.56930	2.30830	-3.29610
O	-0.97350	3.30880	-4.23810
C	-1.73620	1.32500	-3.27160
C	-2.95350	2.22860	-3.48120
C	-2.36120	3.55760	-4.02970
H	-0.47830	2.77620	-2.30380
O	0.61490	1.72670	-3.67780
O	-3.59930	2.42370	-2.21340
O	-1.59480	0.44090	-4.38710
C	-2.37040	-0.66320	-4.38630
C	-4.91400	2.69110	-2.23380
C	-2.95260	4.12040	-5.32690
C	-2.84960	3.21920	-6.54690
H	-2.34940	4.99990	-5.56860
O	-4.27250	4.59240	-5.11700
O	-3.75050	2.10600	-6.38820
C	-3.87780	1.26680	-7.42800
H	-2.49490	4.31970	-3.25340
C	-5.50820	2.77400	-0.88210
C	-4.82440	0.15830	-7.14930

C	-5.56130	0.09490	-5.96400
C	-6.41480	-0.97500	-5.73590
C	-6.54150	-1.98230	-6.68810
C	-5.81440	-1.91710	-7.87290
C	-4.95880	-0.85100	-8.10360
H	-5.46970	0.87770	-5.22570
H	-6.98010	-1.02350	-4.81420
H	-7.20450	-2.81860	-6.50530
H	-5.90620	-2.70380	-8.61040
H	-4.37680	-0.79710	-9.01260
C	-2.20040	-1.47230	-5.61460
C	-1.39160	-1.04710	-6.67130
C	-1.28610	-1.82430	-7.81590
C	-1.97840	-3.02780	-7.90830
C	-2.78240	-3.45440	-6.85500
C	-2.89680	-2.67830	-5.71260
H	-0.86120	-0.10900	-6.60000
H	-0.66820	-1.48830	-8.63840
H	-1.89720	-3.63020	-8.80430
H	-3.32920	-4.38510	-6.93160
H	-3.53050	-2.98940	-4.89420
C	-6.86220	3.10160	-0.77370
C	-7.45850	3.18390	0.47570
C	-6.70710	2.93950	1.62240
C	-5.35790	2.61270	1.51820
C	-4.75620	2.52940	0.27040
H	-7.43400	3.28850	-1.67210
H	-8.50730	3.43810	0.55720
H	-7.17330	3.00340	2.59750
H	-4.77530	2.42160	2.41000
H	-3.70980	2.27390	0.18720
H	-1.79960	0.76060	-2.34550
H	-3.65560	1.77170	-4.16410
H	-4.79660	3.90050	-4.67670
O	-5.53890	2.84390	-3.26800

O	-3.12180	-0.93470	-3.47510
O	-3.27120	1.41050	-8.46820
H	-1.83240	2.84770	-6.67000
H	-3.13520	3.77930	-7.43650
C	1.76320	2.55510	-3.46970
H	2.63180	1.94640	-3.70940
H	1.73620	3.42950	-4.12260
H	1.81710	2.87900	-2.42610

Methyl α-D-Galf **4**, O4-exo, +60°/180°

	**X**	**Y**	**Z**
C	-3.24670	2.70350	-8.42350
O	-4.01430	2.38800	-7.26100
C	-1.83390	2.20170	-8.10480
C	-1.72810	2.43260	-6.59620
C	-3.18940	2.53280	-6.10820
H	-3.22480	3.79600	-8.55560
O	-3.78010	2.08580	-9.53180
O	-1.08930	3.68530	-6.30680
O	-1.73390	0.79280	-8.31510
C	-1.29170	0.35620	-9.51220
C	0.26100	3.69760	-6.30680
C	-3.62640	1.53310	-5.02990
C	-3.32760	0.05680	-5.26450
H	-4.70570	1.65110	-4.91700
O	-3.06080	1.91080	-3.77710
O	-4.01390	-0.39360	-6.43960
C	-4.04330	-1.71730	-6.65720
H	-3.32630	3.52910	-5.67610
C	0.81410	5.02880	-5.96010
C	-4.65100	-2.05810	-7.96710
C	-4.88420	-1.08050	-8.93600
C	-5.40050	-1.44310	-10.17250
C	-5.69450	-2.77610	-10.44370

C	-5.46600	-3.75190	-9.47630
C	-4.94030	-3.39520	-8.24320
H	-4.63270	-0.05070	-8.73340
H	-5.56600	-0.68510	-10.92750
H	-6.09590	-3.05650	-11.40950
H	-5.69070	-4.78940	-9.68770
H	-4.74300	-4.14520	-7.48960
C	-1.26630	-1.12520	-9.57370
C	-1.24090	-1.90610	-8.41740
C	-1.23000	-3.28980	-8.51600
C	-1.25950	-3.89950	-9.76610
C	-1.28470	-3.12290	-10.92170
C	-1.27810	-1.73870	-10.82730
H	-1.22920	-1.43050	-7.44780
H	-1.21530	-3.89160	-7.61700
H	-1.26650	-4.97950	-9.84060
H	-1.31240	-3.59690	-11.89430
H	-1.29940	-1.12440	-11.71720
C	2.20270	5.17830	-5.92940
C	2.76330	6.40520	-5.60630
C	1.94090	7.48990	-5.31240
C	0.55680	7.34540	-5.34260
C	-0.00870	6.11920	-5.66530
H	2.83010	4.32810	-6.15920
H	3.83950	6.51720	-5.58300
H	2.37880	8.44750	-5.06010
H	-0.08200	8.18860	-5.11400
H	-1.08280	6.00570	-5.68850
H	-1.06780	2.72430	-8.66990
H	-1.16050	1.63080	-6.13230
H	-2.11190	1.72470	-3.77820
O	0.92530	2.71730	-6.56230
O	-0.96010	1.09220	-10.41300
O	-3.60520	-2.52900	-5.86830
H	-3.66950	-0.50540	-4.39650

H	-2.25680	-0.12430	-5.38810
C	-5.01500	2.64670	-9.98270
H	-5.26340	2.13920	-10.91190
H	-5.80880	2.48100	-9.25200
H	-4.90390	3.71930	-10.16700

Methyl α-D-Galf **4**, O4-exo, -60°/+60°

	**X**	**Y**	**Z**
C	0.18360	2.53430	-5.74110
O	-0.60100	2.16940	-6.88480
C	-0.83070	2.60710	-4.59600
C	-2.10070	3.10150	-5.29210
C	-1.86330	2.83290	-6.79240
H	0.61980	3.52890	-5.91380
O	1.16050	1.59650	-5.51100
O	-2.27300	4.51670	-5.13010
O	-1.09860	1.29530	-4.09840
C	-0.45270	0.89530	-2.98370
C	-2.82740	4.93700	-3.97260
C	-2.91990	1.93870	-7.43060
C	-2.64810	1.76950	-8.91430
H	-3.89120	2.42230	-7.30300
O	-3.01110	0.68780	-6.76270
O	-3.77430	1.07270	-9.47740
C	-3.72170	0.79920	-10.78850
H	-1.82610	3.79220	-7.31790
C	-2.97980	6.41120	-3.92140
C	-4.92200	0.06550	-11.26770
C	-5.94310	-0.32970	-10.39960
C	-7.04460	-1.01690	-10.89230
C	-7.13420	-1.31160	-12.24980
C	-6.11820	-0.91920	-13.11740
C	-5.01530	-0.23390	-12.62860
H	-5.87130	-0.10250	-9.34590

H	-7.83330	-1.32300	-10.21720
H	-7.99460	-1.84700	-12.63120
H	-6.18690	-1.14790	-14.17310
H	-4.21940	0.07600	-13.29180
C	-0.77420	-0.51060	-2.63400
C	-1.61530	-1.29590	-3.42700
C	-1.88240	-2.60880	-3.06330
C	-1.31390	-3.14410	-1.91090
C	-0.47540	-2.36390	-1.11880
C	-0.20540	-1.05160	-1.47870
H	-2.05690	-0.87890	-4.32010
H	-2.53410	-3.21500	-3.67910
H	-1.52400	-4.16840	-1.63010
H	-0.03320	-2.77940	-0.22250
H	0.44430	-0.43570	-0.87250
C	-3.52990	6.97720	-2.76870
C	-3.68990	8.35200	-2.67690
C	-3.30150	9.16970	-3.73500
C	-2.75300	8.60990	-4.88540
C	-2.59090	7.23440	-4.98160
H	-3.82670	6.33110	-1.95410
H	-4.11630	8.78690	-1.78220
H	-3.42630	10.24280	-3.66290
H	-2.45130	9.24560	-5.70770
H	-2.16490	6.79920	-5.87380
H	-0.50580	3.25790	-3.79030
H	-2.97060	2.57980	-4.90480
H	-2.11960	0.31320	-6.71610
O	-3.15440	4.17670	-3.08840
O	0.28760	1.61210	-2.34910
O	-2.78980	1.12420	-11.49480
H	-1.74100	1.18930	-9.08460
H	-2.54400	2.73990	-9.40210
C	2.26150	1.65790	-6.42260
H	2.99780	0.94080	-6.06750

H	1.94600	1.39030	-7.43290
H	2.69730	2.66110	-6.42830

Methyl α-D-Galf **4**, O4-exo, -60°/-60°

	**X**	**Y**	**Z**
C	-0.72330	0.22470	-3.13140
O	-0.98770	-0.29590	-4.44020
C	-1.69860	1.39500	-2.99060
C	-1.76460	1.94900	-4.41420
C	-1.24930	0.80270	-5.31460
H	0.30980	0.60000	-3.09850
O	-0.92920	-0.74560	-2.18020
O	-0.87460	3.06200	-4.58400
O	-2.99930	0.90450	-2.66180
C	-3.38720	0.94290	-1.37090
C	-1.31050	4.26620	-4.15610
C	-2.26020	0.38120	-6.38080
C	-1.69910	-0.54190	-7.44900
H	-2.56640	1.29210	-6.90300
O	-3.43130	-0.15610	-5.78360
O	-1.33900	-1.78730	-6.82910
C	-0.81060	-2.73860	-7.60810
H	-0.32420	1.13020	-5.79910
C	-0.31970	5.34360	-4.39560
C	-0.45500	-3.95810	-6.83700
C	-0.61730	-4.01970	-5.45040
C	-0.26140	-5.17130	-4.76160
C	0.25270	-6.26610	-5.45100
C	0.41320	-6.20830	-6.83310
C	0.06200	-5.05760	-7.52470
H	-1.01410	-3.16620	-4.92040
H	-0.38430	-5.21460	-3.68700
H	0.52890	-7.16360	-4.91180
H	0.81270	-7.05930	-7.36940

H	0.18440	-4.99900	-8.59760
C	-4.71910	0.31730	-1.17740
C	-5.42000	-0.26960	-2.23430
C	-6.66030	-0.84950	-2.00420
C	-7.20590	-0.84750	-0.72360
C	-6.50930	-0.26360	0.33140
C	-5.26950	0.31670	0.10620
H	-4.99510	-0.26970	-3.22740
H	-7.20160	-1.30360	-2.82420
H	-8.17330	-1.30080	-0.54750
H	-6.93300	-0.26180	1.32740
H	-4.71750	0.77270	0.91650
C	-0.64180	6.63900	-3.98360
C	0.25550	7.67700	-4.18680
C	1.47960	7.42800	-4.80230
C	1.80460	6.13870	-5.21410
C	0.90960	5.09670	-5.01260
H	-1.59530	6.81880	-3.50630
H	0.00260	8.67920	-3.86580
H	2.17980	8.23850	-4.96060
H	2.75610	5.94540	-5.69240
H	1.16130	4.09580	-5.33170
H	-1.37450	2.12960	-2.26030
H	-2.77930	2.25140	-4.65670
H	-3.14550	-0.81790	-5.13770
O	-2.39220	4.42460	-3.63510
O	-2.72420	1.44010	-0.48900
O	-0.64200	-2.60290	-8.80230
H	-0.82110	-0.09860	-7.92170
H	-2.45600	-0.72770	-8.21120
C	0.12960	-1.70200	-2.07550
H	-0.12190	-2.35260	-1.24130
H	0.21420	-2.29350	-2.98860
H	1.08000	-1.19860	-1.87640

Methyl α-D-Galf **4**, O4-exo, -60°/180°

	X	Y	Z
C	-1.89240	4.85860	-6.75670
O	-2.41820	3.52630	-6.83490
C	-1.74170	5.11720	-5.25430
C	-2.90230	4.32390	-4.64910
C	-3.34990	3.35800	-5.76460
H	-2.63180	5.55450	-7.17890
O	-0.69430	4.94200	-7.42250
O	-4.02220	5.16540	-4.33940
O	-0.53000	4.52960	-4.77750
C	0.54990	5.32570	-4.63740
C	-3.96600	5.85850	-3.18240
C	-3.33890	1.89210	-5.35290
C	-3.88790	0.98010	-6.44260
H	-3.95770	1.78890	-4.45860
O	-2.03110	1.46760	-4.99840
O	-5.25080	1.37880	-6.67640
C	-5.91540	0.75440	-7.66000
H	-4.35460	3.64070	-6.08870
C	-5.19460	6.65320	-2.94070
C	-7.29300	1.28240	-7.83670
C	-7.78210	2.34220	-7.06830
C	-9.07400	2.80820	-7.27280
C	-9.88310	2.22090	-8.24140
C	-9.39880	1.16430	-9.00830
C	-8.10800	0.69650	-8.80760
H	-7.15310	2.79800	-6.31740
H	-9.44990	3.63000	-6.67710
H	-10.89010	2.58610	-8.39870
H	-10.02740	0.70690	-9.76140
H	-7.71960	-0.12260	-9.39720
C	1.74650	4.56160	-4.20540
C	1.71150	3.17750	-4.01590

C	2.85810	2.50460	-3.61600
C	4.04180	3.20580	-3.40420
C	4.07960	4.58500	-3.59270
C	2.93630	5.26140	-3.99250
H	0.79220	2.63450	-4.18030
H	2.82860	1.43250	-3.46990
H	4.93440	2.67800	-3.09270
H	4.99970	5.13080	-3.42830
H	2.95230	6.33200	-4.14310
C	-5.24840	7.45240	-1.79630
C	-6.37890	8.21070	-1.52990
C	-7.46280	8.17410	-2.40340
C	-7.41350	7.37850	-3.54450
C	-6.28360	6.61890	-3.81590
H	-4.40020	7.47100	-1.12590
H	-6.41640	8.82970	-0.64280
H	-8.34530	8.76570	-2.19470
H	-8.25630	7.34980	-4.22300
H	-6.24460	6.00090	-4.70100
H	-1.77200	6.17360	-5.00690
H	-2.57540	3.80780	-3.75100
H	-1.42600	1.78420	-5.68520
O	-3.00890	5.82010	-2.44120
O	0.52740	6.51990	-4.83240
O	-5.42930	-0.13720	-8.32280
H	-3.85940	-0.05780	-6.11230
H	-3.31330	1.07730	-7.36420
C	-0.81400	4.96170	-8.84830
H	0.18540	5.13480	-9.24050
H	-1.19450	4.00870	-9.22050
H	-1.47880	5.77080	-9.16420

Methyl α-D-Galf **4**, O4-exo, 180°/+60°

	X	Y	Z
C	-0.57040	2.95410	-7.51360
O	-1.62780	3.72020	-6.92160
C	-0.89650	1.49170	-7.15460
C	-1.78110	1.61630	-5.90990
C	-1.88370	3.13020	-5.65530
H	0.37820	3.24570	-7.03920
O	-0.52860	3.15670	-8.87080
O	-1.21840	1.01270	-4.74050
O	-1.66250	0.87370	-8.18920
C	-1.00240	0.13110	-9.09950
C	-1.32520	-0.32890	-4.62750
C	-3.23520	3.61160	-5.14040
C	-3.59480	2.91960	-3.83630
H	-3.99230	3.37650	-5.89200
O	-3.25990	5.02680	-5.00810
O	-4.93230	3.32170	-3.49560
C	-5.44450	2.80640	-2.36790
H	-1.09900	3.41540	-4.94030
C	-0.80280	-0.82930	-3.33420
C	-6.82980	3.27360	-2.10480
C	-7.47330	4.18880	-2.94190
C	-8.76910	4.59730	-2.65550
C	-9.42850	4.09610	-1.53680
C	-8.78980	3.18410	-0.70050
C	-7.49460	2.77460	-0.98250
H	-6.95950	4.57860	-3.80870
H	-9.26510	5.30700	-3.30510
H	-10.43930	4.41590	-1.31660
H	-9.30180	2.79370	0.16950
H	-6.98720	2.06700	-0.34110
C	-1.91180	-0.41750	-10.13620
C	-3.27180	-0.09940	-10.17170
C	-4.08260	-0.63510	-11.16330
C	-3.54350	-1.48990	-12.12050

C	-2.18830	-1.80860	-12.08780
C	-1.37420	-1.27340	-11.10010
H	-3.68820	0.56550	-9.42920
H	-5.13560	-0.38600	-11.18960
H	-4.17860	-1.90710	-12.89180
H	-1.76830	-2.47330	-12.83160
H	-0.32030	-1.51260	-11.06360
C	-0.76340	-2.21030	-3.12800
C	-0.29010	-2.72080	-1.92830
C	0.14290	-1.85550	-0.92680
C	0.10300	-0.47860	-1.12750
C	-0.36680	0.03670	-2.32780
H	-1.10460	-2.87080	-3.91330
H	-0.25900	-3.79130	-1.77230
H	0.51060	-2.25430	0.01020
H	0.43680	0.19350	-0.34760
H	-0.40130	1.10500	-2.48410
H	0.00400	0.91470	-6.96570
H	-2.75320	1.17170	-6.11830
H	-2.61260	5.28960	-4.33910
O	-1.80100	-1.02110	-5.49940
O	0.19120	-0.06890	-9.05690
O	-4.83050	2.04150	-1.65340
H	-2.91220	3.21400	-3.03630
H	-3.56620	1.83510	-3.93150
C	0.04140	4.41020	-9.25940
H	0.09910	4.40040	-10.34530
H	-0.58630	5.24130	-8.93240
H	1.04490	4.52030	-8.83820

Methyl α-D-Galf **4**, O4-exo, 180°/-60°

	**X**	**Y**	**Z**
C	-1.99200	5.19020	-5.93050
O	-3.33500	4.81220	-6.25640

C	-1.29930	3.86590	-5.57370
C	-2.44630	3.00120	-5.04210
C	-3.71870	3.83590	-5.29480
H	-2.01440	5.84110	-5.04340
O	-1.39410	5.82010	-6.99400
O	-2.34970	2.77320	-3.63240
O	-0.78680	3.23820	-6.75180
C	0.51620	3.42200	-7.04720
C	-1.70420	1.66370	-3.20960
C	-4.91920	3.05640	-5.82290
C	-5.23280	1.82390	-4.99440
H	-4.68430	2.71370	-6.83360
O	-6.05370	3.90560	-5.95660
O	-5.45760	2.24490	-3.63340
C	-5.20910	1.35310	-2.65940
H	-3.99230	4.32620	-4.35280
C	-1.69290	1.56320	-1.73140
C	-5.35370	1.93430	-1.30290
C	-5.55180	3.30240	-1.10340
C	-5.64360	3.80730	0.18610
C	-5.54440	2.95130	1.27910
C	-5.34970	1.58730	1.08230
C	-5.25150	1.08000	-0.20410
H	-5.62010	3.96550	-1.95350
H	-5.78890	4.86880	0.33940
H	-5.61300	3.34820	2.28410
H	-5.26400	0.92270	1.93200
H	-5.08480	0.02500	-0.37080
C	0.89290	2.77530	-8.32880
C	-0.04760	2.13470	-9.13970
C	0.35220	1.54740	-10.33250
C	1.68790	1.59470	-10.72120
C	2.62730	2.23330	-9.91560
C	2.23170	2.82300	-8.72390
H	-1.08420	2.10110	-8.83790

H	-0.37830	1.05290	-10.95960
H	1.99680	1.13500	-11.65160
H	3.66610	2.27070	-10.21740
H	2.95080	3.32280	-8.08960
C	-1.33270	0.34510	-1.15210
C	-1.31820	0.21090	0.22850
C	-1.64900	1.29560	1.03630
C	-2.00080	2.51270	0.46210
C	-2.03130	2.64750	-0.91790
H	-1.07700	-0.48890	-1.79110
H	-1.05010	-0.73770	0.67540
H	-1.63870	1.18960	2.11370
H	-2.26700	3.35270	1.08940
H	-2.31760	3.58750	-1.36520
H	-0.50480	3.99840	-4.84490
H	-2.45150	2.05000	-5.56790
H	-6.32040	4.20150	-5.07570
O	-1.21110	0.85880	-3.96720
O	1.28040	4.03370	-6.33500
O	-4.88910	0.20470	-2.88180
H	-4.42250	1.09890	-5.02320
H	-6.13960	1.34890	-5.36960
C	-1.82700	7.16850	-7.19680
H	-1.21260	7.57390	-7.99720
H	-2.87840	7.20030	-7.48890
H	-1.68140	7.75730	-6.28640

Methyl α-D-Galf **4**, O4-exo, 180°/180°

	**X**	**Y**	**Z**
C	-2.15890	1.11000	-8.84100
O	-2.80960	1.81250	-7.77560
C	-2.50570	-0.36990	-8.60230
C	-2.83450	-0.42720	-7.10550
C	-2.63330	1.01800	-6.60990

H	-1.07300	1.25350	-8.73930
O	-2.60510	1.55880	-10.06010
O	-1.97210	-1.28670	-6.35450
O	-3.67530	-0.73860	-9.33340
C	-3.50620	-1.36480	-10.51250
C	-2.15660	-2.61460	-6.51340
C	-3.59760	1.50020	-5.53120
C	-3.43120	0.76910	-4.20340
H	-4.62110	1.37920	-5.89210
O	-3.43240	2.89390	-5.29490
O	-3.94740	-0.56640	-4.32960
C	-3.61570	-1.43470	-3.35870
H	-1.60250	1.11700	-6.23900
C	-1.38850	-3.41700	-5.53410
C	-4.12460	-2.80330	-3.61790
C	-4.96110	-3.08890	-4.69870
C	-5.38040	-4.39270	-4.92340
C	-4.96520	-5.41480	-4.07590
C	-4.13300	-5.13210	-2.99580
C	-3.71650	-3.83090	-2.76480
H	-5.27020	-2.29700	-5.36480
H	-6.02320	-4.61260	-5.76590
H	-5.28580	-6.43260	-4.25940
H	-3.80180	-5.92860	-2.34230
H	-3.05880	-3.59960	-1.93890
C	-4.80040	-1.66700	-11.17410
C	-6.02180	-1.25680	-10.63370
C	-7.20660	-1.55880	-11.29160
C	-7.17990	-2.27130	-12.48710
C	-5.96420	-2.68190	-13.02780
C	-4.77800	-2.37980	-12.37490
H	-6.04080	-0.70230	-9.70690
H	-8.15140	-1.23820	-10.87180
H	-8.10560	-2.50600	-12.99740
H	-5.94250	-3.23600	-13.95730

H	-3.82710	-2.69140	-12.78470
C	-1.51020	-4.80670	-5.58230
C	-0.85300	-5.59440	-4.64990
C	-0.07190	-4.99900	-3.66360
C	0.05440	-3.61360	-3.61380
C	-0.60040	-2.82180	-4.54600
H	-2.13350	-5.25480	-6.34330
H	-0.95720	-6.67110	-4.68430
H	0.43530	-5.61430	-2.93110
H	0.65740	-3.15040	-2.84360
H	-0.51570	-1.74610	-4.49990
H	-1.68090	-1.02220	-8.87070
H	-3.86420	-0.75500	-6.97940
H	-2.51120	3.06640	-5.05710
O	-2.89100	-3.07220	-7.36030
O	-2.42050	-1.65280	-10.96610
O	-2.95490	-1.11910	-2.39190
H	-3.99320	1.30040	-3.43550
H	-2.38210	0.72880	-3.90690
C	-2.07090	2.82790	-10.44790
H	-2.42620	3.01810	-11.45790
H	-2.42060	3.61820	-9.78080
H	-0.97730	2.80020	-10.44150

Methyl α-D-Galf **4**, C2-endo, +60°/+60°

	**X**	**Y**	**Z**
C	3.90410	3.24250	-0.90770
H	3.40940	4.17820	-1.16350
C	3.07220	2.49170	0.12850
H	3.69690	1.79570	0.68070
C	2.01100	1.80350	-0.73750
C	1.61200	0.39150	-0.31420
H	0.84120	0.04650	-1.01370
O	1.06430	0.52230	0.99400

C	3.85630	2.30130	-2.10730
O	4.80630	1.29460	-1.94090
H	1.10610	2.41410	-0.72080
H	3.99050	2.80260	-3.06860
O	5.24270	3.49350	-0.49840
O	2.42090	3.37110	1.05030
O	2.52460	1.80640	-2.08280
C	2.74820	-0.61220	-0.34060
H	3.54910	-0.34860	0.34880
H	3.15580	-0.72120	-1.34170
O	2.16520	-1.86450	0.07930
H	0.80590	-0.35660	1.29970
C	5.50740	4.67320	0.10250
C	3.04120	3.62780	2.21910
C	2.99090	-2.91980	0.16270
C	2.30310	-4.14860	0.63540
C	0.94380	-4.15930	0.95890
C	0.34550	-5.33210	1.40000
C	1.09730	-6.49740	1.52020
C	2.45200	-6.49020	1.19840
C	3.05350	-5.32020	0.75760
O	4.16780	-2.85750	-0.12250
C	6.92380	4.77650	0.52690
C	7.33550	5.94520	1.17150
C	8.65060	6.08180	1.59100
C	9.56170	5.05230	1.36940
C	9.15490	3.88540	0.72850
C	7.83950	3.74410	0.30750
O	4.67350	5.53510	0.26950
C	2.26820	4.56370	3.07090
O	4.10940	3.14460	2.52450
C	2.81700	4.94400	4.29780
C	2.13120	5.82100	5.12530
C	0.89300	6.32310	4.73290
C	0.34270	5.94690	3.51090

C	1.02640	5.07030	2.67910
H	0.36040	-3.25490	0.86540
H	-0.70750	-5.33730	1.65040
H	0.62800	-7.41040	1.86460
H	3.03750	-7.39560	1.29180
H	4.10470	-5.30100	0.50530
H	6.61720	6.73590	1.33890
H	8.96610	6.98840	2.09090
H	10.58810	5.15880	1.69740
H	9.86280	3.08470	0.55810
H	7.52070	2.83860	-0.18770
H	3.77980	4.54790	4.58970
H	2.56030	6.11430	6.07460
H	0.35780	7.00770	5.37870
H	-0.61950	6.33740	3.20560
H	0.60120	4.77880	1.72990
C	4.99290	0.48150	-3.09930
H	5.76500	-0.24340	-2.85210
H	4.07320	-0.04420	-3.36660
H	5.31840	1.09320	-3.94610

Methyl α-D-Gal*f* **4**, C2-endo, +60°/-60°

	**X**	**Y**	**Z**
C	3.83550	3.07470	-0.67920
H	3.48980	4.09160	-0.86060
C	2.90410	2.39340	0.32430
H	3.45960	1.73070	0.97130
C	1.89020	1.64780	-0.55300
C	1.63440	0.18250	-0.17980
H	0.85590	-0.18050	-0.86270
O	1.14210	0.22820	1.15670
C	3.68000	2.24640	-1.95980
O	4.61820	1.21700	-1.97640
H	0.93000	2.16020	-0.47950

H	3.76290	2.84740	-2.86910
O	5.20290	3.08810	-0.27980
O	2.21050	3.35920	1.12630
O	2.34350	1.77410	-1.90950
C	2.78950	-0.78730	-0.34640
H	3.13160	-0.79450	-1.37740
H	2.45710	-1.79440	-0.08390
O	3.87350	-0.41970	0.52310
H	0.86500	-0.65810	1.42010
C	5.61060	4.03800	0.58180
C	2.54520	3.46350	2.42620
C	5.08290	-0.93370	0.25610
C	4.70970	0.54190	-3.22940
H	5.49110	-0.20610	-3.12340
H	3.76830	0.04860	-3.48350
H	4.97430	1.24620	-4.02420
C	6.13790	-0.40240	1.15760
C	5.85130	0.49600	2.18800
C	6.87220	0.96670	3.00260
C	8.18050	0.54320	2.79860
C	8.47060	-0.35320	1.77370
C	7.45470	-0.82360	0.95530
O	5.27780	-1.74660	-0.62340
C	7.05140	3.90560	0.90490
C	7.56920	4.67710	1.94710
C	8.91010	4.57420	2.28730
C	9.74210	3.70970	1.58060
C	9.22910	2.94250	0.53950
C	7.88620	3.03250	0.20420
O	4.87380	4.88630	1.03460
C	1.80920	4.55790	3.10700
O	3.35040	2.74150	2.97260
C	2.02210	4.74480	4.47470
C	1.36290	5.76150	5.15010
C	0.48820	6.59970	4.46340

C	0.27440	6.41810	3.10000
C	0.93130	5.40070	2.42090
H	4.83980	0.83270	2.35500
H	6.64520	1.67220	3.79110
H	8.97560	0.91980	3.42890
H	9.48990	-0.67760	1.60870
H	7.66860	-1.51720	0.15380
H	6.91140	5.34480	2.48640
H	9.30740	5.16630	3.10160
H	10.78880	3.62960	1.84620
H	9.87310	2.26220	-0.00190
H	7.48160	2.42320	-0.59030
H	2.70540	4.08860	4.99580
H	1.53060	5.90230	6.21010
H	-0.02530	7.39420	4.99020
H	-0.40390	7.07060	2.56540
H	0.76880	5.26080	1.36230

Methyl α-D-Galf **4**, C2-endo, +60°/180°

	**X**	**Y**	**Z**
C	4.22820	3.31760	-0.79990
H	3.69700	4.21450	-1.11480
C	3.35370	2.52100	0.16180
H	3.96620	1.86470	0.77440
C	2.42450	1.76200	-0.79100
C	2.05850	0.34310	-0.35140
H	1.32840	-0.05140	-1.06580
O	1.46640	0.50650	0.93480
C	4.36300	2.36690	-1.98600
O	5.37650	1.44390	-1.73440
H	1.49490	2.32710	-0.88690
H	4.53690	2.87730	-2.93610
O	5.50040	3.66690	-0.26780
O	2.57010	3.36350	1.01180

O	3.08040	1.76010	-2.07120
C	3.21820	-0.64680	-0.27330
H	2.97500	-1.42670	0.44980
H	4.15690	-0.18190	0.02260
O	3.37420	-1.24970	-1.57100
H	1.05120	-0.32530	1.19440
C	5.62510	4.86980	0.33140
C	3.05700	3.64850	2.23500
C	4.34930	-2.15890	-1.69930
C	4.42820	-2.72120	-3.07250
C	3.53880	-2.33940	-4.08020
C	3.65600	-2.88440	-5.35170
C	4.65910	-3.81010	-5.62510
C	5.54710	-4.19260	-4.62310
C	5.43210	-3.65080	-3.35090
O	5.08350	-2.47530	-0.78620
C	6.98780	5.08310	0.87450
C	7.25330	6.28440	1.53570
C	8.51400	6.52510	2.06190
C	9.51660	5.56760	1.93070
C	9.25540	4.36840	1.27380
C	7.99480	4.12290	0.74650
O	4.72040	5.67110	0.40860
C	2.14680	4.52420	3.01220
O	4.11990	3.23240	2.64160
C	2.55910	4.94260	4.27940
C	1.74090	5.76310	5.04200
C	0.50560	6.16970	4.54410
C	0.09100	5.75500	3.28170
C	0.90780	4.93520	2.51450
H	2.76390	-1.61730	-3.86790
H	2.96570	-2.58630	-6.13030
H	4.74910	-4.23250	-6.61790
H	6.32800	-4.91150	-4.83470
H	6.11610	-3.93880	-2.56460

H	6.46520	7.01860	1.63140
H	8.71610	7.45690	2.57410
H	10.50060	5.75550	2.34140
H	10.03440	3.62370	1.17350
H	7.78910	3.19210	0.23830
H	3.52100	4.62020	4.65330
H	2.06460	6.08640	6.02290
H	-0.13310	6.80990	5.13940
H	-0.86920	6.07100	2.89480
H	0.58770	4.61300	1.53440
C	5.78580	0.71490	-2.89200
H	6.51930	-0.01660	-2.56060
H	4.94160	0.19930	-3.35080
H	6.24250	1.38800	-3.62380

Methyl α-D-Galf **4**, C2-endo, -60°/+60°

	**X**	**Y**	**Z**
C	3.85400	3.42040	-0.95720
H	3.44360	4.40670	-1.16520
C	2.97530	2.69570	0.04720
H	3.51220	1.87710	0.51810
C	1.82460	2.19140	-0.83060
C	1.24700	0.84700	-0.41900
H	0.79570	0.98750	0.57020
O	2.29230	-0.11310	-0.34450
C	3.71640	2.54170	-2.19770
O	4.58730	1.45980	-2.09310
H	1.01590	2.92690	-0.80350
H	3.87870	3.07290	-3.13980
O	5.20010	3.53880	-0.51680
O	2.53890	3.62550	1.04330
O	2.34850	2.16080	-2.17500
C	0.16580	0.40900	-1.38800
H	0.57740	0.21990	-2.37820

H	-0.62600	1.15580	-1.46000
O	-0.37760	-0.81620	-0.85530
H	1.91580	-0.93790	-0.01360
C	5.63760	4.74820	-0.10320
C	2.29630	3.16290	2.28620
C	-1.32800	-1.42830	-1.57820
C	-1.79250	-2.69520	-0.95650
C	-1.25420	-3.17460	0.24050
C	-1.71870	-4.36610	0.78140
C	-2.72030	-5.08360	0.13380
C	-3.25910	-4.60860	-1.05910
C	-2.79700	-3.41880	-1.60310
O	-1.75070	-0.98120	-2.62330
C	7.05050	4.70040	0.34720
C	7.62870	5.87600	0.83190
C	8.94700	5.87660	1.26350
C	9.69580	4.70360	1.21300
C	9.12360	3.53010	0.73000
C	7.80420	3.52460	0.29800
O	4.95040	5.74480	-0.10520
C	1.97030	4.25620	3.23440
O	2.34390	1.98830	2.57790
C	1.57840	3.91180	4.53010
C	1.27340	4.90320	5.45120
C	1.36190	6.24390	5.08500
C	1.75510	6.59110	3.79570
C	2.05750	5.60230	2.86920
H	-0.47740	-2.61690	0.74330
H	-1.29950	-4.73530	1.70850
H	-3.08080	-6.01230	0.55800
H	-4.03790	-5.16580	-1.56350
H	-3.20650	-3.03930	-2.52910
H	7.03630	6.78000	0.86620
H	9.39130	6.78910	1.63930
H	10.72480	4.70430	1.54990

H	9.70600	2.61870	0.69030
H	7.35850	2.61530	-0.07790
H	1.51610	2.86720	4.80230
H	0.96760	4.63280	6.45360
H	1.12550	7.01750	5.80470
H	1.82730	7.63320	3.51220
H	2.36670	5.87030	1.86950
C	4.56100	0.59350	-3.22570
H	5.34890	-0.14150	-3.07650
H	3.59810	0.08550	-3.30700
H	4.75400	1.15660	-4.14500

Methyl α-D-Galf **4**, C2-endo, -60°/-60°

	**X**	**Y**	**Z**
C	3.89310	3.22590	-1.02240
H	3.38880	4.15520	-1.28020
C	3.08990	2.46740	0.01770
H	3.70100	1.71450	0.50770
C	1.97970	1.83160	-0.82760
C	1.49570	0.48610	-0.29980
H	1.16590	0.68190	0.72830
O	2.58340	-0.42830	-0.30040
C	3.84010	2.27090	-2.21120
O	4.81260	1.28530	-2.04710
H	1.12190	2.51020	-0.84370
H	3.95430	2.75920	-3.18320
O	5.22120	3.49700	-0.59390
O	2.58260	3.38590	0.99020
O	2.51730	1.76170	-2.16370
C	0.27350	-0.06620	-1.01310
H	-0.55460	0.64240	-0.94830
H	-0.03650	-1.00250	-0.54650
O	0.58890	-0.30780	-2.39310
H	2.35630	-1.17470	0.26750

C	5.54010	4.76440	-0.25130
C	2.45560	2.96410	2.26480
C	-0.36420	-0.85470	-3.15550
C	0.06810	-1.00050	-4.57010
C	1.26560	-0.44500	-5.02840
C	1.63360	-0.59560	-6.35870
C	0.81350	-1.30150	-7.23470
C	-0.38120	-1.85450	-6.78040
C	-0.75510	-1.70220	-5.45260
O	-1.45160	-1.18730	-2.72970
C	6.94640	4.87530	0.20910
C	7.40940	6.12770	0.61950
C	8.71680	6.27660	1.05870
C	9.56950	5.17600	1.09020
C	9.11200	3.92650	0.68170
C	7.80410	3.77260	0.24230
O	4.76390	5.69120	-0.31730
C	2.06070	4.06350	3.17870
O	2.64520	1.81760	2.60730
C	1.75210	3.74350	4.50300
C	1.38960	4.74310	5.39370
C	1.33820	6.06830	4.96860
C	1.64850	6.39160	3.65080
C	2.00700	5.39390	2.75430
H	1.89350	0.10710	-4.34460
H	2.56000	-0.16180	-6.71280
H	1.10390	-1.41960	-8.27110
H	-1.01930	-2.40290	-7.46130
H	-1.68110	-2.12510	-5.08750
H	6.73700	6.97400	0.59090
H	9.07180	7.24830	1.37660
H	10.58990	5.29230	1.43310
H	9.77500	3.07130	0.70590
H	7.44740	2.80370	-0.07540
H	1.79900	2.71100	4.82090

H	1.14830	4.49130	6.41830
H	1.05750	6.84870	5.66470
H	1.61220	7.42200	3.32160
H	2.25280	5.64360	1.73250
C	4.85750	0.34090	-3.11430
H	5.73730	-0.27740	-2.94960
H	3.96520	-0.28730	-3.11750
H	4.94440	0.85260	-4.07870

Methyl α-D-Galf **4**, C2-endo, -60°/180°

	**X**	**Y**	**Z**
C	3.94920	3.34720	-1.03020
H	3.46550	4.29300	-1.26670
C	3.11170	2.57360	-0.02750
H	3.70260	1.80860	0.46840
C	2.02120	1.96490	-0.91440
C	1.54200	0.58540	-0.48870
H	1.06730	0.70450	0.49010
O	2.65400	-0.29650	-0.39950
C	3.90570	2.43630	-2.25420
O	4.85840	1.43000	-2.11030
H	1.15820	2.63390	-0.91970
H	4.04490	2.96020	-3.20410
O	5.27300	3.58220	-0.56810
O	2.58620	3.48440	0.94380
O	2.57320	1.94760	-2.24870
C	0.53160	0.00250	-1.46770
H	0.21950	-0.98990	-1.14070
H	0.95780	-0.06860	-2.46730
O	-0.60760	0.88250	-1.48350
H	2.42020	-1.02630	0.18650
C	5.61000	4.83470	-0.18990
C	2.34670	3.02350	2.18740
C	-1.60540	0.57660	-2.32560

C	-2.71540	1.56300	-2.27420
C	-2.67220	2.68160	-1.43780
C	-3.73350	3.57680	-1.42260
C	-4.84010	3.36230	-2.23930
C	-4.88580	2.24890	-3.07440
C	-3.82720	1.35220	-3.09260
O	-1.58780	-0.40170	-3.04250
C	7.00990	4.90750	0.29660
C	7.48960	6.14080	0.74420
C	8.79200	6.25420	1.20790
C	9.62310	5.13710	1.22720
C	9.14910	3.90640	0.78190
C	7.84600	3.78800	0.31760
O	4.85270	5.77770	-0.24620
C	1.91850	4.10390	3.10960
O	2.47160	1.85960	2.49970
C	1.52360	3.75190	4.40230
C	1.12540	4.73220	5.29940
C	1.12300	6.06980	4.91210
C	1.51840	6.42500	3.62560
C	1.91400	5.44690	2.72310
H	-1.81280	2.84710	-0.80430
H	-3.69740	4.44250	-0.77390
H	-5.66620	4.06200	-2.22520
H	-5.74580	2.08130	-3.70990
H	-3.84990	0.48410	-3.73690
H	6.83390	7.00040	0.72450
H	9.15990	7.21130	1.55440
H	10.63970	5.22570	1.58920
H	9.79530	3.03830	0.79660
H	7.47670	2.83400	-0.02900
H	1.53240	2.70990	4.69130
H	0.81800	4.45550	6.29960
H	0.81410	6.83500	5.61310
H	1.51980	7.46500	3.32570

H	2.22540	5.72150	1.72580
C	4.92640	0.54410	-3.22590
H	5.76280	-0.12690	-3.04300
H	4.00660	-0.03610	-3.32130
H	5.10070	1.10370	-4.15100

Methyl α-D-Galf **4**, C2-endo, 180°/+60°

	**X**	**Y**	**Z**
C	4.17650	3.49920	-0.90310
H	3.83860	4.49460	-1.18640
C	3.35630	2.98710	0.27770
H	3.90870	2.20440	0.79320
C	2.08270	2.46270	-0.39730
C	1.59050	1.13740	0.16510
H	2.37780	0.38550	0.03720
O	0.43420	0.77010	-0.57840
C	3.79280	2.50310	-1.99230
O	4.50910	1.31990	-1.79610
H	1.28590	3.20450	-0.30840
H	3.92910	2.87480	-3.01110
O	5.57890	3.50500	-0.66800
O	2.99660	4.00880	1.21280
O	2.40030	2.34000	-1.79720
C	1.25780	1.26940	1.64160
H	0.47740	2.01410	1.80080
H	2.13060	1.53050	2.23920
O	0.77590	-0.02350	2.05720
H	0.09620	-0.05710	-0.21190
C	6.13220	4.64370	-0.19930
C	3.81380	4.21040	2.26940
C	0.32210	-0.12580	3.31800
C	-0.16830	-1.49010	3.63960
C	-0.14290	-2.52680	2.70290
C	-0.61280	-3.78640	3.05030

C	-1.10920	-4.01820	4.32990
C	-1.13610	-2.98720	5.26550
C	-0.66730	-1.72740	4.92240
O	0.32310	0.80620	4.09310
C	7.58370	4.49380	0.06020
C	8.27930	5.59380	0.56730
C	9.63710	5.49580	0.83320
C	10.30790	4.29910	0.59390
C	9.61800	3.20040	0.08940
C	8.25870	3.29370	-0.17730
O	5.50130	5.66070	-0.01500
C	3.31880	5.28140	3.16570
O	4.82900	3.57580	2.45000
C	4.09980	5.62670	4.27120
C	3.67520	6.62180	5.13920
C	2.46780	7.27650	4.90930
C	1.68670	6.93520	3.80890
C	2.10890	5.94140	2.93620
H	0.24330	-2.34710	1.71010
H	-0.59180	-4.58760	2.32290
H	-1.47490	-5.00150	4.59790
H	-1.52200	-3.16660	6.26070
H	-0.68200	-0.91860	5.63990
H	7.74530	6.51600	0.75050
H	10.17290	6.34970	1.22720
H	11.36770	4.22260	0.80200
H	10.13950	2.27010	-0.09500
H	7.72070	2.44160	-0.56620
H	5.03480	5.10990	4.43780
H	4.28340	6.88750	5.99410
H	2.13610	8.05250	5.58760
H	0.74820	7.44400	3.63090
H	1.50390	5.67610	2.08160
C	4.25260	0.32550	-2.78730
H	4.92450	-0.50420	-2.57970

H	3.21790	-0.01970	-2.73700
H	4.45490	0.72030	-3.78810

Methyl α-D-Galf **4**, C2-endo, 180°/-60°

	**X**	**Y**	**Z**
C	4.07640	3.52010	-1.05160
H	3.66940	4.50790	-1.26050
C	3.23180	2.82210	0.01010
H	3.83110	2.06710	0.51290
C	2.07550	2.22670	-0.80390
C	1.72020	0.78970	-0.43700
H	2.56800	0.14680	-0.69970
O	0.57660	0.44910	-1.21610
C	3.86000	2.61660	-2.26100
O	4.67140	1.48560	-2.13230
H	1.18880	2.84960	-0.68670
H	4.02670	3.10560	-3.22410
O	5.45380	3.62020	-0.70900
O	2.69520	3.72630	0.98030
O	2.48030	2.31470	-2.18610
C	1.47230	0.60460	1.05080
H	2.38740	0.73840	1.62420
H	1.09330	-0.40100	1.24300
O	0.48680	1.56060	1.48470
H	0.41850	-0.49960	-1.13320
C	5.86490	4.73990	-0.07680
C	3.36080	3.88440	2.14490
C	0.53970	1.96930	2.76270
C	-0.44580	3.03740	3.05980
C	-1.23230	3.61450	2.06030
C	-2.11750	4.63480	2.37950
C	-2.22450	5.08000	3.69380
C	-1.44250	4.50530	4.69130
C	-0.55420	3.48890	4.37590

O	1.32190	1.51660	3.57220
C	7.30890	4.69500	0.25510
C	7.86560	5.78700	0.92480
C	9.21170	5.78520	1.25980
C	10.00920	4.69290	0.92800
C	9.45790	3.60220	0.26150
C	8.11100	3.59950	-0.07520
O	5.12670	5.66380	0.18380
C	2.69670	4.86960	3.02900
O	4.37060	3.27560	2.41980
C	3.11720	4.95590	4.35730
C	2.51600	5.86230	5.21820
C	1.50110	6.69430	4.75360
C	1.08390	6.61460	3.42890
C	1.67410	5.70180	2.56750
H	-1.14190	3.27090	1.04010
H	-2.72170	5.08550	1.60270
H	-2.91270	5.87910	3.93930
H	-1.51790	4.85810	5.71160
H	0.07020	3.04480	5.13840
H	7.23440	6.62770	1.17770
H	9.63960	6.63270	1.77930
H	11.05980	4.69140	1.18990
H	10.07790	2.75290	0.00510
H	7.68050	2.75340	-0.59070
H	3.90680	4.30380	4.70430
H	2.83670	5.92030	6.25030
H	1.03060	7.40010	5.42640
H	0.28870	7.25440	3.07050
H	1.34370	5.63030	1.54220
C	4.58240	0.59110	-3.24080
H	5.30860	-0.19930	-3.06490
H	3.58210	0.15940	-3.31640
H	4.82410	1.11090	-4.17340

Methyl α-D-Galf **4**, C2-endo, 180°/180°

	**X**	**Y**	**Z**
C	4.33510	3.51550	-1.05490
H	3.93700	4.47150	-1.39090
C	3.49430	2.97850	0.10180
H	4.05050	2.21100	0.63520
C	2.26970	2.41310	-0.63000
C	1.68970	1.13590	-0.03480
H	2.48080	0.38240	0.04360
O	0.66960	0.70770	-0.93330
C	4.08140	2.45550	-2.12000
O	4.87390	1.33700	-1.84510
H	1.48330	3.17250	-0.65660
H	4.24430	2.79990	-3.14470
O	5.72020	3.63600	-0.76540
O	3.05240	3.96840	1.03480
O	2.69910	2.18690	-1.98680
C	1.03840	1.35830	1.32730
H	0.41320	0.49800	1.57140
H	0.40970	2.24900	1.31410
O	2.05240	1.49610	2.33840
H	0.37790	-0.17400	-0.66930
C	6.17760	4.84060	-0.36660
C	3.85790	4.22710	2.08690
C	1.61430	1.82500	3.56780
C	2.71240	1.96520	4.55340
C	4.02140	1.57680	4.26330
C	5.01980	1.74840	5.21190
C	4.71850	2.31150	6.44820
C	3.41290	2.69770	6.74060
C	2.41190	2.52160	5.79840
O	0.44040	1.99590	3.82000
C	7.61690	4.80030	-0.01280
C	8.22410	5.98150	0.41900

C	9.56580	5.98620	0.77140
C	10.30890	4.81100	0.69470
C	9.70740	3.63140	0.26520
C	8.36460	3.62240	-0.08760
O	5.48380	5.83150	-0.31230
C	3.18070	5.01660	3.14000
O	4.99800	3.82800	2.15690
C	3.94040	5.44710	4.22950
C	3.33450	6.13080	5.27150
C	1.96560	6.38310	5.23480
C	1.20510	5.95680	4.14990
C	1.80920	5.27780	3.10090
H	4.25620	1.15370	3.29810
H	6.03490	1.45050	4.98350
H	5.50140	2.45250	7.18280
H	3.17970	3.14350	7.69870
H	1.39660	2.82800	6.00730
H	7.63490	6.88630	0.47670
H	10.03260	6.90330	1.10660
H	11.35600	4.81440	0.97060
H	10.28490	2.71790	0.20670
H	7.89450	2.70770	-0.41810
H	4.99810	5.22550	4.25390
H	3.92430	6.45700	6.11810
H	1.49070	6.90830	6.05400
H	0.14010	6.14760	4.12470
H	1.21940	4.93380	2.26400
C	4.75400	0.30060	-2.81830
H	5.46450	-0.47470	-2.54010
H	3.74380	-0.11430	-2.82640
H	4.99760	0.68040	-3.81580

Methyl α-D-Galf **4**, C1-exo, +60°/+60°

	X	Y	Z
C	4.37890	2.26780	-1.08090
H	4.18580	3.26770	-1.46650
C	3.33020	1.91440	-0.02990
H	3.70880	1.14150	0.63260
C	2.14510	1.45840	-0.88820
C	1.33300	0.28320	-0.34860
H	0.53810	0.07740	-1.07530
O	0.77080	0.73320	0.88060
C	4.10200	1.24140	-2.17520
O	4.71610	0.03460	-1.84260
H	1.45860	2.30000	-0.99970
H	4.40750	1.56660	-3.17270
O	5.71750	2.17670	-0.60930
O	2.93160	3.04720	0.74830
O	2.68460	1.14920	-2.18720
C	2.13050	-0.99110	-0.15560
H	2.91370	-0.87690	0.59230
H	2.57070	-1.32950	-1.08950
O	1.18030	-1.97320	0.31020
H	0.25300	0.00950	1.25590
C	6.29200	3.29680	-0.12210
C	3.55970	3.25510	1.92260
C	1.65950	-3.19680	0.58490
C	7.66090	3.04410	0.38700
C	8.23340	1.76960	0.36950
C	9.51580	1.58070	0.86670
C	10.23090	2.65820	1.38190
C	9.66230	3.92930	1.40120
C	8.38100	4.12250	0.90630
O	5.73960	4.37430	-0.10750
C	0.60800	-4.12800	1.06810
C	-0.72490	-3.73270	1.20720
C	-1.67310	-4.63960	1.66160
C	-1.29810	-5.94200	1.97910

C	0.02950	-6.33900	1.84180
C	0.97970	-5.43580	1.38790
O	2.82730	-3.49200	0.44520
C	3.06660	4.46990	2.61540
C	2.03260	5.25260	2.09560
C	1.60800	6.38260	2.78140
C	2.21230	6.73810	3.98400
C	3.24440	5.96090	4.50350
C	3.67030	4.83000	3.82240
O	4.42810	2.52630	2.34980
H	7.67510	0.93480	-0.02850
H	9.95740	0.59270	0.85370
H	11.23060	2.50750	1.76950
H	10.21770	4.76690	1.80280
H	7.92610	5.10340	0.91730
H	-1.01580	-2.72200	0.96000
H	-2.70490	-4.33070	1.76830
H	-2.03960	-6.64700	2.33310
H	0.32210	-7.35140	2.08840
H	2.01380	-5.73150	1.27650
H	1.56570	4.97630	1.16150
H	0.80590	6.98680	2.37760
H	1.87970	7.62070	4.51570
H	3.71580	6.23780	5.43760
H	4.47130	4.21830	4.21390
C	4.68790	-0.93410	-2.89050
H	5.21300	-1.81150	-2.52000
H	3.66230	-1.20700	-3.14990
H	5.19520	-0.54910	-3.78030

Methyl α-D-Galf **4**, C1-exo, +60°/-60°

	**X**	**Y**	**Z**
C	4.32220	2.40590	-1.00300
H	4.29870	3.45120	-1.30860

C	3.21470	2.15050	0.02000
H	3.53860	1.43540	0.76160
C	2.03960	1.63590	-0.82070
C	1.35810	0.36240	-0.30530
H	0.52020	0.16210	-0.98530
O	0.87560	0.70590	0.99070
C	3.94750	1.51710	-2.19460
O	4.54070	0.26440	-2.05720
H	1.27200	2.41050	-0.85000
H	4.21770	1.95860	-3.15730
O	5.62420	2.06230	-0.53840
O	2.82510	3.36390	0.67810
O	2.53060	1.46410	-2.15890
C	2.18000	-0.91300	-0.30690
H	2.52000	-1.14210	-1.31280
H	1.56190	-1.74160	0.04670
O	3.31180	-0.77690	0.56900
H	0.36160	-0.03220	1.34140
C	6.29370	2.95440	0.21480
C	3.17460	3.52600	1.96820
C	4.31840	-1.65020	0.41620
C	4.44810	-0.54790	-3.22610
H	4.96850	-1.47560	-3.00190
H	3.40690	-0.76770	-3.47430
H	4.92470	-0.04970	-4.07600
C	7.62490	2.44200	0.61870
C	8.16660	1.28210	0.06100
C	9.41760	0.84020	0.46540
C	10.12640	1.54210	1.43500
C	9.58480	2.69410	1.99960
C	8.34000	3.14750	1.58840
O	5.84480	4.03700	0.52110
C	5.47210	-1.34990	1.30290
C	5.45300	-0.29300	2.21590
C	6.55830	-0.05090	3.02040

C	7.68420	-0.86040	2.92340
C	7.70700	-1.91600	2.01600
C	6.60680	-2.15960	1.20780
O	4.27470	-2.58140	-0.36030
C	2.76280	4.85020	2.49720
C	2.10980	5.79620	1.70320
C	1.74930	7.02380	2.24260
C	2.03790	7.31410	3.57290
C	2.68960	6.37380	4.36680
C	3.05120	5.14620	3.83140
O	3.75290	2.68240	2.61770
H	7.60600	0.72820	-0.67740
H	9.83370	-0.06100	0.03480
H	11.09830	1.18870	1.75600
H	10.13340	3.23710	2.75840
H	7.90760	4.04120	2.01720
H	4.58580	0.34370	2.29920
H	6.54160	0.77730	3.71670
H	8.54800	-0.66250	3.54480
H	8.58600	-2.54220	1.93350
H	6.61430	-2.97370	0.49620
H	1.88840	5.57070	0.67030
H	1.24370	7.75480	1.62490
H	1.75590	8.27250	3.99060
H	2.91530	6.59900	5.40110
H	3.55840	4.40800	4.43710

Methyl α-D-Galf **4**, C1-exo, +60°/180°

	**X**	**Y**	**Z**
C	4.68740	2.54220	-1.04890
H	4.59690	3.58090	-1.36260
C	3.48190	2.16430	-0.19500
H	3.72100	1.32070	0.44760
C	2.41780	1.83800	-1.24840

C	1.49870	0.66590	-0.89910
H	0.73490	0.59340	-1.68010
O	0.91040	1.02920	0.34800
C	4.53310	1.63370	-2.26540
O	5.06460	0.37620	-1.98530
H	1.78910	2.71990	-1.38730
H	4.98050	2.04440	-3.17340
O	5.93680	2.33690	-0.40050
O	3.02460	3.25520	0.61080
O	3.12790	1.59950	-2.47620
C	2.17760	-0.69660	-0.77970
H	1.59640	-1.32740	-0.10580
H	3.19660	-0.63330	-0.40220
O	2.19190	-1.30310	-2.08500
H	0.17100	0.43540	0.52830
C	6.48960	3.38290	0.24940
C	3.48870	3.34300	1.87240
C	2.75130	-2.51660	-2.17760
C	7.74770	3.00820	0.93780
C	8.25260	1.70580	0.89950
C	9.43000	1.40070	1.56880
C	10.10710	2.38910	2.27750
C	9.60540	3.68760	2.31810
C	8.42930	3.99700	1.65100
O	6.00120	4.49100	0.26260
C	2.71500	-3.05810	-3.56080
C	2.12190	-2.35720	-4.61380
C	2.11900	-2.89880	-5.89230
C	2.70730	-4.13840	-6.12700
C	3.29970	-4.83900	-5.07970
C	3.30330	-4.30140	-3.80050
O	3.23570	-3.09650	-1.22780
C	2.95530	4.52910	2.58520
C	2.05170	5.41060	1.98640
C	1.58490	6.51000	2.69400

C	2.01650	6.73630	3.99790
C	2.91820	5.86020	4.59690
C	3.38620	4.75990	3.89360
O	4.25700	2.54060	2.35600
H	7.72360	0.94000	0.35120
H	9.81920	0.39110	1.53900
H	11.02450	2.14760	2.79930
H	10.13080	4.45600	2.87050
H	8.02690	5.00030	1.67660
H	1.67030	-1.39310	-4.43050
H	1.65900	-2.35350	-6.70640
H	2.70490	-4.55770	-7.12520
H	3.75820	-5.80250	-5.26140
H	3.76130	-4.83460	-2.97880
H	1.71890	5.23460	0.97390
H	0.88450	7.19130	2.22830
H	1.65140	7.59530	4.54670
H	3.25540	6.03630	5.61020
H	4.08750	4.07250	4.34600
C	5.26840	-0.42930	-3.14690
H	5.62070	-1.39820	-2.80060
H	4.34000	-0.55870	-3.70400
H	6.02260	0.02510	-3.79640

Methyl α-D-Galf **4**, C1-exo, -60°/+60°

	**X**	**Y**	**Z**
C	4.45420	2.10680	-1.12060
H	4.20800	3.08350	-1.53510
C	3.40590	1.70550	-0.08890
H	3.78490	0.91240	0.54930
C	2.25290	1.23300	-0.97410
C	1.41670	0.11630	-0.37210
H	0.92300	0.53870	0.51330
O	2.26300	-0.95960	0.00890

C	4.26140	1.03420	-2.19110
O	4.94900	-0.11720	-1.81380
H	1.58750	2.07920	-1.16680
H	4.56370	1.34840	-3.19410
O	5.78760	2.10890	-0.62590
O	2.94490	2.79650	0.71340
O	2.85360	0.84900	-2.22580
C	0.35110	-0.34300	-1.34770
H	0.79840	-0.79180	-2.23310
H	-0.29640	0.48300	-1.64500
O	-0.42700	-1.33670	-0.64950
H	1.70890	-1.65430	0.38660
C	6.27030	3.26540	-0.12610
C	3.56750	3.02040	1.88930
C	-1.38460	-1.96620	-1.34910
C	7.64540	3.11340	0.40590
C	8.32010	1.89010	0.37620
C	9.60440	1.79590	0.89530
C	10.21940	2.91690	1.44560
C	9.54870	4.13690	1.47750
C	8.26570	4.23570	0.95980
O	5.63940	4.29930	-0.11690
C	-2.09570	-2.99460	-0.54720
C	-1.75690	-3.26310	0.78160
C	-2.44770	-4.23740	1.48990
C	-3.47770	-4.94680	0.87880
C	-3.81800	-4.68140	-0.44510
C	-3.12990	-3.70910	-1.15620
O	-1.62860	-1.70880	-2.50860
C	3.01390	4.19610	2.60330
C	1.96220	4.95430	2.08250
C	1.48280	6.04960	2.78820
C	2.04890	6.39420	4.01230
C	3.09860	5.64160	4.53300
C	3.58000	4.54610	3.83140

O	4.47520	2.33260	2.30060
H	7.83990	1.02110	-0.04920
H	10.12550	0.84760	0.87170
H	11.22070	2.83980	1.85040
H	10.02620	5.00840	1.90650
H	7.73240	5.17610	0.98020
H	-0.95770	-2.71210	1.25580
H	-2.18260	-4.44370	2.51880
H	-4.01460	-5.70590	1.43360
H	-4.61880	-5.23240	-0.92120
H	-3.38420	-3.49400	-2.18490
H	1.52560	4.68760	1.13120
H	0.66790	6.63560	2.38300
H	1.67330	7.24960	4.55980
H	3.54030	5.91010	5.48390
H	4.39560	3.95420	4.22320
C	4.86650	-1.17220	-2.76990
H	5.51190	-1.97090	-2.41130
H	3.84290	-1.54160	-2.85810
H	5.21520	-0.83090	-3.75020

Methyl α-D-Galf **4**, C1-exo, -60°/-60°

	**X**	**Y**	**Z**
C	4.46100	2.23000	-1.10360
H	4.39060	3.23650	-1.51330
C	3.28860	1.97000	-0.16660
H	3.50300	1.12610	0.48370
C	2.15100	1.66870	-1.14440
C	1.12310	0.68930	-0.58940
H	0.78080	1.13800	0.35370
O	1.75790	-0.55740	-0.34150
C	4.20270	1.20830	-2.20920
O	4.70160	-0.03190	-1.81270
H	1.62790	2.60220	-1.37010

H	4.61850	1.49090	-3.18080
O	5.73740	2.03570	-0.50570
O	2.92250	3.10790	0.62000
O	2.79200	1.21600	-2.35090
C	-0.12700	0.54210	-1.43960
H	-0.62040	1.50870	-1.55950
H	-0.82530	-0.14550	-0.95960
O	0.23700	0.02720	-2.72880
H	1.17920	-1.09080	0.21690
C	6.34400	3.10640	0.04650
C	3.47740	3.23520	1.84300
C	-0.74950	-0.24280	-3.59060
C	7.63600	2.74950	0.68000
C	8.12710	1.44130	0.67280
C	9.33860	1.15390	1.28700
C	10.06320	2.16580	1.91050
C	9.57540	3.47010	1.92020
C	8.36570	3.76190	1.30730
O	5.87410	4.22270	0.02330
C	-0.22860	-0.71260	-4.90090
C	1.13650	-0.67620	-5.19840
C	1.58680	-1.11680	-6.43560
C	0.68170	-1.59790	-7.37770
C	-0.67910	-1.63400	-7.08370
C	-1.13420	-1.18970	-5.85020
O	-1.92630	-0.11370	-3.32080
C	3.04200	4.47480	2.53050
C	2.16030	5.38230	1.93780
C	1.78760	6.53200	2.62100
C	2.29180	6.78250	3.89410
C	3.17220	5.88080	4.48650
C	3.54680	4.73070	3.80750
O	4.24340	2.42310	2.31230
H	7.56150	0.65750	0.19060
H	9.71740	0.14000	1.28060

H	11.00710	1.93800	2.38960
H	10.13780	4.25690	2.40610
H	7.97440	4.76980	1.30960
H	1.83310	-0.29590	-4.46530
H	2.64410	-1.08480	-6.66550
H	1.03580	-1.94300	-8.34090
H	-1.38310	-2.00760	-7.81600
H	-2.18860	-1.21110	-5.61050
H	1.77240	5.18820	0.94860
H	1.10470	7.23380	2.16000
H	1.99990	7.68050	4.42390
H	3.56590	6.07590	5.47560
H	4.23110	4.02300	4.25490
C	4.52450	-1.05610	-2.78890
H	5.05670	-1.93210	-2.42430
H	3.46770	-1.29680	-2.91590
H	4.94410	-0.74680	-3.75210

Methyl α-D-Galf **4**, C1-exo, -60°/180°

	**X**	**Y**	**Z**
C	4.02180	2.02390	-0.95290
H	3.76410	2.99330	-1.37760
C	3.06970	1.69580	0.19130
H	3.48470	0.91360	0.82080
C	1.82090	1.23690	-0.55910
C	1.00450	0.17680	0.16140
H	0.65040	0.63440	1.09280
O	1.82820	-0.94800	0.43680
C	3.69060	0.92190	-1.95790
O	4.38080	-0.23760	-1.60980
H	1.17310	2.10090	-0.72220
H	3.89980	1.19210	-2.99700
O	5.39820	1.99970	-0.59490
O	2.72170	2.83100	0.99050

O	2.28230	0.78040	-1.84520
C	-0.19770	-0.27460	-0.65760
H	-0.75170	-1.04710	-0.12290
H	0.11760	-0.66580	-1.62400
O	-1.03990	0.87690	-0.84660
H	1.39480	-1.49140	1.10620
C	5.96650	3.15930	-0.20530
C	3.46730	3.08660	2.08470
C	-2.13170	0.71950	-1.60980
C	7.38820	2.98380	0.17610
C	8.02620	1.74280	0.10520
C	9.35880	1.62800	0.47760
C	10.05850	2.74610	0.92270
C	9.42450	3.98380	0.99580
C	8.09350	4.10320	0.62390
O	5.37110	4.21360	-0.17430
C	-2.89990	1.98140	-1.76810
C	-2.46910	3.18440	-1.20240
C	-3.21640	4.34100	-1.38040
C	-4.39440	4.30410	-2.12100
C	-4.82650	3.10700	-2.68590
C	-4.08190	1.94940	-2.51110
O	-2.44100	-0.34210	-2.10810
C	3.00980	4.30050	2.80330
C	1.91180	5.04920	2.37180
C	1.52420	6.17980	3.07820
C	2.22890	6.56980	4.21360
C	3.32480	5.82680	4.64480
C	3.71460	4.69570	3.94260
O	4.40120	2.39630	2.42810
H	7.48040	0.87640	-0.23880
H	9.85170	0.66610	0.42160
H	11.09740	2.65300	1.21330
H	9.96790	4.85300	1.34310
H	7.58810	5.05760	0.67730

H	-1.55400	3.21210	-0.62870
H	-2.87960	5.27150	-0.94200
H	-4.97530	5.20760	-2.25810
H	-5.74240	3.07760	-3.26190
H	-4.40600	1.01380	-2.94570
H	1.36750	4.74710	1.48900
H	0.67290	6.75790	2.74270
H	1.92490	7.45270	4.76160
H	3.87420	6.13040	5.52650
H	4.56450	4.11060	4.26560
C	4.17360	-1.31890	-2.51660
H	4.83060	-2.12520	-2.19810
H	3.13680	-1.65980	-2.48860
H	4.42990	-1.01860	-3.53800

Methyl α-D-Galf **4**, C1-exo, 180°/+60°

	**X**	**Y**	**Z**
C	4.35030	2.23260	-1.11030
H	4.23220	3.24900	-1.48190
C	3.23470	1.90690	-0.12120
H	3.54710	1.08980	0.52550
C	2.06790	1.51960	-1.03890
C	1.29390	0.29870	-0.56350
H	1.98040	-0.55420	-0.51050
O	0.27250	0.05010	-1.52270
C	4.05850	1.24400	-2.23440
O	4.53650	-0.01540	-1.86430
H	1.37990	2.36200	-1.13700
H	4.45670	1.53950	-3.20850
O	5.65770	2.05330	-0.58050
O	2.84170	3.02430	0.68150
O	2.64780	1.28690	-2.33710
C	0.69300	0.54220	0.81020
H	-0.01120	1.37460	0.78610

H	1.45460	0.73850	1.56410
O	-0.00800	-0.66760	1.15830
H	-0.23690	-0.71490	-1.22590
C	6.26530	3.12630	-0.03100
C	3.43330	3.17220	1.88680
C	-0.67140	-0.66980	2.32670
C	7.60140	2.79140	0.51620
C	8.13190	1.50110	0.43660
C	9.38610	1.23500	0.96900
C	10.11430	2.25030	1.58270
C	9.58720	3.53670	1.66470
C	8.33470	3.80730	1.13350
O	5.76220	4.22720	0.00490
C	-1.35770	-1.95980	2.59160
C	-1.28850	-3.03310	1.69960
C	-1.94540	-4.22090	1.99240
C	-2.67280	-4.34410	3.17280
C	-2.74400	-3.27620	4.06350
C	-2.08870	-2.08800	3.77480
O	-0.69240	0.28680	3.07120
C	2.94860	4.37370	2.60560
C	1.97470	5.21850	2.06700
C	1.55430	6.33260	2.78070
C	2.10240	6.60970	4.02990
C	3.07440	5.77030	4.56830
C	3.49660	4.65550	3.85920
O	4.26030	2.39930	2.31640
H	7.56380	0.71440	-0.03830
H	9.79560	0.23510	0.90650
H	11.09170	2.03920	1.99780
H	10.15250	4.32600	2.14300
H	7.91280	4.80110	1.19210
H	-0.72280	-2.93770	0.78410
H	-1.88990	-5.05090	1.29990
H	-3.18360	-5.27170	3.39860

H	-3.30920	-3.37130	4.98160
H	-2.13530	-1.25140	4.45830
H	1.55130	5.00290	1.09700
H	0.79910	6.98550	2.36250
H	1.77270	7.47980	4.58360
H	3.50160	5.98610	5.53900
H	4.25090	3.99560	4.26490
C	4.34710	-1.01520	-2.86510
H	4.83040	-1.91870	-2.50020
H	3.28510	-1.20830	-3.02950
H	4.80980	-0.70660	-3.80800

Methyl α-D-Galf **4**, C1-exo, 180°/-60°

	**X**	**Y**	**Z**
C	4.08450	2.22280	-1.08570
H	3.94760	3.24290	-1.44000
C	2.96700	1.85470	-0.11450
H	3.29990	1.04970	0.53630
C	1.82320	1.43710	-1.04790
C	1.08890	0.17000	-0.62330
H	1.78370	-0.67400	-0.70040
O	0.00820	0.01180	-1.53850
C	3.82930	1.25060	-2.23250
O	4.33160	-0.00550	-1.88130
H	1.10280	2.25130	-1.12650
H	4.23280	1.57440	-3.19540
O	5.38890	2.06470	-0.54020
O	2.53220	2.96110	0.68160
O	2.42000	1.26590	-2.35080
C	0.61250	0.21990	0.81860
H	1.45040	0.21480	1.51290
H	-0.01510	-0.64670	1.03520
O	-0.16730	1.41490	1.01100
H	-0.38660	-0.85860	-1.40390

C	5.95940	3.13970	0.04410
C	3.06940	3.11520	1.91120
C	-0.17980	1.95780	2.23880
C	7.29570	2.82710	0.60410
C	7.85720	1.55090	0.51190
C	9.11010	1.30560	1.05720
C	9.80640	2.32790	1.69570
C	9.24850	3.60030	1.79020
C	7.99700	3.85000	1.24670
O	5.42670	4.22580	0.09810
C	-0.91180	3.24710	2.28300
C	-1.39980	3.85810	1.12570
C	-2.04950	5.08170	1.21100
C	-2.21810	5.69750	2.44770
C	-1.73350	5.08960	3.60210
C	-1.08050	3.86950	3.52070
O	0.36390	1.44730	3.19590
C	2.54930	4.32390	2.59090
C	1.82680	5.30180	1.90330
C	1.36520	6.42250	2.57750
C	1.61050	6.56780	3.93910
C	2.32510	5.59210	4.62870
C	2.79920	4.47550	3.95590
O	3.86950	2.34130	2.38730
H	7.31390	0.75880	0.01750
H	9.54350	0.31640	0.98530
H	10.78310	2.13310	2.12060
H	9.78900	4.39510	2.28780
H	7.55110	4.83270	1.31490
H	-1.26130	3.38030	0.16680
H	-2.42120	5.55730	0.31260
H	-2.72110	6.65420	2.51080
H	-1.85550	5.57290	4.56260
H	-0.68570	3.39390	4.40750
H	1.62780	5.18180	0.84900

H	0.80220	7.17630	2.04370
H	1.23900	7.43830	4.46490
H	2.51140	5.70240	5.68920
H	3.35630	3.71040	4.47900
C	4.18540	-0.98680	-2.90690
H	4.68120	-1.88750	-2.55180
H	3.13150	-1.20000	-3.09820
H	4.66000	-0.64750	-3.83320

Methyl α-D-Galf **4**, C1-exo, 180°/180°

	**X**	**Y**	**Z**
C	4.55370	2.31480	-1.21690
H	4.42080	3.33020	-1.58620
C	3.48340	1.99660	-0.17410
H	3.79150	1.14040	0.42170
C	2.26040	1.69180	-1.05110
C	1.36480	0.56550	-0.54700
H	1.96980	-0.33320	-0.38640
O	0.39190	0.34620	-1.56540
C	4.19850	1.32300	-2.31770
O	4.68030	0.05940	-1.96240
H	1.65530	2.59660	-1.15390
H	4.55630	1.60620	-3.31120
O	5.88710	2.12970	-0.76470
O	3.15740	3.08070	0.70030
O	2.78640	1.37500	-2.35500
C	0.61410	0.93290	0.73000
H	-0.18990	0.21370	0.89400
H	0.17620	1.92740	0.64450
O	1.50820	0.89220	1.85670
H	-0.09320	-0.46250	-1.35930
C	6.55920	3.21330	-0.32440
C	3.87380	3.19200	1.83920
C	1.00690	1.34850	3.01940

C	7.90700	2.86310	0.18460
C	8.36510	1.54360	0.21860
C	9.63170	1.26330	0.71340
C	10.44520	2.29420	1.17540
C	9.99050	3.61010	1.14370
C	8.72540	3.89430	0.65070
O	6.10540	4.33550	-0.35250
C	1.98370	1.28980	4.13280
C	3.19820	0.61180	4.01760
C	4.08810	0.60180	5.08260
C	3.77380	1.27170	6.26100
C	2.56190	1.94750	6.37860
C	1.66740	1.95310	5.32030
O	-0.12510	1.77100	3.12330
C	3.26630	4.13920	2.80080
C	1.99310	4.67820	2.60290
C	1.43480	5.50440	3.56810
C	2.14560	5.80200	4.72720
C	3.41810	5.27190	4.92260
C	3.97620	4.44030	3.96470
O	4.88620	2.56180	2.04470
H	7.73100	0.74530	-0.13830
H	9.98450	0.24040	0.73990
H	11.43230	2.07230	1.56110
H	10.62200	4.41190	1.50380
H	8.35880	4.91120	0.62260
H	3.44740	0.10540	3.09700
H	5.03110	0.07880	4.99020
H	4.47460	1.27030	7.08640
H	2.32080	2.47630	7.29160
H	0.72840	2.48360	5.39310
H	1.43840	4.43430	1.70870
H	0.44300	5.91130	3.41960
H	1.70590	6.44370	5.48040
H	3.96800	5.49860	5.82670

H	4.95470	4.00540	4.11180
C	4.43930	-0.94060	-2.95130
H	4.92170	-1.85050	-2.60120
H	3.36900	-1.11810	-3.07610
H	4.87170	-0.64260	-3.91200

Phenyl β-D-Galf **5**, gg

	**X**	**Y**	**Z**
O	-2.51630	1.99850	-0.36630
C	-2.13640	2.94910	-1.25890
O	-0.98630	3.29090	-1.40850
C	-3.27990	3.46960	-2.04300
C	-4.58110	2.99640	-1.85660
C	-5.60700	3.45720	-2.66970
C	-5.34000	4.38390	-3.67300
C	-4.04370	4.85680	-3.86140
C	-3.01680	4.40350	-3.04820
H	-4.78230	2.26060	-1.09250
H	-6.61350	3.08590	-2.52740
H	-6.14080	4.73360	-4.31230
H	-3.83470	5.57170	-4.64650
H	-2.00330	4.75060	-3.19320
O	-1.56570	0.01300	-2.02870
O	-0.89790	-1.42020	1.25740
O	-1.75510	-1.23470	3.87000
C	-0.75470	-1.27540	-0.14390
C	-1.75470	-0.23880	-0.63340
C	-1.51860	1.07800	0.09730
C	-1.62800	0.88990	1.60610
C	-0.64010	-0.21890	1.98600
C	-0.55600	-0.56600	3.45530
H	-2.76130	-0.60850	-0.45630
H	-0.53220	1.46930	-0.14940
H	0.35620	0.16080	1.71950

C	-2.66580	0.04000	-2.82160
C	-1.83920	-1.57270	5.16270
O	-2.94740	0.54780	1.99560
H	0.29330	-1.22840	3.62430
H	-0.41360	0.34200	4.04250
O	-1.06690	-2.49050	-0.74590
H	0.27210	-0.97290	-0.38980
C	-0.10800	-3.47860	-0.79350
H	-1.30560	1.81300	2.10080
H	-3.53810	1.24810	1.68850
O	-3.73270	-0.42310	-2.49130
C	-2.41280	0.74140	-4.10210
C	-1.16310	1.27920	-4.41860
C	-0.99560	1.99100	-5.59830
C	-2.07150	2.17450	-6.46180
C	-3.31850	1.63980	-6.14840
C	-3.48900	0.92340	-4.97380
H	-0.33440	1.15240	-3.73820
H	-0.02760	2.41030	-5.84020
H	-1.93980	2.73830	-7.37670
H	-4.15690	1.78830	-6.81630
H	-4.45470	0.51500	-4.71070
C	-3.11580	-2.26180	5.48420
O	-0.96370	-1.33400	5.96940
C	-3.32450	-2.69700	6.79450
C	-4.50440	-3.34340	7.13390
C	-5.48300	-3.55810	6.16670
C	-5.27900	-3.12510	4.85960
C	-4.09930	-2.47850	4.51560
H	-2.55650	-2.52430	7.53600
H	-4.66210	-3.68010	8.15050
H	-6.40370	-4.06240	6.43170
H	-6.03980	-3.29140	4.10780
H	-3.93840	-2.13970	3.50250
C	-0.37140	-4.52870	-1.66960

C	0.53500	-5.57340	-1.78280
C	1.70690	-5.57500	-1.02940
C	1.95690	-4.52380	-0.15600
C	1.05250	-3.47240	-0.02480
H	-1.28210	-4.50910	-2.25420
H	0.32650	-6.38630	-2.46680
H	2.41360	-6.38950	-1.12020
H	2.85930	-4.51790	0.44240
H	1.24710	-2.67870	0.68100

Phenyl β-D-Galf **5**, gt

	**X**	**Y**	**Z**
O	-2.44060	2.03150	-0.39980
C	-2.03920	2.96660	-1.29950
O	-0.88040	3.27120	-1.46160
C	-3.17350	3.52090	-2.07350
C	-4.48690	3.08880	-1.87280
C	-5.50640	3.58070	-2.67570
C	-5.22100	4.49740	-3.68320
C	-3.91260	4.92900	-3.88600
C	-2.89180	4.44490	-3.08290
H	-4.70270	2.36050	-1.10550
H	-6.52250	3.24130	-2.52220
H	-6.01720	4.87140	-4.31430
H	-3.68970	5.63600	-4.67440
H	-1.86960	4.75970	-3.23890
O	-1.56310	0.01120	-2.06520
O	-0.96630	-1.42540	1.22780
O	0.33370	-1.53200	3.68710
C	-0.78640	-1.29240	-0.16880
C	-1.75400	-0.23120	-0.66850
C	-1.46840	1.08070	0.05630
C	-1.55580	0.91490	1.56910
C	-0.63260	-0.24240	1.95670

C	-0.71190	-0.57850	3.42530
H	-2.77140	-0.56720	-0.48580
H	-0.47270	1.43710	-0.20630
H	0.39550	0.05490	1.71470
C	-2.66640	0.07250	-2.85240
C	0.43990	-1.99140	4.94240
O	-2.87490	0.61790	2.00140
H	-0.55630	0.31560	4.02940
H	-1.67560	-1.01740	3.67640
O	-1.10790	-2.50420	-0.77220
H	0.25160	-1.01120	-0.39260
C	-0.19170	-3.53230	-0.71920
H	-1.18910	1.83020	2.04560
H	-3.44600	1.36100	1.76800
O	-3.74530	-0.35700	-2.51570
C	-2.39950	0.76560	-4.13440
C	-1.13610	1.26620	-4.45770
C	-0.95400	1.97280	-5.63830
C	-2.02860	2.18810	-6.49600
C	-3.28920	1.69040	-6.17580
C	-3.47460	0.97950	-5.00030
H	-0.30790	1.11520	-3.78150
H	0.02460	2.36340	-5.88540
H	-1.88530	2.74780	-7.41170
H	-4.12640	1.86380	-6.83930
H	-4.45040	0.59970	-4.73180
C	1.49520	-3.02850	5.07830
O	-0.25720	-1.59520	5.85280
C	1.80640	-3.49620	6.35680
C	2.77830	-4.47250	6.52320
C	3.44020	-4.99250	5.41370
C	3.12970	-4.53230	4.13720
C	2.16270	-3.55090	3.96760
H	1.28170	-3.08780	7.20960
H	3.01920	-4.83030	7.51600

H	4.19590	-5.75670	5.54420
H	3.63940	-4.93910	3.27330
H	1.91810	-3.19550	2.97820
C	-0.47570	-4.63230	-1.52500
C	0.38520	-5.72090	-1.53080
C	1.53190	-5.71870	-0.73950
C	1.80410	-4.61650	0.06130
C	0.94690	-3.51900	0.08190
H	-1.36770	-4.61870	-2.13780
H	0.15970	-6.57300	-2.15970
H	2.20120	-6.56910	-0.74490
H	2.68610	-4.60470	0.68930
H	1.15910	-2.68370	0.73130

Phenyl β-D-Galf **5**, tg

	**X**	**Y**	**Z**
O	-2.49140	1.93100	-0.25330
C	-2.08520	2.90660	-1.10570
O	-0.92830	3.23680	-1.22770
C	-3.20950	3.47260	-1.88630
C	-4.52010	3.01280	-1.73540
C	-5.52720	3.51780	-2.54590
C	-5.23190	4.47530	-3.51170
C	-3.92620	4.93500	-3.66460
C	-2.91810	4.43770	-2.85350
H	-4.74300	2.25340	-1.00090
H	-6.54100	3.15700	-2.43120
H	-6.01800	4.85940	-4.14940
H	-3.69530	5.67390	-4.42080
H	-1.89770	4.77420	-2.97150
O	-1.54710	0.00150	-1.98640
O	-0.94440	-1.57370	1.24590
O	-0.46250	0.26950	4.28720
C	-0.77530	-1.37220	-0.14630

C	-1.75750	-0.30500	-0.60520
C	-1.51340	0.97610	0.18310
C	-1.63440	0.72860	1.68010
C	-0.67440	-0.41150	2.03110
C	-0.77710	-0.87030	3.47220
H	-2.77060	-0.66960	-0.45640
H	-0.51830	1.36230	-0.03650
H	0.34620	-0.06060	1.83400
C	-2.63530	0.06980	-2.79300
C	-0.54030	0.10180	5.61730
O	-2.95120	0.35710	2.05770
H	-1.78190	-1.22160	3.69790
H	-0.06330	-1.67120	3.66190
O	-1.08950	-2.55740	-0.80300
H	0.25810	-1.07010	-0.36140
C	-0.13720	-3.54990	-0.88440
H	-1.31740	1.62790	2.21570
H	-3.54100	1.09920	1.87410
O	-3.71230	-0.39150	-2.49440
C	-2.35550	0.81230	-4.04450
C	-1.09380	1.34110	-4.32660
C	-0.90010	2.09010	-5.47890
C	-1.96180	2.31980	-6.34900
C	-3.22070	1.79430	-6.06970
C	-3.41740	1.04090	-4.92270
H	-0.27590	1.17780	-3.64080
H	0.07730	2.50200	-5.69430
H	-1.80960	2.91230	-7.24230
H	-4.04800	1.97860	-6.74250
H	-4.39240	0.63840	-4.68600
C	-0.23370	1.34690	6.36640
O	-0.83110	-0.95720	6.13060
C	-0.26060	1.30250	7.76210
C	0.01750	2.44360	8.50060
C	0.32350	3.63610	7.84990

C	0.35040	3.68500	6.45910
C	0.07300	2.54520	5.71640
H	-0.50060	0.37050	8.25500
H	-0.00410	2.40480	9.58210
H	0.54040	4.52650	8.42650
H	0.58720	4.61210	5.95310
H	0.09290	2.58230	4.63680
C	-0.39920	-4.55820	-1.80870
C	0.50110	-5.60350	-1.95910
C	1.66520	-5.64720	-1.19510
C	1.91350	-4.63770	-0.27330
C	1.01510	-3.58650	-0.10450
H	-1.30360	-4.50590	-2.40090
H	0.29390	-6.38390	-2.68050
H	2.36710	-6.46210	-1.31490
H	2.80970	-4.66500	0.33370
H	1.20850	-2.82630	0.63770

Phenyl β-D-Galf **6**, C1-endo, +60°/+60°

	**X**	**Y**	**Z**
C	2.38100	3.02950	-4.17010
O	1.89970	4.27030	-4.61750
C	1.25530	2.45780	-3.31730
C	0.65300	3.70950	-2.66160
C	1.08510	4.86120	-3.59060
O	3.49560	3.15860	-3.31240
H	2.62410	2.42520	-5.04340
O	1.25230	3.89660	-1.37550
C	4.70030	3.56710	-3.82980
C	5.76000	3.59350	-2.92350
H	1.58830	1.72660	-2.58750
O	0.32680	1.87700	-4.23730
C	-0.61140	1.04870	-3.72410
C	0.46280	3.85420	-0.28050

H	-0.42190	3.63010	-2.54330
C	-0.03880	5.64340	-4.25860
C	-0.98620	4.78700	-5.07700
H	0.42810	6.37070	-4.93300
O	-0.72040	6.31490	-3.20520
O	-1.90720	5.71550	-5.68670
H	-0.45530	4.23590	-5.85160
H	-1.54740	4.08890	-4.45770
H	-1.45460	6.81120	-3.58920
H	1.67880	5.56710	-3.00660
C	-2.93160	5.19630	-6.38250
C	-3.82980	6.24020	-6.93950
C	-3.59860	7.60290	-6.73440
C	-4.46710	8.54220	-7.27410
C	-5.56790	8.12880	-8.01910
C	-5.80110	6.77160	-8.22510
C	-4.93560	5.83000	-7.68750
H	-2.74400	7.92330	-6.15640
H	-4.28570	9.59710	-7.11360
H	-6.24350	8.86360	-8.43860
H	-6.65690	6.44910	-8.80400
H	-5.10610	4.77330	-7.84000
C	1.24720	4.01950	0.96840
C	0.55850	4.03990	2.18340
C	1.25340	4.18750	3.37490
C	2.64010	4.31360	3.36030
C	3.33040	4.29230	2.15170
C	2.63910	4.14620	0.95670
H	-0.51810	3.93900	2.18120
H	0.71610	4.20370	4.31430
H	3.18240	4.42800	4.29050
H	4.40840	4.38900	2.14050
H	3.17370	4.12810	0.01820
C	-1.52110	0.51910	-4.76610
C	-1.35750	0.82970	-6.11840

C	-2.57090	-0.31130	-4.36550
C	-3.44850	-0.82560	-5.30800
C	-3.28190	-0.51540	-6.65560
C	-2.23700	0.31080	-7.05840
H	-0.55090	1.47690	-6.42920
H	-2.68940	-0.54340	-3.31610
H	-4.26220	-1.46660	-4.99460
H	-3.96800	-0.91620	-7.39110
H	-2.11050	0.55520	-8.10500
O	-3.08930	4.00350	-6.52990
O	-0.73660	3.70130	-0.32670
O	-0.67700	0.79040	-2.54360
C	7.02250	3.98040	-3.34830
C	7.23970	4.34360	-4.67610
C	6.17650	4.31900	-5.56960
C	4.90130	3.93700	-5.15750
H	5.57760	3.30500	-1.89630
H	7.84040	3.99630	-2.63900
H	8.22500	4.64530	-5.00640
H	6.32910	4.60560	-6.60260
H	4.08550	3.94890	-5.86430

Phenyl β-D-Galf **6**, C1-endo, +60°/-60°

	**X**	**Y**	**Z**
C	0.11070	3.20680	-1.80560
O	0.54270	3.91450	-2.93790
C	-1.29340	3.72780	-1.52990
C	-1.21980	5.19580	-1.97610
C	0.04560	5.25960	-2.85710
O	0.87280	3.51450	-0.65570
H	0.15210	2.14260	-2.03320
O	-1.04480	6.03670	-0.83070
C	2.17330	3.07970	-0.57430
C	2.83220	3.38970	0.61510

C	2.83080	2.37180	-1.57770
H	-1.59600	3.62660	-0.49230
O	-2.16580	2.98640	-2.38820
C	-3.48760	3.05420	-2.11490
C	-1.96090	7.00070	-0.59970
H	-2.11510	5.49300	-2.50270
C	-0.11280	5.79140	-4.27820
C	-1.05240	5.02070	-5.19090
H	0.87600	5.70440	-4.74620
O	-0.47950	7.15680	-4.13310
O	-2.41270	5.20080	-4.75930
H	-0.95490	5.39260	-6.21140
H	-0.80220	3.95950	-5.18240
H	-0.53320	7.56340	-5.00690
H	0.77600	5.89430	-2.35010
C	-3.37540	4.75120	-5.58000
C	-4.73840	5.02750	-5.06310
C	-4.95270	5.77620	-3.90320
C	-6.24620	5.99070	-3.44850
C	-7.32910	5.46530	-4.14730
C	-7.11780	4.72510	-5.30690
C	-5.82720	4.50620	-5.76370
H	-4.11760	6.18710	-3.35580
H	-6.40800	6.56550	-2.54580
H	-8.33680	5.63040	-3.78700
H	-7.95860	4.30970	-5.84720
H	-5.64840	3.92070	-6.65440
C	-1.64810	7.77890	0.62430
C	-2.49690	8.83250	0.97150
C	-2.24160	9.58620	2.10780
C	-1.13840	9.29120	2.90480
C	-0.29080	8.24120	2.56280
C	-0.54170	7.48510	1.42580
H	-3.35020	9.05040	0.34410
H	-2.90090	10.40250	2.37310

H	-0.93950	9.87950	3.79180
H	0.56620	8.01160	3.18270
H	0.11500	6.67000	1.15910
C	-4.30350	2.29260	-3.08740
C	-3.73220	1.65340	-4.19030
C	-5.68530	2.24440	-2.89390
C	-6.48820	1.56190	-3.79360
C	-5.91740	0.92750	-4.89350
C	-4.54090	0.97520	-5.09090
H	-2.66470	1.70050	-4.34730
H	-6.11720	2.75500	-2.04490
H	-7.55990	1.53450	-3.64650
H	-6.54660	0.40230	-5.60100
H	-4.09810	0.48960	-5.95080
O	-3.13690	4.18200	-6.62440
O	-2.91770	7.19980	-1.31420
O	-3.92750	3.67830	-1.17530
C	4.14810	1.96720	-1.37120
C	4.14510	2.98380	0.80370
C	4.81170	2.26760	-0.18850
H	2.30130	3.94290	1.37900
H	2.34420	2.14550	-2.51440
H	4.65560	1.41670	-2.15350
H	4.64870	3.22720	1.73080
H	5.83610	1.95190	-0.04070

Phenyl β-D-Galf **6**, C1-endo, +60°/180°

	**X**	**Y**	**Z**
C	2.69290	5.80680	-6.13760
O	1.52280	6.29440	-5.54190
C	2.76030	4.33480	-5.75030
C	2.10990	4.30910	-4.35950
C	1.28980	5.61170	-4.30010
O	3.85930	6.41020	-5.61130

H	2.62180	5.97160	-7.21230
O	3.14240	4.35500	-3.36860
C	4.12320	7.72230	-5.91820
C	5.32770	8.22200	-5.42380
C	3.28100	8.53780	-6.67130
H	3.77130	3.93910	-5.73550
O	1.96010	3.63880	-6.71100
C	2.06650	2.29160	-6.73250
C	3.21030	3.36170	-2.45630
H	1.51330	3.41650	-4.20700
C	-0.21360	5.44930	-4.08650
C	-0.94580	4.51640	-5.04450
H	-0.66020	6.44600	-4.16920
O	-0.34000	4.94250	-2.76160
O	-0.96000	5.12520	-6.34240
H	-0.47630	3.53440	-5.10670
H	-1.97060	4.38020	-4.69440
H	-1.26990	4.96560	-2.50280
H	1.66940	6.21550	-3.47300
C	-1.62740	4.48980	-7.31370
C	-1.45210	5.15200	-8.63090
C	-0.50840	6.16570	-8.81620
C	-0.33550	6.72760	-10.07380
C	-1.10620	6.28800	-11.14680
C	-2.05130	5.28200	-10.96220
C	-2.22120	4.71210	-9.70870
H	0.09230	6.49650	-7.98110
H	0.40220	7.50640	-10.21820
H	-0.96950	6.72770	-12.12670
H	-2.65020	4.93980	-11.79630
H	-2.94120	3.92030	-9.55450
C	4.34240	3.55510	-1.51610
C	4.54620	2.59570	-0.52170
C	5.59340	2.73670	0.37720
C	6.44260	3.83670	0.28900

C	6.24260	4.79530	-0.70030
C	5.19660	4.65780	-1.60270
H	3.87980	1.74620	-0.46380
H	5.74850	1.99090	1.14600
H	7.25980	3.94660	0.99070
H	6.90270	5.65040	-0.76900
H	5.04090	5.40050	-2.37140
C	1.24560	1.68290	-7.80420
C	0.71440	2.44520	-8.84640
C	1.01560	0.30560	-7.76100
C	0.25080	-0.30060	-8.74640
C	-0.27700	0.46240	-9.78560
C	-0.04140	1.83270	-9.83610
H	0.89400	3.50900	-8.88240
H	1.43300	-0.27470	-6.94950
H	0.06460	-1.36600	-8.70580
H	-0.87260	-0.01220	-10.55520
H	-0.45220	2.42710	-10.64100
O	-2.28290	3.48650	-7.12530
O	2.43480	2.43370	-2.41910
O	2.75730	1.67800	-5.95070
C	3.66560	9.85060	-6.93600
C	4.86590	10.35580	-6.45240
C	5.69420	9.53270	-5.69190
H	5.96390	7.57040	-4.83870
H	2.33270	8.17570	-7.03840
H	3.00850	10.48070	-7.52220
H	5.15270	11.37810	-6.66100
H	6.63220	9.91180	-5.30610

Phenyl β-D-Galf **6**, C1-endo, -60°/+60°

	**X**	**Y**	**Z**
C	-1.87630	3.12480	-2.91940
O	-1.41810	4.05460	-3.85970

C	-1.35940	3.62430	-1.57660
C	-0.02710	4.28470	-1.94060
C	-0.12410	4.54520	-3.46110
O	-1.31120	1.84220	-3.10880
H	-2.96300	3.08590	-2.97930
O	1.00500	3.33640	-1.62900
C	-1.72730	1.08250	-4.17510
C	-1.14460	-0.18110	-4.27220
C	-2.66810	1.49600	-5.11430
H	-1.23980	2.83520	-0.84070
O	-2.30530	4.59740	-1.12640
C	-2.20140	5.00580	0.15490
C	2.27230	3.78780	-1.63400
H	0.13880	5.19830	-1.37800
C	0.02920	6.01080	-3.84200
C	0.14180	6.26400	-5.33880
H	0.98980	6.32470	-3.41860
O	-1.03830	6.73940	-3.24380
O	-1.02650	5.83850	-6.06340
H	0.21970	7.33620	-5.51590
H	1.02410	5.76650	-5.73710
H	-0.80450	7.67540	-3.23990
H	0.65290	3.97030	-3.96960
C	-0.96850	4.67530	-6.73220
C	-2.27220	4.31600	-7.34400
C	-3.46950	4.88140	-6.90130
C	-4.67330	4.48520	-7.46870
C	-4.68570	3.54070	-8.49140
C	-3.49190	2.98300	-8.94030
C	-2.28940	3.36050	-8.36070
H	-3.45480	5.61130	-6.10480
H	-5.60210	4.91340	-7.11430
H	-5.62530	3.23680	-8.93540
H	-3.50080	2.24570	-9.73260
H	-1.35750	2.91940	-8.68640

C	3.25280	2.72440	-1.30780
C	4.60390	3.07090	-1.23470
C	5.55430	2.10520	-0.93760
C	5.16130	0.78790	-0.71450
C	3.81590	0.43840	-0.78820
C	2.86100	1.40190	-1.08320
H	4.89560	4.09700	-1.41160
H	6.60030	2.37720	-0.88020
H	5.90350	0.03420	-0.48370
H	3.51090	-0.58570	-0.61620
H	1.81660	1.13200	-1.14270
C	-3.19530	6.05240	0.49270
C	-4.03440	6.61320	-0.47390
C	-3.27340	6.48700	1.81790
C	-4.18530	7.46950	2.17470
C	-5.02050	8.02680	1.20960
C	-4.94260	7.59920	-0.11280
H	-3.96860	6.28340	-1.50040
H	-2.61770	6.04800	2.55710
H	-4.24550	7.80200	3.20290
H	-5.73090	8.79490	1.48810
H	-5.58950	8.03490	-0.86320
O	0.03720	4.00550	-6.83520
O	2.55770	4.93960	-1.88360
O	-1.37970	4.55450	0.92200
C	-3.03450	0.62470	-6.13730
C	-2.46520	-0.63780	-6.23960
C	-1.51360	-1.03430	-5.30150
H	-0.41070	-0.47750	-3.53390
H	-3.10260	2.48280	-5.07870
H	-3.76290	0.95430	-6.86570
H	-2.75280	-1.30420	-7.04230
H	-1.05720	-2.01390	-5.36960

Phenyl β-D-Galf **6**, C1-endo, -60°/-60°

	**X**	**Y**	**Z**
C	1.77030	2.49510	-3.27710
O	1.25540	3.44330	-4.16980
C	1.46770	3.05130	-1.89120
C	1.57780	4.56650	-2.09280
C	1.43930	4.76170	-3.61860
O	3.17900	2.38830	-3.35210
H	1.29270	1.53760	-3.48250
O	2.87500	4.96090	-1.62320
C	3.75330	1.84490	-4.47490
C	5.14360	1.73930	-4.44180
C	3.04240	1.41140	-5.59160
H	2.14490	2.68880	-1.12410
O	0.12020	2.67330	-1.59860
C	-0.30440	2.86330	-0.33180
C	3.08530	6.28000	-1.45460
H	0.81320	5.10370	-1.54140
C	0.28580	5.65460	-4.03680
C	0.15480	5.70000	-5.54650
H	0.51970	6.65980	-3.67140
O	-0.90890	5.17700	-3.42680
O	-0.88380	6.66030	-5.82940
H	1.08580	6.02450	-6.01340
H	-0.13300	4.72960	-5.94710
H	-1.61770	5.80030	-3.62820
H	2.36760	5.18790	-4.00700
C	-1.22310	6.82720	-7.11720
C	-2.31440	7.81870	-7.29820
C	-2.90670	8.47520	-6.21620
C	-3.92500	9.39330	-6.43560
C	-4.35680	9.66120	-7.73160
C	-3.76850	9.00870	-8.81190
C	-2.75100	8.09050	-8.59670
H	-2.57050	8.26800	-5.21060

H	-4.38200	9.90030	-5.59560
H	-5.15080	10.37770	-7.89960
H	-4.10350	9.21630	-9.82000
H	-2.28590	7.57690	-9.42680
C	4.45650	6.57730	-0.97540
C	4.78970	7.90750	-0.70960
C	6.06300	8.22970	-0.26350
C	7.01140	7.22600	-0.08300
C	6.68400	5.89950	-0.34940
C	5.41010	5.57210	-0.79380
H	4.04430	8.67720	-0.85550
H	6.31740	9.26110	-0.05680
H	8.00540	7.47790	0.26430
H	7.42190	5.12000	-0.21080
H	5.15500	4.54330	-1.00250
C	-1.73050	2.50080	-0.15540
C	-2.53740	2.12990	-1.23430
C	-2.26990	2.54920	1.13230
C	-3.60240	2.22490	1.34020
C	-4.40450	1.85540	0.26340
C	-3.87150	1.81030	-1.02180
H	-2.12330	2.10150	-2.23160
H	-1.63660	2.84040	1.95900
H	-4.01690	2.26070	2.33940
H	-5.44500	1.60430	0.42590
H	-4.49620	1.52650	-1.85880
O	-0.68450	6.22640	-8.02260
O	2.23410	7.11330	-1.67930
O	0.41820	3.28650	0.54330
C	3.73690	0.85930	-6.66610
C	5.12100	0.74560	-6.64080
C	5.82080	1.19150	-5.52130
H	5.67450	2.08670	-3.56480
H	1.96880	1.51040	-5.64440
H	3.18040	0.52350	-7.53210

H	5.65030	0.31880	-7.48270
H	6.90010	1.11150	-5.48660

Phenyl β-D-Galf **6**, C1-endo, -60°/180°

	**X**	**Y**	**Z**
C	3.51180	5.04980	-3.45250
O	2.31920	5.12960	-4.17310
C	3.07300	4.98940	-1.99380
C	1.83010	5.89160	-1.97850
C	1.43510	6.01780	-3.46520
O	4.28460	6.23610	-3.55930
H	4.06530	4.17680	-3.79700
O	2.23210	7.15700	-1.43390
C	4.56560	6.74310	-4.80690
C	4.96620	8.07700	-4.83460
C	4.47850	6.01000	-5.98760
H	3.83380	5.32360	-1.29510
O	2.71490	3.62760	-1.74350
C	2.49620	3.28090	-0.45770
C	1.25680	8.02480	-1.11080
H	1.03360	5.46780	-1.37530
C	0.00100	5.66410	-3.81010
C	-0.20250	5.62140	-5.32280
H	-0.63170	6.43470	-3.36550
O	-0.31790	4.38370	-3.27190
O	0.24830	6.87770	-5.86260
H	0.36830	4.80510	-5.75650
H	-1.25850	5.49400	-5.56330
H	-1.25960	4.35940	-3.06640
H	1.62580	7.04290	-3.78780
C	0.87190	6.87640	-7.05280
C	1.44560	8.20460	-7.38580
C	1.56910	9.21350	-6.42850
C	2.13720	10.43170	-6.77530

C	2.57310	10.65320	-8.07770
C	2.44900	9.64990	-9.03500
C	1.89390	8.42730	-8.68890
H	1.23430	9.03840	-5.41650
H	2.24430	11.20690	-6.02760
H	3.01470	11.60480	-8.34570
H	2.79210	9.81950	-10.04740
H	1.80330	7.63560	-9.41980
C	1.79430	9.32240	-0.63470
C	0.89560	10.27420	-0.14780
C	1.35830	11.50440	0.29580
C	2.71980	11.79330	0.25020
C	3.61820	10.84890	-0.23850
C	3.16030	9.61460	-0.67910
H	-0.15940	10.03860	-0.12040
H	0.65960	12.23850	0.67580
H	3.08020	12.75450	0.59450
H	4.67600	11.07480	-0.27670
H	3.85550	8.88150	-1.06160
C	2.07400	1.86680	-0.31960
C	1.82120	1.05950	-1.43190
C	1.91740	1.34640	0.96720
C	1.51650	0.02980	1.14060
C	1.26520	-0.77260	0.03060
C	1.41610	-0.25630	-1.25330
H	1.93390	1.46340	-2.42740
H	2.11380	1.98070	1.82050
H	1.39870	-0.37130	2.13890
H	0.95030	-1.79960	0.16630
H	1.21720	-0.87930	-2.11570
O	0.95020	5.89820	-7.76490
O	0.07820	7.75830	-1.21230
O	2.63340	4.05780	0.46130
C	4.79720	6.62700	-7.19380
C	5.19950	7.95590	-7.23180

C	5.28410	8.67710	-6.04480
H	5.01220	8.62960	-3.90490
H	4.15010	4.98160	-5.98230
H	4.71240	6.05940	-8.11180
H	5.42550	8.43090	-8.17680
H	5.58310	9.71740	-6.06050

Phenyl β-D-Galf **6**, C1-endo, 180°/+60°

	**X**	**Y**	**Z**
C	3.33630	4.59940	-4.75120
O	2.43680	5.66850	-4.54320
C	2.51090	3.31130	-4.67590
C	1.07010	3.80440	-4.56150
C	1.24950	5.17400	-3.91070
O	4.32440	4.53110	-3.74540
H	3.80750	4.73750	-5.72440
O	0.26090	2.97620	-3.72830
C	5.36060	5.43470	-3.77830
C	6.43070	5.15400	-2.92990
C	5.38080	6.57120	-4.58340
H	2.78120	2.74110	-3.79020
O	2.74320	2.52960	-5.84680
C	2.67070	1.18450	-5.72420
C	-0.46810	2.00510	-4.32370
H	0.62310	3.89140	-5.55150
C	0.12950	6.17010	-4.13900
C	-1.21540	5.62110	-3.69130
H	0.08380	6.39570	-5.21120
O	0.46620	7.34170	-3.40420
O	-2.15870	6.69020	-3.89730
H	-1.52260	4.76070	-4.28280
H	-1.20330	5.34710	-2.63630
H	-0.24710	7.98200	-3.52340
H	1.41290	5.05600	-2.83350

C	-3.42800	6.45910	-3.52070
C	-4.31590	7.62490	-3.75990
C	-3.84070	8.81770	-4.31130
C	-4.70970	9.88110	-4.51560
C	-6.05330	9.76100	-4.17230
C	-6.52990	8.57370	-3.62270
C	-5.66480	7.50890	-3.41680
H	-2.79810	8.91050	-4.57880
H	-4.33910	10.80400	-4.94290
H	-6.72860	10.59200	-4.33290
H	-7.57450	8.47950	-3.35560
H	-6.02220	6.58150	-2.99090
C	-1.31310	1.26880	-3.35630
C	-2.03510	0.16570	-3.81820
C	-2.84650	-0.54740	-2.94820
C	-2.94490	-0.16030	-1.61400
C	-2.22900	0.94000	-1.15090
C	-1.41230	1.65410	-2.01680
H	-1.95060	-0.12250	-4.85690
H	-3.40240	-1.40340	-3.30800
H	-3.58000	-0.71590	-0.93570
H	-2.30810	1.24200	-0.11460
H	-0.85760	2.50920	-1.65940
C	2.86890	0.49260	-7.01950
C	3.09290	1.19150	-8.20850
C	2.81850	-0.90340	-7.03340
C	2.99220	-1.59390	-8.22370
C	3.21670	-0.89480	-9.40700
C	3.26650	0.49620	-9.39760
H	3.12890	2.27100	-8.20070
H	2.64150	-1.43340	-6.10770
H	2.95200	-2.67540	-8.23080
H	3.35170	-1.43410	-10.33610
H	3.43980	1.03940	-10.31750
O	-3.78820	5.40550	-3.04170

O	-0.42540	1.78720	-5.51330
O	2.46750	0.62790	-4.66920
C	6.49050	7.41260	-4.54250
C	7.56390	7.13920	-3.70420
C	7.52530	6.00570	-2.89470
H	6.39140	4.26620	-2.31220
H	4.54480	6.81840	-5.22070
H	6.50320	8.29470	-5.17050
H	8.41900	7.80180	-3.67690
H	8.35370	5.77950	-2.23500

Phenyl β-D-Galf **6**, C1-endo, 180°/-60°

	**X**	**Y**	**Z**
C	3.67320	5.98950	-2.61950
O	2.77180	6.03070	-3.69460
C	3.20140	4.83490	-1.73820
C	1.70740	4.69780	-2.07940
C	1.46480	5.76390	-3.16490
O	3.60060	7.15040	-1.81320
H	4.67660	5.85400	-3.02040
O	0.89420	4.97480	-0.93920
C	4.05440	8.34500	-2.31780
C	3.99410	9.42560	-1.43840
C	4.55400	8.51550	-3.60680
H	3.35520	5.02790	-0.68080
O	3.94100	3.67680	-2.14370
C	3.89810	2.61330	-1.31170
C	0.01730	4.03290	-0.52480
H	1.50070	3.69340	-2.43950
C	0.55100	5.34920	-4.30510
C	-0.80260	4.85010	-3.82730
H	1.02780	4.52060	-4.84300
O	0.42470	6.48760	-5.15220
O	-1.36180	5.84340	-2.94850

H	-1.46690	4.70480	-4.68080
H	-0.71370	3.90260	-3.29690
H	-0.04650	6.22460	-5.95280
H	1.06720	6.66510	-2.69340
C	-2.48920	5.52680	-2.29250
C	-2.91820	6.60020	-1.36160
C	-2.24060	7.81950	-1.28110
C	-2.66440	8.78890	-0.38350
C	-3.75980	8.54600	0.44030
C	-4.43390	7.33100	0.36430
C	-4.01660	6.36130	-0.53510
H	-1.38550	8.00210	-1.91540
H	-2.13730	9.73230	-0.32200
H	-4.08410	9.30120	1.14510
H	-5.27900	7.13730	1.01190
H	-4.52550	5.40970	-0.59630
C	-0.81270	4.50440	0.60700
C	-1.79480	3.64500	1.10440
C	-2.61730	4.05690	2.14190
C	-2.46410	5.32860	2.68690
C	-1.48710	6.18750	2.19380
C	-0.66240	5.78030	1.15580
H	-1.90830	2.66390	0.66480
H	-3.38150	3.39120	2.52140
H	-3.11290	5.65380	3.49030
H	-1.37750	7.18040	2.60910
H	0.08650	6.45100	0.76190
C	4.68100	1.46240	-1.82090
C	5.36220	1.51010	-3.04010
C	4.72340	0.30060	-1.04640
C	5.44080	-0.80230	-1.48560
C	6.11910	-0.75180	-2.70070
C	6.07860	0.40360	-3.47590
H	5.32860	2.40650	-3.64190
H	4.19120	0.27410	-0.10550

H	5.47150	-1.70070	-0.88290
H	6.67850	-1.61310	-3.04350
H	6.60520	0.44210	-4.42060
O	-3.06670	4.47130	-2.44490
O	-0.08580	2.94620	-1.04840
O	3.27870	2.62490	-0.27140
C	5.00430	9.77440	-3.99910
C	4.95210	10.85610	-3.12920
C	4.44170	10.67380	-1.84550
H	3.59930	9.26910	-0.44280
H	4.58220	7.69620	-4.30880
H	5.39230	9.90260	-5.00190
H	5.30070	11.83030	-3.44620
H	4.39320	11.50690	-1.15560

Phenyl β-D-Galf **6**, C1-endo, 180°/180°

	**X**	**Y**	**Z**
C	2.21550	5.02840	-7.21150
O	1.89370	5.95110	-6.20160
C	0.87480	4.59800	-7.81340
C	-0.14930	4.94740	-6.72360
C	0.69830	5.48650	-5.56060
O	2.82020	3.85950	-6.69420
H	2.87360	5.52260	-7.92510
O	-0.90600	3.82890	-6.25450
C	4.10850	3.92940	-6.22130
C	4.84190	5.10880	-6.11290
C	4.67330	2.71250	-5.84110
H	0.87280	3.54400	-8.06940
O	0.59120	5.38920	-8.97080
C	0.77850	4.82020	-10.17980
C	-1.85680	3.35640	-7.08920
H	-0.83140	5.70280	-7.10760
C	0.09160	6.63100	-4.76790

C	-1.16630	6.22390	-4.00680
H	-0.13530	7.45560	-5.45250
O	1.07250	7.02230	-3.81150
O	-2.25300	6.06810	-4.93440
H	-1.00670	5.29110	-3.46660
H	-1.42010	7.00420	-3.28750
H	0.79870	7.85900	-3.41500
H	0.93820	4.66610	-4.87620
C	-3.35250	5.44760	-4.47140
C	-4.37390	5.23120	-5.52440
C	-4.23380	5.75540	-6.81080
C	-5.19850	5.49380	-7.77370
C	-6.30170	4.70630	-7.45970
C	-6.44490	4.18420	-6.17680
C	-5.48720	4.44860	-5.21080
H	-3.36820	6.35170	-7.05850
H	-5.08500	5.89710	-8.77150
H	-7.04750	4.49520	-8.21560
H	-7.29750	3.56320	-5.93500
H	-5.57870	4.03800	-4.21530
C	-2.75490	2.37910	-6.43430
C	-3.78380	1.81900	-7.19340
C	-4.68250	0.94240	-6.60530
C	-4.55970	0.62200	-5.25620
C	-3.53360	1.17680	-4.49660
C	-2.63090	2.05300	-5.08150
H	-3.87720	2.08970	-8.23570
H	-5.48430	0.51640	-7.19420
H	-5.26600	-0.05710	-4.79540
H	-3.44170	0.93190	-3.44640
H	-1.84310	2.49620	-4.49040
C	0.36380	5.71260	-11.28970
C	-0.17160	6.98260	-11.06010
C	0.51690	5.25050	-12.59900
C	0.13840	6.04940	-13.66790

C	-0.39480	7.31470	-13.43600
C	-0.54850	7.77930	-12.13290
H	-0.29100	7.34190	-10.04850
H	0.93210	4.26600	-12.76520
H	0.25820	5.68750	-14.68090
H	-0.69030	7.93820	-14.27040
H	-0.96320	8.76270	-11.95260
O	-3.46920	5.09230	-3.31790
O	-1.95260	3.72570	-8.23850
O	1.23490	3.70820	-10.32460
C	5.97460	2.67460	-5.36220
C	6.72300	3.84550	-5.25890
C	6.14980	5.05330	-5.63570
H	4.40830	6.06200	-6.37430
H	4.08320	1.80970	-5.93240
H	6.40650	1.72510	-5.07160
H	7.73820	3.81480	-4.88530
H	6.71680	5.97220	-5.55360

Phenyl β-D-Galf **6**, C2-exo, +60°/+60°

	**X**	**Y**	**Z**
O	-2.06570	1.81060	1.26410
O	-3.79650	6.67490	0.09930
O	0.17530	4.19330	-0.26040
O	-3.19460	3.23700	-1.18950
O	-1.90410	4.01500	1.75740
O	-1.65450	8.14270	1.28160
C	-0.97570	3.34580	-0.26210
C	-2.24780	4.13530	-0.60010
C	-2.77810	4.59660	0.76930
C	-2.84150	6.10940	0.99100
C	-1.21120	2.94800	1.19520
C	-1.52210	6.80820	0.75580
H	-0.83120	2.50740	-0.93610

H	-2.05370	4.95160	-1.28750
H	-3.13880	6.27580	2.03150
H	-0.29690	2.78870	1.76590
H	-3.78720	4.19870	0.90020
H	-1.28680	6.86330	-0.30630
H	-0.71270	6.29640	1.27310
C	0.77810	4.41430	-1.45190
C	-3.55730	3.42650	-2.47680
C	-0.65290	8.99560	1.02390
C	-1.58880	0.57920	0.89960
C	-0.88720	10.34480	1.60120
C	-2.05560	10.65960	2.29950
C	-2.23060	11.93570	2.81820
C	-1.24390	12.90200	2.64430
C	-0.07820	12.59130	1.94890
C	0.10000	11.31750	1.42870
H	-2.82170	9.90940	2.43200
H	-3.13730	12.17720	3.35780
H	-1.38310	13.89620	3.04990
H	0.68980	13.34180	1.81310
H	1.00030	11.06330	0.88640
C	1.96200	5.29510	-1.32800
C	2.39230	5.78740	-0.09340
C	2.65620	5.63440	-2.49220
C	3.76940	6.45830	-2.42200
C	4.19660	6.94650	-1.18950
C	3.50800	6.61020	-0.02790
H	-4.68290	6.42010	0.38520
H	1.85260	5.53220	0.80650
H	2.31330	5.24890	-3.44250
H	4.30390	6.72140	-3.32560
H	5.06520	7.59070	-1.13530
H	3.83740	6.99360	0.92900
C	-4.44970	2.34320	-2.95800
C	-4.71040	1.20630	-2.18810

C	-5.53840	0.20910	-2.68490
C	-6.11290	0.34230	-3.94590
C	-5.85530	1.47450	-4.71490
C	-5.02380	2.47090	-4.22480
H	-4.26060	1.10190	-1.21190
H	-5.73410	-0.67290	-2.08880
H	-6.75970	-0.43630	-4.33020
H	-6.30140	1.57800	-5.69550
H	-4.81270	3.35320	-4.81340
O	0.37240	3.93230	-2.48500
O	-3.19390	4.36950	-3.14180
O	0.33210	8.68600	0.38680
C	-2.54560	-0.43220	0.80670
C	-2.16200	-1.71640	0.45210
C	-0.82510	-2.00600	0.18430
C	0.12090	-0.99410	0.27910
C	-0.24870	0.30180	0.63460
H	-3.58090	-0.19300	1.01270
H	-2.91200	-2.49410	0.38070
H	-0.52780	-3.00800	-0.09590
H	1.16300	-1.20330	0.07270
H	0.50910	1.06920	0.69570

Phenyl β-D-Galf **6**, C2-exo, +60°/-60°

	**X**	**Y**	**Z**
O	-1.55740	1.98290	0.69400
O	-4.31080	6.49790	1.03010
O	-1.91580	3.87710	-2.27160
O	-4.49840	3.01110	0.09060
O	-1.41230	4.31630	0.57130
O	-3.28260	6.73680	-1.51270
C	-2.42000	3.13660	-1.15650
C	-3.53140	3.92250	-0.44370
C	-2.80640	4.63400	0.71650

C	-2.94840	6.15470	0.80980
C	-1.29790	3.10420	-0.12680
C	-2.43580	6.95480	-0.36920
H	-2.74810	2.14940	-1.46620
H	-4.02350	4.60990	-1.11640
H	-2.32850	6.47020	1.65760
H	-0.29960	3.05320	-0.55950
H	-3.18040	4.21050	1.65340
H	-1.41560	6.65770	-0.61000
H	-2.44660	8.01490	-0.11940
C	-2.58880	3.75740	-3.43790
C	-5.77330	3.10440	-0.34370
C	-3.05020	7.49970	-2.59220
C	-0.65570	1.64260	1.67280
C	-3.99450	7.21900	-3.70210
C	-5.05530	6.32040	-3.56350
C	-5.89710	6.07360	-4.63870
C	-5.68950	6.72210	-5.85240
C	-4.63720	7.62240	-5.99050
C	-3.79230	7.87050	-4.91970
H	-5.22260	5.81230	-2.62560
H	-6.71320	5.37110	-4.52920
H	-6.34490	6.52300	-6.69110
H	-4.46900	8.12070	-6.93640
H	-2.96200	8.55530	-5.01880
C	-2.00240	4.58870	-4.51330
C	-0.90890	5.42760	-4.28560
C	-2.58630	4.53520	-5.78040
C	-2.08110	5.31270	-6.81020
C	-0.99270	6.14960	-6.58090
C	-0.40900	6.20670	-5.31910
H	-4.57680	6.17450	1.90010
H	-0.46370	5.47860	-3.30290
H	-3.44100	3.89350	-5.94100
H	-2.54240	5.27700	-7.78830

H	-0.60430	6.76320	-7.38390
H	0.43160	6.86390	-5.13840
C	-6.64770	2.08210	0.28280
C	-6.15440	1.14110	1.19070
C	-7.01200	0.20310	1.74940
C	-8.36120	0.19840	1.40680
C	-8.85530	1.13450	0.50190
C	-8.00200	2.07350	-0.05900
H	-5.10700	1.14510	1.45480
H	-6.62800	-0.52510	2.45200
H	-9.02740	-0.53450	1.84420
H	-9.90410	1.13110	0.23470
H	-8.37240	2.80580	-0.76310
O	-3.55970	3.04460	-3.56000
O	-6.14830	3.93030	-1.14550
O	-2.15790	8.32050	-2.63780
C	-0.96430	0.48910	2.39320
C	-0.11070	0.05140	3.39510
C	1.05450	0.75730	3.68780
C	1.35070	1.90750	2.96750
C	0.50120	2.36270	1.96120
H	-1.87020	-0.05220	2.15230
H	-0.35660	-0.84650	3.94810
H	1.71960	0.41510	4.46970
H	2.24950	2.46970	3.18830
H	0.74070	3.27140	1.43020

Phenyl β-D-Galf **6**, C2-exo, +60°/180°

	**X**	**Y**	**Z**
O	-3.97630	4.08560	4.37420
O	-3.00720	5.73170	-0.62760
O	-0.62530	4.64720	3.40050
O	-3.18440	2.83820	1.60490
O	-3.28280	5.86440	3.02180

O	-1.23230	7.51740	1.92790
C	-1.90000	4.00800	3.27540
C	-2.38760	4.00910	1.81990
C	-3.27450	5.26370	1.71620
C	-2.85450	6.30020	0.66880
C	-2.90080	4.91640	3.97930
C	-1.41080	6.77580	0.71600
H	-1.86520	3.00530	3.69080
H	-1.56330	4.00740	1.11610
H	-3.50430	7.17300	0.79380
H	-2.48330	5.44500	4.83570
H	-4.28900	4.94120	1.46320
H	-1.22410	7.41900	-0.14390
H	-0.70070	5.94900	0.68350
C	0.45460	3.91670	3.04340
C	-2.87840	2.02890	0.56790
C	-0.02610	8.05340	2.15040
C	-4.98660	4.62170	5.13390
C	-5.97950	3.72470	5.52680
C	-5.05780	5.95970	5.51580
C	0.05460	8.69760	3.48570
C	-0.95440	8.52900	4.43750
C	-0.82210	9.10310	5.69450
C	0.30920	9.85340	6.00370
C	1.31190	10.02890	5.05340
C	1.18760	9.44890	3.79930
H	-1.82600	7.93910	4.19340
H	-1.60010	8.96370	6.43410
H	0.41010	10.30010	6.98480
H	2.19160	10.61180	5.29370
H	1.96620	9.56390	3.05790
C	1.72530	4.63980	3.27720
C	1.78860	5.77280	4.09040
C	2.88480	4.15290	2.66880
C	4.09460	4.80240	2.86300

C	4.15510	5.93210	3.67590
C	3.00360	6.41290	4.29110
H	-3.94200	5.54550	-0.78130
H	0.89380	6.14790	4.56360
H	2.82360	3.27410	2.04150
H	4.98990	4.43070	2.38180
H	5.10040	6.43750	3.82850
H	3.04860	7.29090	4.92110
C	-3.79380	0.86320	0.49000
C	-4.83090	0.67290	1.40720
C	-5.66030	-0.43460	1.29390
C	-5.46030	-1.35550	0.26930
C	-4.42790	-1.16820	-0.64610
C	-3.59680	-0.06290	-0.53690
H	-4.98460	1.38770	2.20250
H	-6.46290	-0.58000	2.00540
H	-6.10870	-2.21840	0.18400
H	-4.27190	-1.88370	-1.44310
H	-2.79110	0.09370	-1.24080
O	0.36390	2.80130	2.58170
O	-1.96740	2.24130	-0.19950
O	0.88430	8.00110	1.35040
C	-6.12340	6.38420	6.30670
C	-7.11460	5.49720	6.70730
C	-7.03680	4.16380	6.31020
H	-5.90420	2.69090	5.21500
H	-4.31210	6.67270	5.19810
H	-6.17450	7.42470	6.60210
H	-7.94000	5.83880	7.31820
H	-7.80220	3.46020	6.61270

Phenyl β-D-Galf **6**, C2-exo, -60°/+60°

	**X**	**Y**	**Z**
O	-1.65710	1.94170	0.46380

O	-2.62350	6.79600	-0.32290
O	-1.94840	4.16590	-2.27220
O	-4.55030	2.98910	-0.11470
O	-1.50420	4.27390	0.60250
O	-2.58030	7.99670	2.26130
C	-2.47790	3.29970	-1.26490
C	-3.60120	3.99700	-0.48970
C	-2.89800	4.61090	0.73930
C	-3.03520	6.12490	0.86600
C	-1.37920	3.14800	-0.22060
C	-2.19200	6.63180	2.01610
H	-2.79800	2.35630	-1.69620
H	-4.09920	4.74420	-1.09950
H	-4.08670	6.33300	1.07580
H	-0.37250	3.13910	-0.63690
H	-3.29500	4.14260	1.64400
H	-1.13310	6.59590	1.76490
H	-2.36440	6.04120	2.91650
C	-2.66140	4.28370	-3.41220
C	-5.74400	3.42040	0.33510
C	-1.90010	8.65650	3.20860
C	-0.77590	1.49470	1.41770
C	-2.36530	10.05840	3.37480
C	-3.37880	10.60090	2.58080
C	-3.77940	11.91680	2.77130
C	-3.17350	12.69630	3.75250
C	-2.16270	12.15880	4.54520
C	-1.75920	10.84470	4.35710
H	-3.84720	9.99510	1.81870
H	-4.56440	12.33470	2.15430
H	-3.48820	13.72190	3.89920
H	-1.69060	12.76410	5.30820
H	-0.97500	10.41540	4.96540
C	-2.06080	5.25780	-4.35350
C	-0.95780	6.04060	-4.00230

C	-2.63640	5.39450	-5.61890
C	-2.11100	6.30190	-6.52690
C	-1.01180	7.08110	-6.17460
C	-0.43830	6.95070	-4.91290
H	-3.39910	7.00890	-0.85330
H	-0.51780	5.94190	-3.02070
H	-3.49150	4.78540	-5.87830
H	-2.55720	6.40370	-7.50770
H	-0.60340	7.79080	-6.88290
H	0.41360	7.55910	-4.63840
C	-6.65450	2.30400	0.68380
C	-6.25450	0.96810	0.59040
C	-7.14120	-0.04440	0.93070
C	-8.42690	0.26890	1.36260
C	-8.82770	1.59920	1.45690
C	-7.94450	2.61440	1.12020
H	-5.25610	0.72680	0.25590
H	-6.82960	-1.07840	0.85880
H	-9.11630	-0.52330	1.62630
H	-9.82720	1.84300	1.79280
H	-8.24270	3.65150	1.18990
O	-3.67730	3.65620	-3.61570
O	-6.01840	4.59720	0.43580
O	-1.00500	8.15140	3.85410
C	-1.09300	0.26540	1.99490
C	-0.25940	-0.28020	2.96010
C	0.89420	0.39250	3.35810
C	1.19900	1.61850	2.78060
C	0.36940	2.18190	1.81320
H	-1.99020	-0.24810	1.67390
H	-0.51190	-1.23610	3.40170
H	1.54370	-0.03390	4.11140
H	2.08840	2.15590	3.08510
H	0.61460	3.14660	1.39570

Phenyl β-D-Galf **6**, C2-exo, -60°/-60°

	X	Y	Z
O	-1.76980	-2.15570	-1.46450
O	0.93620	1.96760	-0.97870
O	1.41120	-0.82430	-2.55320
O	-1.92040	-0.12640	-3.68520
O	-0.68340	-0.35890	-0.61880
O	-0.75040	1.74260	1.26480
C	0.01380	-1.08450	-2.70720
C	-0.75270	0.21870	-2.92790
C	-1.11330	0.69650	-1.50190
C	-0.47860	2.03510	-1.10780
C	-0.49360	-1.52990	-1.33650
C	-1.04050	2.62440	0.16850
H	-0.15750	-1.80030	-3.50440
H	-0.16890	0.94730	-3.48530
H	-0.76050	2.73790	-1.89970
H	0.20170	-2.16560	-0.78870
H	-2.19860	0.80080	-1.43370
H	-2.11980	2.75310	0.08020
H	-0.58300	3.59560	0.35810
C	2.14480	-0.71430	-3.68400
C	-2.65240	0.88580	-4.18600
C	-1.22960	2.07650	2.46770
C	-1.87400	-3.38650	-2.05450
C	-0.90920	1.05510	3.49900
C	-0.27820	-0.14690	3.16690
C	-0.00420	-1.08090	4.15720
C	-0.35420	-0.82070	5.47930
C	-0.98290	0.37630	5.81240
C	-1.26160	1.31110	4.82540
H	-0.01290	-0.34520	2.13820
H	0.48150	-2.01280	3.89790
H	-0.13830	-1.55050	6.24940

H	-1.25530	0.57900	6.84020
H	-1.75130	2.24360	5.07080
C	3.55930	-0.37670	-3.40200
C	4.00590	-0.09050	-2.10890
C	4.45440	-0.33210	-4.47360
C	5.78510	-0.00860	-4.25400
C	6.22780	0.27700	-2.96470
C	5.33790	0.23720	-1.89500
H	1.34010	1.88820	-1.85070
H	3.31250	-0.11670	-1.28100
H	4.09670	-0.55260	-5.47000
H	6.47720	0.02260	-5.08540
H	7.26620	0.53170	-2.79420
H	5.68180	0.46250	-0.89390
C	-3.85310	0.40200	-4.90800
C	-4.18110	-0.95580	-4.96100
C	-5.32240	-1.36510	-5.63670
C	-6.13760	-0.42630	-6.26300
C	-5.81250	0.92690	-6.21200
C	-4.67460	1.34130	-5.53510
H	-3.55000	-1.68340	-4.47210
H	-5.57630	-2.41660	-5.67380
H	-7.02690	-0.74870	-6.78980
H	-6.44660	1.65660	-6.69850
H	-4.41240	2.38930	-5.48540
O	1.67260	-0.87230	-4.78710
O	-2.34480	2.04980	-4.04250
O	-1.85480	3.09600	2.67700
C	-3.17070	-3.79490	-2.36950
C	-3.38210	-5.03150	-2.96000
C	-2.30700	-5.87130	-3.24580
C	-1.01960	-5.45750	-2.92980
C	-0.79090	-4.21820	-2.33510
H	-3.99610	-3.13080	-2.14830
H	-4.39180	-5.33850	-3.20240

H	-2.47330	-6.83430	-3.71040
H	-0.17430	-6.09840	-3.14690
H	0.22150	-3.92100	-2.10280

Phenyl β-D-Galf **6**, C2-exo, -60°/180°

	**X**	**Y**	**Z**
O	-5.71650	3.89440	1.49640
O	-2.62620	7.10410	-0.87640
O	-4.11040	3.91770	-1.65830
O	-6.70080	6.10520	-0.54290
O	-3.78900	5.10500	0.96030
O	-3.29310	7.64840	2.62920
C	-5.13530	4.29820	-0.73520
C	-5.30480	5.82440	-0.71420
C	-4.47570	6.28360	0.50450
C	-3.45150	7.38280	0.25180
C	-4.58260	3.99720	0.65270
C	-2.50100	7.52730	1.43450
H	-6.06450	3.78190	-0.95630
H	-4.96170	6.26740	-1.64380
H	-4.00520	8.31050	0.09950
H	-3.96230	3.10310	0.70800
H	-5.16450	6.62000	1.28230
H	-1.89160	8.42370	1.32340
H	-1.85430	6.65710	1.50550
C	-4.44840	3.87970	-2.96450
C	-7.09630	7.37170	-0.76790
C	-2.84050	7.07080	3.75480
C	-5.53600	3.79870	2.85610
C	-6.69870	3.89210	3.61900
C	-4.30150	3.61900	3.47510
C	-3.82880	7.14410	4.86010
C	-5.16530	7.47780	4.63200
C	-6.06180	7.50780	5.69150

C	-5.62860	7.21780	6.98140
C	-4.29590	6.88770	7.21200
C	-3.40030	6.84380	6.15420
H	-5.50090	7.69830	3.62920
H	-7.10040	7.75250	5.50980
H	-6.33010	7.24320	7.80580
H	-3.95860	6.65830	8.21460
H	-2.36520	6.57720	6.31770
C	-3.30170	3.52140	-3.83240
C	-2.01190	3.35470	-3.32100
C	-3.53380	3.35960	-5.20030
C	-2.48710	3.02960	-6.04860
C	-1.20270	2.86360	-5.53650
C	-0.96690	3.02770	-4.17450
H	-2.96040	7.59210	-1.63740
H	-1.82980	3.48690	-2.26450
H	-4.53560	3.49350	-5.58490
H	-2.67040	2.90250	-7.10760
H	-0.38570	2.60740	-6.19920
H	0.03200	2.90140	-3.77740
C	-8.54900	7.56080	-0.54100
C	-9.37030	6.51840	-0.10310
C	-10.72460	6.74460	0.10140
C	-11.26480	8.00660	-0.12970
C	-10.44840	9.04720	-0.56540
C	-9.09430	8.82620	-0.76980
H	-8.94890	5.54020	0.07730
H	-11.35930	5.93650	0.44130
H	-12.32160	8.17950	0.03020
H	-10.86800	10.02860	-0.74480
H	-8.44890	9.62570	-1.10680
O	-5.56920	4.11860	-3.35680
O	-6.33000	8.24590	-1.11320
O	-1.74740	6.55480	3.84860
C	-4.24570	3.53400	4.86370

C	-5.39810	3.62420	5.63260
C	-6.62630	3.80220	5.00140
H	-7.64520	4.04070	3.11520
H	-3.39000	3.57040	2.90010
H	-3.28220	3.41260	5.34210
H	-5.34010	3.57420	6.71160
H	-7.53280	3.88390	5.58770

Phenyl β-D-Galf **6**, C2-exo, 180°/+60°

	**X**	**Y**	**Z**
O	-2.95100	1.54240	1.46440
O	-4.36650	6.37650	0.99190
O	-1.07010	4.32850	2.57660
O	-1.46060	2.86920	-0.70500
O	-3.67170	3.76330	1.33320
O	-1.98920	8.07110	0.29010
C	-1.39460	3.30450	1.63020
C	-1.56630	3.93390	0.24610
C	-2.98590	4.50780	0.29810
C	-3.02990	5.98500	0.69870
C	-2.80840	2.84880	1.97280
C	-2.49670	6.90010	-0.38230
H	-0.66400	2.50180	1.64740
H	-0.80820	4.68300	0.03980
H	-2.40740	6.11160	1.58800
H	-3.01600	2.86770	3.04290
H	-3.51430	4.34970	-0.64290
H	-3.29120	7.18290	-1.07290
H	-1.68950	6.44000	-0.94890
C	0.20170	4.79360	2.58060
C	-1.27490	3.21550	-1.99470
C	-1.36770	8.99020	-0.46020
C	-4.08420	0.82700	1.76310
C	-0.74840	10.06230	0.36220

C	-0.62210	9.94480	1.74890
C	-0.01500	10.95920	2.47680
C	0.46220	12.09500	1.82910
C	0.33850	12.21420	0.44720
C	-0.26080	11.19940	-0.28510
H	-0.98470	9.06000	2.25130
H	0.08840	10.86130	3.54920
H	0.93290	12.88550	2.40010
H	0.71040	13.09650	-0.05750
H	-0.35850	11.27740	-1.35930
C	0.35460	5.99280	3.43660
C	-0.72210	6.54580	4.13570
C	1.61240	6.59690	3.50720
C	1.79200	7.74080	4.27040
C	0.71840	8.28680	4.96870
C	-0.53660	7.68970	4.89980
H	-4.69680	5.78100	1.67830
H	-1.69760	6.08580	4.07720
H	2.43700	6.16540	2.95680
H	2.76640	8.20900	4.31930
H	0.85890	9.18130	5.56200
H	-1.37230	8.11830	5.43740
C	-1.22810	2.03510	-2.88920
C	-1.46340	0.74190	-2.41390
C	-1.41810	-0.33450	-3.28920
C	-1.13640	-0.12770	-4.63670
C	-0.90230	1.16000	-5.11250
C	-0.95000	2.23950	-4.24260
H	-1.68450	0.58450	-1.36820
H	-1.60310	-1.33520	-2.92060
H	-1.10060	-0.96960	-5.31650
H	-0.68390	1.32060	-6.16030
H	-0.77240	3.24470	-4.59960
O	1.08390	4.28220	1.92990
O	-1.16440	4.36750	-2.35380

O	-1.31700	8.93520	-1.67120
C	-4.09120	-0.49410	1.31660
C	-5.18640	-1.30460	1.57630
C	-6.28110	-0.80750	2.28090
C	-6.26680	0.51150	2.71630
C	-5.17640	1.34000	2.45860
H	-3.23230	-0.86820	0.77430
H	-5.18340	-2.32990	1.22800
H	-7.13510	-1.44070	2.48270
H	-7.11410	0.91350	3.25770
H	-5.19800	2.36900	2.78470

Phenyl β-D-Galf **6**, C2-exo, 180°/-60°

	**X**	**Y**	**Z**
O	-5.64170	3.12650	1.42980
O	-3.06250	6.96540	2.69620
O	-2.22800	2.74300	0.32460
O	-4.84480	4.78640	-1.07370
O	-3.85910	4.34910	2.10490
O	-3.96090	7.95070	0.06760
C	-3.59160	3.13870	0.14820
C	-3.68230	4.61370	-0.25690
C	-3.86500	5.36640	1.07630
C	-2.77810	6.37320	1.43160
C	-4.22140	3.13230	1.54020
C	-2.64300	7.48290	0.41060
H	-4.08710	2.49130	-0.56860
H	-2.80120	4.92490	-0.80890
H	-1.81510	5.84860	1.46840
H	-3.87040	2.33020	2.18780
H	-4.83510	5.86150	1.07560
H	-2.15350	7.12430	-0.49320
H	-2.05610	8.29940	0.83000
C	-1.54520	2.39420	-0.78950

C	-4.75980	5.58690	-2.15750
C	-4.04860	8.83650	-0.93680
C	-6.28790	1.99090	1.01760
C	-5.45230	9.08320	-1.35120
C	-6.52220	8.39150	-0.77890
C	-7.81480	8.61640	-1.23080
C	-8.04700	9.53160	-2.25190
C	-6.98380	10.22680	-2.82180
C	-5.69040	10.00230	-2.37480
H	-6.33960	7.67020	0.00400
H	-8.63930	8.06730	-0.79560
H	-9.05530	9.69800	-2.60950
H	-7.16380	10.93620	-3.61920
H	-4.85420	10.52680	-2.81640
C	-0.14550	2.00840	-0.49510
C	0.37170	2.03240	0.80310
C	0.66800	1.61530	-1.56060
C	1.98570	1.24850	-1.33030
C	2.49840	1.27270	-0.03570
C	1.69120	1.66470	1.02840
H	-3.16700	6.25530	3.34320
H	-0.25560	2.33810	1.62770
H	0.25760	1.60200	-2.56090
H	2.61350	0.94440	-2.15770
H	3.52710	0.98670	0.14350
H	2.09040	1.68400	2.03420
C	-6.06390	5.72370	-2.84610
C	-7.19280	5.00830	-2.43890
C	-8.40130	5.19430	-3.09500
C	-8.49010	6.09540	-4.15230
C	-7.36620	6.80950	-4.55820
C	-6.15540	6.62250	-3.91000
H	-7.12400	4.31610	-1.61240
H	-9.27550	4.63970	-2.77900
H	-9.43640	6.24490	-4.65660

H	-7.43760	7.51820	-5.37270
H	-5.27860	7.18140	-4.20490
O	-2.04640	2.40770	-1.89120
O	-3.73540	6.13370	-2.50010
O	-3.07810	9.35500	-1.44590
C	-7.64070	2.14700	0.71330
C	-8.38680	1.05380	0.30020
C	-7.79410	-0.20230	0.18210
C	-6.44670	-0.34850	0.48420
C	-5.68340	0.74020	0.90180
H	-8.08670	3.12900	0.80410
H	-9.43560	1.18470	0.06510
H	-8.37690	-1.05370	-0.14400
H	-5.97190	-1.31750	0.39460
H	-4.63630	0.59630	1.12550

Phenyl β-D-Galf **6**, C2-exo, 180°/180°

	**X**	**Y**	**Z**
O	0.19400	3.86080	2.87710
O	-4.00810	6.18140	1.33300
O	-0.84950	6.88720	4.40050
O	0.80180	6.32090	1.25150
O	-1.82900	4.93460	2.41070
O	-2.11470	9.19730	1.39600
C	-0.10390	6.12640	3.44450
C	-0.30920	6.73580	2.05800
C	-1.61670	6.09520	1.57120
C	-2.87140	6.96040	1.69110
C	-0.78410	4.76800	3.33770
C	-2.87300	8.15910	0.75650
H	0.94160	6.06050	3.72700
H	-0.35400	7.81880	2.08880
H	-2.96410	7.31510	2.72330
H	-1.20980	4.42480	4.28070

H	-1.51390	5.75900	0.53770
H	-3.89820	8.49900	0.60880
H	-2.43180	7.92380	-0.21100
C	-0.24180	7.98510	4.90820
C	1.03590	7.02840	0.12800
C	-1.73520	10.24090	0.64260
C	-0.10830	2.52250	2.82660
C	0.94280	1.68610	2.45190
C	-1.36080	1.98830	3.12040
C	-0.89290	11.19990	1.40180
C	-0.70220	11.08040	2.78050
C	0.09390	11.99640	3.45350
C	0.70800	13.03210	2.75520
C	0.52000	13.15470	1.38070
C	-0.28090	12.24430	0.70610
H	-1.18290	10.27980	3.32140
H	0.23520	11.90120	4.52180
H	1.33200	13.74350	3.28140
H	0.99790	13.95940	0.83690
H	-0.43500	12.32910	-0.36090
C	-1.14860	8.78860	5.75870
C	-2.51920	8.52680	5.83100
C	-0.60590	9.86150	6.46990
C	-1.42600	10.66540	7.24690
C	-2.79260	10.40550	7.31390
C	-3.33690	9.33770	6.60600
H	-3.96910	5.36180	1.84440
H	-2.94050	7.70180	5.27550
H	0.45550	10.05620	6.40210
H	-1.00290	11.49560	7.79730
H	-3.43330	11.03650	7.91670
H	-4.39960	9.13880	6.65580
C	2.18610	6.49690	-0.64100
C	2.85810	5.33390	-0.25490
C	3.92550	4.86940	-1.01140

C	4.32800	5.56130	-2.15030
C	3.65960	6.72030	-2.53700
C	2.59030	7.18650	-1.78630
H	2.54240	4.79720	0.62800
H	4.44360	3.96720	-0.71290
H	5.16160	5.19690	-2.73740
H	3.97220	7.25790	-3.42280
H	2.06110	8.08380	-2.07620
O	0.90980	8.26580	4.66580
O	0.36700	7.98510	-0.19540
O	-2.05290	10.37110	-0.51970
C	-1.54410	0.60880	3.05010
C	-0.50220	-0.23310	2.68270
C	0.74300	0.31530	2.38130
H	1.90600	2.12450	2.22480
H	-2.19210	2.62450	3.38460
H	-2.51920	0.19750	3.27950
H	-0.65690	-1.30270	2.62710
H	1.56490	-0.32710	2.09110

Phenyl β-D-Galf **6**, C3-exo, +60°/+60°

	**X**	**Y**	**Z**
C	3.64750	4.64450	-2.69000
O	3.68120	4.49430	-4.09480
C	2.17200	4.59650	-2.27920
C	1.42920	4.32570	-3.58880
C	2.40710	4.85900	-4.63640
O	4.15990	5.89480	-2.28280
H	4.23360	3.83810	-2.24990
O	0.18710	5.01890	-3.66710
C	5.52050	6.09250	-2.31490
H	1.86270	5.54990	-1.85760
O	1.99020	3.55700	-1.31530
C	0.96880	3.69330	-0.43990

C	-0.93630	4.32720	-3.37970
H	1.26200	3.25660	-3.69400
C	2.31530	4.30240	-6.05110
C	2.30090	2.78770	-6.10280
H	3.20580	4.65280	-6.58610
O	1.14180	4.85770	-6.63400
O	2.41150	2.44020	-7.49810
H	3.14130	2.36510	-5.55270
H	1.36830	2.38170	-5.71180
H	1.08760	4.55310	-7.54870
H	2.31040	5.94810	-4.69870
C	2.36270	1.13310	-7.80350
C	0.85400	2.53240	0.47400
C	-0.16990	2.54300	1.42430
C	1.72570	1.44250	0.40400
C	1.57200	0.37640	1.27990
C	0.55080	0.39110	2.22570
C	-0.32000	1.47550	2.29700
H	-0.84020	3.39030	1.46730
H	2.51620	1.43080	-0.33210
H	2.24810	-0.46690	1.22410
H	0.43300	-0.44250	2.90650
H	-1.11490	1.48670	3.03150
C	-2.15360	5.16560	-3.48660
C	-2.10190	6.49920	-3.90020
C	-3.27100	7.24340	-3.98340
C	-4.49290	6.66410	-3.65310
C	-4.54710	5.33550	-3.23960
C	-3.38190	4.58770	-3.15700
H	-1.15300	6.94740	-4.15610
H	-3.22930	8.27590	-4.30540
H	-5.40260	7.24770	-3.71760
H	-5.49680	4.88500	-2.98170
H	-3.40940	3.55540	-2.83640
C	2.46100	0.88340	-9.26440

C	2.58280	1.92510	-10.18780
C	2.67010	1.64220	-11.54430
C	2.63680	0.32260	-11.98620
C	2.51550	-0.71770	-11.06860
C	2.42800	-0.43910	-9.71240
H	2.60910	2.94900	-9.84440
H	2.76420	2.45110	-12.25730
H	2.70510	0.10510	-13.04470
H	2.48940	-1.74410	-11.41120
H	2.33350	-1.23800	-8.98980
O	2.25320	0.26600	-6.96330
O	-0.93180	3.15670	-3.07020
O	0.24340	4.66160	-0.42040
C	5.97220	7.25790	-1.69700
C	7.32910	7.54550	-1.67340
C	8.24500	6.67530	-2.26130
C	7.78470	5.51880	-2.87790
C	6.42440	5.22000	-2.91630
H	5.25060	7.92150	-1.23820
H	7.67230	8.45140	-1.18980
H	9.30330	6.90010	-2.24110
H	8.48450	4.83760	-3.34570
H	6.08740	4.32960	-3.42580

Phenyl β-D-Galf **6**, C3-exo, +60°/-60°

	**X**	**Y**	**Z**
C	-0.69580	5.18130	-3.77680
O	0.58500	4.70820	-4.03710
C	-1.56740	4.62360	-4.90030
C	-0.60680	4.60780	-6.09730
C	0.79490	4.66820	-5.46120
O	-0.64240	6.60280	-3.86370
H	-0.98910	4.85760	-2.77880
O	-0.82390	5.79100	-6.87520

C	-1.74030	7.34620	-3.51990
H	-2.45300	5.21610	-5.10630
O	-1.93350	3.29750	-4.51240
C	-2.98110	2.74150	-5.16250
C	-1.28860	5.65500	-8.13480
H	-0.75330	3.73600	-6.71880
C	1.75570	3.52120	-5.76170
C	1.29900	2.12580	-5.36870
H	2.64910	3.71110	-5.15300
O	2.05980	3.63210	-7.14530
O	0.22260	1.70090	-6.22300
H	2.13030	1.42840	-5.47550
H	0.96700	2.11340	-4.33000
H	2.70810	2.95640	-7.38040
H	1.27730	5.59190	-5.78690
C	-0.11780	0.40300	-6.17160
C	-3.25590	1.35940	-4.71030
C	-4.32600	0.67680	-5.29220
C	-2.46460	0.72480	-3.75000
C	-2.74290	-0.58340	-3.38070
C	-3.81030	-1.26030	-3.96260
C	-4.60200	-0.62830	-4.91760
H	-4.92170	1.17390	-6.04460
H	-1.63000	1.24830	-3.30740
H	-2.12270	-1.07760	-2.64440
H	-4.02130	-2.28320	-3.67700
H	-5.42510	-1.15870	-5.37800
C	-1.58040	6.97020	-8.75680
C	-1.51710	8.16070	-8.02720
C	-1.81710	9.36710	-8.64510
C	-2.17610	9.39300	-9.98980
C	-2.23910	8.20830	-10.71910
C	-1.94520	6.99980	-10.10460
H	-1.24290	8.13860	-6.98270
H	-1.77230	10.28730	-8.07710

H	-2.40830	10.33570	-10.46920
H	-2.51870	8.22800	-11.76450
H	-1.99400	6.07240	-10.65850
C	-1.22790	0.06820	-7.09750
C	-1.75920	1.00410	-7.98830
C	-2.81690	0.65010	-8.81380
C	-3.34650	-0.63580	-8.75860
C	-2.81380	-1.57200	-7.87720
C	-1.75800	-1.22210	-7.04950
H	-1.35520	2.00420	-8.03570
H	-3.23040	1.38040	-9.49720
H	-4.17570	-0.90720	-9.40000
H	-3.22930	-2.57020	-7.82820
H	-1.34530	-1.93510	-6.34990
O	0.42960	-0.39260	-5.43760
O	-1.44350	4.58480	-8.67920
O	-3.60390	3.32980	-6.01750
C	-2.87580	6.82990	-2.89750
C	-3.92920	7.68660	-2.58340
C	-3.86240	9.04050	-2.88370
C	-2.72250	9.54460	-3.50810
C	-1.66650	8.70520	-3.82780
H	-2.95870	5.78020	-2.65610
H	-4.80920	7.27960	-2.10120
H	-4.68720	9.69650	-2.63830
H	-2.65660	10.59750	-3.75180
H	-0.77800	9.08260	-4.31730

Phenyl β-D-Galf **6**, C3-exo, +60°/180°

	X	Y	Z
C	5.26140	4.78810	-5.32720
O	4.22130	4.27100	-6.10950
C	4.84000	4.54590	-3.88320
C	3.30970	4.66230	-3.94150

C	2.96920	4.44590	-5.42820
O	5.39890	6.18910	-5.46970
H	6.18260	4.27650	-5.60400
O	2.93480	5.98940	-3.55640
C	5.88070	6.69390	-6.65220
C	6.03470	8.07940	-6.69190
C	6.21200	5.91870	-7.76150
H	5.27610	5.25190	-3.18290
O	5.25000	3.21030	-3.57260
C	5.20890	2.84790	-2.27090
C	2.07790	6.14630	-2.52420
H	2.82320	3.95380	-3.28050
C	2.05220	3.26480	-5.74070
C	2.47080	1.90610	-5.18980
H	1.98530	3.18830	-6.83130
O	0.79490	3.62370	-5.17650
O	3.65000	1.48150	-5.88550
H	2.66930	1.93650	-4.11830
H	1.66620	1.18930	-5.36440
H	0.12570	2.99450	-5.47320
H	2.47310	5.34550	-5.79880
C	4.15550	0.28330	-5.56700
C	5.72040	1.47500	-2.05620
C	5.44820	0.84960	-0.83700
C	6.46630	0.80790	-3.02980
C	6.93440	-0.47520	-2.78420
C	6.65320	-1.09990	-1.57320
C	5.90870	-0.43690	-0.60010
H	4.86990	1.37610	-0.09000
H	6.68020	1.29190	-3.97070
H	7.51060	-0.98900	-3.54180
H	7.01340	-2.10380	-1.38660
H	5.68780	-0.92430	0.34070
C	1.81080	7.57920	-2.24430
C	2.41870	8.60280	-2.97640

C	2.13940	9.92890	-2.67480
C	1.25540	10.24030	-1.64560
C	0.64810	9.22240	-0.91470
C	0.92480	7.89590	-1.21210
H	3.10480	8.36000	-3.77460
H	2.61170	10.72010	-3.24260
H	1.03990	11.27550	-1.41300
H	-0.03930	9.46370	-0.11440
H	0.46080	7.09600	-0.65170
C	5.44490	0.02540	-6.25620
C	6.12230	1.04100	-6.93610
C	7.35060	0.77730	-7.52680
C	7.90280	-0.49850	-7.44970
C	7.22610	-1.51310	-6.77740
C	6.00250	-1.25120	-6.17820
H	5.69220	2.03110	-6.98440
H	7.87890	1.56720	-8.04510
H	8.86080	-0.70170	-7.91150
H	7.65560	-2.50470	-6.71670
H	5.47320	-2.02600	-5.64070
O	3.61720	-0.48350	-4.79640
O	1.59190	5.22270	-1.91220
O	4.79130	3.58010	-1.40230
C	6.71200	6.54390	-8.90200
C	6.87350	7.92270	-8.94940
C	6.52900	8.68740	-7.83650
H	5.76780	8.66100	-5.81890
H	6.07420	4.84800	-7.75780
H	6.96870	5.93750	-9.76170
H	7.25890	8.39810	-9.84180
H	6.64700	9.76360	-7.85800

Phenyl β-D-Galf **6**, C3-exo, -60°/+60°

	X	Y	Z
C	2.29440	4.21710	-2.14880
O	1.65510	3.09590	-1.57130
C	2.83600	3.76330	-3.50820
C	2.22420	2.37950	-3.69610
C	2.06700	1.90610	-2.25590
O	3.39110	4.65630	-1.37720
H	1.56080	5.01880	-2.23890
O	3.06960	1.47790	-4.40790
C	3.14300	5.35350	-0.21820
H	3.92120	3.69340	-3.47530
O	2.43910	4.69990	-4.51000
C	3.24090	4.83540	-5.58970
C	2.87970	1.36050	-5.74070
H	1.25670	2.45380	-4.18620
C	1.05480	0.79600	-2.05720
C	0.89760	0.46390	-0.58580
H	1.45380	-0.08600	-2.57360
O	-0.18370	1.18530	-2.63710
O	0.01180	-0.67230	-0.52600
H	1.85630	0.20200	-0.13630
H	0.45570	1.29610	-0.04000
H	-0.80070	0.44830	-2.54660
H	3.03480	1.56270	-1.87290
C	-0.33400	-1.11300	0.69440
C	2.69060	5.78940	-6.58130
C	3.41760	6.01220	-7.75320
C	1.47810	6.45390	-6.37920
C	1.00290	7.33440	-7.34160
C	1.73100	7.55460	-8.50730
C	2.93870	6.89220	-8.71230
H	4.35270	5.48980	-7.90050
H	0.91230	6.28040	-5.47560
H	0.06370	7.84840	-7.18300
H	1.35710	8.24110	-9.25650

H	3.50430	7.06180	-9.61930
C	3.83390	0.41220	-6.36270
C	4.81280	-0.25150	-5.61860
C	5.68620	-1.12720	-6.24930
C	5.58850	-1.34430	-7.62070
C	4.61480	-0.68310	-8.36510
C	3.74040	0.19300	-7.73920
H	4.88920	-0.08030	-4.55480
H	6.44410	-1.63990	-5.67130
H	6.27130	-2.02760	-8.10980
H	4.53930	-0.85060	-9.43170
H	2.98120	0.71460	-8.30540
C	-1.26550	-2.26900	0.64150
C	-1.72270	-2.79720	-0.56870
C	-2.59440	-3.87810	-0.56800
C	-3.01350	-4.43650	0.63620
C	-2.55940	-3.91270	1.84380
C	-1.68870	-2.83260	1.84730
H	-1.39670	-2.36380	-1.50300
H	-2.94720	-4.28500	-1.50680
H	-3.69320	-5.27920	0.63370
H	-2.88440	-4.34660	2.78060
H	-1.32880	-2.41690	2.77830
O	0.08130	-0.61080	1.71690
O	2.02340	1.96910	-6.34170
O	4.28550	4.23690	-5.71530
C	4.24920	5.97520	0.35900
C	4.09870	6.70090	1.53190
C	2.84820	6.81570	2.13550
C	1.75270	6.18980	1.55410
C	1.88900	5.45120	0.38070
H	5.21410	5.88450	-0.12300
H	4.96240	7.18230	1.97320
H	2.73200	7.38310	3.04960
H	0.77660	6.26390	2.01690

H	1.03010	4.94900	-0.03900

Phenyl β-D-Galf **6**, C3-exo, -60°/-60°

	**X**	**Y**	**Z**
C	0.04040	1.98860	-4.61970
O	0.98800	2.45700	-5.55810
C	-0.38380	3.19780	-3.78230
C	0.19550	4.37770	-4.55220
C	1.44630	3.75610	-5.16210
O	0.59440	1.02570	-3.75230
H	-0.79820	1.55570	-5.16560
O	0.56530	5.47360	-3.71730
C	0.74520	-0.26090	-4.21640
H	0.05970	3.14500	-2.78990
O	-1.80750	3.21900	-3.67830
C	-2.34430	3.79280	-2.57920
C	-0.33980	6.45960	-3.53540
H	-0.49300	4.70830	-5.32690
C	2.02220	4.52150	-6.34050
C	3.39600	4.03310	-6.78220
H	2.18010	5.54660	-5.97960
O	1.07000	4.50920	-7.39640
O	3.39420	2.65650	-7.20050
H	3.71550	4.59880	-7.65650
H	4.11460	4.17180	-5.97610
H	1.29640	5.20980	-8.01960
H	2.22300	3.66860	-4.39530
C	3.77740	1.72590	-6.30840
C	-3.82590	3.79410	-2.61360
C	-4.50840	4.35550	-1.53180
C	-4.54410	3.26350	-3.68830
C	-5.93200	3.29470	-3.67570
C	-6.60830	3.85370	-2.59520
C	-5.89510	4.38430	-1.52320

H	-3.94200	4.76560	-0.70710
H	-4.01810	2.83180	-4.52730
H	-6.48660	2.88340	-4.50930
H	-7.69080	3.87700	-2.58870
H	-6.42060	4.82020	-0.68340
C	0.16010	7.51910	-2.62750
C	1.42620	7.45550	-2.03950
C	1.84680	8.47040	-1.19070
C	1.00950	9.54990	-0.92400
C	-0.25320	9.61540	-1.50760
C	-0.67750	8.60360	-2.35630
H	2.07390	6.61580	-2.24480
H	2.82760	8.41890	-0.73590
H	1.34020	10.33940	-0.26100
H	-0.90510	10.45390	-1.29950
H	-1.65620	8.64100	-2.81430
C	3.71330	0.35040	-6.86140
C	3.19580	0.08560	-8.13100
C	3.16350	-1.21800	-8.60750
C	3.64730	-2.26020	-7.82280
C	4.15710	-1.99980	-6.55450
C	4.18800	-0.70000	-6.07380
H	2.81650	0.89600	-8.73630
H	2.75790	-1.42170	-9.59020
H	3.61700	-3.27640	-8.19530
H	4.52020	-2.81160	-5.93790
H	4.57440	-0.48500	-5.08730
O	4.15220	1.99030	-5.18550
O	-1.43010	6.46070	-4.06130
O	-1.67560	4.24890	-1.67920
C	1.10040	-1.20880	-3.25870
C	1.25470	-2.53770	-3.62820
C	1.05200	-2.93010	-4.94880
C	0.71130	-1.97520	-5.89730
C	0.56530	-0.63700	-5.54520

H	1.24380	-0.89110	-2.23400
H	1.52970	-3.26910	-2.87850
H	1.17560	-3.96600	-5.23660
H	0.57940	-2.26090	-6.93240
H	0.34150	0.09360	-6.30790

Phenyl β-D-Galf **6**, C3-exo, -60°/180°

	**X**	**Y**	**Z**
C	1.94400	8.00890	-4.54100
O	2.11350	6.62570	-4.62760
C	0.43980	8.21910	-4.67250
C	0.02450	7.10510	-5.64520
C	1.22980	6.14050	-5.65440
O	2.51330	8.68850	-5.64920
H	2.36900	8.35090	-3.59800
O	-0.17430	7.71150	-6.93030
C	3.82960	8.45510	-5.97380
C	4.21130	8.85950	-7.25110
C	4.75530	7.86580	-5.11650
H	0.16930	9.20560	-5.03650
O	-0.10930	7.99040	-3.37160
C	-1.39790	8.34540	-3.18330
C	-0.78780	6.97000	-7.87010
H	-0.88960	6.61480	-5.32570
C	0.92340	4.67880	-5.39120
C	2.20910	3.87380	-5.21530
H	0.36360	4.31160	-6.25360
O	0.14480	4.56100	-4.20370
O	3.03550	4.10710	-6.37100
H	2.73130	4.18760	-4.31600
H	1.98690	2.80800	-5.15290
H	-0.40680	3.77310	-4.27160
H	1.72790	6.22040	-6.62250
C	4.36440	4.20790	-6.19930

C	-1.87610	8.02930	-1.81650
C	-3.17070	8.41790	-1.46360
C	-1.07600	7.35400	-0.89090
C	-1.57070	7.07320	0.37550
C	-2.85990	7.46510	0.72460
C	-3.65940	8.13790	-0.19610
H	-3.78160	8.93790	-2.18860
H	-0.07730	7.04690	-1.16490
H	-0.95070	6.54800	1.09040
H	-3.24220	7.24540	1.71350
H	-4.66200	8.44230	0.07510
C	-0.88910	7.67730	-9.16960
C	-0.31660	8.93600	-9.37150
C	-0.42500	9.55450	-10.60970
C	-1.10410	8.92390	-11.64840
C	-1.67570	7.66970	-11.44970
C	-1.56750	7.04670	-10.21500
H	0.21260	9.42260	-8.56510
H	0.02090	10.52840	-10.76500
H	-1.18740	9.40920	-12.61270
H	-2.20370	7.17920	-12.25720
H	-2.00460	6.07180	-10.04810
C	5.05830	4.63550	-7.44020
C	4.36030	5.15510	-8.53230
C	5.05110	5.56650	-9.66400
C	6.43670	5.45160	-9.71630
C	7.13510	4.93230	-8.62970
C	6.44940	4.53220	-7.49270
H	3.28480	5.24750	-8.48830
H	4.50840	5.98110	-10.50370
H	6.97280	5.77170	-10.60090
H	8.21350	4.84720	-8.66730
H	6.98080	4.13910	-6.63700
O	4.92260	3.97220	-5.14920
O	-1.19810	5.84870	-7.65890

O	-2.06700	8.86130	-4.05110
C	6.06550	7.68940	-5.55170
C	6.45650	8.09100	-6.82240
C	5.52180	8.67830	-7.66980
H	3.46970	9.30190	-7.90390
H	4.46830	7.52840	-4.13220
H	6.77910	7.21690	-4.88850
H	7.47250	7.93110	-7.15690
H	5.80860	8.98380	-8.66790

Phenyl β-D-Galf **6**, C3-exo, 180°/+60°

	**X**	**Y**	**Z**
C	4.14180	5.73500	-2.77840
O	5.54870	5.82920	-2.70460
C	3.77730	5.77970	-4.26570
C	5.12940	5.78260	-4.97620
C	6.04800	6.39670	-3.92210
O	3.50100	6.82720	-2.15310
H	3.83980	4.80350	-2.29940
O	5.13790	6.58730	-6.15410
C	3.46240	6.87080	-0.77950
H	3.23440	6.69380	-4.49410
O	2.97660	4.64480	-4.59340
C	2.05890	4.79180	-5.57620
C	4.89360	5.97600	-7.33570
H	5.42940	4.76290	-5.21630
C	7.52090	6.05820	-4.04640
C	8.07300	6.44400	-5.40920
H	7.63880	4.97710	-3.90570
O	8.19440	6.76470	-3.01020
O	9.48080	6.14350	-5.35860
H	7.61760	5.86720	-6.21200
H	7.93690	7.50710	-5.60760
H	9.13900	6.57870	-3.08960

H	5.92800	7.48600	-3.91160
C	10.20510	6.45580	-6.44690
C	1.33380	3.53090	-5.85900
C	0.36190	3.54250	-6.86230
C	1.59850	2.34900	-5.16210
C	0.89290	1.19300	-5.46730
C	-0.07580	1.20920	-6.46680
C	-0.34020	2.38510	-7.16450
H	0.16750	4.46190	-7.39710
H	2.35190	2.33680	-4.38820
H	1.09910	0.27870	-4.92620
H	-0.62380	0.30580	-6.70290
H	-1.09210	2.39780	-7.94280
C	4.99570	6.91080	-8.47920
C	5.44120	8.22590	-8.32240
C	5.53460	9.06030	-9.42780
C	5.18040	8.59050	-10.68930
C	4.73400	7.28090	-10.84790
C	4.64390	6.44190	-9.74720
H	5.71740	8.58820	-7.34300
H	5.88450	10.07720	-9.30590
H	5.25280	9.24450	-11.54910
H	4.45760	6.91600	-11.82860
H	4.30060	5.42220	-9.85560
C	11.64420	6.12620	-6.28960
C	12.15120	5.55930	-5.11740
C	13.50560	5.27040	-5.01700
C	14.35900	5.54470	-6.08200
C	13.85670	6.10940	-7.25150
C	12.50420	6.39960	-7.35580
H	11.48860	5.34590	-4.29120
H	13.89580	4.83130	-4.10800
H	15.41460	5.31830	-6.00080
H	14.51970	6.32270	-8.08010
H	12.10120	6.83860	-8.25810

O	9.71650	6.95110	-7.43940
O	4.63240	4.79780	-7.42450
O	1.86540	5.84120	-6.14660
C	4.21270	6.04110	0.05060
C	4.08620	6.16930	1.43230
C	3.23500	7.11660	1.98700
C	2.49910	7.94830	1.14530
C	2.60840	7.82760	-0.23240
H	4.90220	5.31820	-0.35910
H	4.67060	5.52220	2.07440
H	3.14780	7.21070	3.06160
H	1.83240	8.69250	1.56260
H	2.03730	8.46220	-0.89770

Phenyl β-D-Galf **6**, C3-exo, 180°/-60°

	**X**	**Y**	**Z**
C	2.21790	7.54300	-4.17160
O	2.87030	6.40750	-4.67830
C	1.02140	7.01430	-3.38010
C	0.80450	5.59550	-3.93370
C	1.85870	5.45910	-5.04620
O	1.68280	8.35650	-5.19840
H	2.92930	8.10210	-3.56530
O	-0.50450	5.45250	-4.48270
H	0.13780	7.63120	-3.51370
O	1.40260	6.99420	-1.99930
C	0.40230	6.84980	-1.10280
C	-1.32330	4.50500	-3.97420
H	0.95360	4.86470	-3.14240
C	2.50620	4.09210	-5.18540
C	1.49880	2.96470	-5.33410
H	3.07660	3.89120	-4.27030
O	3.37860	4.17490	-6.30880
O	0.60680	3.29310	-6.41450

H	2.01820	2.03260	-5.56270
H	0.92650	2.82060	-4.41840
H	3.90940	3.36910	-6.34720
H	1.40960	5.74560	-6.00000
C	-0.45510	2.49320	-6.59980
C	0.89100	6.83510	0.29630
C	-0.04780	6.68520	1.31980
C	2.24690	6.96180	0.60980
C	2.65500	6.93930	1.93660
C	1.71630	6.79020	2.95360
C	0.36450	6.66320	2.64390
H	-1.09410	6.58620	1.06520
H	2.97460	7.07590	-0.18040
H	3.70550	7.03760	2.17780
H	2.03790	6.77260	3.98730
H	-0.36540	6.54660	3.43440
C	-2.62000	4.45140	-4.68750
C	-2.94140	5.34790	-5.70980
C	-4.15800	5.24010	-6.36670
C	-5.05580	4.23830	-6.01250
C	-4.73830	3.34200	-4.99590
C	-3.52440	3.44760	-4.33420
H	-2.23730	6.11570	-5.99360
H	-4.40010	5.92850	-7.16540
H	-5.99980	4.15010	-6.53520
H	-5.43410	2.55860	-4.72490
H	-3.26110	2.75300	-3.54880
C	-1.34860	2.98210	-7.67860
C	-1.04010	4.11730	-8.43240
C	-1.91510	4.55250	-9.41740
C	-3.10110	3.86240	-9.65170
C	-3.41150	2.73310	-8.90010
C	-2.53770	2.29250	-7.91750
H	-0.12340	4.65590	-8.24200
H	-1.67530	5.43320	-9.99910

H	-3.78570	4.20810	-10.41600
H	-4.33810	2.20210	-9.07490
H	-2.77200	1.42330	-7.31910
O	-0.65310	1.49700	-5.93660
O	-1.00650	3.77260	-3.06370
O	-0.75780	6.74430	-1.43300
C	2.54080	9.06810	-6.00180
C	1.92950	9.91880	-6.92230
C	2.70760	10.69220	-7.77130
C	4.09810	10.62520	-7.71050
C	4.69750	9.77120	-6.79360
C	3.92980	8.98400	-5.93770
H	0.84860	9.96500	-6.95540
H	2.22480	11.35180	-8.48140
H	4.70420	11.22880	-8.37330
H	5.77690	9.70330	-6.74040
H	4.41800	8.30910	-5.25130

Phenyl β-D-Galf **6**, C3-exo, 180°/180°

	**X**	**Y**	**Z**
C	5.85560	3.96790	-4.32550
O	5.02230	4.55470	-5.29300
C	5.77990	2.46160	-4.59220
C	4.45000	2.28740	-5.34020
C	3.87350	3.70980	-5.43760
O	5.37430	4.16480	-3.01170
H	6.85350	4.38800	-4.44110
O	3.50200	1.46380	-4.65720
C	5.44830	5.41840	-2.45380
C	5.05950	5.50230	-1.11730
C	5.87740	6.55600	-3.13340
H	5.82490	1.88700	-3.67280
O	6.83000	2.06350	-5.48030
C	8.03250	1.80920	-4.92600

C	3.74530	0.13500	-4.66180
H	4.64790	1.86340	-6.32190
C	3.17040	4.05650	-6.73830
C	1.88720	3.25840	-6.94830
H	3.86010	3.88670	-7.57250
O	2.81420	5.43300	-6.64710
O	2.21700	1.89810	-7.27500
H	1.26790	3.28290	-6.05220
H	1.32310	3.69490	-7.77440
H	2.50850	5.72990	-7.51340
H	3.18350	3.87690	-4.60430
C	1.22160	0.99900	-7.17770
C	9.03020	1.35580	-5.92470
C	10.33170	1.09920	-5.48770
C	8.70200	1.18070	-7.27170
C	9.67050	0.75290	-8.16960
C	10.96680	0.49920	-7.73040
C	11.29650	0.67270	-6.38860
H	10.57410	1.23790	-4.44300
H	7.69530	1.37700	-7.61070
H	9.41430	0.61660	-9.21230
H	11.72000	0.16610	-8.43320
H	12.30430	0.47560	-6.04680
C	2.59930	-0.65470	-4.15790
C	1.38960	-0.05530	-3.79900
C	0.32340	-0.84240	-3.38850
C	0.45940	-2.22660	-3.33110
C	1.66550	-2.82570	-3.68410
C	2.73230	-2.04310	-4.09790
H	1.28150	1.01770	-3.85790
H	-0.61600	-0.37730	-3.11940
H	-0.37590	-2.83860	-3.01510
H	1.76920	-3.90220	-3.64550
H	3.66930	-2.49570	-4.39060
C	1.67650	-0.38950	-7.43050

C	2.97480	-0.67900	-7.85440
C	3.36450	-1.99840	-8.03850
C	2.46470	-3.03170	-7.79760
C	1.16800	-2.74540	-7.37800
C	0.77350	-1.42940	-7.19860
H	3.67770	0.12280	-8.02650
H	4.37350	-2.22060	-8.36080
H	2.77470	-4.06040	-7.93190
H	0.47080	-3.54930	-7.18140
H	-0.22520	-1.19310	-6.85930
O	0.08400	1.30670	-6.89250
O	4.78940	-0.33230	-5.05840
O	8.24970	1.94470	-3.74180
C	5.92560	7.77310	-2.45670
C	5.54410	7.86640	-1.12440
C	5.10770	6.72230	-0.45910
H	4.72810	4.60500	-0.61070
H	6.15390	6.51310	-4.17590
H	6.25990	8.65510	-2.98860
H	5.58160	8.81770	-0.60990
H	4.80580	6.77820	0.57930

Phenyl β-D-Galf **6**, O4-exo, +60°/+60°

	**X**	**Y**	**Z**
C	-0.08630	2.05700	-6.46710
O	0.30900	1.12970	-7.44460
C	-1.60360	2.15920	-6.61850
C	-1.80930	1.97760	-8.12620
C	-0.52460	1.27620	-8.60720
O	0.41770	3.35260	-6.71300
H	0.24250	1.68640	-5.49730
O	-1.88600	3.24530	-8.78970
C	1.75900	3.59710	-6.54470
O	-2.23050	1.04460	-5.97770

C	-2.45330	1.15420	-4.64690
C	-3.08050	3.87360	-8.77670
C	-0.69810	-0.09860	-9.25890
C	-1.41830	-1.09590	-8.37360
H	0.30400	-0.48180	-9.46150
O	-1.31770	0.04430	-10.53070
O	-1.27330	-2.38860	-8.98950
C	-1.92170	-3.40800	-8.40780
H	-0.03280	1.92210	-9.33660
C	-3.03310	5.18420	-9.46760
C	-1.72410	-4.69440	-9.12380
C	-0.98010	-4.77730	-10.30370
C	-0.82630	-6.00110	-10.94120
C	-1.41030	-7.14580	-10.40630
C	-2.15220	-7.06650	-9.23060
C	-2.30960	-5.84520	-8.59150
H	-0.52920	-3.88770	-10.71900
H	-0.25070	-6.06230	-11.85590
H	-1.28810	-8.09890	-10.90540
H	-2.60690	-7.95600	-8.81400
H	-2.88420	-5.77040	-7.67850
C	-3.09360	-0.05360	-4.07740
C	-3.33020	-1.19870	-4.84210
C	-3.92340	-2.30930	-4.25730
C	-4.28670	-2.28190	-2.91420
C	-4.05210	-1.14130	-2.14970
C	-3.45510	-0.03100	-2.72760
H	-3.04580	-1.23290	-5.88210
H	-4.09780	-3.19550	-4.85290
H	-4.75080	-3.14930	-2.46160
H	-4.33360	-1.11990	-1.10480
H	-3.26440	0.85960	-2.14480
C	-4.21760	5.91640	-9.57740
C	-4.21610	7.15010	-10.21150
C	-3.03170	7.66100	-10.73630

C	-1.84870	6.93570	-10.62640
C	-1.84570	5.69940	-9.99480
H	-5.12990	5.50940	-9.16350
H	-5.13590	7.71410	-10.29640
H	-3.03070	8.62470	-11.22980
H	-0.92800	7.33400	-11.03250
H	-0.92780	5.13670	-9.90640
H	-2.00290	3.09480	-6.24120
H	-2.72040	1.41850	-8.31980
H	-2.26090	0.21770	-10.41040
O	-4.06460	3.40050	-8.25180
O	-2.15110	2.14310	-4.01760
O	-2.59360	-3.27640	-7.40560
H	-2.48040	-0.86220	-8.28510
H	-0.97980	-1.11640	-7.37700
C	2.69840	2.62540	-6.20850
C	4.03200	2.99700	-6.05000
C	4.43360	4.31430	-6.23130
C	3.48470	5.27480	-6.57610
C	2.15250	4.92150	-6.73280
H	2.41360	1.59150	-6.08480
H	4.75940	2.23890	-5.78800
H	5.47260	4.59100	-6.10930
H	3.78200	6.30570	-6.72190
H	1.40410	5.65770	-6.99650

Phenyl β-D-Galf **6**, O4-exo, +60°/-60°

	**X**	**Y**	**Z**
C	-0.44890	1.99890	-3.12230
O	-1.33240	2.00760	-4.19510
C	-1.15710	1.20610	-2.02630
C	-2.62980	1.55500	-2.25150
C	-2.67960	2.11190	-3.69510
O	-0.28650	3.35220	-2.70420

H	0.49450	1.56730	-3.45320
O	-2.98720	2.55750	-1.28950
C	0.60870	3.65350	-1.71060
O	-0.91940	-0.17740	-2.29800
C	-1.21690	-1.04470	-1.30210
C	-4.29110	2.70400	-1.01410
C	-3.61440	1.42760	-4.69570
C	-3.38230	-0.05700	-4.92630
H	-3.40690	1.89940	-5.66050
O	-4.96780	1.71800	-4.39200
O	-3.78250	-0.79640	-3.75610
C	-3.85130	-2.13250	-3.87590
H	-2.96810	3.16450	-3.64510
C	-4.54320	3.72940	0.02130
C	-4.27130	-2.79760	-2.61770
C	-4.65960	-2.07300	-1.48830
C	-5.01570	-2.74090	-0.32540
C	-4.99000	-4.13190	-0.28350
C	-4.61130	-4.85650	-1.40980
C	-4.25350	-4.19240	-2.57300
H	-4.68400	-0.99400	-1.51790
H	-5.31100	-2.17590	0.54920
H	-5.26340	-4.65050	0.62680
H	-4.58470	-5.93790	-1.37650
H	-3.94310	-4.74290	-3.44980
C	-1.00050	-2.45420	-1.69440
C	-0.62750	-2.81080	-2.99240
C	-0.47190	-4.14950	-3.32230
C	-0.68060	-5.13370	-2.36110
C	-1.04890	-4.77970	-1.06590
C	-1.21160	-3.44470	-0.73290
H	-0.47720	-2.04590	-3.73990
H	-0.19490	-4.42560	-4.33130
H	-0.56350	-6.17760	-2.62310
H	-1.22190	-5.54600	-0.32180

H	-1.51480	-3.15660	0.26360
C	-5.86440	3.98140	0.39880
C	-6.13910	4.93690	1.36590
C	-5.09740	5.64560	1.95900
C	-3.77960	5.39790	1.58390
C	-3.49870	4.44240	0.61750
H	-6.66380	3.42490	-0.07070
H	-7.16320	5.13030	1.65760
H	-5.31270	6.39160	2.71360
H	-2.97080	5.95000	2.04470
H	-2.47680	4.25000	0.32430
H	-0.83300	1.45660	-1.02130
H	-3.26250	0.68840	-2.11330
H	-5.11450	1.62400	-3.43440
O	-5.15860	2.05310	-1.56820
O	-1.62330	-0.67730	-0.22250
O	-3.58550	-2.71510	-4.90560
H	-2.33120	-0.25730	-5.13650
H	-3.98210	-0.38600	-5.77340
C	1.59950	2.78360	-1.25910
C	2.46610	3.20260	-0.25150
C	2.35370	4.47000	0.30470
C	1.35820	5.33060	-0.15480
C	0.48650	4.92720	-1.15490
H	1.70810	1.79130	-1.67240
H	3.23290	2.52220	0.09680
H	3.03000	4.78510	1.08850
H	1.25590	6.32060	0.27170
H	-0.29570	5.58240	-1.51580

Phenyl β-D-Galf **6**, O4-exo, +60°/180°

	**X**	**Y**	**Z**
C	-3.49200	2.46170	-8.15590
O	-4.16080	2.17810	-6.95960

C	-2.04100	2.03850	-7.92560
C	-1.84350	2.27780	-6.42450
C	-3.26850	2.34670	-5.84670
O	-3.44910	3.84870	-8.43540
H	-3.98910	1.92550	-8.96080
O	-1.21530	3.54380	-6.18210
C	-4.60040	4.47920	-8.83800
C	-4.45740	5.82100	-9.18980
C	-5.84800	3.86320	-8.91040
O	-1.86210	0.63660	-8.14800
C	-1.80540	0.22500	-9.43410
C	0.12860	3.59280	-6.29900
C	-3.62510	1.33270	-4.75240
C	-3.29990	-0.13080	-5.02550
H	-4.69970	1.42380	-4.58420
O	-3.00630	1.71570	-3.52860
O	-4.01450	-0.55110	-6.19340
C	-3.80770	-1.80510	-6.61910
H	-3.41210	3.33830	-5.41190
C	0.66650	4.94990	-6.04200
C	-4.43940	-2.05770	-7.93760
C	-5.10800	-1.04670	-8.63250
C	-5.62860	-1.30080	-9.89340
C	-5.48910	-2.56310	-10.46360
C	-4.83040	-3.57380	-9.76920
C	-4.30650	-3.32290	-8.51020
H	-5.20560	-0.06750	-8.18760
H	-6.13770	-0.51320	-10.43390
H	-5.88910	-2.75760	-11.45080
H	-4.71410	-4.55250	-10.21610
H	-3.77450	-4.09270	-7.96940
C	-1.48510	-1.21500	-9.55200
C	-0.95890	-1.94110	-8.48180
C	-0.65530	-3.28520	-8.64200
C	-0.88340	-3.91100	-9.86360

C	-1.41200	-3.18980	-10.93070
C	-1.70720	-1.84400	-10.77850
H	-0.79080	-1.45530	-7.53220
H	-0.24870	-3.84670	-7.81110
H	-0.65390	-4.96230	-9.98310
H	-1.59880	-3.67920	-11.87770
H	-2.12160	-1.27330	-11.59800
C	2.05210	5.12630	-6.06260
C	2.59680	6.38110	-5.83250
C	1.76120	7.46710	-5.58420
C	0.37990	7.29590	-5.56550
C	-0.16970	6.04150	-5.79240
H	2.68970	4.27520	-6.25830
H	3.67080	6.51390	-5.84730
H	2.18660	8.44670	-5.40620
H	-0.26920	8.14070	-5.37460
H	-1.24160	5.90760	-5.78090
H	-1.34860	2.60650	-8.54000
H	-1.23100	1.48920	-5.99530
H	-2.05200	1.57230	-3.58860
O	0.79740	2.62390	-6.58510
O	-1.99740	0.97360	-10.36660
O	-3.15570	-2.61610	-5.99560
H	-3.60480	-0.72650	-4.16510
H	-2.23000	-0.28320	-5.18460
C	-6.94470	4.60060	-9.35200
C	-6.81160	5.93650	-9.70860
C	-5.56000	6.54310	-9.62260
H	-3.47850	6.27810	-9.12330
H	-5.98110	2.83260	-8.61790
H	-7.91230	4.11780	-9.40760
H	-7.67090	6.50070	-10.04650
H	-5.43950	7.58380	-9.89620

Phenyl β-D-Galf **6**, O4-exo, -60°/+60°

	X	Y	Z
C	0.18930	2.38610	-5.62270
O	1.00910	1.29880	-5.27480
C	-1.09480	1.74830	-6.15180
C	-0.59430	0.46080	-6.81610
C	0.81090	0.23860	-6.23010
O	0.71960	3.14030	-6.69000
H	0.05150	3.00180	-4.73520
O	-0.45240	0.62060	-8.23280
C	1.83250	3.91830	-6.47670
O	-1.92310	1.35140	-5.05580
C	-2.71790	2.29810	-4.51090
C	-1.57170	0.50400	-8.97810
C	0.98110	-1.08890	-5.50310
C	2.40360	-1.23950	-4.99410
H	0.77870	-1.88830	-6.21960
O	0.02320	-1.24190	-4.46490
O	2.53600	-2.58100	-4.49150
C	3.73290	-2.92550	-3.99520
H	1.54850	0.32030	-7.03130
C	-1.30600	0.69150	-10.42470
C	3.75920	-4.32120	-3.48550
C	2.61660	-5.12540	-3.47220
C	2.68790	-6.42290	-2.98280
C	3.89600	-6.92470	-2.50700
C	5.03640	-6.12570	-2.51870
C	4.96850	-4.82800	-3.00470
H	1.67900	-4.73400	-3.83950
H	1.80090	-7.04300	-2.97210
H	3.94870	-7.93710	-2.12670
H	5.97620	-6.51480	-2.14890
H	5.84660	-4.19710	-3.01800
C	-3.52210	1.77210	-3.38290
C	-3.40050	0.45150	-2.94150

C	-4.17180	0.00530	-1.87700
C	-5.06550	0.86980	-1.25100
C	-5.18820	2.18600	-1.68900
C	-4.41840	2.63720	-2.75100
H	-2.70610	-0.21810	-3.42800
H	-4.07610	-1.01730	-1.53550
H	-5.66600	0.51820	-0.42160
H	-5.88290	2.85810	-1.20220
H	-4.50280	3.65710	-3.10050
C	-2.36660	0.52470	-11.31820
C	-2.16230	0.69400	-12.67970
C	-0.89900	1.03490	-13.15640
C	0.15980	1.20520	-12.26890
C	-0.03920	1.03340	-10.90570
H	-3.34300	0.26200	-10.93500
H	-2.98580	0.56180	-13.36940
H	-0.74020	1.16850	-14.21900
H	1.14110	1.47230	-12.63940
H	0.78120	1.16740	-10.21580
H	-1.63850	2.39940	-6.82860
H	-1.27180	-0.36100	-6.60430
H	0.08280	-0.46900	-3.88540
O	-2.65920	0.27440	-8.49660
O	-2.74870	3.43860	-4.91610
O	4.68190	-2.16950	-3.97310
H	2.61330	-0.53040	-4.19270
H	3.12100	-1.08230	-5.80080
C	2.53570	3.97030	-5.27570
C	3.64260	4.81060	-5.17390
C	4.05470	5.58400	-6.25150
C	3.34700	5.51570	-7.44990
C	2.24020	4.68760	-7.56560
H	2.24800	3.36210	-4.43150
H	4.18690	4.84880	-4.23860
H	4.91820	6.23020	-6.16230

H	3.65620	6.11170	-8.29940
H	1.67830	4.62790	-8.48880

Phenyl β-D-Galf **6**, O4-exo, -60°/-60°

	**X**	**Y**	**Z**
C	-1.19730	0.00850	-3.06910
O	-2.26210	0.10540	-3.98170
C	-1.07400	1.40470	-2.46270
C	-1.49640	2.32360	-3.61310
C	-2.23120	1.40420	-4.60580
O	0.02850	-0.25990	-3.70950
H	-1.44370	-0.76660	-2.34590
O	-0.36200	2.87600	-4.29220
C	0.25880	-1.52260	-4.20430
O	-2.04940	1.56830	-1.42930
C	-1.73720	1.08940	-0.20600
C	0.23490	3.94180	-3.71810
C	-3.66750	1.83490	-4.89570
C	-4.30230	1.12500	-6.08310
H	-3.63700	2.89440	-5.16570
O	-4.46790	1.74020	-3.72590
O	-4.39740	-0.29750	-5.87380
C	-3.48880	-1.08870	-6.47050
H	-1.66310	1.35440	-5.53600
C	1.39660	4.42690	-4.50130
C	-3.66410	-2.51730	-6.10980
C	-4.39690	-2.90670	-4.98710
C	-4.50470	-4.25280	-4.66380
C	-3.89980	-5.21460	-5.46830
C	-3.17530	-4.82840	-6.59240
C	-3.04800	-3.48350	-6.90620
H	-4.86450	-2.15720	-4.36500
H	-5.06160	-4.55250	-3.78540
H	-3.98920	-6.26380	-5.21640

H	-2.70040	-5.57500	-7.21560
H	-2.47180	-3.16970	-7.76560
C	-2.82340	1.31480	0.77680
C	-4.03470	1.91080	0.41470
C	-5.02510	2.09790	1.36930
C	-4.81280	1.69490	2.68470
C	-3.60680	1.10040	3.04770
C	-2.61500	0.90920	2.09710
H	-4.19830	2.22190	-0.60690
H	-5.96300	2.55870	1.08740
H	-5.58700	1.84340	3.42680
H	-3.44180	0.78690	4.07030
H	-1.67450	0.44820	2.36570
C	2.08500	5.54900	-4.03420
C	3.18050	6.03380	-4.73340
C	3.59620	5.39970	-5.90160
C	2.91390	4.28020	-6.36940
C	1.81580	3.79250	-5.67380
H	1.75270	6.03100	-3.12500
H	3.71110	6.90390	-4.36910
H	4.45210	5.77770	-6.44640
H	3.23810	3.78680	-7.27650
H	1.28700	2.92240	-6.03480
H	-0.07720	1.61110	-2.08630
H	-2.12590	3.12640	-3.24010
H	-4.30940	0.86910	-3.33530
O	-0.15090	4.42720	-2.67740
O	-0.68380	0.54210	0.03320
O	-2.63460	-0.67900	-7.22740
H	-3.73890	1.32700	-6.99210
H	-5.32760	1.47110	-6.19720
C	1.50550	-1.71360	-4.79830
C	1.84330	-2.95940	-5.30630
C	0.94490	-4.02170	-5.22440
C	-0.29630	-3.81800	-4.63630

C	-0.65360	-2.57090	-4.13010
H	2.19560	-0.88120	-4.84820
H	2.81380	-3.10100	-5.76510
H	1.20930	-4.99340	-5.62080
H	-1.01150	-4.62740	-4.57980
H	-1.63680	-2.43340	-3.70790

Phenyl β-D-Galf **6**, O4-exo, -60°/180°

	**X**	**Y**	**Z**
C	-2.08840	4.43490	-6.60640
O	-2.55870	3.12470	-6.44800
C	-1.84880	4.92640	-5.17830
C	-2.95480	4.21540	-4.38420
C	-3.47090	3.12350	-5.33550
O	-3.08060	5.29340	-7.13750
H	-1.19960	4.40520	-7.23460
O	-4.05540	5.08610	-4.09890
C	-3.69030	4.95900	-8.32600
C	-4.88910	5.61730	-8.59010
C	-3.17550	4.04120	-9.23710
O	-0.60240	4.41790	-4.69440
C	0.51290	5.09880	-5.03540
C	-3.91740	5.93840	-3.06320
C	-3.51230	1.71750	-4.76410
C	-3.95350	0.70340	-5.81990
H	-4.21760	1.71810	-3.93070
O	-2.26060	1.31470	-4.22630
O	-5.17750	1.17400	-6.41170
C	-5.31740	1.09480	-7.74780
H	-4.46740	3.40390	-5.67790
C	-5.11550	6.79270	-2.87820
C	-6.52000	1.81980	-8.22880
C	-7.23230	2.69450	-7.40480
C	-8.33320	3.37600	-7.90510

C	-8.73460	3.18120	-9.22340
C	-8.02850	2.30830	-10.04600
C	-6.92060	1.63540	-9.55320
H	-6.91680	2.84780	-6.38310
H	-8.87640	4.06150	-7.26760
H	-9.59440	3.71320	-9.61090
H	-8.33630	2.16070	-11.07300
H	-6.35480	0.96600	-10.18610
C	1.74030	4.47790	-4.48360
C	1.69980	3.29750	-3.73650
C	2.87670	2.75130	-3.24310
C	4.09500	3.37790	-3.49010
C	4.13800	4.55400	-4.23450
C	2.96480	5.10270	-4.73110
H	0.75350	2.81210	-3.54620
H	2.84410	1.83670	-2.66530
H	5.01100	2.94960	-3.10300
H	5.08510	5.04110	-4.42680
H	2.98400	6.01500	-5.31120
C	-5.13290	7.68300	-1.80210
C	-6.23210	8.50390	-1.59650
C	-7.31840	8.44260	-2.46570
C	-7.30360	7.55860	-3.54100
C	-6.20700	6.73330	-3.74920
H	-4.28230	7.72160	-1.13570
H	-6.24300	9.19160	-0.76090
H	-8.17550	9.08450	-2.30560
H	-8.14700	7.51300	-4.21780
H	-6.19310	6.04850	-4.58460
H	-1.88350	6.00790	-5.09290
H	-2.55250	3.81370	-3.45860
H	-1.58740	1.43060	-4.91190
O	-2.91780	5.98690	-2.38060
O	0.48520	6.09980	-5.71620
O	-4.54430	0.49870	-8.46590

H	-4.13760	-0.26680	-5.35990
H	-3.19110	0.59960	-6.58870
C	-3.87130	3.79430	-10.41730
C	-5.06600	4.44750	-10.69130
C	-5.57080	5.36170	-9.77120
H	-5.27530	6.31580	-7.85900
H	-2.25810	3.50890	-9.03560
H	-3.47430	3.07170	-11.11910
H	-5.60750	4.23540	-11.60340
H	-6.50790	5.86770	-9.96480

Phenyl β-D-Galf **6**, O4-exo, 180°/+60°

	**X**	**Y**	**Z**
C	-0.78840	2.87200	-7.49530
O	-2.12850	2.74740	-7.09110
C	-0.12720	1.55180	-7.08960
C	-0.98070	1.07070	-5.90820
C	-2.13780	2.08080	-5.82340
O	-0.10490	3.88610	-6.78770
H	-0.77620	3.06560	-8.56650
O	-0.28290	1.09910	-4.66050
C	-0.41590	5.20160	-7.03580
O	-0.26800	0.58490	-8.13530
C	0.63580	0.63520	-9.13760
C	0.57380	0.08290	-4.41650
C	-3.51470	1.46940	-5.59900
C	-3.54840	0.70550	-4.28600
H	-3.72770	0.78200	-6.42100
O	-4.53310	2.45840	-5.65800
O	-4.81520	0.03050	-4.22130
C	-5.02980	-0.74330	-3.14620
H	-1.92090	2.80110	-5.02720
C	1.19580	0.17830	-3.07530
C	-6.36580	-1.39250	-3.16800

C	-7.29000	-1.14930	-4.18740
C	-8.52840	-1.77690	-4.16390
C	-8.85040	-2.64870	-3.12770
C	-7.93130	-2.89330	-2.11050
C	-6.69360	-2.26670	-2.12930
H	-7.04000	-0.47070	-4.98990
H	-9.24300	-1.58570	-4.95410
H	-9.81660	-3.13710	-3.11250
H	-8.18070	-3.57120	-1.30440
H	-5.97170	-2.44780	-1.34490
C	0.42130	-0.43120	-10.14440
C	-0.58910	-1.38680	-10.00630
C	-0.75070	-2.36750	-10.97530
C	0.09060	-2.39960	-12.08380
C	1.09800	-1.44840	-12.22410
C	1.26420	-0.46740	-11.25780
H	-1.24120	-1.36110	-9.14550
H	-1.53300	-3.10750	-10.86590
H	-0.03840	-3.16560	-12.83790
H	1.75230	-1.47320	-13.08590
H	2.04280	0.27660	-11.35490
C	2.13030	-0.79400	-2.71080
C	2.73470	-0.74420	-1.46340
C	2.40880	0.27570	-0.57290
C	1.47780	1.24620	-0.93220
C	0.87100	1.20100	-2.18000
H	2.37410	-1.58060	-3.41160
H	3.45870	-1.49860	-1.18420
H	2.88040	0.31390	0.40090
H	1.22430	2.03810	-0.23940
H	0.14780	1.95310	-2.45970
H	0.91910	1.68180	-6.83090
H	-1.32850	0.05890	-6.10830
H	-4.39230	3.09640	-4.94450
O	0.79010	-0.79380	-5.22330

O	1.51260	1.46930	-9.18450
O	-4.21030	-0.88700	-2.26310
H	-3.45600	1.38690	-3.43740
H	-2.74830	-0.03150	-4.22450
C	-1.42860	5.61980	-7.89580
C	-1.63990	6.98440	-8.08210
C	-0.86440	7.92560	-7.41700
C	0.13900	7.49330	-6.55220
C	0.36530	6.13820	-6.36020
H	-2.05950	4.90720	-8.40480
H	-2.42760	7.30570	-8.75210
H	-1.04000	8.98290	-7.56620
H	0.75190	8.21420	-6.02570
H	1.14500	5.78860	-5.69580

Phenyl β-D-Galf **6**, O4-exo, 180°/-60°

	**X**	**Y**	**Z**
C	-2.07920	5.11100	-6.23160
O	-2.92360	4.03740	-6.49450
C	-1.02250	4.59800	-5.24030
C	-1.62560	3.29670	-4.69360
C	-3.03840	3.25230	-5.29750
O	-2.87090	6.13310	-5.63260
H	-1.65630	5.45910	-7.17370
O	-1.71400	3.32760	-3.26980
C	-2.33800	7.38570	-5.48570
O	0.18620	4.37500	-5.97500
C	1.32080	4.25960	-5.24950
C	-1.06080	2.38680	-2.55250
C	-3.56940	1.87440	-5.67840
C	-3.48330	0.86580	-4.54800
H	-2.96110	1.49100	-6.50100
O	-4.89350	1.97650	-6.19030
O	-4.17950	1.42480	-3.41590

C	-4.08710	0.76660	-2.24800
H	-3.73350	3.73340	-4.60320
C	-1.30310	2.53300	-1.09900
C	-4.74040	1.49570	-1.13370
C	-5.38240	2.72110	-1.32990
C	-5.95510	3.38260	-0.25320
C	-5.88770	2.82870	1.02200
C	-5.24680	1.60950	1.22030
C	-4.67540	0.94390	0.14620
H	-5.42340	3.15480	-2.31840
H	-6.44910	4.33340	-0.40680
H	-6.32950	3.35030	1.86180
H	-5.18540	1.18320	2.21310
H	-4.16390	0.00240	0.28870
C	2.52090	4.07600	-6.09880
C	2.44480	4.05080	-7.49390
C	3.59860	3.87820	-8.24630
C	4.82950	3.73010	-7.61340
C	4.90810	3.75430	-6.22320
C	3.75800	3.92660	-5.46730
H	1.48900	4.16600	-7.98390
H	3.53810	3.85920	-9.32670
H	5.72750	3.59540	-8.20320
H	5.86500	3.63860	-5.73100
H	3.80470	3.94670	-4.38720
C	-0.79500	1.55180	-0.24500
C	-1.02510	1.63250	1.12000
C	-1.76080	2.69430	1.63910
C	-2.26660	3.67470	0.79190
C	-2.04280	3.59600	-0.57450
H	-0.23260	0.72870	-0.66330
H	-0.63700	0.86660	1.77880
H	-1.94730	2.75260	2.70400
H	-2.84830	4.49300	1.19470
H	-2.44730	4.34820	-1.23500

H	-0.83670	5.30410	-4.43560
H	-1.02200	2.44870	-5.00930
H	-5.47460	2.28030	-5.47980
O	-0.38960	1.51470	-3.05690
O	1.32660	4.30670	-4.03990
O	-3.51050	-0.29400	-2.13700
H	-2.45150	0.65210	-4.27320
H	-3.96560	-0.06390	-4.84910
C	-1.04950	7.74360	-5.88170
C	-0.61610	9.05420	-5.68660
C	-1.44520	10.00130	-5.10190
C	-2.72990	9.63070	-4.70650
C	-3.17680	8.33220	-4.89460
H	-0.37620	7.03000	-6.33320
H	0.38550	9.32520	-5.99620
H	-1.09810	11.01520	-4.95260
H	-3.38870	10.35770	-4.24830
H	-4.17210	8.03250	-4.59270

Phenyl β-D-Galf **6**, O4-exo, 180°/180°

	**X**	**Y**	**Z**
C	-2.28710	1.10110	-8.78490
O	-2.95940	1.78490	-7.75800
C	-2.60870	-0.37760	-8.55620
C	-2.94200	-0.45230	-7.05870
C	-2.83350	1.00150	-6.56450
O	-0.88290	1.22100	-8.67940
H	-2.64180	1.49000	-9.73800
O	-2.03010	-1.25250	-6.30270
C	-0.29120	2.42940	-8.95800
C	1.10290	2.43340	-8.92780
C	-0.99100	3.59560	-9.25900
O	-3.79120	-0.74300	-9.27570
C	-3.63590	-1.08050	-10.57220

C	-2.12590	-2.59020	-6.46420
C	-3.89360	1.45790	-5.56890
C	-3.76780	0.77210	-4.21250
H	-4.88360	1.26980	-5.98980
O	-3.82120	2.86350	-5.36260
O	-4.14930	-0.60790	-4.33950
C	-3.74640	-1.44300	-3.36560
H	-1.84000	1.15370	-6.12680
C	-1.31090	-3.34100	-5.48280
C	-4.12920	-2.85150	-3.62740
C	-4.94210	-3.20990	-4.70440
C	-5.24350	-4.54600	-4.92950
C	-4.73390	-5.52750	-4.08570
C	-3.92580	-5.17230	-3.00850
C	-3.62710	-3.83930	-2.77730
H	-5.32670	-2.44780	-5.36600
H	-5.86870	-4.82190	-5.76880
H	-4.96210	-6.56980	-4.26960
H	-3.52150	-5.93640	-2.35770
H	-2.98930	-3.55090	-1.95380
C	-4.91250	-1.49010	-11.20540
C	-6.11030	-1.54420	-10.48770
C	-7.28060	-1.93600	-11.12340
C	-7.26250	-2.27390	-12.47370
C	-6.07020	-2.22100	-13.19120
C	-4.89840	-1.83050	-12.56000
H	-6.12250	-1.28280	-9.43960
H	-8.20720	-1.97820	-10.56570
H	-8.17700	-2.57870	-12.96670
H	-6.05580	-2.48350	-14.24110
H	-3.96600	-1.78500	-13.10570
C	-1.31860	-4.73530	-5.55030
C	-0.61210	-5.47960	-4.61810
C	0.10300	-4.83600	-3.61220
C	0.11450	-3.44570	-3.54280

C	-0.58860	-2.69690	-4.47530
H	-1.89130	-5.22240	-6.32680
H	-0.62630	-6.56050	-4.66840
H	0.64850	-5.41770	-2.87980
H	0.66620	-2.94560	-2.75740
H	-0.59280	-1.61860	-4.41440
H	-1.78100	-1.01940	-8.83900
H	-3.94790	-0.84750	-6.93770
H	-2.92900	3.09540	-5.07060
O	-2.82410	-3.09270	-7.31620
O	-2.56320	-1.04430	-11.13420
O	-3.12280	-1.07040	-2.39460
H	-4.43410	1.26390	-3.50390
H	-2.74540	0.83700	-3.83800
C	-0.27960	4.75940	-9.54380
C	1.10920	4.77250	-9.52110
C	1.79670	3.60150	-9.20830
H	1.62440	1.51540	-8.68930
H	-2.07020	3.61550	-9.25830
H	-0.82710	5.66370	-9.77840
H	1.65130	5.68310	-9.74030
H	2.87920	3.59520	-9.18540

Phenyl β-D-Galf **6**, C2-endo, +60°/+60°

	**X**	**Y**	**Z**
C	3.69870	3.16770	-0.88310
H	3.24490	4.12190	-1.14710
C	2.88710	2.47240	0.20250
H	3.52680	1.80320	0.77190
C	1.80760	1.72900	-0.59690
C	1.46870	0.32770	-0.09270
H	0.66610	-0.06480	-0.72810
O	1.00670	0.49010	1.24280
C	3.59130	2.20440	-2.05220

H	4.34880	1.41510	-1.99060
H	0.89110	2.32220	-0.58720
O	3.72950	2.89860	-3.25320
O	5.07130	3.34550	-0.54630
O	2.26010	3.39120	1.09820
O	2.28720	1.64880	-1.94840
C	2.63730	-0.63650	-0.16960
H	3.45490	-0.34260	0.48830
H	3.00700	-0.72510	-1.19040
O	2.12200	-1.90960	0.26800
H	0.77980	-0.38090	1.59270
C	5.43190	4.51730	0.02410
C	2.89900	3.67320	2.25330
C	2.99510	-2.92880	0.33050
C	2.37190	-4.18670	0.81450
C	1.02090	-4.25770	1.16410
C	0.48340	-5.45660	1.61310
C	1.28790	-6.58790	1.71520
C	2.63460	-6.52040	1.36750
C	3.17540	-5.32430	0.91870
O	4.16130	-2.81350	0.01970
C	6.87520	4.55760	0.35560
C	7.37850	5.70910	0.96570
C	8.72270	5.78940	1.29790
C	9.57200	4.72050	1.02310
C	9.07420	3.57080	0.41640
C	7.72940	3.48590	0.08260
O	4.64930	5.41640	0.23260
C	2.14820	4.64100	3.08780
O	3.96430	3.18360	2.55710
C	2.71510	5.04450	4.29900
C	2.04960	5.95100	5.11110
C	0.81400	6.45960	4.71870
C	0.24600	6.06050	3.51220
C	0.90910	5.15410	2.69590

H	0.39660	-3.37960	1.08440
H	-0.56340	-5.50880	1.88350
H	0.86590	-7.52150	2.06550
H	3.26090	-7.39960	1.44680
H	4.21970	-5.25830	0.64570
H	6.70800	6.53110	1.17490
H	9.10910	6.68270	1.77110
H	10.62130	4.78320	1.28280
H	9.73430	2.73980	0.20410
H	7.34160	2.59360	-0.38660
H	3.67550	4.64290	4.59110
H	2.49230	6.26210	6.04840
H	0.29460	7.16720	5.35250
H	-0.71410	6.45620	3.20700
H	0.46990	4.84470	1.75880
C	4.01570	2.18320	-4.39560
C	4.48060	2.92820	-5.47650
C	3.84550	0.80590	-4.50570
C	4.16370	0.17800	-5.70760
C	4.63160	0.91010	-6.79190
C	4.78410	2.28960	-6.67080
H	4.60260	3.99780	-5.36480
H	3.45430	0.22670	-3.68170
H	4.03240	-0.89350	-5.79140
H	4.87140	0.41380	-7.72320
H	5.14660	2.87260	-7.50800

Phenyl β-D-Galf **6**, C2-endo, +60°/-60°

	**X**	**Y**	**Z**
C	3.67400	2.89800	-0.60580
H	3.36990	3.92330	-0.81250
C	2.76310	2.28320	0.45580
H	3.33130	1.68210	1.15040
C	1.75510	1.44280	-0.35010

C	1.66060	-0.03350	0.05610
H	0.86740	-0.47430	-0.56000
O	1.28360	-0.02340	1.42720
C	3.45430	2.03420	-1.83940
H	4.16690	1.20350	-1.87090
H	0.76130	1.87570	-0.23520
O	3.59330	2.81130	-2.98940
O	5.06610	2.84890	-0.29330
O	2.06600	3.29800	1.18700
O	2.12400	1.55600	-1.73010
C	2.89260	-0.88080	-0.21180
H	3.15130	-0.85670	-1.26980
H	2.69300	-1.91750	0.06650
O	3.99340	-0.39260	0.57550
H	1.08770	-0.92600	1.70810
C	5.56280	3.80580	0.51690
C	2.39140	3.47320	2.48430
C	5.20470	-0.92950	0.35180
C	6.24690	-0.37250	1.24980
C	5.94770	0.56130	2.24490
C	6.95830	1.05620	3.05720
C	8.26780	0.62070	2.88700
C	8.56890	-0.31410	1.90050
C	7.56380	-0.80820	1.08350
O	5.40200	-1.77370	-0.49660
C	7.02630	3.67640	0.70450
C	7.64410	4.48910	1.65720
C	9.01160	4.39560	1.86780
C	9.76970	3.49680	1.12200
C	9.15700	2.68810	0.17020
C	7.78820	2.77170	-0.03760
O	4.86980	4.65980	1.02360
C	1.66020	4.61090	3.09480
O	3.18380	2.77460	3.07660
C	1.85490	4.86630	4.45410

C	1.19750	5.92380	5.06550
C	0.34330	6.73470	4.32260
C	0.14810	6.48510	2.96720
C	0.80300	5.42660	2.35200
H	4.93410	0.90470	2.38550
H	6.72300	1.78930	3.81740
H	9.05520	1.01610	3.51560
H	9.58900	-0.64850	1.76290
H	7.78660	-1.52990	0.30960
H	7.04260	5.18170	2.22990
H	9.48710	5.02110	2.61220
H	10.83720	3.42250	1.28750
H	9.74460	1.98210	-0.40150
H	7.30920	2.13340	-0.76490
H	2.52240	4.23050	5.01930
H	1.35060	6.11730	6.11940
H	-0.16880	7.56090	4.79950
H	-0.51400	7.11660	2.38900
H	0.65440	5.23380	1.29970
C	3.82080	2.17420	-4.18970
C	4.31410	2.97510	-5.21680
C	3.56870	0.82230	-4.40510
C	3.83340	0.27490	-5.65830
C	4.32870	1.06310	-6.68980
C	4.56330	2.41760	-6.46310
H	4.50050	4.02350	-5.02310
H	3.15490	0.20300	-3.62270
H	3.63870	-0.77730	-5.82380
H	4.52680	0.62970	-7.66140
H	4.94830	3.04370	-7.25810

Phenyl β-D-Galf **6**, C2-endo, +60°/180°

	**X**	**Y**	**Z**
C	4.32040	3.09720	-0.96930

H	4.20420	4.17680	-1.00370
C	3.33380	2.45420	0.01760
H	3.87050	2.02890	0.85700
C	2.59260	1.39070	-0.81490
C	2.32140	0.03550	-0.16280
H	2.13580	-0.67210	-0.97790
O	1.14860	0.21410	0.62670
C	3.99400	2.43190	-2.30390
H	4.86970	2.23990	-2.92220
H	1.62330	1.80260	-1.10820
O	3.08880	3.29790	-2.96260
O	5.67970	2.75910	-0.66590
O	2.41570	3.43480	0.50290
O	3.40800	1.19970	-1.97950
C	3.43320	-0.50830	0.72540
H	3.15060	-1.49020	1.11130
H	3.62210	0.14900	1.57230
O	4.61470	-0.63650	-0.07840
H	0.88150	-0.63950	0.99040
C	6.22150	3.37200	0.40820
C	2.18310	3.48570	1.83200
C	5.78150	-0.83050	0.55380
C	6.92350	-0.89200	-0.39230
C	6.77210	-0.57390	-1.74410
C	7.86800	-0.63100	-2.59420
C	9.11310	-1.01400	-2.10360
C	9.26470	-1.33430	-0.75760
C	8.17490	-1.26820	0.09750
O	5.87370	-0.94270	1.75790
C	7.64700	3.02230	0.60400
C	8.25150	3.37270	1.81390
C	9.58870	3.07710	2.03260
C	10.33210	2.44400	1.03920
C	9.73420	2.10050	-0.16910
C	8.39260	2.38060	-0.38700

O	5.58670	4.12110	1.11780
C	1.22970	4.56540	2.18790
O	2.69600	2.73160	2.62770
C	0.89320	4.73100	3.53340
C	0.00730	5.72920	3.91170
C	-0.54810	6.56790	2.94880
C	-0.21580	6.40590	1.60680
C	0.67060	5.40840	1.22390
H	5.80480	-0.27120	-2.11790
H	7.75170	-0.37460	-3.63940
H	9.96600	-1.05790	-2.76910
H	10.23420	-1.62530	-0.37450
H	8.28130	-1.50400	1.14740
H	7.66360	3.86990	2.57330
H	10.05280	3.34130	2.97400
H	11.37730	2.21740	1.20860
H	10.30950	1.60530	-0.93900
H	7.92550	2.10300	-1.31960
H	1.33150	4.07360	4.27140
H	-0.25050	5.85440	4.95540
H	-1.23950	7.34700	3.24410
H	-0.64740	7.05770	0.85820
H	0.92930	5.28180	0.18280
C	2.71210	3.00970	-4.25210
C	1.87900	3.95060	-4.85660
C	3.11320	1.87420	-4.95250
C	2.68400	1.69990	-6.26660
C	1.85800	2.63380	-6.87900
C	1.45590	3.76020	-6.16390
H	1.57810	4.82360	-4.29190
H	3.73480	1.12270	-4.49010
H	2.99850	0.81610	-6.80750
H	1.52730	2.48630	-7.89870
H	0.81090	4.49680	-6.62620

Phenyl β-D-Galf **6**, C2-endo, -60°/+60°

	**X**	**Y**	**Z**
C	3.64420	3.41780	-0.72290
H	3.54040	4.48370	-0.91600
C	2.64760	2.97570	0.34080
H	3.08710	2.23390	1.00230
C	1.46980	2.38670	-0.46650
C	1.19340	0.92720	-0.13210
H	0.86350	0.89040	0.91110
O	2.41030	0.19930	-0.28970
C	3.22930	2.63870	-1.96170
H	3.68240	1.64370	-1.98330
H	0.56280	2.96540	-0.29150
O	3.57440	3.35240	-3.10750
O	4.99490	3.10550	-0.39260
O	2.27280	4.12890	1.10890
O	1.81720	2.55060	-1.85190
C	0.10470	0.36400	-1.02210
H	0.41590	0.35690	-2.06510
H	-0.82090	0.93320	-0.92250
O	-0.11520	-0.99010	-0.57720
H	2.24490	-0.72120	-0.05020
C	5.77890	4.09320	0.09490
C	1.64750	3.90780	2.28030
C	-0.94190	-1.74960	-1.31350
C	-1.07500	-3.13000	-0.78110
C	-0.39200	-3.55220	0.36250
C	-0.54700	-4.85340	0.82160
C	-1.38160	-5.73810	0.14470
C	-2.06390	-5.32040	-0.99500
C	-1.91170	-4.02110	-1.45680
O	-1.51040	-1.33670	-2.30190
C	7.15960	3.62550	0.36800
C	8.08270	4.55090	0.86090

C	9.38600	4.15880	1.12850
C	9.77490	2.84020	0.90700
C	8.85780	1.91450	0.41760
C	7.55260	2.30270	0.14730
O	5.38430	5.22210	0.27980
C	1.38680	5.16120	3.02820
O	1.34040	2.79810	2.66080
C	0.66080	5.08160	4.21890
C	0.40070	6.22960	4.95270
C	0.86610	7.46280	4.50310
C	1.59160	7.54580	3.31810
C	1.85200	6.40000	2.57880
H	0.25490	-2.86490	0.88810
H	-0.01670	-5.17770	1.70770
H	-1.50050	-6.75220	0.50510
H	-2.71340	-6.00760	-1.52170
H	-2.43590	-3.68390	-2.34050
H	7.76770	5.57160	1.02860
H	10.09870	4.87890	1.50920
H	10.79230	2.53420	1.11600
H	9.16000	0.88950	0.24600
H	6.84030	1.58480	-0.23220
H	0.30660	4.11760	4.55720
H	-0.16350	6.16500	5.87400
H	0.66400	8.35850	5.07680
H	1.95540	8.50400	2.97020
H	2.41800	6.46230	1.66070
C	3.58620	2.68410	-4.31220
C	4.24920	3.32570	-5.35550
C	2.96840	1.45320	-4.51630
C	3.03530	0.86260	-5.77600
C	3.69590	1.49170	-6.82410
C	4.29920	2.72870	-6.60750
H	4.71990	4.28250	-5.17030
H	2.42750	0.96480	-3.71880

H	2.55460	-0.09490	-5.93330
H	3.73800	1.02720	-7.80060
H	4.81670	3.23050	-7.41540

Phenyl β-D-Galf **6**, C2-endo, -60°/-60°

	**X**	**Y**	**Z**
C	3.76380	2.74960	-0.80980
H	3.27890	3.65410	-1.17040
C	3.01560	2.19010	0.38790
H	3.67220	1.59890	1.02230
C	1.96990	1.30130	-0.28400
C	1.37610	0.22660	0.61150
H	0.99300	0.74850	1.49700
O	2.41100	-0.67700	0.97930
C	3.65630	1.63600	-1.85360
H	4.60300	1.11290	-1.99170
H	1.15590	1.92460	-0.66800
O	3.24910	2.21570	-3.07240
O	5.13880	3.01420	-0.53140
O	2.42690	3.25190	1.13860
O	2.70400	0.71340	-1.36650
C	0.16880	-0.48340	0.01120
H	-0.64000	0.22840	-0.14560
H	-0.16090	-1.26610	0.69310
O	0.46840	-1.15770	-1.22390
H	2.16360	-1.10370	1.80810
C	5.51020	4.29440	-0.30310
C	2.42120	3.16510	2.48580
C	0.18330	-0.52430	-2.37470
C	0.55200	-1.32570	-3.56820
C	1.21330	-2.55100	-3.46130
C	1.52870	-3.26980	-4.60650
C	1.18600	-2.77180	-5.85970
C	0.53380	-1.54740	-5.96920

C	0.21960	-0.82540	-4.82830
O	-0.33600	0.57090	-2.42530
C	6.96150	4.41030	-0.01870
C	7.48090	5.67950	0.24760
C	8.83180	5.83530	0.52110
C	9.67220	4.72490	0.53030
C	9.15880	3.45860	0.26530
C	7.80720	3.29790	-0.00860
O	4.73780	5.22530	-0.33150
C	1.86370	4.38450	3.11940
O	2.82680	2.19780	3.09110
C	1.72120	4.39980	4.50900
C	1.21070	5.52270	5.14360
C	0.84180	6.63730	4.39470
C	0.98450	6.62690	3.01010
C	1.49280	5.50440	2.37040
H	1.48170	-2.93320	-2.48730
H	2.04470	-4.21750	-4.52160
H	1.43690	-3.33250	-6.75130
H	0.27980	-1.15270	-6.94430
H	-0.28080	0.13020	-4.89990
H	6.81780	6.53350	0.23790
H	9.23030	6.82020	0.72730
H	10.72660	4.84670	0.74410
H	9.81210	2.59570	0.27220
H	7.40800	2.31540	-0.21340
H	2.01300	3.52860	5.07900
H	1.10040	5.53070	6.22020
H	0.44430	7.51400	4.89040
H	0.70050	7.49430	2.42850
H	1.60780	5.49630	1.29650
C	3.38790	1.47820	-4.22560
C	3.17060	2.17230	-5.41420
C	3.72000	0.12590	-4.25180
C	3.86430	-0.51370	-5.47900

C	3.66150	0.17120	-6.66970
C	3.30510	1.51690	-6.63010
H	2.90490	3.22060	-5.36890
H	3.84030	-0.43840	-3.33940
H	4.11370	-1.56620	-5.49400
H	3.76350	-0.33990	-7.61800
H	3.13520	2.06270	-7.54980

Phenyl β-D-Galf **6**, C2-endo, -60°/180°

	**X**	**Y**	**Z**
C	3.76330	2.74970	-0.81070
H	3.27790	3.65370	-1.17160
C	3.01530	2.19000	0.38710
H	3.67200	1.59820	1.02090
C	1.96940	1.30160	-0.28500
C	1.37490	0.22700	0.61020
H	0.99190	0.74880	1.49580
O	2.40910	-0.67730	0.97790
C	3.65640	1.63580	-1.85420
H	4.60300	1.11240	-1.99160
H	1.15580	1.92520	-0.66910
O	3.24970	2.21510	-3.07330
O	5.13820	3.01510	-0.53240
O	2.42770	3.25180	1.13830
O	2.70360	0.71350	-1.36730
C	0.16730	-0.48210	0.00940
H	-0.64090	0.23010	-0.14820
H	-0.16340	-1.26440	0.69120
O	0.46740	-1.15720	-1.22520
H	2.16140	-1.10440	1.80640
C	5.50860	4.29540	-0.30270
C	2.42230	3.16450	2.48550
C	0.18300	-0.52410	-2.37660
C	0.55150	-1.32650	-3.56940

C	1.21210	-2.55210	-3.46180
C	1.52730	-3.27170	-4.60650
C	1.18490	-2.77430	-5.86000
C	0.53330	-1.54960	-5.97040
C	0.21940	-0.82680	-4.82990
O	-0.33550	0.57140	-2.42780
C	6.95960	4.41210	-0.01740
C	7.47780	5.68140	0.25100
C	8.82840	5.83790	0.52550
C	9.66970	4.72830	0.53370
C	9.15750	3.46190	0.26660
C	7.80630	3.30040	-0.00830
O	4.73560	5.22580	-0.33070
C	1.86650	4.38420	3.11980
O	2.82700	2.19640	3.09020
C	1.72440	4.39930	4.50940
C	1.21540	5.52250	5.14450
C	0.84750	6.63780	4.39610
C	0.98970	6.62760	3.01150
C	1.49650	5.50480	2.37130
H	1.48030	-2.93380	-2.48750
H	2.04270	-4.21970	-4.52100
H	1.43560	-3.33570	-6.75120
H	0.27960	-1.15540	-6.94560
H	-0.28050	0.12900	-4.90220
H	6.81400	6.53480	0.24210
H	9.22590	6.82290	0.73340
H	10.72380	4.85060	0.74830
H	9.81160	2.59950	0.27280
H	7.40800	2.31780	-0.21460
H	2.01550	3.52750	5.07910
H	1.10550	5.53030	6.22120
H	0.45110	7.51480	4.89220
H	0.70640	7.49560	2.43030
H	1.61120	5.49690	1.29730

C	3.38830	1.47720	-4.22620
C	3.17090	2.17100	-5.41510
C	3.72040	0.12500	-4.25190
C	3.86440	-0.51510	-5.47900
C	3.66150	0.16930	-6.66990
C	3.30520	1.51500	-6.63070
H	2.90520	3.21930	-5.37010
H	3.84070	-0.43890	-3.33920
H	4.11380	-1.56770	-5.49370
H	3.76340	-0.34210	-7.61800
H	3.13510	2.06060	-7.55050

Phenyl β-D-Galf **6**, C2-endo, 180°/+60°

	**X**	**Y**	**Z**
C	4.34230	3.09160	-0.78900
H	3.93880	4.07050	-1.03580
C	3.52620	2.45120	0.33350
H	4.17010	1.82680	0.95230
C	2.51240	1.60730	-0.43620
C	1.92740	0.42610	0.31270
H	2.73930	-0.27660	0.53540
O	0.97660	-0.18080	-0.55560
C	4.16850	2.12800	-1.96840
H	5.10480	1.65750	-2.26850
H	1.69800	2.24320	-0.80130
O	3.63430	2.87140	-3.04410
O	5.72840	3.20320	-0.46810
O	2.84210	3.40460	1.14490
O	3.28850	1.11320	-1.53470
C	1.27170	0.85410	1.61550
H	0.48420	1.58790	1.44320
H	1.99440	1.26050	2.32080
O	0.69680	-0.34280	2.17390
H	0.57820	-0.92470	-0.08550

C	6.16820	4.39120	0.00580
C	3.45890	3.82910	2.27150
C	-0.00270	-0.20310	3.31310
C	-0.56160	-1.48890	3.80170
C	-0.35850	-2.69190	3.12040
C	-0.90100	-3.86960	3.61690
C	-1.64700	-3.85400	4.79190
C	-1.85120	-2.65680	5.47320
C	-1.31080	-1.47800	4.98040
O	-0.14760	0.86570	3.86620
C	7.60540	4.35170	0.36570
C	8.19220	5.51890	0.86020
C	9.53290	5.52710	1.21550
C	10.29530	4.36970	1.08020
C	9.71410	3.20400	0.58930
C	8.37260	3.19140	0.23220
O	5.45660	5.36370	0.11380
C	2.60610	4.74270	3.06530
O	4.57640	3.48130	2.57910
C	3.16830	5.36600	4.18180
C	2.40130	6.21960	4.96070
C	1.06780	6.45080	4.63240
C	0.50320	5.82900	3.52240
C	1.26850	4.97830	2.73660
H	0.22150	-2.70420	2.20900
H	-0.74200	-4.80010	3.08740
H	-2.06880	-4.77390	5.17680
H	-2.43090	-2.64380	6.38710
H	-1.46220	-0.54190	5.50010
H	7.58840	6.41010	0.96230
H	9.98410	6.43330	1.59840
H	11.34170	4.37600	1.35840
H	10.30660	2.30430	0.48540
H	7.92010	2.28680	-0.14680
H	4.20420	5.17550	4.42670

H	2.84050	6.70390	5.82320
H	0.46870	7.11510	5.24240
H	-0.53410	6.00630	3.26990
H	0.83140	4.49270	1.87640
C	3.60150	2.29640	-4.29250
C	3.27320	3.15570	-5.34050
C	3.86480	0.95160	-4.54280
C	3.81200	0.48170	-5.85340
C	3.49000	1.33070	-6.90460
C	3.21710	2.67110	-6.63910
H	3.07030	4.19610	-5.12120
H	4.08830	0.26700	-3.73840
H	4.01770	-0.56430	-6.04380
H	3.44700	0.95420	-7.91820
H	2.96310	3.34520	-7.44750

Phenyl β-D-Galf **6**, C2-endo, 180°/-60°

	**X**	**Y**	**Z**
C	4.62710	3.39370	-0.60140
H	4.39320	4.44530	-0.73790
C	3.77520	2.76850	0.51680
H	4.41940	2.39150	1.30700
C	3.00020	1.63780	-0.18390
C	2.81890	0.36080	0.61880
H	3.81020	-0.07270	0.79910
O	2.03240	-0.51100	-0.18770
C	4.28680	2.55930	-1.83600
H	5.14680	2.34550	-2.46920
H	2.02150	2.01430	-0.48990
O	3.30540	3.28980	-2.54720
O	6.02870	3.22750	-0.35770
O	2.85990	3.72130	1.05690
O	3.79200	1.34140	-1.34310
C	2.18350	0.59700	1.97880

H	2.85220	1.14750	2.63970
H	1.94790	-0.35920	2.44870
O	0.97140	1.34810	1.78520
H	2.01830	-1.38330	0.22570
C	6.59850	4.10350	0.49860
C	2.90360	3.98630	2.38150
C	0.33050	1.78120	2.88190
C	-0.85550	2.60920	2.55070
C	-1.26620	2.81230	1.23070
C	-2.37020	3.60940	0.96450
C	-3.06490	4.21110	2.01000
C	-2.65540	4.01280	3.32500
C	-1.55540	3.21290	3.59570
O	0.70420	1.52740	4.00760
C	8.04630	3.85020	0.68920
C	8.75430	4.69070	1.55160
C	10.11060	4.48940	1.76080
C	10.76760	3.44750	1.11110
C	10.06550	2.60700	0.25190
C	8.70800	2.80500	0.03910
O	5.97200	4.98790	1.03830
C	1.84230	4.93340	2.79320
O	3.70260	3.47630	3.13480
C	1.72280	5.23860	4.15070
C	0.72280	6.09550	4.58450
C	-0.16250	6.65190	3.66560
C	-0.04640	6.35080	2.31250
C	0.95090	5.49270	1.87410
H	-0.71980	2.35140	0.42090
H	-2.68610	3.76630	-0.05880
H	-3.92170	4.83860	1.79910
H	-3.18880	4.48870	4.13770
H	-1.22070	3.05970	4.61210
H	8.23210	5.49520	2.05100
H	10.65600	5.14250	2.42960

H	11.82620	3.29040	1.27490
H	10.57620	1.79690	-0.25240
H	8.16190	2.15260	-0.62660
H	2.41150	4.79230	4.85440
H	0.62830	6.32490	5.63800
H	-0.94920	7.31340	4.00590
H	-0.74280	6.77310	1.60050
H	1.03320	5.24600	0.82620
C	2.88970	2.83070	-3.77390
C	1.99920	3.66220	-4.45230
C	3.30200	1.62850	-4.34410
C	2.82550	1.27700	-5.60540
C	1.94230	2.10090	-6.29150
C	1.52960	3.29560	-5.70510
H	1.69050	4.58990	-3.98810
H	3.96680	0.96060	-3.81810
H	3.14860	0.34200	-6.04570
H	1.57540	1.81560	-7.26880
H	0.84030	3.94830	-6.22600

Phenyl β-D-Galf **6**, C2-endo, 180°/180°

	**X**	**Y**	**Z**
C	4.83600	2.98590	-0.48150
H	4.70520	4.06310	-0.46780
C	3.85790	2.25450	0.44960
H	4.42450	1.70960	1.20120
C	3.07940	1.30600	-0.47780
C	2.75550	-0.06340	0.09390
H	3.68620	-0.54660	0.41100
O	2.13300	-0.80120	-0.95370
C	4.53680	2.39610	-1.86270
H	5.42100	2.23180	-2.47640
H	2.14900	1.79100	-0.78980
O	3.64600	3.29530	-2.49200

O	6.16320	2.64130	-0.06940
O	2.92050	3.11330	1.10240
O	3.94500	1.14700	-1.60910
C	1.77580	0.00360	1.26200
H	1.40860	-0.99990	1.48290
H	0.92550	0.63890	1.01570
O	2.44950	0.52100	2.42150
H	2.06900	-1.72600	-0.68380
C	7.16250	3.42830	-0.51650
C	3.39860	3.87010	2.11390
C	1.66840	0.91940	3.44130
C	2.44060	1.53690	4.54630
C	3.83650	1.55950	4.55230
C	4.51390	2.18660	5.58870
C	3.80370	2.79530	6.61860
C	2.41130	2.77120	6.61660
C	1.73140	2.14120	5.58670
O	0.46200	0.79800	3.43080
C	8.48160	3.03290	0.03310
C	9.61360	3.72320	-0.40650
C	10.86680	3.38850	0.08500
C	10.99650	2.36460	1.01980
C	9.87060	1.67570	1.46200
C	8.61460	2.00590	0.97120
O	6.97830	4.35010	-1.28090
C	2.32250	4.51830	2.89690
O	4.58260	3.97100	2.34600
C	2.68820	5.32960	3.97240
C	1.71230	5.90920	4.76820
C	0.36650	5.68000	4.49600
C	-0.00220	4.87350	3.42290
C	0.97120	4.29340	2.62240
H	4.38680	1.09980	3.74470
H	5.59590	2.20870	5.58770
H	4.33450	3.29290	7.42040

H	1.85860	3.25290	7.41250
H	0.65090	2.12750	5.56450
H	9.49870	4.51700	-1.13170
H	11.74190	3.92460	-0.25870
H	11.97470	2.10430	1.40410
H	9.97160	0.88140	2.19020
H	7.73960	1.47370	1.31480
H	3.73710	5.48480	4.18250
H	1.99870	6.53080	5.60650
H	-0.39530	6.12660	5.12240
H	-1.04840	4.69130	3.21470
H	0.68760	3.65560	1.79830
C	3.30760	3.09200	-3.80790
C	2.53360	4.09630	-4.38840
C	3.68880	1.97890	-4.55350
C	3.30000	1.89090	-5.88860
C	2.53330	2.88830	-6.47750
C	2.14950	3.99120	-5.71720
H	2.24800	4.95050	-3.78820
H	4.26190	1.17940	-4.10920
H	3.59880	1.02440	-6.46520
H	2.23380	2.80770	-7.51430
H	1.55080	4.77670	-6.16110

Phenyl β-D-Galf **6**, C1-exo, +60°/+60°

	**X**	**Y**	**Z**
C	4.08100	2.32250	-1.01440
H	3.97060	3.34510	-1.37230
C	3.05510	2.02740	0.07250
H	3.43600	1.27070	0.75320
C	1.82790	1.55280	-0.72060
C	1.11630	0.33290	-0.13660
H	0.23140	0.13880	-0.75390
O	0.72800	0.70050	1.18160

C	3.70240	1.35080	-2.11880
H	4.15990	0.36710	-1.96410
H	1.10660	2.36960	-0.77780
O	4.09620	1.86440	-3.35310
O	5.42610	2.07970	-0.61370
O	2.70580	3.19240	0.82290
O	2.28580	1.26010	-2.05000
C	1.97290	-0.91950	-0.14060
H	2.86120	-0.81360	0.48200
H	2.27020	-1.18890	-1.15330
O	1.14200	-1.96070	0.40970
H	0.28440	-0.05540	1.58740
C	6.13390	3.12460	-0.12790
C	3.35770	3.40740	1.98520
C	1.70790	-3.16920	0.56810
C	7.50640	2.73500	0.27130
C	7.97350	1.42450	0.14070
C	9.26750	1.10910	0.53200
C	10.09920	2.09550	1.05460
C	9.63580	3.40200	1.18720
C	8.34370	3.72170	0.79750
O	5.67900	4.24220	-0.03740
C	0.78420	-4.15700	1.18090
C	-0.51380	-3.81580	1.57020
C	-1.33880	-4.77290	2.14560
C	-0.87560	-6.07180	2.33460
C	0.41710	-6.41520	1.94700
C	1.24480	-5.46160	1.37250
O	2.85060	-3.40620	0.23980
C	2.92880	4.66430	2.64360
C	1.94770	5.49310	2.09310
C	1.58440	6.66340	2.74570
C	2.19670	7.01290	3.94590
C	3.17590	6.18970	4.49610
C	3.54130	5.01910	3.84790

O	4.19490	2.65100	2.42500
H	7.32680	0.65940	-0.26330
H	9.62750	0.09360	0.43010
H	11.10780	1.84610	1.35920
H	10.28180	4.16900	1.59450
H	7.97100	4.73180	0.89640
H	-0.87300	-2.80750	1.42360
H	-2.34330	-4.50550	2.44730
H	-1.52120	-6.81600	2.78360
H	0.77790	-7.42500	2.09350
H	2.25090	-5.71510	1.06810
H	1.47500	5.22180	1.16050
H	0.82410	7.30380	2.31760
H	1.91190	7.92680	4.45180
H	3.65350	6.46180	5.42840
H	4.30140	4.37190	4.26320
C	4.17680	1.00540	-4.42790
C	4.92220	1.45990	-5.51280
C	3.54870	-0.23620	-4.46970
C	3.68930	-1.02820	-5.60680
C	4.43290	-0.58710	-6.69460
C	5.04490	0.66350	-6.64280
H	5.39940	2.42950	-5.45450
H	2.94210	-0.57860	-3.64380
H	3.20180	-1.99450	-5.63800
H	4.53260	-1.20770	-7.57540
H	5.62650	1.01970	-7.48370

Phenyl β-D-Galf **6**, C1-exo, +60°/-60°

	**X**	**Y**	**Z**
C	4.05880	2.33450	-0.88890
H	4.07440	3.37070	-1.22450
C	3.00860	2.15270	0.20590
H	3.36990	1.49490	0.98260

C	1.79040	1.56600	-0.53190
C	1.25460	0.24620	0.03750
H	0.35810	-0.00210	-0.54370
O	0.91370	0.52960	1.38870
C	3.58520	1.43290	-2.01960
H	4.01070	0.42810	-1.92690
H	0.97510	2.28870	-0.50020
O	3.95270	1.98420	-3.24670
O	5.37410	1.91240	-0.52830
O	2.65360	3.40880	0.79430
O	2.17250	1.39820	-1.90280
C	2.16780	-0.95960	-0.10060
H	2.40570	-1.14060	-1.14820
H	1.66840	-1.84650	0.29430
O	3.37650	-0.73570	0.64660
H	0.44700	-0.22820	1.76220
C	6.13550	2.76740	0.18460
C	3.02140	3.62910	2.07350
C	4.36420	-1.63740	0.51740
C	7.49690	2.23700	0.42670
C	7.95700	1.07400	-0.19390
C	9.24230	0.61820	0.05990
C	10.06900	1.31210	0.93770
C	9.61060	2.46900	1.56260
C	8.32970	2.93360	1.30490
O	5.72820	3.83950	0.57300
C	5.53710	-1.31900	1.36920
C	5.53990	-0.23780	2.25350
C	6.66160	0.01970	3.02930
C	7.78140	-0.79850	2.93170
C	7.78060	-1.87980	2.05490
C	6.66420	-2.13930	1.27520
O	4.28510	-2.59660	-0.22070
C	2.66580	4.99580	2.52880
C	2.07190	5.93050	1.67690

C	1.76430	7.19990	2.14790
C	2.04640	7.54300	3.46700
C	2.63930	6.61400	4.31820
C	2.94870	5.34500	3.85130
O	3.57070	2.80010	2.76460
H	7.30910	0.52780	-0.86300
H	9.59510	-0.28630	-0.41750
H	11.06860	0.94850	1.14000
H	10.25160	3.00620	2.24950
H	7.95980	3.83010	1.78320
H	4.67450	0.40190	2.33720
H	6.66290	0.86580	3.70380
H	8.65820	-0.58850	3.53050
H	8.65470	-2.51280	1.97300
H	6.65350	-2.97240	0.58590
H	1.85590	5.66410	0.65270
H	1.30530	7.92240	1.48550
H	1.80560	8.53380	3.83130
H	2.85990	6.88020	5.34390
H	3.41020	4.61490	4.50180
C	3.97200	1.16740	-4.35620
C	4.69220	1.64550	-5.44810
C	3.30990	-0.05510	-4.42500
C	3.39090	-0.80450	-5.59630
C	4.10890	-0.33950	-6.69130
C	4.75530	0.89220	-6.61200
H	5.19720	2.59940	-5.36850
H	2.72250	-0.41550	-3.59310
H	2.87700	-1.75610	-5.64800
H	4.16220	-0.92690	-7.59850
H	5.31770	1.26690	-7.45790

Phenyl β-D-Galf **6**, C1-exo, +60°/180°

	X	Y	Z
C	3.78690	2.29710	-0.95680
H	3.79160	3.31930	-1.33200
C	2.78300	2.14950	0.17940
H	3.12560	1.39830	0.88590
C	1.48390	1.73800	-0.53660
C	0.78400	0.52700	0.08910
H	-0.14600	0.35900	-0.46350
O	0.51580	0.91720	1.43230
C	3.24000	1.36380	-2.02370
H	3.54820	0.33110	-1.84470
H	0.78220	2.57240	-0.51450
O	3.63490	1.77490	-3.29430
O	5.10750	1.88760	-0.61200
O	2.56880	3.37670	0.88310
O	1.83050	1.49150	-1.90600
C	1.58070	-0.77520	0.08940
H	1.10740	-1.48470	0.77110
H	2.61270	-0.63690	0.41280
O	1.55580	-1.31580	-1.24190
H	-0.09920	0.28730	1.82820
C	5.97010	2.83630	-0.18610
C	3.27400	3.57710	2.01610
C	2.32700	-2.38820	-1.48230
C	7.30060	2.27410	0.14530
C	7.58950	0.91490	-0.00350
C	8.85150	0.43640	0.32170
C	9.82790	1.30770	0.79650
C	9.54220	2.66230	0.94720
C	8.28290	3.14490	0.62280
O	5.67110	4.00520	-0.09320
C	2.23600	-2.84510	-2.89070
C	1.48750	-2.14980	-3.84360
C	1.42850	-2.61390	-5.14920
C	2.11280	-3.76980	-5.51130

C	2.86450	-4.46150	-4.56620
C	2.92790	-4.00010	-3.25930
O	3.01550	-2.90930	-0.62970
C	2.97460	4.88870	2.63950
C	2.04020	5.77360	2.09520
C	1.79580	6.99070	2.71670
C	2.48100	7.33140	3.87950
C	3.41430	6.45240	4.42310
C	3.66070	5.23490	3.80590
O	4.05820	2.76870	2.46130
H	6.83060	0.23940	-0.37020
H	9.07370	-0.61640	0.20520
H	10.81090	0.93120	1.04980
H	10.30070	3.33960	1.31770
H	8.04700	4.19400	0.73590
H	0.96710	-1.24600	-3.56320
H	0.85860	-2.06700	-5.88840
H	2.06840	-4.12610	-6.53280
H	3.40190	-5.35760	-4.84890
H	3.50900	-4.52700	-2.51490
H	1.51030	5.50870	1.19190
H	1.07100	7.67440	2.29390
H	2.28870	8.28170	4.36140
H	3.94850	6.71740	5.32630
H	4.38370	4.54370	4.21660
C	3.82180	0.80580	-4.26860
C	4.64170	-0.29740	-4.05680
C	3.21790	1.01730	-5.50120
C	3.43780	0.11290	-6.53390
C	4.24490	-1.00130	-6.33150
C	4.84140	-1.20290	-5.09210
H	5.12600	-0.44560	-3.10090
H	2.58660	1.88550	-5.63830
H	2.96540	0.27540	-7.49450
H	4.40120	-1.71290	-7.13140

H	5.46810	-2.06920	-4.92510

Phenyl β-D-Galf **6**, C1-exo, -60°/+60°

	**X**	**Y**	**Z**
C	4.49030	2.04450	-1.10020
H	4.28870	3.05740	-1.44160
C	3.45400	1.61850	-0.06500
H	3.88020	0.89330	0.62370
C	2.38090	0.97210	-0.93390
C	1.46110	0.01280	-0.20590
H	0.87870	0.61360	0.50370
O	2.24670	-0.94500	0.49140
C	4.29050	1.04840	-2.24830
H	5.13590	0.37010	-2.36670
H	1.76810	1.75020	-1.40350
O	4.10440	1.79730	-3.43060
O	5.83350	1.95120	-0.62360
O	2.89340	2.71370	0.65650
O	3.15030	0.27940	-1.92460
C	0.50430	-0.65540	-1.17440
H	1.03800	-1.29370	-1.87670
H	-0.07890	0.08350	-1.72540
O	-0.37820	-1.46300	-0.36970
H	1.64720	-1.53150	0.96960
C	6.37940	3.06250	-0.08120
C	3.44350	3.02520	1.85100
C	-1.27030	-2.22670	-1.02130
C	7.74910	2.82250	0.43110
C	8.35650	1.56580	0.36590
C	9.63860	1.39030	0.86850
C	10.31910	2.46340	1.43670
C	9.71590	3.71690	1.50340
C	8.43480	3.89660	1.00330
O	5.79970	4.12360	-0.02800

C	-2.10620	-3.03990	-0.10150
C	-1.95270	-2.98180	1.28620
C	-2.75690	-3.76250	2.10570
C	-3.71530	-4.60360	1.54740
C	-3.87060	-4.66390	0.16500
C	-3.06950	-3.88490	-0.65720
O	-1.36870	-2.23650	-2.22970
C	2.78320	4.19030	2.48620
C	1.71520	4.85870	1.88190
C	1.13270	5.94800	2.51560
C	1.61160	6.37590	3.75050
C	2.67670	5.71270	4.35450
C	3.26150	4.62380	3.72510
O	4.37010	2.41220	2.33100
H	7.82620	0.73320	-0.07280
H	10.10710	0.41590	0.81820
H	11.31870	2.32310	1.82840
H	10.24430	4.55130	1.94610
H	7.95360	4.86380	1.05070
H	-1.20900	-2.32870	1.71900
H	-2.63610	-3.71520	3.18030
H	-4.34070	-5.21150	2.18900
H	-4.61560	-5.31760	-0.27010
H	-3.18000	-3.92260	-1.73220
H	1.34580	4.52740	0.92250
H	0.30560	6.46420	2.04560
H	1.15580	7.22640	4.24160
H	3.05040	6.04600	5.31400
H	4.09030	4.10140	4.18250
C	4.13720	1.14180	-4.63890
C	4.16980	1.96180	-5.76620
C	4.13170	-0.24420	-4.77780
C	4.17430	-0.79880	-6.05530
C	4.21010	0.00950	-7.18440
C	4.20440	1.39460	-7.03170

H	4.17170	3.03600	-5.63330
H	4.07690	-0.89150	-3.91530
H	4.17020	-1.87670	-6.15910
H	4.23810	-0.43160	-8.17210
H	4.23130	2.03820	-7.90200

Phenyl β-D-Galf **6**, C1-exo, -60°/-60°

	**X**	**Y**	**Z**
C	4.02770	1.85590	-0.95040
H	3.88860	2.81690	-1.44170
C	3.02580	1.69930	0.18630
H	3.40010	1.00810	0.93830
C	1.82170	1.11660	-0.54430
C	0.81830	0.41300	0.35390
H	0.54840	1.14360	1.12820
O	1.45350	-0.71740	0.93780
C	3.66440	0.71480	-1.90340
H	4.39930	-0.09020	-1.87580
H	1.30160	1.91430	-1.08420
O	3.58200	1.24160	-3.20810
O	5.38830	1.70990	-0.54360
O	2.65910	2.93860	0.79080
O	2.42330	0.19940	-1.46660
C	-0.49040	0.05920	-0.34140
H	-0.99360	0.96810	-0.66700
H	-1.13160	-0.48340	0.35200
O	-0.31020	-0.82450	-1.46260
H	0.92940	-1.00670	1.69420
C	6.07060	2.83190	-0.22620
C	3.34190	3.33200	1.88750
C	-0.25670	-0.28080	-2.69150
C	7.45370	2.53950	0.21860
C	7.94960	1.23530	0.29230
C	9.25180	1.01330	0.71930

C	10.06310	2.08730	1.07440
C	9.57090	3.38810	1.00360
C	8.27040	3.61440	0.57760
O	5.59100	3.94070	-0.30240
C	-0.05440	-1.29770	-3.75310
C	0.14720	-2.64740	-3.45710
C	0.32320	-3.56310	-4.48570
C	0.29830	-3.13830	-5.81030
C	0.10510	-1.79260	-6.10780
C	-0.06860	-0.87480	-5.08360
O	-0.38140	0.90710	-2.90320
C	2.87740	4.64490	2.39560
C	1.84650	5.35690	1.77680
C	1.45040	6.58570	2.28740
C	2.07920	7.10970	3.41330
C	3.10800	6.40320	4.03110
C	3.50650	5.17490	3.52460
O	4.22920	2.67540	2.38440
H	7.31840	0.40260	0.01830
H	9.63420	0.00230	0.77590
H	11.07810	1.91070	1.40740
H	10.20090	4.22320	1.28110
H	7.87480	4.61910	0.51930
H	0.17050	-2.97370	-2.42770
H	0.48280	-4.60830	-4.25380
H	0.44030	-3.85390	-6.61040
H	0.10040	-1.45930	-7.13720
H	-0.21190	0.17440	-5.30100
H	1.36110	4.95070	0.90150
H	0.65190	7.13550	1.80630
H	1.76870	8.06870	3.80830
H	3.59840	6.81110	4.90550
H	4.30520	4.61730	3.99380
C	3.58590	0.36430	-4.26810
C	3.71980	0.94320	-5.52860

C	3.46440	-1.01700	-4.13780
C	3.51200	-1.81270	-5.27830
C	3.65700	-1.24740	-6.53810
C	3.75200	0.13680	-6.65770
H	3.80370	2.01960	-5.60550
H	3.30610	-1.47590	-3.17370
H	3.40790	-2.88440	-5.17300
H	3.67920	-1.87520	-7.41910
H	3.85720	0.59330	-7.63390

Phenyl β-D-Galf **6**, C1-exo, -60°/180°

	**X**	**Y**	**Z**
C	3.99580	2.21270	-1.03100
H	3.85650	3.22070	-1.41960
C	2.97430	1.91210	0.05500
H	3.35260	1.14050	0.72120
C	1.75160	1.43330	-0.73710
C	1.06200	0.23580	-0.10200
H	0.76380	0.53290	0.91010
O	1.99390	-0.83850	-0.06180
C	3.64060	1.19680	-2.10420
H	4.09600	0.22150	-1.90240
H	1.03250	2.24820	-0.82740
O	4.05570	1.66660	-3.35070
O	5.34790	2.02070	-0.62510
O	2.60360	3.06490	0.81590
O	2.22600	1.10740	-2.05580
C	-0.17460	-0.19540	-0.87780
H	-0.61460	-1.08610	-0.42810
H	0.07240	-0.40530	-1.91800
O	-1.11110	0.89450	-0.80530
H	1.69840	-1.48490	0.59040
C	6.01940	3.09640	-0.15800
C	3.26500	3.29970	1.96900

C	-2.26790	0.75800	-1.47100
C	7.40790	2.76390	0.23800
C	7.92780	1.47330	0.10910
C	9.23570	1.21280	0.49520
C	10.02840	2.23420	1.01090
C	9.51230	3.52100	1.14190
C	8.20630	3.78590	0.75710
O	5.52480	4.19840	-0.07940
C	-3.13950	1.95430	-1.34420
C	-2.74510	3.08250	-0.62000
C	-3.59140	4.17960	-0.53080
C	-4.83240	4.15750	-1.16110
C	-5.22840	3.03470	-1.88330
C	-4.38530	1.93660	-1.97520
O	-2.55270	-0.23850	-2.10060
C	2.80650	4.54390	2.63240
C	1.79640	5.34400	2.09160
C	1.40470	6.50250	2.74870
C	2.01740	6.86900	3.94360
C	3.02560	6.07480	4.48390
C	3.41940	4.91590	3.83120
O	4.12950	2.56870	2.39820
H	7.31120	0.68140	-0.29030
H	9.63700	0.21270	0.39410
H	11.04790	2.02760	1.31120
H	10.12800	4.31510	1.54380
H	7.79250	4.78010	0.85420
H	-1.78170	3.09930	-0.13150
H	-3.28300	5.05230	0.03010
H	-5.49040	5.01430	-1.08970
H	-6.19310	3.01660	-2.37360
H	-4.68100	1.05910	-2.53360
H	1.32390	5.06020	1.16260
H	0.62210	7.12060	2.32810
H	1.71050	7.77380	4.45300

H	3.50370	6.36040	5.41190
H	4.20210	4.29120	4.23900
C	4.14350	0.77060	-4.39320
C	4.89100	1.18880	-5.49150
C	3.52100	-0.47470	-4.39300
C	3.66890	-1.30610	-5.50070
C	4.41440	-0.90110	-6.60130
C	5.02120	0.35300	-6.59190
H	5.36360	2.16210	-5.46680
H	2.91470	-0.79060	-3.55630
H	3.18500	-2.27470	-5.49920
H	4.51920	-1.55220	-7.45930
H	5.60430	0.68170	-7.44300

Phenyl β-D-Galf **6**, C1-exo, 180°/+60°

	**X**	**Y**	**Z**
C	4.47070	1.95210	-1.08390
H	4.25520	2.97360	-1.38870
C	3.45810	1.49400	-0.03560
H	3.91670	0.77130	0.63900
C	2.37610	0.84400	-0.89520
C	1.50610	-0.18820	-0.20460
H	2.14230	-1.03090	0.09140
O	0.53980	-0.61400	-1.15950
C	4.25020	0.99020	-2.25550
H	5.10350	0.33340	-2.42470
H	1.73700	1.61740	-1.33650
O	4.00830	1.77180	-3.40630
O	5.82190	1.85340	-0.63630
O	2.89820	2.57460	0.70870
O	3.13960	0.18710	-1.91460
C	0.82860	0.37830	1.03250
H	0.21660	1.24730	0.79070
H	1.54840	0.64730	1.80350

O	-0.01650	-0.67770	1.52870
H	-0.03670	-1.26010	-0.73110
C	6.39500	2.96900	-0.12960
C	3.48470	2.90180	1.88280
C	-0.77610	-0.39400	2.60060
C	7.77250	2.71950	0.35620
C	8.36070	1.45310	0.30380
C	9.65190	1.26990	0.77970
C	10.36050	2.34490	1.30860
C	9.77650	3.60800	1.36270
C	8.48640	3.79550	0.88910
O	5.83220	4.03920	-0.08730
C	-1.62620	-1.53810	3.01650
C	-1.61140	-2.75820	2.33550
C	-2.42690	-3.79870	2.75990
C	-3.25900	-3.62850	3.86270
C	-3.27600	-2.41400	4.54360
C	-2.46270	-1.37190	4.12260
O	-0.75380	0.68480	3.15290
C	2.76530	3.99140	2.58110
C	1.55660	4.50480	2.10350
C	0.91220	5.51790	2.80010
C	1.47020	6.02480	3.97020
C	2.67550	5.51620	4.44750
C	3.32100	4.50120	3.75710
O	4.47710	2.34780	2.29820
H	7.80880	0.61920	-0.10460
H	10.10560	0.28810	0.73920
H	11.36720	2.19860	1.67940
H	10.32690	4.44390	1.77470
H	8.02000	4.77030	0.92690
H	-0.96490	-2.89060	1.48010
H	-2.41360	-4.74260	2.23050
H	-3.89390	-4.44170	4.19130
H	-3.92280	-2.28090	5.40110

H	-2.46700	-0.42370	4.64220
H	1.12380	4.10900	1.19650
H	-0.02590	5.91160	2.43090
H	0.96570	6.81590	4.51050
H	3.10970	5.91090	5.35690
H	4.25650	4.09640	4.11770
C	4.02910	1.15790	-4.63640
C	4.02380	2.01490	-5.73620
C	4.04750	-0.22270	-4.82130
C	4.07650	-0.73400	-6.11710
C	4.07520	0.11160	-7.21910
C	4.04500	1.49050	-7.02040
H	4.00740	3.08400	-5.56780
H	4.01970	-0.89900	-3.98000
H	4.09130	-1.80770	-6.25660
H	4.09330	-0.29630	-8.22130
H	4.04250	2.16280	-7.86910

Phenyl β-D-Galf **6**, C1-exo, 180°/-60°

	**X**	**Y**	**Z**
C	4.68360	2.16700	-0.72130
H	4.72920	3.22340	-0.96880
C	3.61400	1.88760	0.34860
H	4.07290	1.41770	1.21520
C	2.61550	0.95020	-0.35260
C	2.00450	-0.14380	0.50640
H	2.80680	-0.82920	0.80550
O	1.05640	-0.81370	-0.32000
C	4.24860	1.31470	-1.91370
H	5.07530	0.81350	-2.41520
H	1.81650	1.54930	-0.79590
O	3.57610	2.18990	-2.80000
O	5.97660	1.70070	-0.31710
O	2.95610	3.09040	0.74320

O	3.39410	0.33290	-1.38780
C	1.36970	0.37790	1.78370
H	2.11990	0.77490	2.46670
H	0.83990	-0.43040	2.29040
O	0.43660	1.41560	1.43310
H	0.74520	-1.60160	0.14270
C	6.66660	2.50190	0.52360
C	2.99020	3.45520	2.04400
C	-0.12750	2.10180	2.43910
C	7.98860	1.93970	0.88800
C	8.42850	0.70460	0.40460
C	9.67550	0.22060	0.77650
C	10.48720	0.96350	1.62910
C	10.05080	2.19420	2.11320
C	8.80550	2.68130	1.74490
O	6.22920	3.55850	0.92160
C	-1.01380	3.18830	1.95370
C	-1.26870	3.37860	0.59320
C	-2.08970	4.41890	0.18220
C	-2.65490	5.27530	1.12290
C	-2.39990	5.08910	2.47810
C	-1.58360	4.04770	2.89340
O	0.08840	1.85580	3.60710
C	2.20780	4.68730	2.29490
C	1.57170	5.38410	1.26430
C	0.82540	6.51660	1.55350
C	0.70630	6.95690	2.86760
C	1.33720	6.26440	3.89730
C	2.08630	5.13270	3.61280
O	3.56960	2.82090	2.89710
H	7.79730	0.12830	-0.25600
H	10.01460	-0.73620	0.40140
H	11.45920	0.58340	1.91710
H	10.68120	2.77170	2.77680
H	8.45400	3.63480	2.11440

H	-0.82200	2.71760	-0.13500
H	-2.28510	4.56550	-0.87220
H	-3.28960	6.09060	0.79910
H	-2.83070	5.76070	3.20930
H	-1.36900	3.89830	3.94230
H	1.65290	5.03220	0.24690
H	0.32410	7.04780	0.75540
H	0.11370	7.83500	3.09110
H	1.23900	6.60340	4.92040
H	2.57400	4.57980	4.40350
C	3.15150	1.71070	-4.01590
C	2.54430	2.64990	-4.84890
C	3.29820	0.39060	-4.43590
C	2.84410	0.02640	-5.70180
C	2.24170	0.95550	-6.54030
C	2.09260	2.27050	-6.10440
H	2.43820	3.66890	-4.49960
H	3.73930	-0.35510	-3.79220
H	2.96030	-1.00090	-6.02410
H	1.88830	0.66030	-7.51960
H	1.62380	3.00670	-6.74530

Phenyl β-D-Galf **6**, C1-exo, 180°/180°

	**X**	**Y**	**Z**
C	4.00370	2.47750	-0.75800
H	4.18810	3.50070	-1.07980
C	2.86830	2.44530	0.26800
H	3.17480	1.94310	1.17670
C	1.71570	1.72740	-0.45770
C	1.43890	0.30260	0.02690
H	2.30050	-0.33840	-0.18880
O	0.29780	-0.13090	-0.70530
C	3.48930	1.64690	-1.92990
H	3.82050	0.60550	-1.84840

H	0.79300	2.30010	-0.36250
O	3.93280	2.19290	-3.13280
O	5.21750	1.88290	-0.29120
O	2.47810	3.78900	0.58490
O	2.07720	1.73810	-1.84460
C	1.14030	0.22820	1.52420
H	0.56050	-0.67090	1.73620
H	0.57640	1.09480	1.87150
O	2.39580	0.15220	2.22010
H	0.19450	-1.08310	-0.58280
C	5.94310	2.60300	0.59060
C	2.42380	4.13650	1.88650
C	2.37560	0.33370	3.55170
C	7.17980	1.90620	1.01410
C	7.51710	0.63800	0.53550
C	8.67490	0.01600	0.98290
C	9.49930	0.65490	1.90500
C	9.16590	1.92020	2.38140
C	8.00950	2.54420	1.93840
O	5.59330	3.69300	0.98440
C	3.73790	0.33760	4.14060
C	4.88220	0.15650	3.35980
C	6.13680	0.17320	3.95340
C	6.25730	0.37690	5.32450
C	5.11930	0.56210	6.10560
C	3.86340	0.54050	5.51650
O	1.34940	0.47090	4.18140
C	2.10190	5.57300	2.07000
C	1.92600	6.43840	0.98740
C	1.63300	7.77700	1.21160
C	1.51470	8.25780	2.51250
C	1.68950	7.39750	3.59350
C	1.98240	6.05950	3.37390
O	2.61390	3.35610	2.79290
H	6.87350	0.14100	-0.17550

H	8.93300	-0.96860	0.61530
H	10.39930	0.16530	2.25490
H	9.80470	2.41570	3.10080
H	7.73320	3.52250	2.30680
H	4.79000	0.00660	2.29410
H	7.01990	0.03220	3.34650
H	7.23770	0.39410	5.78370
H	5.21220	0.72360	7.17190
H	2.97180	0.68390	6.11130
H	2.02000	6.06450	-0.02160
H	1.49820	8.44580	0.37130
H	1.28670	9.30230	2.68390
H	1.59790	7.77080	4.60530
H	2.12170	5.38080	4.20410
C	3.88210	1.41100	-4.26640
C	4.65440	1.84910	-5.33950
C	3.10610	0.26050	-4.37500
C	3.12490	-0.45870	-5.56780
C	3.89410	-0.03380	-6.64420
C	4.65520	1.12710	-6.52470
H	5.24740	2.74770	-5.22870
H	2.47780	-0.06290	-3.55780
H	2.52110	-1.35360	-5.65140
H	3.89820	-0.59670	-7.56840
H	5.25800	1.47040	-7.35590
